# Identification of Pathogen Genomic Differences That Impact Human Immune Response and Disease during Cryptococcus neoformans Infection

**DOI:** 10.1128/mBio.01440-19

**Published:** 2019-07-16

**Authors:** Aleeza C. Gerstein, Katrina M. Jackson, Tami R. McDonald, Yina Wang, Benjamin D. Lueck, Sara Bohjanen, Kyle D. Smith, Andrew Akampurira, David B. Meya, Chaoyang Xue, David R. Boulware, Kirsten Nielsen

**Affiliations:** a Department of Microbiology and Immunology, University of Minnesota, Minneapolis, Minnesota, USA; b Public Health Research Institute, Rutgers University, Newark, New Jersey, USA; c Infectious Diseases Institute and School of Medicine, College of Health Sciences, Makerere University, Kampala, Uganda; d Department of Medicine, University of Minnesota, Minneapolis, Minnesota, USA; Duke University Medical Center

**Keywords:** fungus, HIV, cryptococcosis, meningitis, GWAS, polymorphism, virulence, variant, genome analysis, CNS, pathogenesis, SNP, GWAS

## Abstract

Patient outcomes during infection are due to a complex interplay between the quality of medical care, host immunity factors, and the infecting pathogen’s characteristics. To probe the influence of pathogen genotype on human survival, immune response, and other parameters of disease, we examined Cryptococcus neoformans isolates collected during the Cryptococcal Optimal Antiretroviral Therapy (ART) Timing (COAT) Trial in Uganda. We measured human participants’ survival, meningitis disease parameters, immunologic phenotypes, and pathogen *in vitro* growth characteristics. We compared those clinical data to whole-genome sequences from 38 C. neoformans isolates of the most frequently observed sequence type (ST), ST93, in our Ugandan participant population and to sequences from an additional 18 strains of 9 other sequence types representing the known genetic diversity within the Ugandan *Cryptococcus* clinical isolates. We focused our analyses on 652 polymorphisms that were variable among the ST93 genomes, were not in centromeres or extreme telomeres, and were predicted to have a fitness effect. Logistic regression and principal component analysis identified 40 candidate *Cryptococcus* genes and 3 hypothetical RNAs associated with human survival, immunologic response, or clinical parameters. We infected mice with 17 available KN99α gene deletion strains for these candidate genes and found that 35% (6/17) directly influenced murine survival. Four of the six gene deletions that impacted murine survival were novel. Such bedside-to-bench translational research identifies important candidate genes for future studies on virulence-associated traits in human *Cryptococcus* infections.

## Abstract (precis)

Even with the best available care, mortality rates in cryptococcal meningitis range from 20% to 60%. Disease is often due to infection by the fungus Cryptococcus neoformans and involves a complex interaction between the human host and the fungal pathogen. Although previous studies have suggested genetic differences in the pathogen impact human disease, it has proven quite difficult to identify the specific C. neoformans genes that impact the outcome of the human infection. Here, we take advantage of a Ugandan patient cohort infected with closely related C. neoformans strains to examine the role of pathogen genetic variants on several human disease characteristics. Using a pathogen whole-genome sequencing approach, we showed that 40 C. neoformans genes are associated with human disease. Surprisingly, many of these genes are specific to *Cryptococcus* and have unknown functions. We also show deletion of some of these genes alters disease in a mouse model of infection, confirming their role in disease. These findings are particularly important because they are the first to identify C. neoformans genes associated with human cryptococcal meningitis and lay the foundation for future studies that may lead to new treatment strategies aimed at reducing patient mortality.

## INTRODUCTION

Cryptococcus neoformans is the etiological agent of cryptococcal meningitis, the most common brain infection in sub-Saharan Africa, and is responsible for 15% of AIDS-related deaths (1). As with all fungal pathogens, a major clinical concern is the small number of antifungal drug classes available (*n* = 3) (2, 3). Researchers seek to identify the pathogen virulence factors that influence human health in order to develop novel drug targets to improve patient survival (4). In addition to the virulence factors that are common among all human-pathogenic fungi, such as the ability to grow at 37°C, a number of *Cryptococcus*-specific virulence factors have been identified. The best studied include the polysaccharide capsule, the synthesis of melanin, and the secretion of extracellular enzymes such as phospholipases, laccase, and urease (5). As we have previously discussed (6), there is not a clear quantitative association between *in vitro* virulence factor defects and clinical parameters of disease (7–13); thus, studies clarifying this relationship are required.

Additional potential virulence targets have been identified through reverse genetic screens of the C. neoformans gene knockout collection (14). A screen of 1,201 knockout mutants from 1,180 genes (20% of the protein-coding genes) identified 164 mutants with reduced infectivity and 33 with increased infectivity in a screen for murine lung infectivity (7). Desalermos and colleagues (15) screened the same mutants for virulence in Caenorhabditis elegans and Galleria mellonella infection models and identified 12 mutants through a dual-species stepwise screening approach; all 12 also had attenuated virulence in a murine model (4 overlapped those identified in the original murine lung screen). Many of the identified genes are associated with melanin production (which is not required for killing of C. elegans); thus, the emerging picture is that genes that influence virulence are involved in multiple independent or parallel pathways such as melanization (15).

A complementary tactic to identify novel virulence factors is to use a forward genetics approach to look for an association between strain background and virulence. At a coarse level, there is a clear correlation between *Cryptococcus* variation and human infectivity. C. neoformans var. *grubii* strains cause the majority of infections in immunocompromised patients (16), while C. gattii is strongly implicated in cryptococcosis in immunocompetent individuals (17). A few studies have demonstrated that there is also an influence of phylogenetic relatedness on disease within var. *grubii* strains. PCR/amplified fragment length polymorphism (AFLP)/multilocus sequence type (MLST) analyses divided var. *grubii* strains into three groups, namely, VNI, VNII, and VNB strains (18). Beale and colleagues (10) found that among strains from South Africa, survival was lower for eight patients infected with VNB strains than for those infected with the more common VNI or VNII strains (isolated from 175 and 47 patients, respectively). Similarly, Wiesner and colleagues (9) used MLST to type 111 strains isolated from Ugandan patients with their first episode of cryptococcal meningitis and conducted BURST clustering analysis to group strains with similar sequence types (STs) (all of which were in the VN1 clade). The members of BURST group 3 had significantly improved survival (62%) relative to those of BURST groups 1 and 2 (20% for both groups). Yet additional, finer-resolution studies performed by Mukaremera and colleagues within individual MLSTs showed that there was also substantial variation in rates of patient survival associated with individual strain differences (19). Interestingly, while the South African clinical strains exhibited diversity in STs, the Ugandan clinical strains were closely related, with ST93 strains accounting for approximately 60% of the isolates (9, 10, 19).

The conclusions that emerge from these studies are 2-fold. Strain background can significantly influence human disease, and there is tremendous disparity in strain frequency; some strain groups are much more common than others. ST93 is common in Uganda but is also the ST strain most frequently isolated from HIV-infected patients in Brazil (85% [20, 21]) and India (71% [22, 23]). Sequence type prevalence also has a clear geographic component, as different ST groups are dominant in other well-sampled countries (e.g., China, Thailand, Vietnam, Indonesia, Botswana, and France [22–24]).

Here we sought to identify candidate genes associated with clinical phenotypes in human subjects. We took advantage of the large number of patients in Uganda infected with closely related ST93 strains and combined this with a powerful data set collected during the Cryptococcal Optimal ART Timing (COAT) trial (ClinicalTrials registration no. NCT01075152) in Uganda (25). When participants enrolled in the trial, strains were isolated and participant survival and quantitative clinical and immunologic data were collected prior to treatment (26). We sequenced the whole genomes of 38 ST93 strains, half from participants that survived the infection and half from participants that died, reasoning that restricting our search to variants among closely related strains would reduce background genetic noise. We conducted a series of statistical tests that identified 40 candidate genes and 3 hypothetical RNAs associated with patient survival and clinical, immunologic, or *in vitro* phenotypes. We measured the virulence of 17 available KN99α knockout mutants for these genes in mice and found that 35% (6/17) had a significant association with mouse survival. Pathogen whole-genome sequencing paired with statistical analyses of human clinical outcome data and *in vivo* virulence tests thus provides a new method to empirically probe the relationship between pathogen genotype and human clinical phenotype.

## RESULTS

Fifty-six C. neoformans VNI strains isolated from HIV-infected, ART-naive patients presenting with their first episode of cryptococcal meningitis at Mulago Hospital, Kampala, Uganda, were subjected to whole-genome sequencing. The majority of strains (*n* = 38) were chosen from ST93 isolates (the dominant genotype in Uganda [9, 19, 25]), collected as part of the Cryptococcal Optimal ART Timing (COAT) trial, where an array of human immunologic phenotypes and disease parameters were recorded for all participants (26). Approximately half of these strains were derived from participants who survived the infection (*n* = 21) and half from participants who died (*n* = 17). The remaining 18 strains were chosen to represent the diversity of the clinical strains in Uganda for phylogenetic purposes.

We identified 127,344 single nucleotide polymorphisms (SNPs) and 15,032 insertions/deletions (referred to as indels) associated with 7,561 “genes” (this total includes predicted genes, hypothetical RNAs, and other genomic features that have associated CNAG designations on FungiDB) among the 56 sequenced C. neoformans strains. For ease of reference, we refer to these SNPs, insertions, and deletions cumulatively as “variants.” Over three-quarters of the identified variants were noncoding variants not predicted to change the amino acid sequence of a gene: synonymous changes within the gene (22%), intergenic regions (3%), or regions identified as upstream or downstream of the associated gene (within 5 kb of the nearest gene; 43% upstream, 10% downstream). The remaining (genic) variants are associated with 5,812 different genes. Nonsynonymous coding changes represent the largest class (90%) of these variants, with the remainder small insertion and deletion mutations.

**FIG 1:**
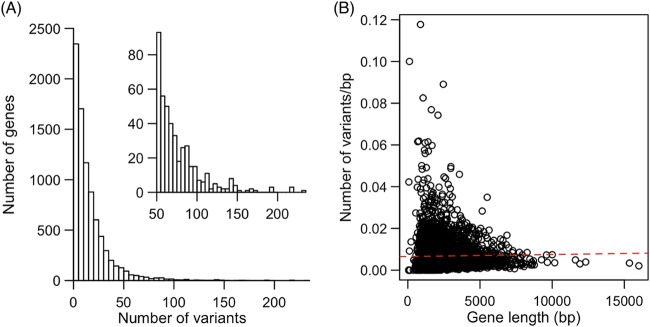
Summary of variants identified among all strains. (A) The number of variants per gene with a long right tail. The inset panel presents the same data magnified to show genes with at least 50 variants for visualization purposes. (B) There was no correlation between gene length and the number of variants per base pair in each gene (*P = *0.20).

**FIG 2:**
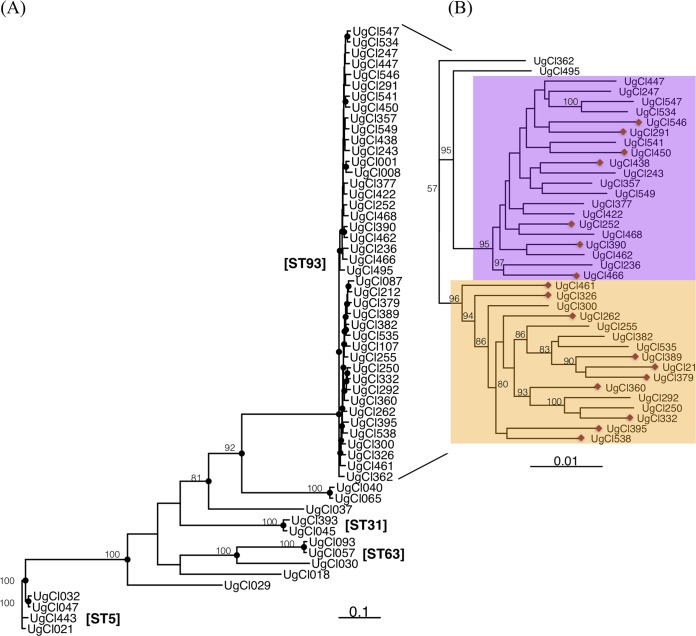
Phylogenetic analysis of all sequenced strains. (A) The majority of ST93 strains fall into two well-supported clades, magnified in panel B for ease of viewing as follows: ST93A (purple background) and ST93B (yellow background). Bootstrap values of >50 are indicated with the numeric bootstrap value. A red diamond at the end of the terminal branch indicates a strain isolated from a patient who died.

The majority of genes have relatively few variants within the strain set, though 435 genes have over 50 variants (Fig. 1A). There was not a significant relationship between gene length and the number of variants per base pair (Pearson's correlation test; *t*_4254_ = 1.29, *P = *0.20, correlation value [cor] = 0.02) (Fig. 1B), indicating that gene length is not the sole predictor of the number of variants in each gene. The numbers of variants in all sequenced genomes were extremely similar among strains of the same sequence type (* t* = 1.2868, df = 4254, *P = *0.1982), reflective of the phylogenetic distance from sequenced strains to the H99 reference genome (Fig. 2).

With this phylogenetic strain knowledge, we classified all variants into four categories: (i) “common” variants differentiating Ugandan clinical isolates from the reference H99 genome; (ii) “other” variants present only in non-ST93 genomes; (iii) “allST93” variants present in all ST93 genomes but in no other Ugandan ST genomes; (iv) “someST93” variants present in some of the ST93 genomes. For our study, we considered the most interesting variants to be the “allST93” or “someST93” variants because these categories would potentially identify variants that could explain the increased overall pathogenesis of ST93 in humans (category iii) and would allow us to identify variants within ST93 associated with human clinical outcomes and phenotypes (category iv).

### Common variants in ST93.

**FIG 3:**
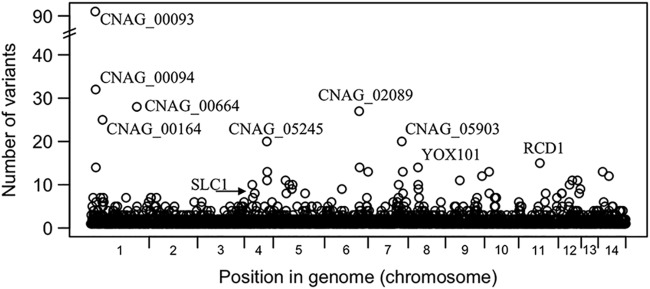
Variants that were common to all ST93 genomes are dispersed among 2,715 genes. A small number of clustered genes had a large number of variants. Genes with more than 20 variants and named genes are indicated.

Variants that are in all ST93 strains but not in the other sequenced strains (or the reference genome) can potentially tell us something about what differentiates strains in ST93 from other Ugandan strains. We identified 5,110 variants common to all 38 ST93 genomes (4,681 SNPs and 429 small indels). These variants were dispersed across the genome and associated with 2,575 genes and 140 hypothetical RNAs (Fig. 3; see also Table S1 in the supplemental material). The majority of these genes had one or a small number of variants, while a few genes had a very high number of variants (Table S1; 23 genes with at least 10 variants). The proportion of named genes in this set (8%, 2 of 24) matches that in the full gene set (8%, 686 of 8,338). The proportion of genes with a description (i.e., those not correlated to the “hypothetical protein” or “hypothetical RNA” classification) is actually lower in this gene set (33%) than in the whole gene set (49%; this difference was shown to be significant [*P < *0.0001] by the Fisher exact test).

10.1128/mBio.01440-19.4 TABLE S1 Genes, hypothetical RNAs, and intergenic regions with variants present in all ST93 genomes. Download Table S1, PDF file, 0.9 MB. Copyright © 2019 Gerstein et al. 2019 Gerstein et al. This content is distributed under the terms of the Creative Commons Attribution 4.0 International license.

### ST93 clade-specific variants.

**FIG 4:**
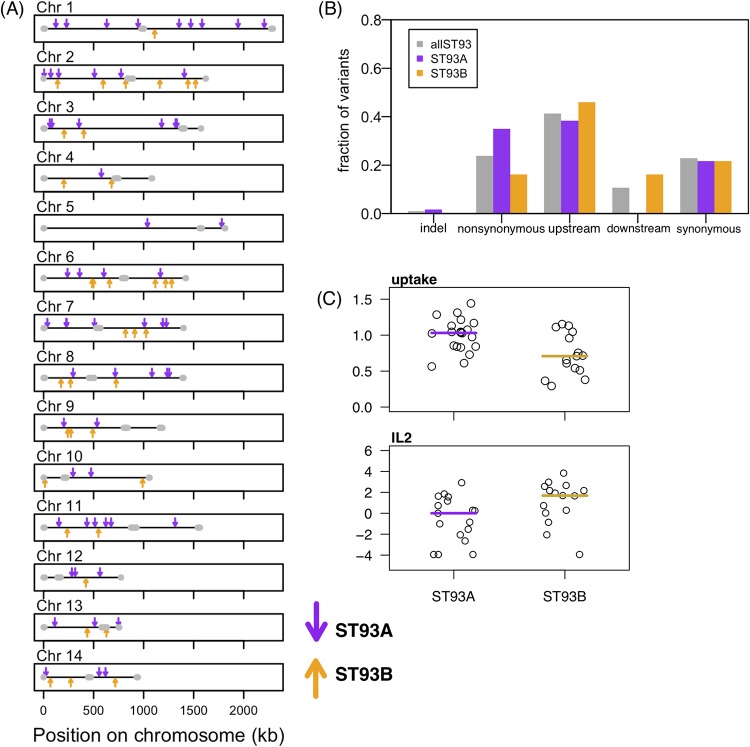
Clade-specific variants. (A) Variants that are specific to ST93A (purple) and ST93B (yellow) clades are distributed across the genome. (B) Upstream variants represent the majority class found in all ST93 genomes (“allST93”) and among the variants that are specific to either clade. In contrast, ST93A variants were more likely to be nonsynonymous and less likely to be downstream than the allST93 or ST93B variants. The distributions of the ST93A and ST93B variant classes are significantly different (*P = *0.009). (C) IL-2 cytokine levels in the CSF and *in vitro* phagocyte uptake levels differed between ST93A and ST93B strains (*t* test results: IL-2, *P = *0.022; uptake, *P = *0.011).

Examining the phylogenetic tree of the ST93 COAT strains, we surprisingly identified a well-supported split between the ST93 strains (Fig. 2B), with 20 of the sequenced strains in one group (“clade A”), 16 strains in a second group (“clade B”), and 2 ST93 strains outside the primary clades. Patient survival was approximately evenly split between the clades—7 patients that died had strains from clade A whereas 10 patients that died had strains from clade B (Fig. 2B) (Fisher’s exact test, *P = *0.18). We identified 97 variants that differentiate strains in one clade from the other; 60 variants were unique to and in all clade A strains, and 37 variants were unique to and in all clade B strains. Clade-specific variants were located throughout the genome (Fig. 4A) in 96 different genes, indicating that the differences between the two clades appear to involve the entire genome and not only a specific region. All except one of the genes contained only a single clade-associated variant—only CNAG_06422 in clade B contains two variants in the 5′ untranslated region (5′UTR) that are three bases apart. The distributions of variant classes differed between the two clades (chi-square test; χ^2^ = 13.44, df = 4, *P = *0.009); an increased number of nonsynonymous and decreased downstream SNPs were observed in clade A compared to clade B (Fig. 4B). Twenty-seven clade-specific mutations caused nonsynonymous amino acid changes (21 in clade A, 6 in clade B), and one small insertion mutation was present in clade A (Table S2). Although the majority of these variants are in genes that have not been characterized, four are in the following genes of known function: *LIV11* (CNAG_05422), encoding a virulence protein of unknown function; *HSX1* (CNAG_03772), encoding a high-affinity glucose transporter; *PTP2* (CNAG_05155), encoding a protein tyrosine phosphatase; and *SPT8* (CNAG_06597), encoding a predicted saga histone acetyltransferase complex component.

10.1128/mBio.01440-19.5 TABLE S2 ST93A and ST93B clade-specific variants. Download Table S2, PDF file, 0.1 MB. Copyright © 2019 Gerstein et al. 2019 Gerstein et al. This content is distributed under the terms of the Creative Commons Attribution 4.0 International license.

In addition to survival rates, we also determined whether variants in the ST93 strains were associated with clinical measures of disease, with cerebrospinal fluid (CSF) immune cytokine levels, or with *in vitro* phenotypes (25, 26) (Table 1) (see Materials and Methods for more details). We collectively refer to these three classes of phenotypes as “quantitative infection phenotypes.” We identified a significant association between the ST93 A/B clade and the *in vitro* macrophage uptake rate and patient CSF interleukin-2 (IL-2) level (Fig. 4C) (nonparametric Wilcoxon rank sum test; uptake *W* = 226, *P = *0.011; IL-2 *W* = 66.5, *P = *0.022). There was not a significant relationship between ST93 clade and the other quantitative infection phenotypes (see Fig. S1A in the supplemental material; nonsignificant *t* test results are listed in Table S3).

**TABLE 1:** Survival and quantitative infection phenotypes measured from participants enrolled in the COAT trial

Class	*n*	Phenotype variable
Survival	38	Patient survival

Clinical parameters	38	CD4 T cell
	35	CSF white cell
	31	CSF protein
	35	HIV load
	37	CSF clearance rate (EFA)
	30	CSF CrAg LFA titer

Immune cytokines	36	IL-1β
	36	IL-2
	36	IL-4
	36	IL-5
	36	IL-6
	36	IL-7
	36	IL-8
	36	IL-10
	36	IL-12
	36	IL-13
	36	IL-17
	36	G-CSF
	36	GM-CSF
	36	IFN-γ
	36	MCP-1
	36	TNF-α
	36	MIP-1β

*In vitro* characteristics	37	Absolute growth at 30°C
	37	Fluconazole MIC
	37	Amphotericin B MIC
	37	Cell wall chitin
	38	Macrophage adherence
	38	Macrophage uptake

10.1128/mBio.01440-19.1 FIG S1 Clade-specific differences in phenotype. Bar indicates median value. Download FIG S1, PDF file, 0.1 MB. Copyright © 2019 Gerstein et al. 2019 Gerstein et al. This content is distributed under the terms of the Creative Commons Attribution 4.0 International license.

10.1128/mBio.01440-19.6 TABLE S3 Statistical analysis of ST93 clade-specific associations with quantitative infection phenotypes. Download Table S3, PDF file, 0.1 MB. Copyright © 2019 Gerstein et al. 2019 Gerstein et al. This content is distributed under the terms of the Creative Commons Attribution 4.0 International license.

### Variant association with survival and quantitative infection phenotypes.

**FIG 5:**
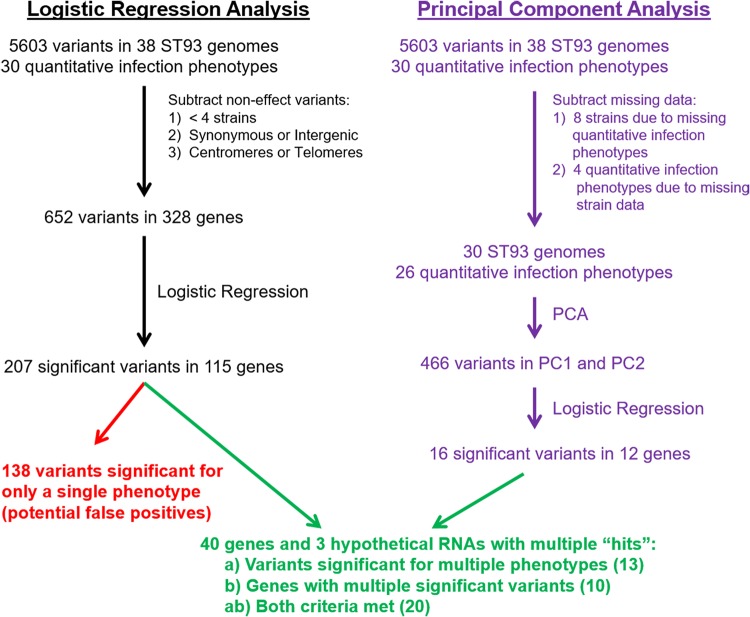
Flow chart for the bioinformatic approaches used to identify C. neoformans genes associated with survival and quantitative infection phenotypes. Survival was analyzed with logistic regression. Two complementary approaches were used for quantitative analysis of the infection phenotypes: (i) logistic regression followed by cluster analysis and (ii) principal component analysis (PCA). The clinical parameters, immune cytokines, and *in vitro* characteristics analyzed are listed in Table 1.

Our primary objective was to look for associations between the identified variants and patient survival rate or quantitative infection phenotypes. To do this, we parsed the 5,605 variants that were in some (but not all) of the ST93 genomes, with the goal of minimizing the number of statistical tests that we would have to perform to reduce the likelihood of false positives (Fig. 5). We removed variants that were in very few (<4) strains, with the rationale that for these variants we would have low power to detect a significant result and low confidence if we did. This removed 75% of the variants (the majority of variants, 47%, were in only a single genome). We also removed variants that mapped to either the centromeric or extreme telomeric regions. The centromeric region in C. neoformans is enriched for transposable elements (27), and the level of sequence misalignments that lead to false variant calls is high in these regions. Finally, we also removed variants without a predicted function, i.e., synonymous and intergenic variants; we acknowledge that these variants could have a fitness effect and that their removal might introduce bias. This left us with 652 variants.

To identify variants associated with patient survival, we conducted logistic regression tests independently for each variant against the number of days that a patient survived from the date of enrollment in the COAT trial. The test results for 12 variants from 7 genes were statistically significant (Table 2). Three of these genes are named: CNAG_06574 encodes APP1, a cytoplasmic protein involved in extracellular secretion and reduced phagocytosis (28); CNAG_05662 encodes ITR4, a protein involved in transport or sensing of 5-carbon and 6-carbon sugar alcohols (e.g., inositol, mannitol, sorbitol) (29–31); and CNAG_05663 encodes SCW1, a protein with homology to a cell wall integrity protein. The other four genes are listed as encoding “hypothetical proteins” on FungiDB.

**TABLE 2:** Significant variants from the linear regression analysis^*a*^

Gene^*b*^	Chr	Expression category^*e*^	Variant position(s)	Effect(s)^*c*^	*Class*^*d*^	Phenotypes
**05185**	4	D	**667433**; 667446	Up	*ab*	**Survival**, uptake; uptake
**02176**	6	D	988405; 988733; 988843; 988922; 989188; 989334; 989490; 989732; 990771; 990777; 990851; 990885; **991027**	Down; NS; NS; NS; NS; NS; NS; NS; NS; NS; NS; NS; Up	*ab*	Chitin, SERT; IL-1β, IL-13, MCP-1, MIP-1β; MIP-1β; LFA titer; IL-12; AMP; HIV RNA, SERT; IL-2; IL-10, MIP-1β; MIP-1β; IL-10, MIP-1β; SERT; IL-13, TNF-α, **survival**
**06574**	7	E	164473; 164887; 164926; 165027; **165704**; 165873; 166309; 167135; 167224; 167292; 167370	Up	*ab*	HIV RNA; IL-2, TNF-α; IL-2, MIP-1β; MIP-1β; **survival**, EFA; IL-13; growth; IL-13; GM-CSF; IL-1β, G-CSF, MIP-1β, uptake; CD4, uptake
**04373**	9	E	705343; **706175**	Up	*ab*	IL-8, EFA; **survival**
**07026**	12	D	**11092**; **11094**; 11400; 11406; 11407; 11410;11413	Up	*ab*	IL-1β, IL-13, **survival**, EFA; IL-13, **survival**, LFA titer; IL-1β, IL-7, IL-13, LFA titer; IL-1β, IL-7, IL-13, LFA titer; IL-1β, IL-7, IL-13, LFA titer; IL-1β, IL-7, IL-13, LFA titer; IL-1β
**05663**	14	D	910323; 910328; **910555**	Down	*ab*	TNF-α; IL-1β, IL-13, TNF-α; **survival**
**05662**	14	D	910742; **910822**; **910834**; 910926; 910939; **910964**; **910966**; **910979**; 911099; 911129; 911206; 911262; 911292; 911308; 911321; 911352	Down	*ab*	AMP; **survival**, FLC; **survival**; SERT; growth, SERT; **survival**, AMP; **survival**; **survival**, uptake; IL-12, GM-CSF, growth, TNF-α, MCP-1; IL-12, IL-13, IL-17, MIP-1β, TNF-α, growth, FLC, AMP, SERT; IL-8, MCP-1, MIP-1β; MCP-1; IL-2; adherence; IL-5; MCP-1
00014	1	E	47564; 47575; 47671	NS	*b*	G-CSF; G-CSF; GM-CSF
00363	1	E	927896; 927901	NS	*b*	IL-2; IL-2
07950	1	N	975152; 975212; 975397	Up	*ab*	IL-8, HIV RNA; IL-4, IL-6, IL-8, GM-CSF, IFN-γ, FLC; EFA
06704	2	D	270700	Up	*a*	IL-2, protein
02798	3	E	750294	Up	*a*	CD4, AMP
06876	5	N	7093	Down	*a*	IFN-γ, MIP-1β, TNF-α
01371	5	D	475470	Up	*a*	MCP-1, HIV RNA
01241	5	D	836479; 836697; 836899	Up	*ab*	IL-2; IL-4, IL-5, IL-7, IL-17, GM-CSF, TNF-α, chitin; IL-5, IL-12, IL-13, IL-17, G-CSF, TNF-α
02475	6	D	221273; 221275; 221282	Up	*ab*	IL-7, growth; growth; growth
02177	6	E	990701	Up	*a*	IL-1β, IL-6, IL-10
02112	6	E	1160524; 1160528; 1160532	Up	*b*	AMP; AMP; AMP
06525	7	D	11056; 14006	NS; Up	*ab*	IL-5, IL-10; IL-6, IL-8
12610*	7	D	49744	Up	*a*	MCP-1, uptake
05746	7	E	752861	UTR—3	*a*	IL-17, GM-CSF, MCP-1, TNF-α
05913	7	E	1205599; 1205600	Up	*ab*	MIP-1β, adherence; IL-13, IL-17, MIP-1β, adherence
05937	7	D	1263610	Up	*ab*	Uptake, SERT
07703	7	D	1341024	NS	*a*	IL-6, IL-8
06968	8	E	1383765	Indel	*a*	IL-12, IL-17
04100	9	N	5213; 7729; 8171	Up	*ab*	Adherence, FLC, SERT; growth; EFA, SERT
04102	9	D	10033	Down	*a*	GM-CSF, EFA
04179	9	D	220963	Up	*a*	EFA, SERT, protein
04535	9	E	1115286	Up	*a*	IL-17, G-CSF, LFA
07837	10	D	13558; 15288; 15302	Up; Down; Down	*b*	IL-2; WBCc; CD4
04922	10	D	18908; 18915; 18933; 18941; 18988; 18992; 18997	Up	*b*	IL-2; IL-2; IL-2; IL-2; adherence; adherence; adherence
08006	11	E	804710; 804742	Up	*ab*	IL-4, IL-5, IL-6, MIP-1β, TNF-α, adherence, chitin; IL-4, IFN-γ, MCP-1, adherence
01802	11	D	966644; 966669; 966700	Up	*b*	WBC; IL-2; IL-7
05987	12	D	14009; 14035; 14125;14197; 14202; 15014	NS; NS; Indel; NS; Indel; Up	*ab*	IL-2; IL-2; chitin; EFA, adherence; EFA, adherence; adherence
06169	12	E	502808; 502888; 502890; 503049; 503112; 503311; 503313; 503321; 503327; 503401	Down	*ab*	IL-8; GM-CSF, growth; IL-6, IL-8, GM-CSF; GM-CSF, HIV RNA; HIV RNA, WBC; G-CSF; IL-12, IL-13, G-CSF; IL-12, IL-13, G-CSF, MIP-1β; IL-12, IL-13, MIP-1β; IL-10, chitin
06256	13	N	11118; 11130	Up	*ab; b*	IFN-γ, TNF-α; TNF-α
13108*	13	N	128625; 128715; 128729	Up	*ab*	IL-13, G-CSF; IL-13, G-CSF; IL-13, G-CSF
06332	13	D	219021; 219311; 219312	Up	*b*	Adherence; EFA; EFA
06422	13	E	436551; 436554	Up	*b*	IL-2; IL-2
06490	13	D	655915	Indel	*a*	Protein, HIV RNA, CD4
05450	14	E	342562	NS	*a*	IL-6, IL-7, IL-12, IL-13, G-CSF, MIP-1β
05661	14	D	908850; 908994; 909011; 909638; 910152; 910181	Up	*ab*	IL-8, GM-CSF, IFN-γ, MCP-1, MIP-1β; uptake, FLC; IL-1β, IL-8, MIP-1β, uptake, FLC; adherence; uptake; IL-1β, IL-6, IFN-γ, HIV RNA
13204*	14	E	924025; 924047; 924049; 924050	Up	*b*	GM-CSF; IL-13; IL-13; IL-13

a The gray block denotes genes with variants associated with survival; gene numbers and variant positions that are associated with survival are indicated in bold. Dark gray text indicates genes, variants, and phenotypes that were identified as lower confidence in the *post hoc* bootstrap analysis. Semicolons are used as separators of different variants. When only one effect is listed, it is common among all variants of the gene. Chr, chromosome.

b Gene number corresponds to the CNAG number from the Cryptococcus neoformans H99 reference genome on FungiDB. Hypothetical RNAs are indicated with an asterisk (*).

c Effect data designate location or type of variant as follows: Up, upstream of the coding region; Down, downstream of the coding region; NS, nonsynonymous change in the coding region; Indel, small insertion or deletion.

d Class type designations are indicated as follows: a, the gene(s) has one variant significant for at least two phenotypes; b, there are multiple variants in the same gene with at least one significant phenotype each; ab, both criteria are fulfilled.

e E indicates expression; D indicates differential expression between the VNI and VNII clinical strains in the CSF; N indicates no expression detected. Data are from reference 32 and were analyzed in FungiDB as percentile of expression compared to all other genes in the experiment.

We took two complementary approaches to look for an association between the variants and the quantitative infection phenotypes. Our first tactic was to treat all measured phenotypes as independent. For our second tactic, we used principal-component analysis (PCA) to distill the 30 measured phenotypes into a smaller number of independent variables. Due to the nature of data collection for these types of phenotypic data, some strains were missing data for some phenotypes (Table S4). The most consequential example was that of two strains missing all cytokine data.

10.1128/mBio.01440-19.7 TABLE S4 Phenotypes measured from patients enrolled in the COAT trial (clinical and cytokines) and *in vitro*. Download Table S4, PDF file, 0.4 MB. Copyright © 2019 Gerstein et al. 2019 Gerstein et al. This content is distributed under the terms of the Creative Commons Attribution 4.0 International license.

For the first tactic, we analyzed phenotypes in each class as independent data sets in a logistic regression approach (Fig. 5), similarly to the approach used for patient survival. Due to missing data, the tactics taken to reduce the number of statistical tests left us with 466 variants in 230 genes for the cytokine data set (a subset of the 652 variants in 328 genes for the survival, clinical, and *in vitro* data sets) (Fig. 5). For each data set, we then conducted logistic regression analyses for each variant against each phenotype. Across all tests, 207 variants from 115 different genes were significant for at least one phenotype. The majority (138 variants) were significant for a single phenotype. To partially correct for false positives, we focused our further analyses only on the variants that were significant for at least two phenotypes (“*class a*”), on multiple significant variants that were identified in the same gene (“*class b*”), or on variants that fulfilled both criteria (“*class ab*”). This narrowed the list to 145 variants from 40 genes and 3 hypothetical RNAs, with 13 variants in *class a*, 36 variants in *class b*, and 96 variants in *class ab* (Table 2) (full information about significant variants, including class, is provided in Table S5 and full statistical information about each significant variant and phenotype in Table S6).

10.1128/mBio.01440-19.8 TABLE S5 Significant variants in genes and hypothetical RNAs with quantitative infection phenotypes based on class designation. Download Table S5, PDF file, 0.1 MB. Copyright © 2019 Gerstein et al. 2019 Gerstein et al. This content is distributed under the terms of the Creative Commons Attribution 4.0 International license.

10.1128/mBio.01440-19.9 TABLE S6 Logistic regression analysis of all significant variants in genes and hypothetical RNAs associated with quantitative infection phenotypes. Download Table S6, PDF file, 0.1 MB. Copyright © 2019 Gerstein et al. 2019 Gerstein et al. This content is distributed under the terms of the Creative Commons Attribution 4.0 International license.

Following the use of the default parameters described for the SnpEff program, we used a very broad definition for calling variants upstream or downstream variants (±5 kb). Over 80% of the significant variants were located either upstream or downstream of genes (86 variants upstream, 34 variants downstream), with 20% within 1 kb (Table S6). Of the remaining variants, 21 were nonsynonymous, while 4 were indels. The majority of significant genes contained multiple significant variants (Table 2). In some cases, different variants in the same gene influenced the same phenotype, generally because the multiple significant variants were linked (e.g., three nonsynonymous variants in CNAG_00014, with the majority of ST93 strains falling into two haplotypes; one upstream SNP and two upstream insertions in CNAG_02112, with two haplotypes that influenced amphotericin B resistance). In other cases, such as that of CNAG_07950, there were six different haplotypes and three significant upstream variants that were associated with 8 unique phenotypes (e.g., IL-8 was associated with two variants, while HIV RNA, IL-4, IL-6, granulocyte-macrophage colony-stimulating factor [GM-CSF], gamma interferon [IFN-γ], fluconazole MIC, and early fungicidal activity [EFA] were each associated with a single variant).

It was unavoidable that, even after we minimized the number of tests and implemented the variant class criteria described above, some of the identified variant × trait associations would represent false positives. To determine the genes that we had most confidence in, we conducted a *post hoc* bootstrap procedure on all identified *class a*, *b*, and *ab* variants. For each variant × significant trait association, the data were randomized 500 times (i.e., the measured phenotype was randomly assigned to one of the observed genotypes) and the logistic regression model was rerun to compare the observed estimate to the bootstrap replicate estimates. For 74 cases (24%), there were at least 25 (i.e., >5%) bootstrap replicates with estimates more extreme than the observed estimate (Table S7). These 74 cases predominantly involved a subset of traits, namely, the traits measured *in vitro* (macrophage adherence and uptake, cell wall chitin, antifungal drug resistance, and absolute growth), the levels of the cytokines granulocyte colony-stimulating factor (G-CSF) and GM-CSF, and LFA titer. However, the results of this cross-validation analysis emphatically did not influence our overall screen conclusions. Only five genes (CNAG_00014, CNAG_02112, CNAG_05185, CNAG 05937, and CNAG_12610) no longer met the criteria identified above.

10.1128/mBio.01440-19.10 TABLE S7 The majority of genes associated with quantitative infection phenotypes are uncharacterized. Download Table S7, PDF file, 0.1 MB. Copyright © 2019 Gerstein et al. 2019 Gerstein et al. This content is distributed under the terms of the Creative Commons Attribution 4.0 International license.

We also conducted PCA as a second tactic to reduce the potential influence of phenotypic correlation on the results (Fig. 5). As PCA requires complete data sets, we used data from the 27 phenotypes that had missing data from only three or fewer strains. That is, we excluded cryptococcal antigen (CrAg) lateral flow assay (LFA) titer, HIV RNA viral load, CSF protein, and CSF white blood cell (WBC) data (Table S4) and had to exclude 8 strains (Ugandan clinical strain 212 [UgCl212], UgCl332, UgCl357, UgCl422, UgCl447, UgCl461, UgCl541, and UgCl549) (Table 1). The “prcomp” function from R programming language was used to perform PCA on the two phenotypes which were scaled to have unit variance and shifted to be zero centered. We continued with the first two principal components by comparing the observed results to 20 data sets where the phenotypic data were randomized among strains (Fig. S2A). Logistic regression analysis was run for each of the 466 variants that passed filtration against PC1 and PC2. The PCA yielded only 16 significant variants in 12 genes (Table 3). Only one of these genes, CNAG_07727, was not identified in the first analysis, and 12 of these variants were previously found to be statistically significant. Thus, implementation of our two analysis tactics—the linear regression analysis and the PCA—yielded an overlapping set of variants and similar outcomes.

**TABLE 3:** Significant variants from PCA

Gene	Chr	Position	Effect	PCA1 *P* value	PCA2 *P* value
CNAG_07950	1	975212	Upstream	0.047	0.141
CNAG_01241	5	836697	Upstream	0.04	0.505
	5	836899	Upstream	0.025	0.29
CNAG_02176	6	988733	Stop gained	0.047	0.749
	6	989490	NS	0.834	0.03
	6	989960	NS	0.967	0.039
CNAG_07703	7	1341024	NS	0.031	0.289
CNAG_07727	8	818838	Upstream	0.036	0.726
CNAG_08006	11	804710	5′UTR	0.048	0.312
CNAG_05987	12	19741	Upstream	0.355	0.031
CNAG_06169	12	503321	3′UTR	0.048	0.795
CNAG_05450	14	342562	NS	0.024	0.142
CNAG_05661	14	908850	Upstream	0.042	0.928
CNAG_05663	14	910328	Downstream	0.042	0.12
CNAG_05662	14	911099	Downstream	0.045	0.143
	14	911129	Downstream	0.048	0.046

10.1128/mBio.01440-19.2 FIG S2 PCA. (A) Each dashed line represents 1 of 20 randomized trials. (B) There was no association between PC1 or PC2 and clade. Download FIG S2, PDF file, 0.1 MB. Copyright © 2019 Gerstein et al. 2019 Gerstein et al. This content is distributed under the terms of the Creative Commons Attribution 4.0 International license.

**FIG 6:**
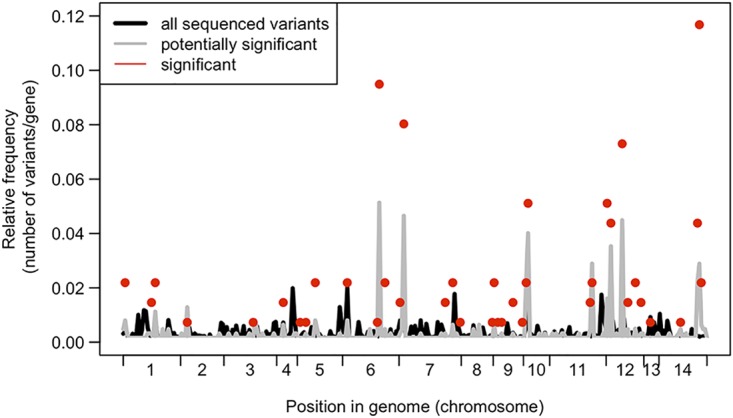
Comparing variant frequencies across the genome. Data represent relative frequencies of variants per gene for significant genes (red dots) compared to all sequenced variants across all genomes (black line) and all variants that were variable within ST93 genomes (gray line). The only genes shown here are those with at least one potential significant variant; hence, the gray and black lines do not reach 0.

The majority of genes with a high number of significant variants were also genes with high numbers of sequenced variants and potentially significant variants (Fig. 6). In addition to variation among genes in regard to the number of significant variants within a gene (“sig variants,” ranging in number from 1 to 34), there were also variations in the number of variants that were identified within a strain (“sequenced variants”; range, 1 to 210) and in the number of variants that passed our filters (“potentially significant variants”; range, 1 to 32). These results highlight a limitation of genetic association screens such as the one that we performed. Without additional biological validation, it is difficult, if not impossible, to ascertain whether a given gene has many significant variants because of strong selection acting on that gene (e.g., if a knockout phenotype is beneficial, there are many different positions that can reduce gene expression or protein levels) or because of relaxed selection and chance (i.e., if there is relaxed selection, then many variants could be present, with statistical significance arising by chance). However, the fact that we do see areas of discordance between all the sequenced variants, potentially significant variants, and significant variants suggests that many of our significant variants do not represent just a statistical artifact.

### *In vivo* virulence of identified genes.

Our goal was to identify pathogen variants in genes that impact human clinical disease phenotypes. We reasoned that, for the gene variants to have a high probability of influencing human clinical disease, they should be expressed *in vivo*. Expression data are not available as part of the COAT data set, so how the specific variants influence gene expression in humans is unknown. However, data representing levels of *in vivo* gene expression in cerebrospinal fluid (CSF) are available from two human patients infected with two different, genetically distinct strains (32). We analyzed these data for *in vivo* expression of the 40 genes and 3 hypothetical RNAs (Table 2). Thirty-seven (37/40) of the genes and two (2/3) of the RNAs were expressed in at least one of the strains. Interestingly, we noted differential expression of 56% of the genes between the two strains, but because the strains were not fully sequenced, we were unable to determine what variants they contain.

**FIG 7:**
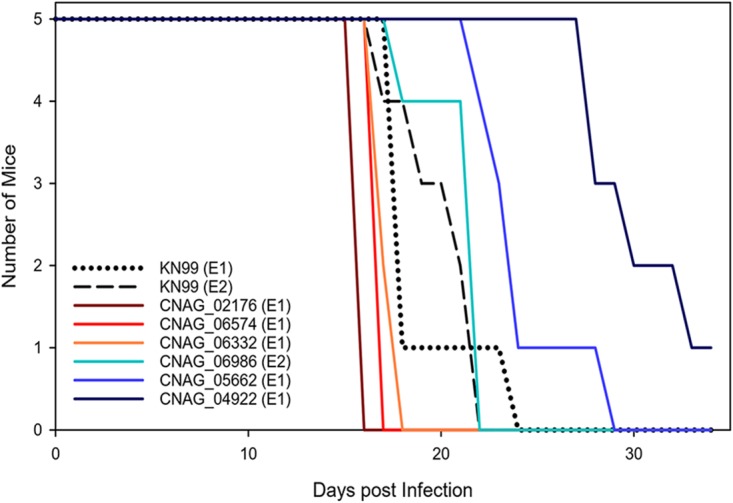
Deletion strain virulence in mice. Groups of five 6-to-8-week-old C57BL/6 mice were infected intranasally with 5 × 10^4^ cells. Progression to severe morbidity was monitored for 35 days, and mice were sacrificed when endpoint criteria were reached. Strains were tested in two separate experiments (indicated as experiment 1 [E1] and E2, respectively). Statistical analysis of the survival curves are presented in Table 4.

Mukaremera and colleagues recently showed that the mouse inhalation model of cryptococcosis accurately recapitulates human infections and can be used to dissect C. neoformans genetic factors that influence human disease (19). Thus, as a first step to probe the biological significance of the genes identified in our analyses, we tested the virulence of 17 available KN99α deletion strains in the mouse inhalation model. Six (35%) of the tested deletion strains had a significant effect on mouse survival compared to the control KN99α strain; three strains (CNAG_02176, CNAG_06574, and CNAG_06332) had increased virulence, and three strains (CNAG_06986, CNAG_04922, and CNAG_05662) had decreased virulence (statistical data are listed in Table 4, strains with differences that were found to be statistically significant are shown in Fig. 7, and strains with differences that were found not to be statistically significant are shown in Fig. S3A). Although the use of gene deletion mutants represents only one way to biologically probe whether a candidate gene has a true virulence phenotype, we did find that the number of significant variants in a gene (Table 2) was a significant predictor of the deletion mutations having a virulence effect (linear model, *F*_1,15_ = 8.493, *P = *0.011).

**TABLE 4:** Survival curve statistical results

Gene knockout	χ^2^ statistic (df = 1)	*P* value
CNAG_00363 (*tco6Δ*)	0.05	0.82
CNAG_02176	9	0.0027
CNAG_04373	3.07	0.08
CNAG_04535	2.79	0.095
CNAG_04922	9.97	0.0016
CNAG_05662 (*itr4Δ*)	6.22	0.013
CNAG_05663	0.61	0.43
CNAG_05913	0.07	0.79
CNAG_05937	0.09	0.77
CNAG_06169	0.13	0.72
CNAG_06332	4.05	0.044
CNAG_06490	1.02	0.31
CNAG_06574 (*app1Δ*)	9	0.0027
CNAG_06704	5.83	0.016
CNAG_06876	0.05	0.82
CNAG_06986	7	0.0082
CNAG_07703	0.05	0.31
CNAG_07837	1.8	0.18

10.1128/mBio.01440-19.3 FIG S3 Deletion strain virulence in mice. (A) Groups of five 6-to-8-week-old C57BL/6 mice were infected intranasally with 5 × 10^4^ cells. Progression to severe morbidity was monitored for 35 days, and mice were sacrificed when endpoint criteria were reached. Strains were tested in two separate experiments (experiment 1 [E1] and E2). The deletion strains were compared to the KN99α strain in the same experiment. (B) Groups of four 6-to-8-week-old C57BL/6 mice were infected intranasally with 1 × 10^3^ cells. Mice were sacrificed at 7 days postinfection, lungs were homogenized in 4 ml of PBS, and serial dilutions were plated on YPD with chloramphenicol medium. CFUs were enumerated at 48 h. Download FIG S3, PDF file, 0.1 MB. Copyright © 2019 Gerstein et al. 2019 Gerstein et al. This content is distributed under the terms of the Creative Commons Attribution 4.0 International license.

### *In vivo* and *in vitro* analysis of *itr4*Δ and clinical strains.

**FIG 8:**
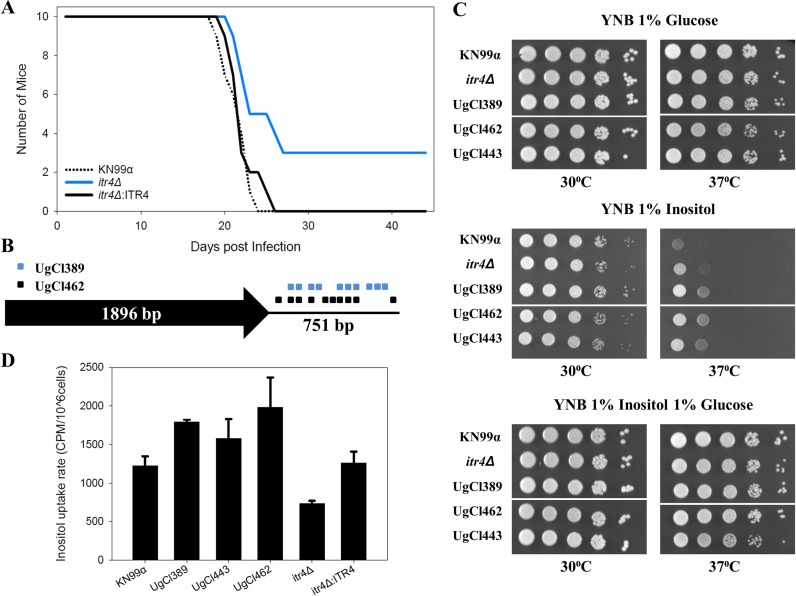
Analysis of *ITR4* through *in vivo* virulence and *in vitro* growth and inositol uptake. (A) Groups of 10 6-to-8-week-old C57BL/6 mice were infected intranasally with 1 × 10^3^ cells. Progression to severe morbidity was monitored for 44 days, and mice were sacrificed when endpoint criteria were reached. (B) Schematic diagram showing locations of the variants in the UgCl389 and UgCl462 clinical isolates relative to the ITR4 coding region. UgCl443 has the H99 reference allele. (C) Growth assay of C. neoformans wild-type strain *KN99*α, *itr4Δ* mutant, and clinical strains on medium with different inositol levels. Yeast cells were cultured in YPD medium. Equal cell concentrations were spotted as 10-fold serial dilutions onto YNB plates made with 1% glucose, 1% inositol, or 1% glucose and 1% inositol. Plates were incubated at 30°C or 37°C, and growth was examined after 4 days. The assay was repeated three times with similar results. (D) Inositol uptake analysis of C. neoformans strains. Yeast cells were mixed with 3H-labeled inositol and incubated at 30°C for 10 min in triplicate (repeated twice with similar patterns). Error bars indicate standard deviations of results from the three replicates. All strains presented were grown on the same plate, but some strains that were present on the plate have been removed for clarity. Each white line indicates a location where a strain was removed.

The gene with the highest number of significant variants in our candidate gene list was CNAG_05662 (*ITR4*), which has been reported to be a member of the inositol transporter gene family (30, 31). The *itr4Δ* mutant strain had reduced virulence in the mouse model whereas the *itr4Δ*:ITR4 complement strain had virulence equivalent to that of laboratory reference background strain KN99α showing that the ITR4 deletion is responsible for the virulence defect in the *itr4*Δ mutant (Fig. 8A) (mutant strain *itr4Δ* chi-square statistic for test of equality = 6.22, *P = *0.013; complement strain *itr4Δ*:ITR4 chi-square statistic = 0.51, *P = *0.47). In this lower-inoculum experiment, where the infection was less likely to overwhelm the initial immune response, three of the mutant strain *itr4Δ*-infected mice survived until the experiment was ended on day 44 (Fig. 8A). Terminal CFU from the brain and lungs of the survivors showed complete fungal clearance in one mouse and a low (2 × 10^2^ CFU) fungal burden in the lungs in the second mouse. The third mouse had 5.64 × 10^5^ CFU in the lungs and 1.35 × 10^4^ CFU in the brain. Evaluation of the fungal burden at 7 days postinfection showed higher levels of *itr4*Δ mutant CFU in the lungs than of reference strain KN99α CFU and complement strain *itr4Δ*:ITR4 CFU and no mutant strain *itr4*Δ CFU in the brain (Fig. S3B), suggesting that the reduced pathogenesis observed in the *itr4Δ* mutant was likely due to reduced growth in or delayed dissemination to the brain.

To further determine the role of the genetic variants in the biological function of *ITR4*, reference strain KN99α, mutant strain *itr4Δ*, and three clinical strains (UgCl389, UgCl462, and UgCl443) were tested for growth with inositol and inositol uptake. The variants associated with the *ITR4* locus in these clinical strains are proximal to the coding region—both UgCl389 and UgCl462 have 11 single nucleotide polymorphisms (SNPs) immediately downstream of the coding region whereas UgCl443 contains the H99 reference allele for *ITR4* (Fig. 8B). All the clinical strains showed enhanced growth with inositol only at 37°C compared to reference strain KN99α, and their levels of growth were similar to that seen with the *itr4*Δ mutant (Fig. 8C). UgCl389 and UgCl462 were also more efficient at inositol uptake, while the efficiency of uptake by UgCl443 was similar to that seen with reference strain KN99α but the mutant strain *itr4*Δ had decreased inositol uptake (Fig. 8D). Taken together, these data highlight the complex nature of the multiple variants across the clinical strains. Due to differences between the clinical strains with respect to their genetic backgrounds, interpretation of the impact of specific variants and/or gene alleles is challenging.

## DISCUSSION

Virulence is a multifaceted phenotype, as many different pathogen and host characteristics determine the severity of a given infection. Here we paired a powerful data set from the Cryptococcal Optimal ART Timing (COAT) trial in Uganda (26) with pathogen whole-genome sequencing technology to identify the candidate C. neoformans genes that were statistically associated with both survival and quantitative human infection phenotypes. The technique of using genome-wide association studies (GWAS) to uncover genic variants linked to disease was developed 14 years ago in the context of human disease genetics (33). Here we looked for associations between variants within 38 ST93 C. neoformans isolates from participants enrolled in the COAT trial both for patient survival and for an additional 29 associated clinical, immunologic, and *in vitro* phenotypes. We employed two complementary tactics to identify candidate genes. The first treated each measured phenotype as independent and yet included only genes with a variant significantly associated with multiple phenotypes (13 genes) or genes with multiple significant variants (10 genes) or both (20 genes). The use of this “*class*” approach to identify variants in the logistic regression analysis probably reduced the number of false positives in our analysis but likely also introduced bias into the analysis through exclusion of single variants associated with one phenotype. We also conducted a PCA to examine the first two principal components from a PCA of the 27 phenotypes and 30 strains with sufficient data. The resultant reduction of power was unfortunate but not surprising in dealing with human data. The detrimental impacts of missing clinical data have been previously discussed (34) and indeed represent the reason that we employed both tactics. The PCA yielded a total of 12 genes, including 11 genes that overlapped those identified in the first analysis and 1 additional gene. The observation that the logistic regression analysis performed using our class approach and the PCA yielded quite similar outcomes provides additional confidence that significant bias was not introduced by the *class* approach. Combining the data, we identified 40 candidate C. neoformans genes and three hypothetical RNAs associated with infection phenotypes among the ST93 strains.

The statistical analysis was blind with respect to any prior knowledge of the genes and thus did not depend on prior annotation. Accordingly, the majority of genes that we identified have not yet been named, and the proteins encoded by roughly half (*n* = 19) of those genes are listed as “hypothetical proteins” on FungiDB. Interestingly, only 2 of these 19 genes are conserved among fungal taxa, and curating information about orthologues from FungiDB (https://fungidb.org/fungidb/) suggests that the majority of others either are unique to C. neoformans or have orthologues only in the very closely related species complex C. gattii (see Table S7 in the supplemental material). This is consistent with the logic of Liu et al. (7), who purposely targeted genes that did not have homologues in Saccharomyces cerevisiae during the construction of the original H99 gene deletion collection (an 1,180 gene collection in C. neoformans H99, which corresponds to ∼20% of the protein-coding genes) (14).

We took advantage of the newer KN99α gene deletion collection (35) and found that 35% (6/17) of the available gene knockouts had an effect on virulence in mice. The significant genes with a virulence change in mice include two named genes, *ITR4* (CNAG_05662) and *APP1* (CNAG_06574), and one hypothetical protein-encoding gene (CNAG_02176), as well as genes encoding two additional hypothetical proteins that have orthologues only in closely related species (CNAG_04922 and CNAG_06332) and one hypothetical protein with broad taxonomic distribution (CNAG_06968). The *app1*Δ mutant has previously been shown to have decreased virulence in mice (28). Interestingly, this contradicts date from our mouse model, which showed increased virulence of the *app1*Δ mutant. This difference could be due to the differential immune responses in BALB/c mice (previous study, type 1 immune response) and C57BL/6 mice (current study, type 2 immune response) and likely gives a hint with respect to the mechanism of *APP1* in human disease.

Intriguingly, *ITR4* (synonym *PTP1*) was the top hit in a screen that identified genes that were overexpressed in an intracellular environment (amoebae and murine macrophages) compared to the laboratory medium (yeast extract-peptone-dextrose [YPD]) (29). In that study, the *itr4*Δ mutant did not differ from the wild-type strain in mouse assays or Galleria mellonella virulence assays (29), though those previous studies were performed in a genetic background different from the background of our KN99α reference strain and in BALB/c mice. Using gene complementation, we clearly show the virulence defect in the *itr4*Δ mutant is due to deletion of the *ITR4* gene. And yet the phenotypic data showing enhanced growth at 37°C on inositol but reduced inositol uptake of the *itr4Δ* mutant, combined with enhanced growth and uptake by the clinical strains, are not straightforward and not conclusive with respect to gene function. All of the clinical strains appeared to be better adapted for growth and uptake of inositol than the KN99α reference strain. This is not surprising, given that the clinical strains were isolated from the central nervous system, which is an inositol-rich environment. Because most of the *ITR4* gene variants are proximal to the coding region, these alterations may alter expression of the *ITR4* gene, or transcript/protein stability *in vivo*, rather than abolish gene expression such as occurs in the *itr4Δ* mutant. This could explain the differences between the *in vitro* inositol phenotypes that we observed in our clinical isolates and those shown by the mutant. It is also possible that the genetic background of the clinical isolates influences the function of the different *ITR4* gene variants, as these genes are known to be part of larger inositol acquisition and utilization pathways. Additional interactions between variants and pathways may also exist. Combinations of variants in different genes within one isolate might also be important. If so, standard genetic replacement and allele swap experiments may disrupt these gene combinations. Instead, quantitative trait locus (QTL) or linkage disequilibrium strategies may be necessary to define networks of variants that interact. Larger clinical populations will be needed for these types of analyses.

There was no clear relationship between the genes that were identified in both of our statistical analyses and the gene deletion virulence in mice (five genes were significant in both, including two with a significant gene deletion virulence effect; Table S7). We note, however, that although data have indicated a good link between strain survival in mice and human virulence (19), there are two major limitations with respect to interpretation and extrapolation of the virulence tests that we performed in this study. The first is that the phenotype of a gene knockout does not necessarily recapitulate the effect of a natural point or indel mutation (36–38). Importantly, variants located upstream of a gene were extremely prevalent in our data set, suggesting that they would not be phenocopied with a gene deletion if an increase in expression is required to influence the trait. Expression data are not available as part of the COAT data set, so how the specific variants influence gene expression in humans is as yet unknown. However, our analysis of the *in vivo* CSF expression data reported previously by Chen et al. (32) does suggest that expression differences in these genes can exist between strains.

The second reason for caution in interpreting the data is that the gene knockout collection is in the KN99α genetic background. It has previously been shown that although ST93 and KN99α are both VNI strains, they are phylogenetically quite distantly related (9). We see this distance in our own data set: 2,941 variants were present in the closely related ST93 genomes that we sequenced and over 40,000 variants were present across all the genomes compared to the H99 reference strain. Genetic background is known to play a significant role in the effect of a mutation. A large study in Saccharomyces cerevisiae recently found that 16% to 42% of deletion phenotypes changed between pairs of strains, depending on the environment (39). To fully probe the influence of the variants and genes that we identified in our screen, these variants need to be studied in the ST93 background. Given these limitations, we anticipate that additional studies will uncover more genes with an impact on pathogenesis from our study. It would also of course be of general interest to reconstruct a knockout collection in a strain background more representative of typical clinical strains (14, 23).

We purposefully chose to focus our study on strains from ST93, which was the most prevalent ST group among the strains that we sampled from participants in the COAT trial (∼63% of all strains). In the COAT trial, ST93 did not significantly influence mortality (among the patients infected by group ST93 strains, 22 died and 24 survived; among the patients infected by non-group ST93 strains, 9 died and 16 survived [Fisher’s exact test *P = *0. 45]). ST93 was similarly the most prevalent among patients with advanced HIV infections in Brazil (20). In contrast, ST93 isolates were less common than ST5 isolates among immunocompetent patients in Vietnam, and non-ST5 strains were associated with decreased mortality compared to ST5 strains (40). Other studies have found no ST93 isolates (41, 42). This picture of geography having a major impact on which group is most prevalent raises the issue of whether it is merely chance or the effect of selection that sorts lineages geographically. How this geographic distribution of genotypes affects underlying variants is unknown. It is probable that the genes identified in this study, using ST93 as a model, will also be found to be important in other genetic backgrounds. It is less clear whether specific variants, especially those outside the protein-coding region, will be retained across genetic lineages and can be used as markers to define human disease risk.

As additional “genome-enabled” clinical data sets are constructed, we can hope to gain a clearer global picture of the link between broad and narrow ranges of genomic variability and clinical outcome. Our narrow analysis in the ST93 strains was possible because of the large number of patients infected with this sequence type in Uganda. Only when similar studies are performed in patient populations throughout the world, with other dominant STs, or in the context of increased genetic diversity, will we be able to determine how broadly applicable our study is to the global population of C. neoformans.

Statistical association techniques using human clinical data, such as those employed here, offer a complementary approach to genetic screens of mutant collections. They offer the benefits of not having to choose a particular strain background (typically the reference strain) and of not having to make decisions about which genes are likely to be important. For example, the method of selection of genes for the initial C. neoformans knockout collection was biased against genes with homologs in S. cerevisiae and against C. neoformans*-*specific genes (7). There are also inherent biases in forward genetics methods. Here we had only the statistical power to find association with common variants. The majority of variants that we sampled were singleton variants in only a single genome (Fig. 1A), and some of these may well have an extremely important influence on virulence that remained undetected in our current analysis. Hence, we have treated our pathogen GWAS analysis like a genetic screen; the power lies in the opportunity to compare studies of different types to find candidate genes or alleles to focus our attention on.

Our analysis did not identify variants in many of the genes that were previously identified through *in vitro* and in-animal mutant screens as virulence factors in C. neoformans, such as genes involved in capsule formation and melanin synthesis. There could be several reasons for this result. Importantly, all of the ST93 strains analyzed were isolated from patients with cryptococcal meningitis; thus, all these strains by definition are capable of causing disease and in our study the readout was not presence or absence of disease but rather the severity of disease. Previous studies may have identified virulence factors involved in the early stages of infection that impact the ability of C. neoformans to infect and then survive within the host, whereas our study identified virulence factors that promote or inhibit the progression of disease. Also, our analysis utilized human clinical data for association with genetic differences between strains whereas previous studies utilized surrogates (either *in vitro* conditions or animal models). By studying genetic differences in the context of human infection, we have not only the potential to define genes that promote disease in humans but also the potential to define aspects of the host-pathogen interaction that are specific to C. neoformans and the human host.

## MATERIALS AND METHODS

### Ethics statement.

Animal experiments were done in accordance with the Animal Welfare Act, United States federal law, and NIH guidelines. Mice were handled in accordance with guidelines defined by the University of Minnesota Animal Care and Use Committee (IACUC) under protocol 1607-34001A. Participant data were collected as part of the COAT trial (ClinicalTrials registration no. NCT01075152) (26, 43). All participants were enrolled at Mulago Hospital, Makerere University, Kampala, Uganda. Written informed consent was obtained from all subjects or a proxy, and all data were deidentified. Institutional Review Board (IRB) approvals were obtained at both the University of Minnesota (0810M49622) and Makerere University.

### Strain selection.

We utilized C. neoformans isolates collected in Uganda as part of the Cryptococcal Optimal ART Timing (COAT) trial (26). We focused primarily on 38 UgCl COAT strains that had previously been MLST genotyped as sequence type 93 (ST93), representing the most prevalent ST group in this collection of strains (25). An additional 18 strains from 10 MLST groups were also subjected to whole-genome sequencing to represent the strain diversity in Ugandan clinical isolates (9).

Clinical isolates were subjected to colony purification from the CSF of participants that presented at the clinic with their first episode of cryptococcal meningitis. The ST93 clinical isolate strains were purposefully chosen to represent strains from both participants who survived (*n* = 21) and those who died (*n* = 17). Patient infection phenotypes (i.e., clinical and cytokine parameters; Table 1) were measured on the day that patients were diagnosed with cryptococcal meningitis, prior to antifungal or ART treatment. Cytokine data were log_2_ transformed prior to analysis, as described previously (44).

### Library preparation and Illumina sequencing.

DNA was extracted using the cetyltrimethylammonium bromide (CTAB) DNA isolation method (45). Colony-purified cultures, maintained as glycerol stocks at −80°C, were inoculated into 250 ml of yeast extract-peptone-dextrose (YPD) agar in Erlenmeyer flasks and grown overnight at 30°C with continuous shaking prior to DNA isolation.

Strains were subjected to whole-genome sequencing in two sets. In the first set of strains, genomic DNA libraries from 16 strains were prepared by the Mayo Bioinformatics Core for 101-bp paired-end sequencing. The samples were combined into two pools (pool A, UgCl001, UgCl018, UgCl021, UgCl029, UgCl030, UgCl037, UgCl040, UgCl045, UgCl057, and UgCl107; pool B, UgCl008, UgCl032, UgCl047, UgCl065, UgCl087, and UgCl093). Each pool was sequenced on a single lane of an Illumina HiSeq 2000 instrument.

In the second set of strains, genomic DNA libraries from the 40 strains were prepared by the University of Minnesota Genomics Center for 300-bp paired-end sequencing with an Illumina TruSeq DNA LT kit. The samples were combined into four pools; each pool was sequenced in a single lane of an Illumina HiSeq instrument (pool 1, UgCl212, UgCl236, UgCl243, UgCl247, UgCl250, UgCl389, UgCl541, UgCl547, and UgCl549; pool 2, UgCl252, UgCl255, UgCl262, UgCl291, UgCl292, UgCl300, UgCl326, UgCl332, UgCl357, and UgCl360; pool 3, UgCl362, UgCl377, UgCl379, UgCl382, UgCl390, UgCl393, UgCl395, UgCl422, UgCl438, and UgCl443; pool 4, UgCl447, UgCl450, UgCl461, UgCl462, UgCl466, UgCl468, UgCl495, UgCl534, UgCl535, UgCl538, and UgCl546). In the second set of sequencing runs, the runs generated more than approximately 22 million pass filter reads for pools 1 and 2 and more than approximately 17 million pass filter reads for pools 3 and 4. In all runs, >70% of the bases represent a quality value (Q) above Q30. The average library insertion size ranged from 400 to 500 bp. Genome sequences are available at NCBI under BioProject ID PRJNA549026.

### Variant calling.

Variant calling for each strain was adapted from the best practices described for the Genome Analysis Toolkit (GATK v3.3.0) (46–48). For each strain, the two paired-end fastq files were trimmed using trimmomatic (49) and aligned to the C. neoformans H99 reference genome (downloaded from FungiDB [http://fungidb.org/fungidb/] on 1 February 2016; “FungiDB-26_Cneoformans_H99_Genome.fasta”) with bwa mem (50). The output (.SAM) files from all other strains were converted to .BAM files and sorted, duplicates were marked and indexed, and a final index was built with Picard tools (http://broadinstitute.github.io/picard). Variants were called for each sample with GATK HaplotypeCaller run in VCF mode for each strain (with flags –genotyping_mode DISCOVERY –emitRefConfidence GVCF -variant_index_type LINEAR -variant_index_parameter 128000 -ploidy 1) to obtain gVCF files. GATK GenotypeGVCFs was then run to merge the 41 gVCF records. Variants were annotated with SnpEff (51) followed by GATK VariantAnnotator. SNPs and indels were separated into two tables from the single merged and annotated VCF file using GATK SelectVariants, VariantFiltration, and VariantsToTable. Coverage across chromosomes was determined using GATK DepthOfCoverage on the sorted BAM files.

### Phylogenetic tree building.

SNPhylo (52), a pipeline designed to construct phylogenetic trees from SNP data, was used to generate a PHYLIP file from the original VCF. SNPhylo reduces redundant SNP information resulting from linkage disequilibrium. As we knew *a priori* that our ST93 samples were highly related, we ran SNPhylo with the linkage disequilibrium flag set at a very high value (0.99), which still reduced the number of SNPs by ∼94% on each chromosome. A total of 7,383 markers were selected. In SNPhylo, MUSCLE was used to perform multiple alignments and to generate the PHYLIP file.

Bootstrap analysis was conducted using RAxML. A total of 20 maximum likelihood (ML) trees were generated (-m ASC_GTRGAMMA –asc-corr=lewis), and support values from 100 bootstrap replicates were determined for the best-fitted ML tree (-m ASC_GTRGAMMA –asc-corr=lewis -p 3 -b 12345 -#100). Bipartitions were then drawn on the best tree (-m ASC_GTRGAMMA –asc-corr=lewis -p 3 -f b). This tree was read into R using the read.raxml command in the treeio library. Further tree visualizations were created using ggtree.

### Clinical data.

The methods of collection of clinical and immunological data were as described previously (26, 43). Clinical and immunological data used in this study are listed in Table 1. Briefly, the clinical parameters of disease were participant mortality due to cryptococcosis (days after initial diagnosis), CD4^+^ T-cell count, cerebrospinal fluid (CSF) white blood cell (WBC) count, serum and CSF protein levels, HIV load, CSF *Cryptococcus* clearance rate of early fungicidal activity (EFA), and lateral flow assay (LFA) measurement of CrAg titer (Immy Inc., Norman, OK). As immunological data, CSF levels of 19 cytokines and chemokines (granulocyte colony-stimulating factor [G-CSF], granulocyte-macrophage colony-stimulating factor [GM-CSF], interferon-γ, tumor necrosis factor alpha [TNF], interleukin-1β [IL-1β], IL-2, IL-4, IL-5, IL-6, IL-7, IL-8, IL-10, IL-12, IL-13, IL-17, monocyte chemoattractant protein 1 [MCP-1] [CCL2], macrophage inflammatory protein-1α [MIP-1α] [CCL3], MIP-1β [CCL4], and vascular endothelial growth factor [VEGF]) were analyzed. We refer to these cytokines and chemokines collectively as “cytokines.”

*In vitro* assays of drug resistance, macrophage adherence and uptake, cell wall chitin, and absolute growth were also performed on the clinical isolates. Drug resistance assays for fluconazole and amphotericin B were performed as described previously (25, 53). MH-S macrophage cell cultures were used to determine C. neoformans cell uptake by macrophages. Briefly, 5 × 10^5^ MH-S cells per well were incubated at 37°C with 5% CO_2_ for 2 h in a 96-well culture plate to allow adherence. C. neoformans cultures were grown overnight in Dulbecco’s modified Eagle medium (DMEM) supplemented with 2% glucose, collected by centrifugation, washed, and resuspended in 0.1% Uvitex solution for 10 min. Cells were then collected by centrifugation and washed, and 5 × 10^5^ cells and 4 μg E1 anti-GXM antibody (54) were added to each well in the MH-S culture plate. After 2 h of coincubation, the culture plate was centrifuged to collect cells, spent medium was decanted, and the mixtures were washed to remove extracellular C. neoformans cells. Samples were then resuspended in 0.25% trypsin–EDTA for 15 min to release the adherent cells from the wells and fixed with 3.7% formaldehyde for 30 min on ice. Samples were then stained with a second anti-GXM antibody (m18b7) conjugated to Alexa Fluor 488 fluorophore (1:2,000) and phycoerythrin (PE)-labeled CD45 (1:100) in a reaction mixture containing phosphate-buffered saline (PBS), 1 μg/ml bovine serum albumin (BSA), and 2 mM Tris-HCl. Cells were analyzed on a BD LSRII flow cytometer (BD Biosciences, Inc.), and data were analyzed using FlowJo software. Gating on Uvitex, CD45, and m18b7 allowed differentiation of (1) free C. neoformans cells (Uvitex positive [Uvitex^+^], CD45 negative [CD45^−^]), (2) free macrophages (Uvitex^−^, CD45^+^), (3) macrophages with intracellular C. neoformans (Uvitex^+^, CD45^+^, m18b7^−^), and (4) macrophages with extracellular C. neoformans (Uvitex^+^, CD45^+^, m18b7^+^). To analyze cell wall chitin content, C. neoformans cells were grown in DMEM supplemented with 2% glucose, 10% fetal bovine serum (FBS), 1% penicillin-streptomycin (Pen-Strep), and beta-mercaptoethanol (1 ml/liter) at 37°C overnight and were then fixed for 30 min in 3.7% formaldehyde. The cell concentration was adjusted to 1 × 10^6^ cells/ml, and the cells were stained with 1 μg/ml calcofluor white (Sigma-Aldrich)–PBS for 5 min at 25°C and then washed with PBS. The median calcofluor white fluorescence intensity was then determined for each strain by flow cytometric analysis of the cell population on an LSR II Fortessa flow cytometer.

Biomarkers analyzed as continuous variables were log_2_ transformed for normalization, analyzed again, and then back-transformed for calculation of geometric mean values. All “mean” biomarker values represent geometric means. Low (“out-of-range”) measurements were set to a value equal to half of the manufacturer's listed assay limit of detection (LOD).

### Survival curves.

Survival curve analyses were performed in three experiments that tested the virulence of strain KN99α (55) compared to single deletion strains in the following genes deleted: Experiment 1 (E1)—CNAG_00363, CNAG_02176, CNAG_04373, CNAG_04535, CNAG_04922, CNAG_05662, CNAG_05663, CNAG_05913, CNAG_06169, CNAG_06332, CNAG_06574, CNAG_06704, CNAG_06876, and CNAG_07837; Experiment 2 (E2)—CNAG_05973, CNAG_06490, CNAG_06986; Experiment 3 (E3)—CNAG_07703 (35). For E1, five C57BL/6 mice per group were anesthetized by intraperitoneal pentobarbital injection and inoculated intranasally with 5 × 10^4^ cells suspended in 50 μl PBS, whereas E2 and E3 used 1 × 10^4^ cells suspended in 50 μl PBS. Animals were monitored for morbidity and sacrificed with carbon dioxide when endpoint criteria were reached. Endpoint criteria were defined as 20% total body weight loss, loss of two grams of weight in 2 days, or symptoms of neurological damage. On day 34, the remaining mouse was sacrificed. Lungs and brain were removed and homogenized in 4 ml and 2 ml PBS, respectively. Serial dilutions of the lungs and of the entire homogenized brain were plated on YPD with chloramphenicol. CFU were counted after 48 h.

Significance was determined using the *survfit* command from the survival R package (56). Kaplan-Meier estimators from each knockout strain were compared to the data measured for the KN99α strain in the relevant experiment. *P* values were obtained by comparing the two curves using the G-rho family log rank test (57), implemented with the *survdiff* function.

### ITR4 survival curve.

Ten C57BL/6 mice per group were anesthetized and inoculated intranasally with 1 × 10^3^ KN99α, *itr4Δ*, or *itr4Δ:ITR4* cells suspended in 50 μl PBS. Animals were treated as described above. The *itr4Δ*-infected mice that survived the infection initially showed early signs of disease (minor weight loss, reduced activity) but regained weight at later time points. On day 44, the mice were sacrificed. Lungs and brain were collected from each mouse to determine fungal burden and processed as described above.

For determination of CFU counts at 7 days postinfection, 4 C57BL/6 mice per group were anesthetized and inoculated intranasally with 1 × 10^3^ KN99α, *itr4Δ*, or *itr4Δ:ITR4* cells suspended in 50 μl PBS. After 7 days, the mice were sacrificed, and lungs and brain were collected and processed as described above.

### Inositol growth assays.

Yeast cells of C. neoformans reference strain *KN99*α and the *itr4Δ* mutant and clinical strains were cultured in YPD medium overnight. Concentrations of overnight cultures were determined by measuring the optical density at 600 nm (OD_600_) and were adjusted to the same cell density. Serial 10-fold dilutions were prepared, and 5 μl of each dilution was spotted on yeast nitrogen base (YNB) plates with 1% glucose or, 1% inositol, 1% glucose + 1% inositol. Plates were then incubated at 30°C or 37°C for 48 h before photography was performed. The assay was repeated at least three times with similar results.

### Inositol uptake assay.

The inositol uptake assay was performed following a previously published method (31). In brief, the *Cryptococcus* strains were grown in YPD liquid cultures overnight at 30°C. Cells were diluted in YPD to an OD_600_ of 1.0, grown at 30°C, and collected at an OD_600_ of 5.0 by centrifugation at 2,600 × *g* for 5 min. Cells were then washed twice with PBS at 4°C and resuspended in 2% glucose to reach a final concentration of 2 × 10^8^ cells/ml as determined by the use of a hemacytometer. For the uptake assay, the reaction mixture (200 μl) contained 2% glucose, 40 mM citric acid-KH_2_PO_4_ (pH 5.5), and 0.15 μM myo-[2-^3^H]-inositol (MP Biomedicals) (1 μCi/μl). An additional 200 μM concentration of unlabeled inositol (Sigma-Aldrich) was added to the reaction mixtures for competition assays. Equal volumes of the reaction and cell mixtures (60 μl each) were warmed to 30°C and mixed for the uptake assay, which was performed for 10 min at 30°C. As negative controls, mixtures were kept at 0°C (on ice) during the 10-min incubation. Aliquots of 100 μl were removed and transferred onto prewetted Metricel filters (1.2-μm pore size) on a vacuum manifold. The filters were washed four times each with 2 ml of ice-cold water. The washed filters were removed and added to liquid scintillation vials for measurements on a PerkinElmer TRI-CARB 2900TR scintillation counter.

### Data availability.

All data and scripts are available at GitHub at https://github.com/acgerstein/UgClGenomics.
