# Supplementary material for: Identification of Pathogen Genomic Differences That Impact Human Immune Response and Disease during Cryptococcus neoformans Infection
Source: mBio. 2019 Jul 16;10(4):e01440-19. doi: 10.1128/mBio.01440-19 (PMC6635531; doi:10.1128/mBio.01440-19)
Supplement: TABLE S1 [file mBio.01440-19-st001.pdf]

Table S1. Genes with variants present in all ST93 genomes

name	chrom	chromStart	chromEnd	length	alias	description	freq.gene	freq.up	freq.syn	Freq
CNAG_00004	chr1	16736	19388	2652	null	hypothetical protein CNAG_00004	0	1	0	1
CNAG_00005	chr1	20906	22642	1736	null	TPR repeat-containing protein	0	1	0	1
CNAG_00008	chr1	28619	32831	4212	null	hypothetical protein CNAG_00008	0	3	0	3
CNAG_00010	chr1	36020	39419	3399	null	cation transporter	1	0	0	1
CNAG_00013	chr1	45340	46007	667	null	hypothetical protein CNAG_00013	0	1	0	1
CNAG_00015	chr1	50476	51193	717	null	hypothetical protein CNAG_00015	0	1	0	1
CNAG_00017	chr1	54702	57423	2721	null	hypothetical protein CNAG_00017	1	0	1	2
CNAG_00018	chr1	59229	61865	2636	null	hypothetical protein CNAG_00018	5	0	0	5
CNAG_00019	chr1	62033	62415	382	null	mitochondrial import inner membrane translocase subunit TIM9	0	1	0	1
CNAG_00020	chr1	62588	66221	3633	null	hypothetical protein CNAG_00020	2	0	0	2
CNAG_00043	chr1	130356	132334	1978	null	hypothetical protein CNAG_00043	0	1	0	1
CNAG_00073	chr1	201593	204477	2884	null	nuclear mRNA splicing protein	0	6	1	7
CNAG_00074	chr1	205680	208220	2540	null	protein PNS1	1	0	2	3
CNAG_00075	chr1	208635	209249	614	null	hypothetical protein CNAG_00075	0	3	1	4
CNAG_07947	chr1	212669	213106	437	null	hypothetical protein CNAG_07947	0	1	0	1
CNAG_00078	chr1	217128	221286	4158	null	vacuolar protein	0	1	2	3
CNAG_00079	chr1	223667	224474	807	null	hypothetical protein CNAG_00079	0	1	0	1
CNAG_00080	chr1	225551	227173	1622	null	hypothetical protein CNAG_00080	1	1	0	2
CNAG_00081	chr1	227666	230931	3265	null	pre-mRNA-processing factor 6	0	0	1	1
CNAG_00084	chr1	236469	239638	3169	null	glutamine-tRNA ligase	1	1	0	2
CNAG_00090	chr1	249912	251676	1764	null	hypothetical protein CNAG_00090	0	0	1	1
CNAG_00092	chr1	256069	256590	521	null	mitochondrial protein	0	3	0	3
**CNAG_00093**	**chr1**	**256916**	**259695**	**2779**	**null**	**hypothetical protein CNAG_00093**	**8**	**74**	**9**	**91**
**CNAG_00094**	**chr1**	**261285**	**262588**	**1303**	**null**	**NAD-dependent epimerase/dehydratase**	**1**	**15**	**16**	**32**
**CNAG_00095**	**chr1**	**262739**	**266546**	**3807**	**null**	**hypothetical protein CNAG_00095**	**5**	**0**	**9**	**14**
CNAG_00096	chr1	266961	268587	1626	null	hypothetical protein CNAG_00096	2	2	2	6
CNAG_00098	chr1	273829	275038	1209	null	palmitoyl-protein thioesterase	0	0	1	1
CNAG_00099	chr1	275157	278008	2851	null	minichromosome maintenance protein 3	0	1	1	2
CNAG_00101	chr1	280893	281839	946	null	hypothetical protein CNAG_00101	0	1	0	1
CNAG_00104	chr1	285901	289440	3539	null	elongation factor 3	0	0	1	1
CNAG_00105	chr1	289772	291138	1366	null	pyridoxal reductase	1	2	0	3
CNAG_00106	chr1	292159	296404	4245	TCO5	two-component system sensor protein	1	1	0	2
CNAG_07312	chr1	309264	312058	2794	null	hypothetical protein CNAG_07312	1	0	0	1
CNAG_00117	chr1	316825	318851	2026	ERG24	delta14-sterol reductase	2	0	2	4
CNAG_00119	chr1	319922	320592	670	null	hypothetical protein CNAG_00119	0	0	1	1
CNAG_00122	chr1	324829	326898	2069	null	alpha-amylase	0	1	2	3
CNAG_00127	chr1	341632	343375	1743	null	hypothetical protein CNAG_00127	0	1	0	1
CNAG_00132	chr1	360605	362888	2283	null	hypothetical protein CNAG_00132	0	3	0	3
CNAG_00133	chr1	364008	364781	773	null	hypothetical protein CNAG_00133	0	1	0	1
CNAG_00136	chr1	368615	372320	3705	null	ubiquitin-activating enzyme E1	0	1	0	1
CNAG_00137	chr1	372877	374191	1314	null	hypothetical protein CNAG_00137	1	0	0	1
CNAG_00140	chr1	379229	380739	1510	null	U1 small nuclear ribonucleoprotein 70kDa	0	1	0	1
CNAG_00142	chr1	383256	385933	2677	null	hypothetical protein CNAG_00142	2	0	0	2
CNAG_00143	chr1	386986	389134	2148	null	phosphoribosylaminoimidazolesuccinocarboxamide synthase	1	0	0	1
CNAG_00144	chr1	389309	390317	1008	null	hypothetical protein CNAG_00144	0	0	1	1

CNAG_00150	chr1	411943	415150	3207	null	peptidase	1	1	1	3
CNAG_00151	chr1	415523	418296	2773	null	transcription elongation regulator 1	4	0	3	7
CNAG_00152	chr1	418482	420087	1605	null	hypothetical protein CNAG_00152	1	1	0	2
CNAG_00154	chr1	422287	424170	1883	null	oxidoreductase	2	0	0	2
CNAG_00156	chr1	427608	431302	3694	SP1	hypothetical protein CNAG_00156	0	1	0	1
CNAG_00158	chr1	433036	435353	2317	null	glycosyltransferase	0	2	0	2
CNAG_00159	chr1	435868	436123	255	null	hypothetical protein CNAG_00159	0	1	0	1
CNAG_00160	chr1	436421	437840	1419	null	hypothetical protein CNAG_00160	0	0	1	1
CNAG_00161	chr1	437991	439360	1369	null	auxin-induced protein	0	1	1	2
CNAG_00162	chr1	439854	441268	1414	AOX1	alternative oxidase, mitochondrial	0	0	1	1
CNAG_00163	chr1	442897	446300	3403	null	general transcription factor 3C polypeptide 4	3	3	0	6
**CNAG_00164**	**chr1**	**447026**	**448723**	**1697**	**null**	**hypothetical protein CNAG_00164**	**0**	**24**	**1**	**25**
CNAG_00165	chr1	450185	451450	1265	null	methylthioadenosine phosphorylase	0	2	1	3
CNAG_00166	chr1	452494	454578	2084	null	ATP-dependent RNA helicase DBP8	2	1	2	5
CNAG_00167	chr1	454912	457078	2166	null	hypothetical protein CNAG_00167	2	1	4	7
CNAG_07315	chr1	459955	462704	2749	LIV6	hypothetical protein CNAG_07315	0	0	1	1
CNAG_00171	chr1	463029	464438	1409	null	peroxin-2	1	0	1	2
CNAG_00175	chr1	474659	476107	1448	null	hypothetical protein CNAG_00175	2	0	4	6
CNAG_00178	chr1	481142	485162	4020	null	DNA repair protein REV1	0	1	1	2
CNAG_00179	chr1	488572	489943	1371	GPA2	guanine nucleotide-binding protein subunit alpha	0	1	0	1
CNAG_00181	chr1	491751	493761	2010	FYV10	macrophage erythroblast attacher isoform 1	1	0	0	1
CNAG_00183	chr1	497481	498775	1294	null	alternative cyclin Pcl12	0	2	0	2
CNAG_00187	chr1	507083	509382	2299	UBP16	ubiquitin carboxyl-terminal hydrolase 1	0	0	1	1
CNAG_00188	chr1	509423	510478	1055	null	endoplasmic reticulum protein	0	0	1	1
CNAG_00190	chr1	512338	514159	1821	null	hypothetical protein CNAG_00190	0	2	0	2
CNAG_00192	chr1	517793	521395	3602	null	hypothetical protein CNAG_00192	0	2	0	2
CNAG_07321	chr1	545312	548518	3206	null	hypothetical protein CNAG_07321	1	0	0	1
CNAG_07323	chr1	551679	554213	2534	null	cactin	1	0	0	1
CNAG_07332	chr1	572025	574718	2693	null	hypothetical protein CNAG_07332	0	1	0	1
CNAG_07333	chr1	575093	580250	5157	null	ubiquitin carboxyl-terminal hydrolase 25/28	0	0	1	1
CNAG_07027	chr1	593866	595294	1428	null	protein SPT2	0	0	1	1
CNAG_07340	chr1	599292	599678	386	null	hypothetical protein CNAG_07340	0	1	0	1
CNAG_00234	chr1	613927	615256	1329	null	tRNA-dihydrouridine synthase 4	0	0	1	1
CNAG_00235	chr1	615351	617537	2186	AMT1	amt family ammonium transporter	0	1	0	1
CNAG_00239	chr1	628128	630490	2362	null	hypothetical protein CNAG_00239	0	1	0	1
CNAG_00240	chr1	630798	634045	3247	null	hypothetical protein CNAG_00240	0	1	0	1
CNAG_07344	chr1	642832	646263	3431	null	ras guanyl-nucleotide exchange factor	0	1	0	1
CNAG_00255	chr1	667625	668043	418	null	hypothetical protein CNAG_00255	0	1	0	1
CNAG_00257	chr1	669541	675329	5788	null	kinesin family member 21A	1	1	2	4
CNAG_00258	chr1	676394	680311	3917	null	hypothetical protein CNAG_00258	0	0	1	1
CNAG_00263	chr1	689599	690700	1101	null	serine/threonine/tyrosine-interacting protein	0	1	0	1
CNAG_00267	chr1	696435	698233	1798	null	hypothetical protein CNAG_00267	0	0	1	1
CNAG_00276	chr1	717809	718990	1181	null	hypothetical protein CNAG_00276	1	0	0	1
CNAG_00288	chr1	745984	746917	933	null	oligoribonuclease	0	1	0	1
CNAG_07349	chr1	765989	767223	1234	null	hypothetical protein CNAG_07349	1	0	0	1
CNAG_00300	chr1	772002	775338	3336	null	gamma-tubulin complex component 2	0	0	1	1
CNAG_00308	chr1	790860	792851	1991	null	gly-X carboxypeptidase	0	0	1	1

CNAG_00313	chr1	803371	806503	3132	null	hypothetical protein CNAG_00313	1	0	0	1
CNAG_00326	chr1	833626	835621	1995	null	hypothetical protein CNAG_00326	0	1	0	1
CNAG_00330	chr1	843583	845386	1803	null	transcription factor TFIIIB component b\'\'	1	0	0	1
CNAG_00332	chr1	848559	852220	3661	null	hypothetical protein CNAG_00332	0	1	1	2
CNAG_00335	chr1	860082	861484	1402	null	hypothetical protein CNAG_00335	0	1	0	1
CNAG_00337	chr1	865584	868458	2874	null	hypothetical protein CNAG_00337	0	0	1	1
CNAG_07352	chr1	869003	870059	1056	null	hypothetical protein CNAG_07352	0	0	1	1
CNAG_07353	chr1	870118	871091	973	null	26S proteasome non-ATPase regulatory subunit 9	0	1	0	1
CNAG_00342	chr1	874981	877529	2548	null	hypothetical protein CNAG_00342	0	0	1	1
CNAG_00343	chr1	877920	879310	1390	null	hypothetical protein CNAG_00343	0	1	0	1
CNAG_00347	chr1	886207	891417	5210	null	DEAH box polypeptide 36	1	0	2	3
CNAG_07356	chr1	895486	896310	824	null	succinate dehydrogenase, cytochrome b556 subunit	1	0	0	1
CNAG_07253	chr1	896521	897474	953	null	hypothetical protein CNAG_07253	0	0	1	1
CNAG_00364	chr1	928880	930286	1406	null	tRNA threonylcarbamoyladenosine biosynthesis protein	0	0	1	1
CNAG_00374	chr1	951148	953086	1938	null	hypothetical protein CNAG_00374	0	0	1	1
CNAG_00377	chr1	960400	965385	4985	null	DNA topoisomerase II	0	4	0	4
CNAG_07950	chr1	976947	977798	851	null	hypothetical protein CNAG_07950	0	3	0	3
CNAG_07358	chr1	1004400	1005094	694	null	hypothetical protein CNAG_07358	0	2	0	2
CNAG_00387	chr1	1019438	1021867	2429	null	hypothetical protein CNAG_00387	0	0	1	1
CNAG_00388	chr1	1022092	1024835	2743	null	1-phosphatidylinositol-4-phosphate 5-kinase	1	3	0	4
CNAG_07951	chr1	1026293	1027739	1446	null	WD-repeat protein JIP5	0	0	1	1
CNAG_00391	chr1	1030456	1031601	1145	null	hypothetical protein CNAG_00391	0	2	0	2
CNAG_00392	chr1	1031778	1033485	1707	null	kelch repeat-containing protein	1	0	0	1
CNAG_00393	chr1	1033937	1036850	2913	null	1,4-alpha-glucan-branching enzyme	1	2	0	3
CNAG_00394	chr1	1037392	1037911	519	null	diphthamide biosynthesis protein 3	0	1	0	1
CNAG_00395	chr1	1038038	1039781	1743	null	structure-specific endonuclease subunit SLX1	3	2	0	5
CNAG_00396	chr1	1040370	1042329	1959	PKA1	AGC/PKA protein kinase	0	1	0	1
CNAG_00397	chr1	1043270	1045035	1765	null	2-oxoisovalerate dehydrogenase E1 component, beta subunit	0	0	1	1
CNAG_00398	chr1	1045238	1046318	1080	null	hypothetical protein CNAG_00398	0	2	0	2
CNAG_00399	chr1	1047225	1048849	1624	null	transformer-2-beta isoform 3	1	0	0	1
CNAG_00400	chr1	1050472	1051379	907	null	secondary thiamine-phosphate synthase enzyme	0	0	1	1
CNAG_00401	chr1	1051433	1053159	1726	null	phosphatidylinositol glycan, class U	0	0	1	1
CNAG_00402	chr1	1053739	1058486	4747	null	translation initiation factor 4G	1	0	1	2
CNAG_00403	chr1	1059161	1060952	1791	null	mitochondrial protein	1	0	2	3
CNAG_00404	chr1	1061805	1063875	2070	null	RING zinc finger protein	0	1	1	2
CNAG_00405	chr1	1064036	1067169	3133	KIC1	STE/STE20/YSK protein kinase	2	0	3	5
CNAG_00406	chr1	1067315	1070103	2788	null	hypothetical protein CNAG_00406	1	1	0	2
CNAG_00407	chr1	1071048	1073410	2362	null	glyoxal oxidase	1	0	0	1
CNAG_00409	chr1	1076718	1077969	1251	null	hypothetical protein CNAG_00409	0	1	0	1
CNAG_00410	chr1	1078249	1081100	2851	null	hypothetical protein CNAG_00410	0	3	0	3
CNAG_00413	chr1	1085131	1087389	2258	OFD1	nuclear protein	0	2	0	2
CNAG_00417	chr1	1094549	1096292	1743	null	elongation factor 1-gamma	0	1	0	1
CNAG_00421	chr1	1102100	1102999	899	null	charged multivesicular body protein 3	0	1	0	1
CNAG_00422	chr1	1103304	1107204	3900	null	hypothetical protein CNAG_00422	1	0	0	1
CNAG_00423	chr1	1108125	1109003	878	null	kinetochore protein Spc24, fungi type	0	1	0	1
CNAG_00425	chr1	1111224	1112489	1265	null	hypothetical protein CNAG_00425	0	0	1	1
CNAG_00426	chr1	1112743	1115306	2563	null	UBA/TS-N domain-containing protein	0	1	0	1

CNAG_00466	chr1	1209299	1210069	770	null	adaptin ear-binding coat-associated protein 2	0	1	0	1
CNAG_00474	chr1	1220031	1221105	1074	null	hypothetical protein CNAG_00474	1	0	0	1
CNAG_00482	chr1	1241052	1242310	1258	null	26S proteasome regulatory subunit N10	0	1	0	1
CNAG_00504	chr1	1287972	1289624	1652	null	peptidyl-prolyl cis-trans isomerase-like 4	0	0	1	1
CNAG_00509	chr1	1300209	1301823	1614	null	translation initiation factor 3 subunit M	0	1	1	2
CNAG_00512	chr1	1305539	1307282	1743	null	mitochondrial carrier protein	0	0	1	1
CNAG_00520	chr1	1325020	1330280	5260	null	hypothetical protein CNAG_00520	0	2	0	2
CNAG_00522	chr1	1333192	1336895	3703	null	C2 domain-containing protein	2	0	1	3
CNAG_00527	chr1	1345836	1348215	2379	null	translation initiation factor eIF-2B subunit epsilon	1	0	0	1
CNAG_00535	chr1	1365312	1366288	976	null	large subunit ribosomal protein L16	1	0	0	1
CNAG_00536	chr1	1366906	1367539	633	null	hypothetical protein CNAG_00536	0	1	0	1
CNAG_00539	chr1	1372146	1374367	2221	null	membrane transporter	1	2	0	3
CNAG_00541	chr1	1380548	1382373	1825	null	dimethylaniline monooxygenase	0	1	1	2
CNAG_00542	chr1	1383091	1384908	1817	null	salicylate hydroxylase	1	0	0	1
CNAG_00543	chr1	1385674	1386516	842	null	hypothetical protein CNAG_00543	0	0	1	1
CNAG_00544	chr1	1386636	1388404	1768	null	hypothetical protein CNAG_00544	0	1	0	1
CNAG_00545	chr1	1389395	1396434	7039	null	cohesin loading factor subunit SCC2	0	0	1	1
CNAG_00546	chr1	1397884	1402238	4354	CHS4	chitin synthase	1	0	0	1
CNAG_00547	chr1	1402747	1406925	4178	null	hypothetical protein CNAG_00547	2	0	1	3
CNAG_00550	chr1	1414393	1417894	3501	null	DNA mismatch repair protein MSH5	0	1	0	1
CNAG_00551	chr1	1418394	1420854	2460	null	poly(A) polymerase	0	3	0	3
CNAG_07367	chr1	1422312	1424374	2062	null	amino acid transporter	0	1	1	2
CNAG_00556	chr1	1434372	1436338	1966	CCK1	CK1/CK1/CK1-G protein kinase	0	0	1	1
CNAG_00559	chr1	1444617	1445704	1087	null	hypothetical protein CNAG_00559	1	0	1	2
CNAG_00560	chr1	1446174	1447237	1063	null	V-type H -transporting ATPase subunit E	1	1	0	2
CNAG_00563	chr1	1451540	1452544	1004	null	hypothetical protein CNAG_00563	1	0	0	1
CNAG_00564	chr1	1453599	1455614	2015	null	hypothetical protein CNAG_00564	0	1	0	1
CNAG_00567	chr1	1462858	1464368	1510	null	WD40 repeat protein	0	1	0	1
CNAG_00570	chr1	1468943	1470619	1676	PKR1	cAMP-dependent protein kinase regulator	0	0	1	1
CNAG_00571	chr1	1470961	1473455	2494	null	hypothetical protein CNAG_00571	0	1	0	1
CNAG_00574	chr1	1479373	1482187	2814	null	amino acid/metabolite permease	0	7	0	7
CNAG_00575	chr1	1483468	1486076	2608	CAT3	catalase	0	1	1	2
CNAG_07369	chr1	1490766	1491948	1182	null	hypothetical protein CNAG_07369	1	0	1	2
CNAG_00580	chr1	1492251	1495182	2931	null	translation initiation factor IF-2	1	2	0	3
CNAG_00582	chr1	1498614	1499574	960	null	vacuolar transporter chaperone 1	0	1	0	1
CNAG_00583	chr1	1500279	1503651	3372	null	hypothetical protein CNAG_00583	1	0	0	1
CNAG_00585	chr1	1506247	1507297	1050	null	hypothetical protein CNAG_00585	0	1	0	1
CNAG_00586	chr1	1507859	1508397	538	null	hypothetical protein CNAG_00586	1	1	0	2
CNAG_00587	chr1	1508885	1509614	729	null	hypothetical protein CNAG_00587	0	4	0	4
CNAG_00588	chr1	1511997	1512725	728	null	hypothetical protein CNAG_00588	0	1	0	1
CNAG_00591	chr1	1521073	1522449	1376	null	hypothetical protein CNAG_00591	1	0	0	1
CNAG_00593	chr1	1524145	1525500	1355	null	hypothetical protein CNAG_00593	1	1	0	2
CNAG_00594	chr1	1525900	1528108	2208	null	lipid particle protein	0	1	0	1
CNAG_00595	chr1	1529446	1530958	1512	null	hypothetical protein CNAG_00595	1	0	0	1
CNAG_00597	chr1	1534561	1536623	2062	DIP5	amino acid transporter	0	0	1	1
CNAG_00598	chr1	1538820	1540760	1940	null	nicotinamide mononucleotide permease	0	1	0	1
CNAG_00600	chr1	1544357	1546235	1878	CAP60	capsular associated protein	0	1	0	1

CNAG_00601	chr1	1546728	1548198	1470	CEL1	glycosyl hydrolase	0	1	0	1
CNAG_00603	chr1	1550413	1551496	1083	null	pre-rRNA-processing protein PNO1	1	0	0	1
CNAG_00604	chr1	1551879	1553792	1913	null	hypothetical protein CNAG_00604	0	1	0	1
CNAG_00606	chr1	1557138	1559655	2517	null	transcription initiation factor TFIID subunit 7	1	0	0	1
CNAG_00607	chr1	1559765	1561587	1822	null	hypothetical protein CNAG_00607	0	1	0	1
CNAG_00609	chr1	1562694	1566500	3806	null	hypothetical protein CNAG_00609	0	1	0	1
CNAG_07956	chr1	1569339	1571912	2573	null	WD repeat and SOF domain-containing protein 1	0	1	1	2
CNAG_00613	chr1	1577213	1578041	828	FCY1	cytosine deaminase	0	1	0	1
CNAG_00619	chr1	1582928	1584456	1528	null	tubulin folding cofactor C	0	0	1	1
CNAG_07373	chr1	1584888	1592226	7338	null	carbamoyl-phosphate synthase, large subunit	1	1	1	3
CNAG_00622	chr1	1594084	1595573	1489	ARG8	acetylornithine aminotransferase	1	0	1	2
CNAG_00623	chr1	1595674	1598729	3055	EGCrP1	cytoplasmic protein	0	0	1	1
CNAG_00625	chr1	1600864	1606646	5782	null	ARF guanyl-nucleotide exchange factor	0	0	2	2
CNAG_00627	chr1	1609523	1611827	2304	null	specific transcriptional repressor	1	0	0	1
CNAG_07375	chr1	1612961	1617276	4315	null	guanyl-nucleotide exchange factor	1	0	0	1
CNAG_07377	chr1	1620412	1632337	11925	TRA1	transformation/transcription domain-associated protein	0	1	1	2
CNAG_00632	chr1	1632780	1633611	831	null	hypothetical protein CNAG_00632	1	0	0	1
CNAG_00633	chr1	1633888	1638454	4566	null	hypothetical protein CNAG_00633	1	0	0	1
CNAG_00635	chr1	1641789	1646163	4374	null	cytoplasmic protein	1	0	1	2
CNAG_00640	chr1	1656588	1657697	1109	null	small subunit ribosomal protein S4-A	0	1	0	1
CNAG_00641	chr1	1658271	1662312	4041	null	transcription elongation factor SPT5	0	0	1	1
CNAG_00642	chr1	1664119	1675519	11400	null	hypothetical protein CNAG_00642	1	1	0	2
CNAG_00643	chr1	1675864	1678772	2908	null	hypothetical protein CNAG_00643	1	0	1	2
CNAG_00644	chr1	1679062	1680485	1423	null	sphingolipid delta-4 desaturase	1	0	1	2
CNAG_07959	chr1	1682371	1685547	3176	null	hypothetical protein CNAG_07959	1	0	1	2
CNAG_00647	chr1	1685760	1686256	496	null	hypothetical protein CNAG_00647	0	1	0	1
CNAG_00649	chr1	1688094	1690677	2583	null	tryptophan synthase, beta subunit	0	3	0	3
CNAG_00651	chr1	1694356	1697782	3426	null	ATP-binding cassette, subfamily D (ALD), peroxisomal long-chain fatty acid import protein	0	0	2	2
CNAG_00653	chr1	1701095	1704056	2961	null	hypothetical protein CNAG_00653	0	3	0	3
CNAG_00654	chr1	1705239	1705730	491	SRX1	sulfiredoxin	0	1	0	1
CNAG_00657	chr1	1708978	1709762	784	null	hypothetical protein CNAG_00657	0	0	0	0
CNAG_00658	chr1	1710449	1712659	2210	null	hypothetical protein CNAG_00658	0	1	0	1
CNAG_00660	chr1	1713340	1716027	2687	null	histone deacetylase HOS3	1	0	0	1
CNAG_00662	chr1	1718893	1720152	1259	null	carboxymethylenebutenolidase	0	4	0	4
CNAG_00663	chr1	1720880	1722400	1520	null	hypothetical protein CNAG_00663	0	0	2	2
**CNAG_00664**	**chr1**	**1723252**	**1725606**	**2354**	**null**	**hypothetical protein CNAG_00664**	**19**	**0**	**9**	**28**
CNAG_00665	chr1	1725974	1728798	2824	null	DNA topoisomerase 2-associated protein PAT1	0	3	2	5
CNAG_00666	chr1	1729589	1734337	4748	null	nucleoporin nsp1	2	0	0	2
CNAG_00667	chr1	1734797	1736980	2183	null	hypothetical protein CNAG_00667	1	0	0	1
CNAG_00669	chr1	1740515	1741269	754	null	hypothetical protein CNAG_00669	1	1	0	2
CNAG_00671	chr1	1743982	1746660	2678	null	cytoplasmic protein	1	1	0	2
CNAG_00674	chr1	1750408	1752237	1829	null	hypothetical protein CNAG_00674	1	0	0	1
CNAG_00678	chr1	1762205	1763515	1310	URE7	urease accessory protein UreG	0	1	0	1
CNAG_00682	chr1	1772139	1778248	6109	null	kinesin	1	0	0	1
CNAG_00683	chr1	1779417	1782113	2696	null	CMGC/CLK protein kinase	1	0	0	1
CNAG_00687	chr1	1788072	1792772	4700	null	hypothetical protein CNAG_00687	2	0	0	2
CNAG_00688	chr1	1792919	1793738	819	null	hypothetical protein CNAG_00688	0	1	0	1

CNAG_00696	chr1	1811919	1816687	4768	null	alpha-mannosidase	1	0	0	1
CNAG_00698	chr1	1819918	1822366	2448	null	hypothetical protein CNAG_00698	0	1	0	1
CNAG_00699	chr1	1822784	1827321	4537	null	transmembrane receptor	0	1	0	1
CNAG_00703	chr1	1839818	1840750	932	null	large subunit ribosomal protein L31e	0	1	0	1
CNAG_00707	chr1	1850044	1851083	1039	null	protein SYM1	0	1	0	1
CNAG_00713	chr1	1862292	1863976	1684	null	V-type H -transporting ATPase 54 kDa subunit	0	0	1	1
CNAG_00714	chr1	1864230	1865426	1196	null	hypothetical protein CNAG_00714	1	0	0	1
CNAG_00715	chr1	1866026	1866468	442	null	hypothetical protein CNAG_00715	0	2	0	2
CNAG_00716	chr1	1866813	1867660	847	null	cytochrome c	0	1	0	1
CNAG_00730	chr1	1901529	1907595	6066	AFR1	ATP-binding cassette transporter	1	0	0	1
CNAG_00732	chr1	1909284	1910521	1237	null	hypothetical protein CNAG_00732	1	1	0	2
CNAG_00736	chr1	1922249	1925599	3350	null	exocyst protein	1	0	1	2
CNAG_00745	chr1	1953608	1957242	3634	HRK1	HAL protein kinase	0	1	0	1
CNAG_00748	chr1	1962552	1963949	1397	null	hypothetical protein CNAG_00748	1	2	0	3
CNAG_00753	chr1	1974597	1977345	2748	null	hypothetical protein CNAG_00753	2	0	0	2
CNAG_00754	chr1	1977648	1979859	2211	null	ATP-binding cassette, sub-family E, member 1	0	2	0	2
CNAG_00757	chr1	1984123	1988735	4612	DOA4	ubiquitin carboxyl-terminal hydrolase 8	0	0	2	2
CNAG_00758	chr1	1991621	1995118	3497	null	regulatory protein ral2	1	0	0	1
CNAG_00759	chr1	1995540	1997246	1706	null	hypothetical protein CNAG_00759	1	0	0	1
CNAG_07960	chr1	2003336	2005202	1866	null	hypothetical protein CNAG_07960	0	1	0	1
CNAG_00765	chr1	2008525	2009452	927	null	hypothetical protein CNAG_00765	1	0	0	1
CNAG_00768	chr1	2016264	2017451	1187	null	hypothetical protein CNAG_00768	0	2	0	2
CNAG_00769	chr1	2018003	2020204	2201	PBS2	STE/STE7 protein kinase	0	1	0	1
CNAG_00770	chr1	2021389	2025285	3896	null	DNA mismatch repair protein MSH2	0	0	1	1
CNAG_00774	chr1	2031278	2033154	1876	null	phosphoglycerate dehydrogenase	0	1	0	1
CNAG_00775	chr1	2033870	2035556	1686	null	U3 small nucleolar RNA-associated protein 15	1	0	0	1
CNAG_00777	chr1	2040201	2045595	5394	null	CCR4-NOT transcriptional complex subunit CAF120	0	0	1	1
CNAG_00782	chr1	2056818	2059656	2838	null	STE/STE20/Fray protein kinase	0	0	1	1
CNAG_00783	chr1	2060189	2061628	1439	null	hypothetical protein CNAG_00783	0	1	0	1
CNAG_00785	chr1	2063601	2065325	1724	null	ATP-dependent RNA helicase eIF4A	1	0	0	1
CNAG_00789	chr1	2076571	2077687	1116	null	hypothetical protein CNAG_00789	0	1	0	1
CNAG_00791	chr1	2081273	2082633	1360	null	hypothetical protein CNAG_00791	0	2	0	2
CNAG_00792	chr1	2087805	2094531	6726	null	ATP-binding cassette transporter	1	1	0	2
CNAG_00793	chr1	2094929	2096130	1201	null	DNA-directed RNA polymerase I, II, and III subunit RPABC1	0	3	1	4
CNAG_00796	chr1	2101462	2106512	5050	MDR1	ATP-binding cassette, subfamily B (MDR/TAP), member 1	0	0	1	1
CNAG_00797	chr1	2108173	2110606	2433	null	acetyl-CoA synthetase	0	0	1	1
CNAG_00800	chr1	2118415	2119369	954	null	nicotinamidase	0	2	0	2
CNAG_00805	chr1	2128994	2131387	2393	null	hypothetical protein CNAG_00805	1	4	0	5
CNAG_00806	chr1	2131721	2132489	768	null	hypothetical protein CNAG_00806	1	0	0	1
CNAG_00823	chr1	2179310	2185060	5750	null	cadmium ion transporter	1	0	0	1
CNAG_00829	chr1	2200171	2201246	1075	null	E3 ubiquitin ligase complex SCF subunit sconC	1	0	0	1
CNAG_00849	chr1	2250127	2250626	499	null	hypothetical protein CNAG_00849	0	1	0	1
CNAG_00850	chr1	2251267	2252869	1602	null	plant-inducible protein	0	1	0	1
CNAG_00851	chr1	2253124	2254143	1019	null	hypothetical protein CNAG_00851	0	1	0	1
CNAG_00853	chr1	2256869	2258266	1397	null	exosome complex component RRP43	0	0	1	1
CNAG_00854	chr1	2258878	2259890	1012	ERG2	C-8 sterol isomerase	0	6	0	6
CNAG_00855	chr1	2260511	2263902	3391	null	ligase	0	0	1	1

CNAG_07387	chr1	2264305	2266895	2590	null	siderophore-iron transporter Str3	1	0	1	2
CNAG_07837	chr10	13687	14776	1089	null	hypothetical protein CNAG_07837	0	1	0	1
CNAG_04921	chr10	19344	20694	1350	null	hypothetical protein CNAG_04921	0	1	0	1
CNAG_04916	chr10	33922	36215	2293	null	hypothetical protein CNAG_04916	0	1	0	1
CNAG_07840	chr10	48150	48428	278	null	hypothetical protein CNAG_07840	0	0	1	1
CNAG_04907	chr10	57169	59307	2138	null	hypothetical protein CNAG_04907	1	0	0	1
CNAG_07842	chr10	63703	64558	855	null	hypothetical protein CNAG_07842	0	1	0	1
CNAG_04903	chr10	71891	72492	601	null	hypothetical protein CNAG_04903	0	1	0	1
CNAG_04899	chr10	79360	80687	1327	null	cytoplasmic protein	0	1	0	1
CNAG_04898	chr10	82310	83827	1517	null	MFS transporter	1	0	0	1
CNAG_04895	chr10	88824	92541	3717	null	nuclear protein	0	0	1	1
CNAG_04894	chr10	93831	94256	425	null	hypothetical protein CNAG_04894	0	1	0	1
CNAG_04890	chr10	100361	102055	1694	null	hypothetical protein CNAG_04890	0	0	1	1
CNAG_04888	chr10	104754	105977	1223	null	hypothetical protein CNAG_04888	1	1	0	2
CNAG_04886	chr10	108075	109019	944	null	hypothetical protein CNAG_04886	0	1	0	1
CNAG_04884	chr10	113852	114668	816	null	large subunit ribosomal protein L44	0	1	0	1
CNAG_04879	chr10	122775	128453	5678	null	glycogen debranching enzyme	1	0	1	2
CNAG_04878	chr10	129029	131157	2128	null	hypothetical protein CNAG_04878	0	2	0	2
CNAG_04875	chr10	137557	140485	2928	null	hypothetical protein CNAG_04875	0	1	0	1
CNAG_04873	chr10	145289	146998	1709	null	hypothetical protein CNAG_04873	0	0	1	1
CNAG_04872	chr10	147333	148843	1510	null	mitochondrial protein	1	1	0	2
CNAG_04871	chr10	149234	150052	818	null	hypothetical protein CNAG_04871	0	2	0	2
**CNAG_04870**	**chr10**	**150964**	**153778**	**2814**	**null**	**hypothetical protein CNAG_04870**	**7**	**3**	**3**	**13**
CNAG_04869	chr10	154331	157112	2781	PNB1	carboxylesterase	2	6	0	8
CNAG_04868	chr10	158213	160773	2560	null	cytoplasmic protein	1	0	0	1
CNAG_04867	chr10	161167	163144	1977	null	vacuolar protein	1	0	0	1
CNAG_04865	chr10	167056	169308	2252	null	mannitol dehydrogenase	1	0	0	1
CNAG_04863	chr10	178057	179186	1129	VPS25	ESCRT-II complex subunit VPS25	0	0	1	1
CNAG_04862	chr10	181272	188080	6808	null	glutamate synthase (NADPH/NADH)	0	1	1	2
CNAG_04861	chr10	189220	190731	1511	null	transglycosylase SLT domain-containing protein	1	0	0	1
CNAG_04860	chr10	191111	194170	3059	XPT1	hypothetical protein CNAG_04860	1	0	1	2
CNAG_04857	chr10	197373	199130	1757	null	hypothetical protein CNAG_04857	1	2	0	3
CNAG_04854	chr10	232071	232567	496	null	hypothetical protein CNAG_04854	0	3	0	3
CNAG_04847	chr10	247100	248896	1796	null	hypothetical protein CNAG_04847	1	0	0	1
CNAG_04845	chr10	252348	253431	1083	null	mannose-P-dolichol utilization defect 1	0	1	0	1
CNAG_04844	chr10	253638	254234	596	null	mitochondrial import receptor subunit tom22	0	1	0	1
CNAG_04843	chr10	254647	256938	2291	null	Atypical/ABC1/ABC1-B protein kinase	1	0	0	1
CNAG_04842	chr10	257423	259285	1862	null	hypothetical protein CNAG_04842	0	1	0	1
CNAG_04841	chr10	261229	264729	3500	null	transcriptional regulatory protein	1	0	0	1
CNAG_04838	chr10	268918	271617	2699	null	hypothetical protein CNAG_04838	0	1	0	1
CNAG_04837	chr10	272604	274410	1806	MLN1	hypothetical protein CNAG_04837	0	0	1	1
CNAG_04836	chr10	276708	279401	2693	null	nuclear protein	1	1	0	2
CNAG_04835	chr10	280893	281993	1100	null	dihydrodipicolinate synthase	0	1	0	1
CNAG_04834	chr10	282587	284307	1720	null	MFS transporter	1	0	0	1
CNAG_04825	chr10	302059	303476	1417	null	hypothetical protein CNAG_04825	0	1	0	1
CNAG_04823	chr10	306229	307677	1448	null	peptidyl-prolyl cis-trans isomerase H	0	1	0	1
CNAG_07845	chr10	320689	321503	814	null	hypothetical protein CNAG_07845	0	1	1	2

CNAG_04815	chr10	322938	328931	5993	BNI1	cytokinesis protein	2	1	0	3
CNAG_04813	chr10	329717	330845	1128	null	V-type ATPase, C subunit	0	5	0	5
CNAG_07847	chr10	333491	334293	802	null	hypothetical protein CNAG_07847	1	6	0	7
CNAG_04810	chr10	336658	336990	332	null	hypothetical protein CNAG_04810	0	1	2	3
CNAG_04809	chr10	337247	338850	1603	null	COP9 signalosome complex subunit 5	0	0	1	1
CNAG_04808	chr10	339372	342048	2676	null	XPG N-terminal domain-containing protein	0	2	3	5
CNAG_04807	chr10	344828	346024	1196	null	hypothetical protein CNAG_04807	0	2	0	2
CNAG_04805	chr10	349871	351948	2077	null	peptidyl-prolyl isomerase CWC27	0	0	1	1
CNAG_04804	chr10	352256	355441	3185	SRE1	hypothetical protein CNAG_04804	1	0	0	1
CNAG_04803	chr10	357069	361792	4723	null	protein transporter SEC31	2	5	0	7
CNAG_04801	chr10	364203	366424	2221	null	hypothetical protein CNAG_04801	0	3	0	3
CNAG_04796	chr10	376381	379013	2632	CNA1	serine/threonine-protein phosphatase 2B catalytic subunit A1	0	0	1	1
CNAG_04794	chr10	381415	383533	2118	null	spermine transporter	0	2	0	2
CNAG_04791	chr10	391294	392531	1237	null	hypothetical protein CNAG_04791	0	0	1	1
CNAG_04786	chr10	404760	408406	3646	null	hypothetical protein CNAG_04786	0	1	1	2
CNAG_04784	chr10	409609	411799	2190	null	monosaccharide transporter	0	1	0	1
CNAG_04782	chr10	417303	420112	2809	null	hypothetical protein CNAG_04782	1	0	0	1
CNAG_04781	chr10	421704	423119	1415	null	hypothetical protein CNAG_04781	0	1	0	1
CNAG_07848	chr10	423662	426019	2357	null	hypothetical protein CNAG_07848	1	2	0	3
CNAG_04777	chr10	432736	434274	1538	null	S-adenosylmethionine-dependent methyltransferase	1	0	0	1
CNAG_04774	chr10	437190	441379	4189	null	nuclear protein	1	0	1	2
CNAG_04773	chr10	443623	445518	1895	null	hypothetical protein CNAG_04773	0	0	1	1
CNAG_04770	chr10	451460	452771	1311	null	nucleolar essential protein 1	1	0	0	1
CNAG_04767	chr10	460758	462091	1333	null	hypothetical protein CNAG_04767	1	0	0	1
CNAG_04766	chr10	462521	463033	512	null	hypothetical protein CNAG_04766	0	1	0	1
CNAG_04760	chr10	471990	473144	1154	null	cytoplasmic protein	0	1	0	1
CNAG_04759	chr10	474185	476068	1883	null	hypothetical protein CNAG_04759	1	0	1	2
CNAG_04758	chr10	477936	479720	1784	AMT2	amt family ammonium transporter	0	2	0	2
CNAG_04757	chr10	480145	481330	1185	null	mitochondrial protein	0	1	0	1
CNAG_04755	chr10	486568	492318	5750	BCK1	STE/STE11/BCK1 protein kinase	0	2	0	2
CNAG_04753	chr10	497576	499456	1880	null	gluconolactonase	0	2	1	3
CNAG_04752	chr10	502886	504229	1343	null	hypothetical protein CNAG_04752	2	0	1	3
CNAG_07853	chr10	506082	506358	276	null	hypothetical protein CNAG_07853	0	0	1	1
CNAG_04747	chr10	512676	514481	1805	null	hypothetical protein CNAG_04747	0	1	0	1
CNAG_04746	chr10	515150	517166	2016	null	hypothetical protein CNAG_04746	1	0	0	1
CNAG_04742	chr10	523993	525699	1706	null	DNA primase large subunit	0	0	2	2
CNAG_04741	chr10	526624	528771	2147	null	endosome protein	1	0	1	2
CNAG_04740	chr10	529498	532968	3470	null	hypothetical protein CNAG_04740	0	0	1	1
CNAG_04733	chr10	549226	553811	4585	null	DNA repair protein RAD5	1	0	0	1
CNAG_04730	chr10	558260	561329	3069	GPR4	hypothetical protein CNAG_04730	1	0	1	2
CNAG_04729	chr10	562690	565642	2952	null	ATP-dependent RNA helicase DBP7	0	2	0	2
CNAG_04725	chr10	571117	574160	3043	null	hypothetical protein CNAG_04725	0	1	0	1
CNAG_04724	chr10	574783	578931	4148	null	ubiquitin-protein ligase E3 C	0	0	2	2
CNAG_04723	chr10	579160	581019	1859	null	hypothetical protein CNAG_04723	1	0	0	1
CNAG_04721	chr10	588593	590503	1910	null	hypothetical protein CNAG_04721	0	1	0	1
CNAG_04720	chr10	591034	595059	4025	null	GTPase activating protein	1	0	0	1
CNAG_04718	chr10	598625	599545	920	null	hypothetical protein CNAG_04718	0	1	0	1

CNAG_04717	chr10	601030	602980	1950	null	hypothetical protein CNAG_04717	0	1	0	1
CNAG_04716	chr10	603483	604968	1485	null	KH domain-containing protein	0	0	1	1
CNAG_04712	chr10	612468	614011	1543	null	solute carrier family 25 (mitochondrial dicarboxylate transporter), member 10	0	0	1	1
CNAG_04711	chr10	614660	616410	1750	null	hypothetical protein CNAG_04711	1	0	0	1
CNAG_04710	chr10	616795	618780	1985	null	hypothetical protein CNAG_04710	1	0	0	1
CNAG_07855	chr10	626069	641700	15631	null	midasin	2	0	0	2
CNAG_04705	chr10	642670	645481	2811	null	hypothetical protein CNAG_04705	1	0	0	1
CNAG_04703	chr10	650327	653039	2712	null	DNA clamp loader	1	1	0	2
CNAG_04702	chr10	653344	659424	6080	null	hypothetical protein CNAG_04702	0	1	1	2
CNAG_04700	chr10	660049	662457	2408	null	hypothetical protein CNAG_04700	1	0	0	1
CNAG_04699	chr10	662694	664758	2064	null	microfibrillar-associated protein 1	0	0	1	1
CNAG_04698	chr10	664839	665541	702	null	cytoplasmic protein	1	0	0	1
CNAG_04697	chr10	665841	669654	3813	null	zinc finger protein	1	0	0	1
CNAG_04696	chr10	670947	673391	2444	null	DNA clamp loader	0	2	1	3
CNAG_04694	chr10	677619	679538	1919	null	periodic tryptophan protein 1	0	1	0	1
CNAG_04693	chr10	679939	682745	2806	SIN1	hypothetical protein CNAG_04693	1	1	0	2
CNAG_04691	chr10	687001	688455	1454	null	hypothetical protein CNAG_04691	1	0	0	1
CNAG_04689	chr10	690191	691190	999	null	hypothetical protein CNAG_04689	0	1	0	1
CNAG_04687	chr10	694379	696319	1940	null	stearoyl-CoA desaturase (delta-9 desaturase)	0	1	0	1
CNAG_07856	chr10	698782	700795	2013	null	hypothetical protein CNAG_07856	0	1	0	1
CNAG_04682	chr10	706085	706948	863	null	DNA replication complex GINS protein PSF3	1	0	0	1
CNAG_04681	chr10	708117	709999	1882	null	hypothetical protein CNAG_04681	2	0	0	2
CNAG_04680	chr10	710137	710957	820	null	hypothetical protein CNAG_04680	0	1	0	1
CNAG_04675	chr10	721021	721725	704	null	hypothetical protein CNAG_04675	1	0	0	1
CNAG_04674	chr10	722107	724319	2212	ADA3	transcriptional adapter 3	0	1	0	1
CNAG_04669	chr10	731108	732408	1300	null	mitochondrial matrix protein import protein	0	1	0	1
CNAG_04662	chr10	740538	744490	3952	CTF4	chromosome transmission fidelity protein 4	1	0	0	1
CNAG_08000	chr10	744906	753038	8132	null	hypothetical protein CNAG_08000	1	0	0	1
CNAG_04659	chr10	754538	756872	2334	null	pyruvate decarboxylase	0	0	1	1
CNAG_04657	chr10	760039	760866	827	null	short-chain dehydrogenase	0	1	0	1
CNAG_04655	chr10	765366	768051	2685	null	rab family protein	0	1	0	1
CNAG_04654	chr10	768783	770007	1224	null	UNC-50 family protein	0	0	1	1
CNAG_04653	chr10	770424	771411	987	null	hypothetical protein CNAG_04653	0	1	0	1
CNAG_04648	chr10	779205	784211	5006	null	sister chromatid cohesion protein PDS5	1	0	0	1
CNAG_04647	chr10	784611	786265	1654	null	glutathione synthetase	0	1	0	1
CNAG_07857	chr10	787504	790736	3232	null	hypothetical protein CNAG_07857	0	0	1	1
CNAG_04641	chr10	793533	797215	3682	null	general transcription factor 3C polypeptide 3 (transcription factor C subunit 4)	1	0	0	1
CNAG_04640	chr10	797817	801668	3851	ACL1	ATP-citrate synthase subunit 1	0	0	1	1
CNAG_04639	chr10	803664	806762	3098	null	hypothetical protein CNAG_04639	2	0	0	2
CNAG_04633	chr10	824680	825487	807	null	hypothetical protein CNAG_04633	0	3	0	3
CNAG_04632	chr10	827628	829682	2054	null	uracil permease	0	1	1	2
CNAG_04630	chr10	833236	835428	2192	null	hypothetical protein CNAG_04630	0	1	1	2
CNAG_04629	chr10	836568	838736	2168	null	Mob1 family protein	1	0	0	1
CNAG_04626	chr10	843599	847962	4363	null	hypothetical protein CNAG_04626	1	0	0	1
CNAG_04623	chr10	851056	851958	902	null	hypothetical protein CNAG_04623	0	1	0	1
CNAG_04621	chr10	855966	858653	2687	null	glycogen(starch) synthase	0	2	0	2
CNAG_04619	chr10	861556	864446	2890	null	prolyl oligopeptidase	1	0	0	1

CNAG_04617	chr10	869228	872114	2886	null	OPT family small oligopeptide transporter	1	1	0	2
CNAG_04616	chr10	874357	876688	2331	null	hypothetical protein CNAG_04616	0	1	0	1
CNAG_04615	chr10	877803	881405	3602	null	L-aminoadipate-semialdehyde dehydrogenase	1	0	0	1
CNAG_04614	chr10	881594	882971	1377	null	hypothetical protein CNAG_04614	0	1	0	1
CNAG_04608	chr10	900946	902179	1233	null	signal peptidase I	0	1	0	1
CNAG_04604	chr10	908379	910683	2304	null	tryptophan-tRNA ligase	0	1	0	1
CNAG_04600	chr10	919119	923443	4324	null	DNA topoisomerase III	0	0	2	2
CNAG_04599	chr10	923603	926905	3302	null	3-methyl-2-oxobutanoate hydroxymethyltransferase	0	1	0	1
CNAG_04598	chr10	927451	928644	1193	null	hypothetical protein CNAG_04598	2	0	0	2
CNAG_04597	chr10	929456	931386	1930	null	hypothetical protein CNAG_04597	2	1	0	3
CNAG_04592	chr10	941326	941781	455	null	hypothetical protein CNAG_04592	0	1	0	1
CNAG_04589	chr10	946527	947945	1418	null	hypothetical protein CNAG_04589	0	1	1	2
CNAG_04587	chr10	951356	954729	3373	null	hypothetical protein CNAG_04587	0	2	0	2
CNAG_04586	chr10	958575	961194	2619	null	LIM-homeobox protein	0	1	0	1
CNAG_04583	chr10	967957	971353	3396	null	hypothetical protein CNAG_04583	1	0	0	1
CNAG_04582	chr10	973824	975180	1356	null	hypothetical protein CNAG_04582	0	1	0	1
CNAG_04579	chr10	979775	982878	3103	null	alpha-1,2-mannosyltransferase	1	0	0	1
CNAG_07859	chr10	983139	984432	1293	null	peptidyl-prolyl cis-trans isomerase-like 2	1	0	0	1
CNAG_07860	chr10	984623	986814	2191	null	peptidyl-prolyl cis-trans isomerase-like 2	0	0	1	1
CNAG_07861	chr10	986887	987646	759	null	hypothetical protein CNAG_07861	1	0	0	1
CNAG_04561	chr10	1029797	1032095	2298	null	monooxygenase	0	1	0	1
CNAG_07867	chr10	1033823	1037705	3882	null	hypothetical protein CNAG_07867	0	1	1	2
CNAG_04558	chr10	1039133	1039490	357	null	hypothetical protein CNAG_04558	1	0	0	1
CNAG_04552	chr10	1048840	1050864	2024	ITR1A	hypothetical protein CNAG_04552	0	1	0	1
CNAG_08002	chr11	13611	17959	4348	null	hypothetical protein CNAG_08002	2	2	2	6
CNAG_01465	chr11	24643	26831	2188	null	5\' flap endonuclease	1	2	1	4
CNAG_01466	chr11	27405	30080	2675	null	hypothetical protein CNAG_01466	1	0	1	2
CNAG_01467	chr11	30743	32480	1737	null	solute carrier family 35, member F5	0	0	1	1
CNAG_01468	chr11	32899	35725	2826	null	hypothetical protein CNAG_01468	0	4	1	5
CNAG_01470	chr11	40128	41085	957	null	NADH dehydrogenase (ubiquinone) flavoprotein 2	0	1	0	1
CNAG_01472	chr11	43768	47046	3278	null	endoplasmic reticulum protein	0	1	0	1
CNAG_01473	chr11	47870	50614	2744	null	hypothetical protein CNAG_01473	1	2	1	4
CNAG_01474	chr11	51658	54509	2851	null	hypothetical protein CNAG_01474	0	2	1	3
CNAG_01476	chr11	59360	64296	4936	null	hypothetical protein CNAG_01476	2	0	1	3
CNAG_01477	chr11	64925	67166	2241	null	serine palmitoyltransferase	0	1	0	1
CNAG_01482	chr11	77686	79914	2228	null	solute carrier family 25 (mitochondrial phosphate transporter), member 23/24/25/41	1	0	1	2
CNAG_01483	chr11	80306	83058	2752	null	hypothetical protein CNAG_01483	0	1	0	1
CNAG_01485	chr11	85231	87291	2060	null	D-lactate dehydrogenase (cytochrome)	1	4	0	5
CNAG_01487	chr11	91126	92639	1513	null	hypothetical protein CNAG_01487	0	1	0	1
CNAG_07587	chr11	93845	94863	1018	null	hypothetical protein CNAG_07587	1	0	0	1
CNAG_01494	chr11	106393	107292	899	null	hypothetical protein CNAG_01494	1	0	1	2
CNAG_01497	chr11	113096	115332	2236	null	integral membrane protein	1	1	0	2
CNAG_01498	chr11	115811	118127	2316	null	arylsulfatase	0	1	0	1
CNAG_01501	chr11	124970	126051	1081	null	hypothetical protein CNAG_01501	0	1	0	1
CNAG_01504	chr11	131033	132021	988	null	hypothetical protein CNAG_01504	1	0	0	1
CNAG_01506	chr11	135280	136753	1473	null	hypothetical protein CNAG_01506	1	0	0	1
CNAG_01508	chr11	138708	142251	3543	null	hypothetical protein CNAG_01508	2	0	0	2

CNAG_01510	chr11	144203	148498	4295	null	hypothetical protein CNAG_01510	1	0	0	1
CNAG_01513	chr11	156452	158331	1879	null	hypothetical protein CNAG_01513	0	1	1	2
CNAG_07591	chr11	164856	166560	1704	null	mitochondrial metalloendopeptidase OMA1	1	0	0	1
CNAG_01519	chr11	169147	170495	1348	null	endonuclease/exonuclease/phosphatase	0	1	0	1
CNAG_01520	chr11	170775	172918	2143	null	histone-arginine methyltransferase CARM1	0	0	1	1
CNAG_01521	chr11	173170	174622	1452	null	metallo-beta-lactamase	0	1	1	2
CNAG_01522	chr11	174892	176369	1477	null	phenylalanine-tRNA ligase	1	0	0	1
CNAG_01523	chr11	176967	178586	1619	HOG1	CMGC/MAPK/P38 protein kinase	0	1	0	1
CNAG_01526	chr11	183463	185651	2188	null	cleavage and polyadenylation specificity factor subunit 4	1	0	0	1
CNAG_01530	chr11	195573	200242	4669	MGA2	suppressor protein SPT23	1	0	0	1
CNAG_01533	chr11	210678	213902	3224	null	rho GTPase activator	1	0	0	1
CNAG_01535	chr11	216233	218650	2417	null	amino acid permease	2	0	0	2
CNAG_01539	chr11	234981	236904	1923	null	myo-inositol-1-phosphate synthase	0	1	0	1
CNAG_01543	chr11	243958	246865	2907	null	AP-3 complex subunit beta	1	0	0	1
CNAG_01544	chr11	246940	249039	2099	null	histidyl-tRNA synthetase	0	1	0	1
CNAG_01547	chr11	252608	254985	2377	null	WD-repeat protein	1	0	0	1
CNAG_01559	chr11	275860	278967	3107	null	hypothetical protein CNAG_01559	1	0	0	1
CNAG_01562	chr11	289580	290604	1024	BLP4	hypothetical protein CNAG_01562	0	1	0	1
CNAG_01563	chr11	291541	294715	3174	null	histone deacetylase 6/10	0	1	0	1
CNAG_01565	chr11	297397	299983	2586	null	biotin	0	1	1	2
CNAG_01566	chr11	300936	304325	3389	null	gamma-tubulin complex component 3	0	0	1	1
CNAG_01567	chr11	304997	306644	1647	null	hypothetical protein CNAG_01567	0	2	0	2
CNAG_01570	chr11	310746	312316	1570	null	hypothetical protein CNAG_01570	0	1	0	1
CNAG_01571	chr11	313890	315030	1140	null	hypothetical protein CNAG_01571	0	1	0	1
CNAG_01572	chr11	318753	321154	2401	null	tyrosine phosphatase	0	1	0	1
CNAG_01573	chr11	321774	324900	3126	null	hypothetical protein CNAG_01573	1	1	0	2
CNAG_01579	chr11	342670	348752	6082	null	vacuolar membrane-associated protein IML1	1	1	0	2
CNAG_01580	chr11	349363	353974	4611	SCP1	hypothetical protein CNAG_01580	0	0	1	1
CNAG_01583	chr11	355697	356778	1081	null	vacuolar-sorting protein SNF7	0	1	0	1
CNAG_01585	chr11	359061	359587	526	null	hypothetical protein CNAG_01585	0	1	0	1
CNAG_01586	chr11	359880	361055	1175	null	F-type H -transporting ATPase subunit B	0	1	1	2
CNAG_01587	chr11	361270	362814	1544	null	RNA polymerase-associated protein LEO1	0	0	2	2
CNAG_01591	chr11	367146	371325	4179	null	hypothetical protein CNAG_01591	0	1	0	1
CNAG_01592	chr11	371683	372681	998	null	protein-S-isoprenylcysteine O-methyltransferase	0	1	0	1
CNAG_01595	chr11	379142	381236	2094	null	hypothetical protein CNAG_01595	0	1	0	1
CNAG_01599	chr11	385459	386204	745	null	hypothetical protein CNAG_01599	0	1	0	1
CNAG_01600	chr11	386383	387978	1595	null	ribosome assembly protein RRB1	0	0	1	1
CNAG_01601	chr11	388128	389977	1849	null	lipase	1	0	1	2
CNAG_01602	chr11	390498	393052	2554	null	IQ domain-containing calmodulin-binding protein	0	0	1	1
CNAG_01604	chr11	397155	398231	1076	null	TIGR01458 family HAD hydrolase	1	0	0	1
CNAG_01608	chr11	402254	404624	2370	null	nuclear GTP-binding protein	0	0	1	1
CNAG_01610	chr11	408380	412445	4065	null	hypothetical protein CNAG_01610	0	3	0	3
CNAG_01612	chr11	417866	420148	2282	null	CAMK/CAMKL protein kinase	1	0	0	1
CNAG_01616	chr11	432446	436177	3731	null	hypothetical protein CNAG_01616	0	0	2	2
CNAG_01620	chr11	443769	445734	1965	null	hypothetical protein CNAG_01620	0	1	0	1
CNAG_01628	chr11	460321	460844	523	null	small subunit ribosomal protein S20	0	1	0	1
CNAG_01630	chr11	461993	464395	2402	null	polyadenylation factor subunit 2	0	0	1	1

CNAG_01640	chr11	486069	496381	10312	null	hypothetical protein CNAG_01640	0	0	1	1
CNAG_01641	chr11	496810	500718	3908	null	DNA polymerase phi subunit	1	0	0	1
CNAG_01642	chr11	501349	506389	5040	null	DNA mismatch repair protein MSH3	0	1	0	1
CNAG_01651	chr11	521792	524727	2935	null	glicosidase	0	1	0	1
CNAG_01653	chr11	528035	529038	1003	CIG1	cytokine inducing-glycoprotein	0	1	0	1
CNAG_01654	chr11	531228	533781	2553	CAS34	hypothetical protein CNAG_01654	0	0	1	1
CNAG_01656	chr11	537932	539422	1490	null	hypothetical protein CNAG_01656	0	1	0	1
CNAG_01659	chr11	546138	547891	1753	null	hypothetical protein CNAG_01659	0	1	0	1
CNAG_01663	chr11	553278	555208	1930	null	dynactin 4	1	2	0	3
CNAG_01665	chr11	557157	558284	1127	null	tRNA-splicing endonuclease subunit Sen34	0	1	0	1
CNAG_01667	chr11	559536	560976	1440	COQ2	4-hydroxybenzoate polyprenyl transferase	1	0	0	1
CNAG_01676	chr11	578499	580492	1993	null	tRNA (adenine-N(1)-)-methyltransferase non-catalytic subunit TRM6	1	0	0	1
CNAG_01679	chr11	586213	587075	862	null	small subunit ribosomal protein S15	1	0	0	1
CNAG_01682	chr11	596693	598851	2158	null	mitochondrial outer membrane 72K protein	0	0	1	1
CNAG_01692	chr11	626011	627789	1778	UBA3	ubiquitin-activating enzyme E1 C	0	1	0	1
CNAG_01694	chr11	629579	635048	5469	null	hypothetical protein CNAG_01694	0	0	1	1
CNAG_01695	chr11	636089	637637	1548	null	hypothetical protein CNAG_01695	0	0	1	1
CNAG_01696	chr11	638120	639974	1854	null	chaperone DnaJ	1	1	0	2
CNAG_01697	chr11	640317	642052	1735	CSN1201	hypothetical protein CNAG_01697	0	1	1	2
CNAG_01698	chr11	642668	644751	2083	null	hypothetical protein CNAG_01698	3	0	1	4
CNAG_07598	chr11	648479	652230	3751	null	importin-alpha export receptor	1	1	0	2
CNAG_01703	chr11	658204	659849	1645	null	hypothetical protein CNAG_01703	1	0	0	1
CNAG_01704	chr11	660627	664013	3386	null	serine/threonine protein kinase	0	0	1	1
CNAG_01706	chr11	665349	667221	1872	null	hypothetical protein CNAG_01706	0	1	0	1
CNAG_01707	chr11	667543	671984	4441	CRG3	hypothetical protein CNAG_01707	2	2	1	5
CNAG_01708	chr11	672209	675519	3310	null	hypothetical protein CNAG_01708	1	0	0	1
CNAG_01709	chr11	675850	677223	1373	null	translation initiation factor 5	0	2	0	2
CNAG_01710	chr11	678096	679850	1754	null	hypothetical protein CNAG_01710	1	0	0	1
CNAG_01712	chr11	683539	685951	2412	null	hypothetical protein CNAG_01712	1	0	1	2
CNAG_01715	chr11	690345	694031	3686	null	ribosome biogenesis protein BMS1	1	1	1	3
CNAG_01716	chr11	694262	696861	2599	null	hypothetical protein CNAG_01716	0	1	0	1
**CNAG_01717**	**chr11**	**697418**	**698958**	**1540**	**null**	**cell differentiation protein rcd1**	**1**	**11**	**3**	**15**
CNAG_01718	chr11	699124	701492	2368	null	hypothetical protein CNAG_01718	1	0	0	1
CNAG_01719	chr11	702153	703748	1595	null	hypothetical protein CNAG_01719	0	0	1	1
CNAG_01721	chr11	706574	708081	1507	HEM3	porphobilinogen deaminase	0	1	0	1
CNAG_01722	chr11	708600	719766	11166	null	vacuolar protein sorting-associated protein vps13	1	1	0	2
CNAG_01727	chr11	729251	731501	2250	null	hsp71-like protein	0	1	0	1
CNAG_01729	chr11	734192	738425	4233	null	ATP-dependent RNA helicase DHX37/DHR1	2	0	0	2
CNAG_01730	chr11	738664	740350	1686	STE7	STE/STE7/MEK1 protein kinase	0	1	0	1
CNAG_01731	chr11	741852	744219	2367	null	hypothetical protein CNAG_01731	1	0	0	1
CNAG_01732	chr11	744407	746913	2506	null	nuclear protein localization protein 4	1	0	0	1
CNAG_01733	chr11	747258	749115	1857	null	pre-mRNA-processing factor 19	0	1	0	1
CNAG_01737	chr11	755647	756908	1261	ERG25	methylsterol monooxygenase	0	1	0	1
CNAG_01738	chr11	757556	760476	2920	null	hypothetical protein CNAG_01738	0	1	0	1
CNAG_01745	chr11	773985	775416	1431	null	glycerol-3-phosphate dehydrogenase (NAD())	1	0	0	1
CNAG_01749	chr11	786452	787004	552	null	hypothetical protein CNAG_01749	0	1	0	1
CNAG_01750	chr11	787897	790112	2215	null	hsp72-like protein	0	0	1	1

CNAG_01752	chr11	793346	794813	1467	null	solute carrier family 25 (mitochondrial 2-oxodicarboxylate transporter), member 21	0	2	0	2
CNAG_01754	chr11	797201	800374	3173	null	hypothetical protein CNAG_01754	0	1	1	2
CNAG_01755	chr11	800650	803402	2752	null	hypothetical protein CNAG_01755	1	0	0	1
CNAG_01764	chr11	825780	828809	3029	null	cytoplasmic protein	1	0	0	1
CNAG_07604	chr11	833183	835557	2374	FRE5	metalloreductase	2	1	0	3
CNAG_01768	chr11	837322	839472	2150	null	hypothetical protein CNAG_01768	1	0	0	1
CNAG_01770	chr11	844448	846832	2384	null	MRS7 family protein	0	1	1	2
CNAG_01771	chr11	847016	847758	742	null	hypothetical protein CNAG_01771	0	1	0	1
CNAG_07605	chr11	853153	857339	4186	null	hypothetical protein CNAG_07605	1	4	1	6
CNAG_01777	chr11	861050	862529	1479	null	glyoxylate reductase	0	1	0	1
CNAG_01779	chr11	864585	865940	1355	null	hypothetical protein CNAG_01779	1	1	0	2
CNAG_01781	chr11	871014	871784	770	null	hypothetical protein CNAG_01781	1	0	0	1
CNAG_01787	chr11	929453	930708	1255	null	hypothetical protein CNAG_01787	2	6	0	8
CNAG_01788	chr11	934108	935267	1159	null	tRNA(His) guanylyltransferase	0	1	0	1
CNAG_01790	chr11	936586	937665	1079	null	upstream activation factor subunit UAF30	2	0	1	3
CNAG_01792	chr11	940876	943251	2375	null	ATP-dependent DNA helicase PIF1	1	0	0	1
CNAG_01795	chr11	949788	952914	3126	null	hypothetical protein CNAG_01795	1	1	0	2
CNAG_01796	chr11	953590	955607	2017	null	hypothetical protein CNAG_01796	0	1	0	1
CNAG_01799	chr11	960422	961192	770	null	ubiquinol-cytochrome c reductase subunit 8	0	1	0	1
CNAG_01802	chr11	963200	964287	1087	null	Fe-S cluster assembly protein DRE2	0	4	0	4
CNAG_01806	chr11	971095	973503	2408	null	hypothetical protein CNAG_01806	1	0	1	2
CNAG_01807	chr11	974143	978083	3940	null	ATP-dependent helicase	0	1	0	1
CNAG_01809	chr11	980387	982277	1890	null	U4/U6 small nuclear ribonucleoprotein PRP3	0	0	1	1
CNAG_01810	chr11	982425	983076	651	null	DNA-directed RNA polymerase I subunit RPA12	0	1	0	1
CNAG_01811	chr11	983570	984814	1244	null	hypothetical protein CNAG_01811	0	1	1	2
CNAG_01817	chr11	994494	996872	2378	null	signal recognition particle receptor subunit alpha	0	1	0	1
CNAG_01823	chr11	1007280	1008995	1715	null	myo-inositol-1(or 4)-monophosphatase	0	1	0	1
CNAG_01834	chr11	1036816	1037934	1118	null	hypothetical protein CNAG_01834	0	1	0	1
CNAG_01847	chr11	1072993	1075444	2451	null	hypothetical protein CNAG_01847	0	2	0	2
CNAG_01848	chr11	1077440	1082155	4715	null	hypothetical protein CNAG_01848	1	0	0	1
CNAG_01849	chr11	1083198	1084159	961	null	hypothetical protein CNAG_01849	0	1	0	1
CNAG_01858	chr11	1105182	1106955	1773	null	hypothetical protein CNAG_01858	2	0	0	2
CNAG_01859	chr11	1108389	1111106	2717	null	hypothetical protein CNAG_01859	0	1	0	1
CNAG_01864	chr11	1125431	1127249	1818	null	hypothetical protein CNAG_01864	0	1	0	1
CNAG_01866	chr11	1130384	1133806	3422	null	hypothetical protein CNAG_01866	2	1	0	3
CNAG_01867	chr11	1134879	1137425	2546	null	mitochondrial division protein 1	1	0	0	1
CNAG_01869	chr11	1138855	1140962	2107	null	hypothetical protein CNAG_01869	0	0	1	1
CNAG_01871	chr11	1142824	1144496	1672	null	prolactin regulatory element-binding protein	0	1	0	1
CNAG_01874	chr11	1147346	1148132	786	null	glutathione S-transferase	0	1	0	1
CNAG_01875	chr11	1148539	1153810	5271	null	WD-repeat protein	1	0	2	3
CNAG_01880	chr11	1164342	1166073	1731	null	hypothetical protein CNAG_01880	0	0	1	1
CNAG_01882	chr11	1167426	1169655	2229	null	histone-lysine N-methyltransferase, H3 lysine-79 specific	0	1	0	1
CNAG_01883	chr11	1171247	1173092	1845	null	hypothetical protein CNAG_01883	1	0	0	1
CNAG_01885	chr11	1176246	1178873	2627	CID14	DNA polymerase sigma subunit	0	0	1	1
CNAG_07612	chr11	1180451	1181678	1227	null	hypothetical protein CNAG_07612	0	0	1	1
CNAG_01890	chr11	1185400	1188066	2666	MET6	5-methyltetrahydropteroyltriglutamate-homocysteine S-methyltransferase	0	0	1	1
CNAG_01892	chr11	1190592	1191370	778	null	hypothetical protein CNAG_01892	0	1	0	1

CNAG_01905	chr11	1225284	1229083	3799	KSP1	serine/threonine protein kinase	1	1	0	2
CNAG_01926	chr11	1283010	1285419	2409	PEX5	peroxisome targeting signal receptor	1	0	0	1
CNAG_01927	chr11	1285703	1286625	922	null	hypothetical protein CNAG_01927	0	1	0	1
CNAG_01930	chr11	1294486	1297745	3259	null	endopeptidase	0	0	1	1
CNAG_01934	chr11	1303727	1305031	1304	null	hypothetical protein CNAG_01934	0	1	0	1
CNAG_01939	chr11	1320732	1323273	2541	null	hypothetical protein CNAG_01939	1	0	0	1
CNAG_01951	chr11	1349346	1350219	873	null	small subunit ribosomal protein S22-A	0	2	0	2
CNAG_01954	chr11	1357019	1358251	1232	null	aldo-keto reductase	0	0	2	2
CNAG_01955	chr11	1358803	1360073	1270	null	alcohol dehydrogenase	1	0	0	1
CNAG_01973	chr11	1411957	1416172	4215	null	C2H2 zinc finger protein Zas1A	1	0	0	1
CNAG_01982	chr11	1433046	1435425	2379	null	hypothetical protein CNAG_01982	0	0	1	1
CNAG_01985	chr11	1451439	1453423	1984	null	hypothetical protein CNAG_01985	0	0	1	1
CNAG_01986	chr11	1454419	1455489	1070	null	hypothetical protein CNAG_01986	0	1	0	1
CNAG_01988	chr11	1460712	1466749	6037	PHY1	bacteriophytochrome histidine kinase	1	0	1	2
CNAG_01992	chr11	1475351	1478171	2820	null	hypothetical protein CNAG_01992	0	2	0	2
CNAG_01997	chr11	1491668	1493368	1700	null	prephenate dehydrogenase (NADP)	0	1	1	2
CNAG_01999	chr11	1495221	1495929	708	null	hypothetical protein CNAG_01999	0	1	1	2
CNAG_02001	chr11	1498310	1503547	5237	null	inositol-polyphosphate 5-phosphatase	1	3	0	4
CNAG_02002	chr11	1503793	1505216	1423	null	hypothetical protein CNAG_02002	2	2	1	5
CNAG_08013	chr11	1505558	1509778	4220	null	hypothetical protein CNAG_08013	4	1	2	7
CNAG_02004	chr11	1510028	1511243	1215	null	OTU domain-containing protein 6B	0	2	0	2
CNAG_02005	chr11	1511624	1513041	1417	null	hypothetical protein CNAG_02005	1	0	0	1
CNAG_02007	chr11	1515814	1517116	1302	null	adenylate kinase 1	0	1	0	1
CNAG_02008	chr11	1517678	1518933	1255	OVA1	nuclear protein	1	0	0	1
CNAG_02010	chr11	1522326	1524376	2050	null	tartrate transporter	0	0	1	1
CNAG_02013	chr11	1530761	1531868	1107	null	hypothetical protein CNAG_02013	0	2	0	2
CNAG_07618	chr11	1531659	1532438	779	null	hypothetical protein CNAG_07618	1	1	0	2
CNAG_05991	chr12	22624	24569	1945	null	glycosyl hydrolase family 88	0	1	0	1
CNAG_05995	chr12	34421	36635	2214	null	hypothetical protein CNAG_05995	1	0	0	1
CNAG_08014	chr12	37409	37961	552	null	hypothetical protein CNAG_08014	1	0	0	1
CNAG_05996	chr12	38318	41417	3099	null	solute carrier family 32 (vesicular inhibitory amino acid transporter)	0	0	1	1
CNAG_05997	chr12	42493	43341	848	null	hypothetical protein CNAG_05997	0	1	0	1
CNAG_05999	chr12	45350	46323	973	null	peptide alpha-N-acetyltransferase	1	0	0	1
CNAG_06008	chr12	63801	65178	1377	null	asparaginase	0	0	1	1
CNAG_07896	chr12	83434	84135	701	null	hypothetical protein CNAG_07896	1	0	0	1
CNAG_06019	chr12	93824	99518	5694	null	hypothetical protein CNAG_06019	2	1	1	4
CNAG_06020	chr12	100066	101803	1737	null	hypothetical protein CNAG_06020	0	2	0	2
CNAG_06024	chr12	108487	109780	1293	null	peroxin-14	1	0	0	1
CNAG_06027	chr12	113281	115023	1742	null	aryl-alcohol dehydrogenase	0	2	0	2
CNAG_06032	chr12	124300	125357	1057	null	ADP-ribosylation factor-like 2	0	0	1	1
CNAG_06033	chr12	125456	127028	1572	null	pfkB family carbohydrate kinase superfamily	0	1	0	1
CNAG_06034	chr12	131492	133588	2096	null	allantoin permease	0	1	0	1
CNAG_06035	chr12	134325	135845	1520	null	alcohol dehydrogenase	0	7	1	8
CNAG_06036	chr12	136817	139467	2650	null	hypothetical protein CNAG_06036	1	6	1	8
CNAG_06048	chr12	170774	171903	1129	null	hypothetical protein CNAG_06048	0	2	0	2
CNAG_06049	chr12	172239	173254	1015	null	GTP-binding protein ryh1	0	4	1	5
CNAG_06050	chr12	174931	176452	1521	UGE2	UDP-glucose 4-epimerase	0	3	0	3

CNAG_06055	chr12	185700	188304	2604	null	hypothetical protein CNAG_06055	0	1	0	1
CNAG_06056	chr12	189769	190866	1097	null	hypothetical protein CNAG_06056	0	1	0	1
CNAG_07898	chr12	191256	192798	1542	null	hypothetical protein CNAG_07898	0	2	0	2
CNAG_06058	chr12	194388	196624	2236	null	protein DGCR14	2	0	0	2
CNAG_06059	chr12	196765	197842	1077	null	cytoplasmic protein	0	1	0	1
CNAG_06063	chr12	206149	207599	1450	null	cytochrome c oxidase assembly protein subunit 15	1	0	0	1
CNAG_06064	chr12	207779	208820	1041	PTP1	hypothetical protein CNAG_06064	0	1	0	1
CNAG_06071	chr12	227453	228304	851	null	hypothetical protein CNAG_06071	0	1	0	1
CNAG_06072	chr12	228441	229614	1173	null	chorismate mutase	0	1	0	1
CNAG_06075	chr12	235462	236177	715	null	hypothetical protein CNAG_06075	0	1	0	1
CNAG_06082	chr12	256216	257443	1227	null	delayed-type hypersensitivity antigen	0	1	0	1
CNAG_06087	chr12	265454	269164	3710	null	kinetochore protein Spc7/SPC105	0	1	0	1
CNAG_06092	chr12	281878	283266	1388	CLN1	cyclin	1	0	0	1
CNAG_06095	chr12	293679	294525	846	null	large subunit ribosomal protein L13e	0	1	0	1
CNAG_06101	chr12	308438	309584	1146	null	ADP,ATP carrier protein	0	0	1	1
CNAG_06105	chr12	322858	326030	3172	null	hypothetical protein CNAG_06105	1	1	0	2
CNAG_06107	chr12	328508	330105	1597	null	G protein beta subunit-like	0	1	0	1
CNAG_06113	chr12	345527	346757	1230	null	hypothetical protein CNAG_06113	0	1	0	1
CNAG_06114	chr12	347461	348818	1357	null	rab family protein	0	0	2	2
CNAG_06115	chr12	350062	351907	1845	null	acid phosphatase	0	1	0	1
CNAG_08016	chr12	353689	354468	779	null	hypothetical protein CNAG_08016	0	1	0	1
CNAG_06117	chr12	354757	358586	3829	null	hypothetical protein CNAG_06117	0	0	1	1
CNAG_06120	chr12	365657	366909	1252	null	cardiolipin synthase	0	1	0	1
CNAG_06122	chr12	368629	369575	946	null	glycerol-1-phosphatase	0	0	1	1
CNAG_06125	chr12	376054	377754	1700	TEF1	elongation factor 1-alpha	0	0	1	1
CNAG_06129	chr12	383626	384848	1222	null	cytoplasmic protein	1	0	0	1
CNAG_06130	chr12	384890	385925	1035	null	hypothetical protein CNAG_06130	1	1	0	2
CNAG_06131	chr12	386143	390710	4567	null	hypothetical protein CNAG_06131	1	0	1	2
CNAG_06135	chr12	397538	398818	1280	null	hypothetical protein CNAG_06135	0	1	0	1
CNAG_06136	chr12	399076	403603	4527	null	hypothetical protein CNAG_06136	0	4	1	5
CNAG_06138	chr12	406093	406768	675	null	NADH dehydrogenase (ubiquinone) Fe-S protein 6	1	0	0	1
CNAG_08018	chr12	421214	426703	5489	null	hypothetical protein CNAG_08018	1	5	0	6
CNAG_06147	chr12	427010	427888	878	null	hypothetical protein CNAG_06147	0	2	0	2
CNAG_06148	chr12	428078	432954	4876	null	hypothetical protein CNAG_06148	1	1	8	10
CNAG_06149	chr12	433184	435347	2163	null	hypothetical protein CNAG_06149	2	0	1	3
CNAG_06154	chr12	444338	446240	1902	null	cytoplasmic protein	1	0	0	1
CNAG_06156	chr12	450467	454196	3729	null	hypothetical protein CNAG_06156	0	1	0	1
CNAG_06158	chr12	458235	462417	4182	null	hypothetical protein CNAG_06158	2	0	0	2
CNAG_06160	chr12	465158	469736	4578	null	guanyl nucleotide exchange factor Sql2	2	1	0	3
CNAG_06162	chr12	476850	479021	2171	null	hypothetical protein CNAG_06162	0	1	1	2
CNAG_06163	chr12	480281	482590	2309	null	hypothetical protein CNAG_06163	0	3	0	3
CNAG_06164	chr12	482936	484843	1907	null	hypothetical protein CNAG_06164	0	2	1	3
CNAG_06170	chr12	506561	507628	1067	null	S-formylglutathione hydrolase	2	0	0	2
CNAG_06172	chr12	508999	511436	2437	null	transketolase	0	1	0	1
CNAG_06173	chr12	512228	513505	1277	null	hypothetical protein CNAG_06173	1	1	0	2
CNAG_06174	chr12	513784	520391	6607	null	PEK/GCN2 protein kinase	2	0	1	3
**CNAG_06175**	**chr12**	**520646**	**524049**	**3403**	**null**	**26S proteasome regulatory subunit N2**	**0**	**10**	**1**	**11**

CNAG_07908	chr12	524967	527499	2532	null	aconitate hydratase, mitochondrial	1	1	0	2
CNAG_07909	chr12	527878	529784	1906	null	meiotic recombinase Dmc1	0	2	0	2
CNAG_06178	chr12	530239	535005	4766	null	hypothetical protein CNAG_06178	0	1	0	1
CNAG_07910	chr12	545342	545815	473	null	hypothetical protein CNAG_07910	2	2	0	4
CNAG_06183	chr12	546566	549439	2873	null	origin recognition complex subunit 4	0	1	2	3
CNAG_06184	chr12	549827	551970	2143	null	hypothetical protein CNAG_06184	0	1	0	1
CNAG_06185	chr12	552437	553559	1122	null	hypothetical protein CNAG_06185	0	1	1	2
CNAG_06188	chr12	560605	564086	3481	null	hypothetical protein CNAG_06188	0	0	1	1
CNAG_06189	chr12	564810	565394	584	null	hypothetical protein CNAG_06189	1	0	0	1
CNAG_06192	chr12	570825	576013	5188	null	hypothetical protein CNAG_06192	1	0	1	2
CNAG_06194	chr12	584297	585776	1479	null	hypothetical protein CNAG_06194	1	2	0	3
CNAG_06195	chr12	585940	587277	1337	null	hypothetical protein CNAG_06195	0	2	1	3
CNAG_06197	chr12	589118	592431	3313	null	hypothetical protein CNAG_06197	1	0	0	1
CNAG_06199	chr12	594613	599808	5195	null	nuclear DNA helicase II	2	0	1	3
CNAG_06200	chr12	600122	601651	1529	null	hypothetical protein CNAG_06200	0	4	0	4
CNAG_06202	chr12	605702	607346	1644	null	hypothetical protein CNAG_06202	1	2	0	3
**CNAG_06203**	**chr12**	**614081**	**616896**	**2815**	**MAT2**	**hypothetical protein CNAG_06203**	**1**	**0**	**0**	**1**
CNAG_06205	chr12	621585	623141	1556	BLP3	hypothetical protein CNAG_06205	2	2	0	4
CNAG_06206	chr12	623871	625945	2074	null	ATP-dependent RNA helicase DBP9	0	0	1	1
CNAG_06209	chr12	634288	636211	1923	null	hypothetical protein CNAG_06209	0	1	0	1
CNAG_07912	chr12	636724	637155	431	null	hypothetical protein CNAG_07912	0	1	0	1
CNAG_06221	chr12	664636	666603	1967	null	diphthamide biosynthesis protein 2	0	1	0	1
CNAG_06228	chr12	683316	686592	3276	null	MIF4G/MA4 domain-containing protein	0	2	0	2
CNAG_06229	chr12	686899	687859	960	null	hypothetical protein CNAG_06229	0	2	0	2
CNAG_06230	chr12	688602	690621	2019	null	hypothetical protein CNAG_06230	1	10	0	11
CNAG_06232	chr12	693665	694979	1314	null	transcription factor C subunit 7	0	4	0	4
CNAG_06233	chr12	696087	696959	872	null	hypothetical protein CNAG_06233	0	1	0	1
CNAG_06234	chr12	697249	697698	449	null	hypothetical protein CNAG_06234	2	0	0	2
CNAG_06235	chr12	698588	700084	1496	null	hypothetical protein CNAG_06235	0	1	0	1
CNAG_06236	chr12	700610	702635	2025	null	RNA exonuclease NGL2	0	1	2	3
CNAG_06237	chr12	702927	704044	1117	null	YeeE/YedE family protein	1	0	2	3
CNAG_06238	chr12	704184	705405	1221	null	glutathione S-transferase	1	2	0	3
CNAG_06239	chr12	706370	708263	1893	null	hypothetical protein CNAG_06239	6	0	2	8
CNAG_06241	chr12	710724	713329	2605	CFO1	acidic laccase	0	3	0	3
CNAG_06243	chr12	717336	718953	1617	null	hypothetical protein CNAG_06243	0	0	1	1
CNAG_06244	chr12	719455	721996	2541	null	hypothetical protein CNAG_06244	2	0	0	2
CNAG_06246	chr12	723621	724740	1119	null	hypothetical protein CNAG_06246	0	1	0	1
CNAG_06247	chr12	726406	729345	2939	null	hypothetical protein CNAG_06247	1	0	0	1
CNAG_06248	chr12	729800	734579	4779	null	protein TIF31	0	8	1	9
CNAG_06249	chr12	737769	739439	1670	null	taurine catabolism dioxygenase TauD	1	0	0	1
CNAG_06250	chr12	740300	742398	2098	null	hypothetical protein CNAG_06250	0	1	0	1
CNAG_06252	chr12	746822	749259	2437	null	hypothetical protein CNAG_06252	1	0	0	1
CNAG_06253	chr12	750942	753314	2372	null	sugar transporter	0	1	0	1
CNAG_06259	chr13	12544	14859	2315	null	MFS transporter, SP family, general alpha glucoside:H symporter	1	0	0	1
CNAG_06260	chr13	17420	19723	2303	null	alpha-glucosidase	1	0	0	1
CNAG_07922	chr13	26005	28148	2143	null	transcription factor	0	1	0	1
CNAG_06271	chr13	51440	52740	1300	null	hypothetical protein CNAG_06271	1	1	0	2

CNAG_06281	chr13	84222	85181	959	null	hypothetical protein CNAG_06281	1	0	0	1
CNAG_06282	chr13	85832	90001	4169	null	WD-repeat protein 48	0	0	1	1
CNAG_06296	chr13	121177	121763	586	null	hypothetical protein CNAG_06296	0	1	0	1
CNAG_06299	chr13	126116	128221	2105	null	hypothetical protein CNAG_06299	1	0	2	3
CNAG_06301	chr13	133897	136742	2845	SCH9	AGC/Akt protein kinase	0	2	0	2
CNAG_07931	chr13	144516	145156	640	null	hypothetical protein CNAG_07931	1	0	0	1
CNAG_06321	chr13	183500	184622	1122	null	hypothetical protein CNAG_06321	0	3	0	3
CNAG_06325	chr13	193650	196881	3231	null	GTPase activator	0	1	0	1
CNAG_06329	chr13	205993	208048	2055	null	high-affinity nicotinic acid transporter	0	0	1	1
CNAG_06332	chr13	213774	217238	3464	null	hypothetical protein CNAG_06332	1	0	0	1
CNAG_06333	chr13	217573	219499	1926	null	replication factor C subunit 3/5	0	1	0	1
CNAG_06337	chr13	229945	231437	1492	null	hypothetical protein CNAG_06337	0	1	0	1
CNAG_06338	chr13	234722	239916	5194	null	ABC transporter PMR5	1	0	1	2
CNAG_06339	chr13	241189	243911	2722	null	hypothetical protein CNAG_06339	1	1	0	2
CNAG_06340	chr13	244718	246098	1380	null	pre-rRNA-processing protein TSR4	0	0	1	1
CNAG_06341	chr13	246366	247375	1009	null	hypothetical protein CNAG_06341	1	0	0	1
CNAG_06344	chr13	250615	251145	530	null	hypothetical protein CNAG_06344	0	1	0	1
CNAG_06347	chr13	258885	259378	493	BLP2	hypothetical protein CNAG_06347	0	1	0	1
CNAG_06348	chr13	261276	266510	5234	PDR5-3	ABC transporter PMR5	0	2	0	2
CNAG_06349	chr13	267898	269044	1146	null	hypothetical protein CNAG_06349	0	1	0	1
CNAG_06350	chr13	269171	270980	1809	null	hypothetical protein CNAG_06350	1	0	0	1
CNAG_06352	chr13	272335	275773	3438	null	hypothetical protein CNAG_06352	1	0	0	1
CNAG_06354	chr13	278701	280533	1832	null	hypothetical protein CNAG_06354	1	1	0	2
CNAG_06361	chr13	291146	292961	1815	null	26S proteasome regulatory subunit N6	0	1	0	1
CNAG_06362	chr13	293389	301614	8225	null	hypothetical protein CNAG_06362	1	0	0	1
CNAG_06365	chr13	307832	308914	1082	null	derlin-2/3	0	2	0	2
CNAG_06368	chr13	313556	314828	1272	null	hypothetical protein CNAG_06368	0	1	0	1
CNAG_06379	chr13	335174	338668	3494	null	N-acetyltransferase 10	0	1	0	1
CNAG_06381	chr13	340858	343701	2843	null	hypothetical protein CNAG_06381	2	1	0	3
CNAG_06385	chr13	354340	355024	684	null	GAF domain-containing protein	0	1	0	1
CNAG_06388	chr13	360543	360985	442	null	hypothetical protein CNAG_06388	0	1	0	1
CNAG_06389	chr13	362681	363843	1162	null	hypothetical protein CNAG_06389	0	2	1	3
CNAG_06390	chr13	364292	366348	2056	null	hypothetical protein CNAG_06390	0	0	1	1
CNAG_07936	chr13	375991	384869	8878	null	hypothetical protein CNAG_07936	0	0	1	1
CNAG_06400	chr13	387802	391181	3379	PMA1	plasma-membrane proton-efflux P-type ATPase	0	1	0	1
CNAG_06402	chr13	398850	399326	476	null	26S proteasome complex subunit DSS1	0	1	0	1
CNAG_06404	chr13	402860	403241	381	null	hypothetical protein CNAG_06404	0	1	0	1
CNAG_06405	chr13	405564	407334	1770	null	hypothetical protein CNAG_06405	1	0	0	1
CNAG_06410	chr13	413082	414016	934	null	ATP-dependent Clp endopeptidase, proteolytic subunit ClpP	0	0	1	1
CNAG_06411	chr13	414440	416491	2051	null	mutanase	1	1	0	2
CNAG_06412	chr13	416755	417850	1095	null	rRNA-processing protein EFG1	1	1	0	2
CNAG_06413	chr13	419158	419933	775	null	hypothetical protein CNAG_06413	0	1	0	1
CNAG_06416	chr13	425147	428601	3454	DAP2	dipeptidyl aminopeptidase	0	0	1	1
CNAG_06427	chr13	447112	449090	1978	null	hypothetical protein CNAG_06427	0	0	1	1
CNAG_07937	chr13	452266	455538	3272	CAS1	O-acetyltransferase	1	0	0	1
CNAG_07938	chr13	455840	463336	7496	null	hypothetical protein CNAG_07938	0	0	1	1
CNAG_06431	chr13	463486	465222	1736	null	acyl-CoA oxidase	0	1	0	1

CNAG_06432	chr13	465694	467354	1660	null	acetate kinase	0	0	1	1
CNAG_06433	chr13	468088	470394	2306	null	AMP-binding protein	0	0	2	2
CNAG_07939	chr13	472094	472410	316	null	hypothetical protein CNAG_07939	0	3	0	3
CNAG_06435	chr13	477119	480564	3445	null	hypothetical protein CNAG_06435	0	2	0	2
CNAG_06437	chr13	481411	482223	812	null	hypothetical protein CNAG_06437	0	1	0	1
CNAG_06443	chr13	499184	501752	2568	SSA1	glucose-regulated protein	0	0	1	1
CNAG_06445	chr13	503610	505125	1515	null	CMGC/CDK/CDK7 protein kinase	0	0	1	1
CNAG_06447	chr13	508573	509459	886	null	large subunit ribosomal protein L22	0	1	0	1
CNAG_06448	chr13	509976	511615	1639	null	cystathionine gamma-lyase	1	0	1	2
CNAG_06449	chr13	512372	514650	2278	null	hypothetical protein CNAG_06449	2	2	0	4
CNAG_06451	chr13	519348	520000	652	null	hypothetical protein CNAG_06451	0	1	0	1
CNAG_06452	chr13	520342	521995	1653	null	integral membrane protein	0	1	0	1
CNAG_06454	chr13	524184	526421	2237	null	Atypical/RIO/RIO1 protein kinase	1	0	0	1
CNAG_07941	chr13	531153	533674	2521	null	hypothetical protein CNAG_07941	0	1	0	1
CNAG_06460	chr13	538552	540408	1856	null	hypothetical protein CNAG_06460	0	0	1	1
CNAG_07942	chr13	541373	545785	4412	null	cell division control protein 25	1	1	1	3
CNAG_06465	chr13	549224	550954	1730	null	transcription regulator	0	0	1	1
CNAG_06466	chr13	551123	552840	1717	null	jumonji domain containing 5	0	1	0	1
CNAG_06472	chr13	565251	568581	3330	null	nucleolar protein 4	1	0	0	1
CNAG_06474	chr13	573397	575577	2180	null	RNA-binding protein Musashi	0	0	1	1
CNAG_06475	chr13	576327	579391	3064	null	hypothetical protein CNAG_06475	0	3	0	3
CNAG_06483	chr13	634761	637339	2578	null	hypothetical protein CNAG_06483	0	4	0	4
CNAG_06484	chr13	637421	638929	1508	null	hypothetical protein CNAG_06484	0	1	0	1
CNAG_06486	chr13	642466	644773	2307	null	hypothetical protein CNAG_06486	1	3	0	4
CNAG_06490	chr13	653089	657988	4899	null	CAMK/CAMKL protein kinase	1	2	1	4
CNAG_06492	chr13	660061	661380	1319	null	cytoplasmic protein	0	1	0	1
CNAG_06505	chr13	694763	696493	1730	null	hypothetical protein CNAG_06505	0	0	1	1
CNAG_06511	chr13	711860	713695	1835	null	hypothetical protein CNAG_06511	0	1	0	1
CNAG_07944	chr13	716491	721044	4553	null	urea carboxylase	2	1	0	3
CNAG_06518	chr13	732275	732934	659	null	hypothetical protein CNAG_06518	0	3	0	3
CNAG_05333	chr14	2566	5152	2586	null	hypothetical protein CNAG_05333	1	1	0	2
CNAG_05334	chr14	6952	8084	1132	null	hypothetical protein CNAG_05334	1	2	0	3
CNAG_05336	chr14	9303	11536	2233	null	glucose transporter	0	3	1	4
CNAG_05338	chr14	15460	16141	681	null	hypothetical protein CNAG_05338	0	1	0	1
CNAG_05341	chr14	23769	25696	1927	null	hypothetical protein CNAG_05341	0	3	0	3
CNAG_05342	chr14	26659	27699	1040	null	hypothetical protein CNAG_05342	1	0	1	2
CNAG_05343	chr14	29424	34237	4813	null	hypothetical protein CNAG_05343	1	0	1	2
CNAG_05344	chr14	34892	38774	3882	null	hemolysin	0	1	0	1
CNAG_05345	chr14	39608	41860	2252	null	amino acid transporter	0	1	0	1
CNAG_05346	chr14	42476	43972	1496	null	sarcosine oxidase	0	0	1	1
CNAG_07869	chr14	46321	48236	1915	null	hypothetical protein CNAG_07869	0	0	1	1
CNAG_07033	chr14	50267	51525	1258	null	hypothetical protein CNAG_07033	0	0	1	1
CNAG_05349	chr14	54923	59328	4405	null	hypothetical protein CNAG_05349	0	1	0	1
CNAG_05352	chr14	66641	68828	2187	null	acetyl-CoA transporter	1	0	0	1
CNAG_05353	chr14	68991	73809	4818	null	nuclear pore complex protein Nup133	1	0	0	1
CNAG_05360	chr14	89594	92207	2613	null	ATP-dependent RNA helicase MAK5	0	0	2	2
CNAG_05369	chr14	114242	117097	2855	null	beta-glucosidase	0	1	0	1

CNAG_05371	chr14	119005	121692	2687	null	hypothetical protein CNAG_05371	1	0	0	1
CNAG_05372	chr14	122360	123625	1265	BLP5	B2-aldehyde-forming enzyme	0	2	0	2
CNAG_05373	chr14	124230	125858	1628	null	elongator complex protein 4	1	0	0	1
CNAG_05374	chr14	126153	129432	3279	null	chromosome transmission fidelity protein 18	0	0	1	1
**CNAG_05375**	**chr14**	**133828**	**135128**	**1300**	**null**	**hypothetical protein CNAG_05375**	**1**	**10**	**2**	**13**
CNAG_05377	chr14	138145	140359	2214	ITR3	MFS transporter, SP family, solute carrier family 2 (myo-inositol transporter), member 13	0	2	0	2
CNAG_05378	chr14	141431	142546	1115	null	3-hydroxyacyl-CoA dehydrogenase	0	1	0	1
CNAG_05379	chr14	143093	144297	1204	null	regucalcin	0	2	0	2
CNAG_05380	chr14	144801	148667	3866	null	hypothetical protein CNAG_05380	0	0	1	1
CNAG_05381	chr14	149570	151758	2188	ITR3C	MFS transporter, SP family, solute carrier family 2 (myo-inositol transporter), member 13	1	4	1	6
CNAG_07874	chr14	156158	158302	2144	null	sugar transporter	0	1	0	1
CNAG_05383	chr14	159780	161240	1460	null	hypothetical protein CNAG_05383	0	1	1	2
CNAG_05387	chr14	172703	174850	2147	null	galactose transporter	0	3	0	3
CNAG_05390	chr14	182619	183721	1102	null	hypothetical protein CNAG_05390	0	0	1	1
CNAG_05393	chr14	192619	195143	2524	null	ATPase	0	0	1	1
CNAG_05395	chr14	198991	202766	3775	VAM6	hypothetical protein CNAG_05395	2	1	2	5
CNAG_07875	chr14	202785	203202	417	null	hypothetical protein CNAG_07875	0	1	0	1
CNAG_05396	chr14	203661	204639	978	null	LRP16 family protein	1	0	0	1
CNAG_05401	chr14	212012	214552	2540	null	hypothetical protein CNAG_05401	0	0	1	1
CNAG_05416	chr14	250965	252927	1962	null	pre-mRNA-processing protein 45	0	1	0	1
CNAG_08023	chr14	253205	257719	4514	null	hypothetical protein CNAG_08023	0	0	1	1
CNAG_05419	chr14	258430	260581	2151	null	hypothetical protein CNAG_05419	1	0	1	2
CNAG_05420	chr14	261459	263499	2040	null	RNA polymerase II transcription factor	0	2	0	2
CNAG_05428	chr14	288275	291148	2873	TAF5	transcription initiation factor TFIID subunit 5	0	0	3	3
CNAG_05430	chr14	293442	294756	1314	null	hypothetical protein CNAG_05430	0	0	1	1
CNAG_05431	chr14	295214	298064	2850	RIM101	pH-response transcription factor pacC/RIM101	0	2	0	2
CNAG_05435	chr14	305603	307291	1688	null	hypothetical protein CNAG_05435	1	0	0	1
CNAG_05439	chr14	312138	313974	1836	null	CAMK/CAMK1/CAMK1-CMK protein kinase	0	1	0	1
CNAG_05440	chr14	315895	316752	857	null	pyridoxamine 5\'-phosphate oxidase	0	1	0	1
CNAG_05441	chr14	317429	319537	2108	null	lipid particle protein	0	2	1	3
CNAG_05443	chr14	321800	324154	2354	null	hypothetical protein CNAG_05443	0	0	1	1
CNAG_05445	chr14	329263	331675	2412	null	smooth muscle cell associated protein-1 isoform 2	1	1	0	2
CNAG_07876	chr14	339489	340119	630	null	hypothetical protein CNAG_07876	0	1	0	1
CNAG_05451	chr14	344936	345685	749	null	hypothetical protein CNAG_05451	0	3	0	3
**CNAG_05452**	**chr14**	**346448**	**349039**	**2591**	**null**	**hypothetical protein CNAG_05452**	**1**	**10**	**1**	**12**
CNAG_05453	chr14	350156	350473	317	null	hypothetical protein CNAG_05453	0	4	0	4
CNAG_05454	chr14	351626	353014	1388	null	hypothetical protein CNAG_05454	0	1	0	1
CNAG_05455	chr14	355010	355872	862	null	translation initiation factor eIF-1A	0	1	0	1
CNAG_05457	chr14	358117	362715	4598	null	pentatricopeptide repeat protein	1	0	0	1
CNAG_05458	chr14	363830	365248	1418	null	endo-1,3(4)-beta-glucanase	0	1	0	1
CNAG_05460	chr14	369675	370899	1224	null	2-dehydropantoate 2-reductase	0	1	0	1
CNAG_05463	chr14	376143	378108	1965	null	hypothetical protein CNAG_05463	0	1	1	2
CNAG_08025	chr14	380847	381479	632	null	hypothetical protein CNAG_08025	1	0	0	1
CNAG_05466	chr14	381746	383395	1649	null	hypothetical protein CNAG_05466	1	0	1	2
CNAG_05468	chr14	385391	387151	1760	null	AP endonuclease 1	1	0	0	1
CNAG_05469	chr14	387487	390094	2607	HRD1	E3 ubiquitin-protein ligase synoviolin	0	2	0	2
CNAG_05471	chr14	396698	400835	4137	null	alpha-glucosidase	0	1	0	1

CNAG_05473	chr14	407647	408596	949	null	hypothetical protein CNAG_05473	0	0	1	1
CNAG_07879	chr14	409483	411079	1596	null	TRIAD3, E3 ubiquitin-protein ligase RNF216	0	1	0	1
CNAG_05476	chr14	414099	414767	668	null	hypothetical protein CNAG_05476	0	3	0	3
CNAG_05477	chr14	415120	417522	2402	null	protein disulfide-isomerase	0	5	0	5
CNAG_05478	chr14	417866	418976	1110	null	charged multivesicular body protein 2A	1	0	0	1
CNAG_05479	chr14	420406	424320	3914	null	hypothetical protein CNAG_05479	0	2	0	2
CNAG_05480	chr14	424641	427248	2607	HXK1	hexokinase	0	1	4	5
CNAG_07881	chr14	430064	430500	436	null	hypothetical protein CNAG_07881	0	1	1	2
CNAG_05482	chr14	430612	431888	1276	null	hypothetical protein CNAG_05482	0	1	0	1
CNAG_05484	chr14	433124	435547	2423	null	CAMKK/CAMKK-META protein kinase	1	0	0	1
CNAG_05486	chr14	437892	441787	3895	null	hypothetical protein CNAG_05486	0	2	0	2
CNAG_08026	chr14	472367	472736	369	null	hypothetical protein CNAG_08026	0	1	0	1
CNAG_05497	chr14	481110	483283	2173	null	dihydroxy-acid dehydratase	0	1	0	1
CNAG_05499	chr14	485991	487179	1188	null	SHO1 osmosensor	1	0	0	1
CNAG_05500	chr14	487625	489085	1460	null	hypothetical protein CNAG_05500	1	0	0	1
CNAG_05501	chr14	489206	490098	892	null	hypothetical protein CNAG_05501	0	1	0	1
CNAG_05502	chr14	491447	493566	2119	null	hypothetical protein CNAG_05502	1	1	0	2
CNAG_05503	chr14	493855	495881	2026	null	LIM domain-containing protein	0	1	0	1
CNAG_07883	chr14	498870	500936	2066	null	hypothetical protein CNAG_07883	0	3	0	3
CNAG_07884	chr14	501182	502485	1303	null	hypothetical protein CNAG_07884	0	1	0	1
CNAG_05508	chr14	503394	506151	2757	null	hypothetical protein CNAG_05508	0	2	0	2
CNAG_05510	chr14	507758	508627	869	null	hypothetical protein CNAG_05510	0	1	0	1
CNAG_05512	chr14	512789	515087	2298	null	hypothetical protein CNAG_05512	1	0	0	1
CNAG_05513	chr14	515548	516461	913	null	septum-promoting GTP-binding protein 1	0	3	0	3
CNAG_05517	chr14	525447	528333	2886	null	solute carrier family 45, member 1/2/4	0	1	0	1
CNAG_05518	chr14	528483	533310	4827	null	exocyst complex component 4, partial	1	1	0	2
CNAG_05519	chr14	534120	537106	2986	null	hypothetical protein CNAG_05519	0	1	0	1
CNAG_05521	chr14	541591	542770	1179	null	aldose reductase	0	1	0	1
CNAG_05525	chr14	553983	554682	699	null	small subunit ribosomal protein S26	1	0	0	1
CNAG_05527	chr14	556744	563672	6928	null	senataxin	0	0	1	1
CNAG_05529	chr14	567651	569731	2080	null	hypothetical protein CNAG_05529	0	1	0	1
CNAG_05530	chr14	569925	570587	662	null	hypothetical protein CNAG_05530	0	2	0	2
CNAG_05534	chr14	578651	579995	1344	PLR1	pyridoxal reductase	0	1	0	1
CNAG_05549	chr14	613857	617209	3352	null	CMGC/CDK protein kinase	1	0	0	1
CNAG_08027	chr14	647870	649385	1515	null	hypothetical protein CNAG_08027	0	1	0	1
CNAG_05565	chr14	657005	659158	2153	null	hypothetical protein CNAG_05565	1	0	0	1
CNAG_05568	chr14	662178	663454	1276	null	septum formation protein Maf	0	2	0	2
CNAG_05571	chr14	665806	674266	8460	null	U3 small nucleolar RNA-associated protein 20	1	0	0	1
CNAG_05580	chr14	687177	688167	990	null	hypothetical protein CNAG_05580	0	1	0	1
CNAG_05581	chr14	688759	693231	4472	CHS3	chitin synthase	1	3	0	4
CNAG_05583	chr14	695945	697547	1602	GCS1	ceramide glucosyltransferase	0	0	0	0
CNAG_05585	chr14	699600	700641	1041	null	arf/Sar family protein	0	0	1	1
CNAG_05587	chr14	705694	707141	1447	null	hypothetical protein CNAG_05587	1	0	0	1
CNAG_05588	chr14	707701	712813	5112	null	hypothetical protein CNAG_05588	0	1	0	1
CNAG_07886	chr14	712981	714870	1889	null	hypothetical protein CNAG_07886	0	1	0	1
CNAG_05590	chr14	716557	722222	5665	TCO2	hypothetical protein CNAG_05590	0	4	0	4
CNAG_05591	chr14	723384	724981	1597	null	hypothetical protein CNAG_05591	1	1	0	2

CNAG_05592	chr14	726474	729759	3285	ECA1	potassium/sodium efflux P-type ATPase, fungal-type	1	1	0	2
CNAG_05601	chr14	745534	748512	2978	null	calpain-like protease palB/RIM13	1	0	1	2
CNAG_05604	chr14	753134	753855	721	null	hypothetical protein CNAG_05604	0	1	0	1
CNAG_05610	chr14	769995	770768	773	null	hypothetical protein CNAG_05610	0	1	0	1
CNAG_05611	chr14	770962	773010	2048	null	cysteine synthase A	0	1	0	1
CNAG_07889	chr14	773343	773855	512	null	hypothetical protein CNAG_07889	0	1	0	1
CNAG_05613	chr14	776131	782461	6330	null	hypothetical protein CNAG_05613	1	0	0	1
CNAG_05614	chr14	782633	783791	1158	null	YbgI/family dinuclear metal center protein	0	4	0	4
CNAG_05616	chr14	786283	790175	3892	null	ABC transporter	2	0	0	2
CNAG_05617	chr14	790517	793630	3113	null	phosphatidylinositol glycan, class O	0	1	0	1
CNAG_05619	chr14	796985	798344	1359	null	TPR repeat-containing protein	0	1	0	1
CNAG_05622	chr14	804417	808920	4503	RPH1	specific transcriptional repressor	0	2	0	2
CNAG_05623	chr14	809438	810920	1482	null	chorismate synthase	0	1	0	1
CNAG_05626	chr14	814886	815974	1088	null	hypothetical protein CNAG_05626	0	1	0	1
CNAG_05627	chr14	816234	818728	2494	null	ATP-dependent rRNA helicase SPB4	0	0	0	0
CNAG_05628	chr14	818938	821957	3019	null	hypothetical protein CNAG_05628	0	1	0	1
CNAG_05630	chr14	825400	826966	1566	null	hypothetical protein CNAG_05630	0	1	0	1
CNAG_05631	chr14	827845	829649	1804	null	NADH-ubiquinone oxidoreductase 49 kDa subunit, mitochondrial	0	2	0	2
CNAG_05635	chr14	836988	837623	635	null	large subunit ribosomal protein L19	0	1	0	1
CNAG_05655	chr14	889044	889766	722	null	EF-hand calcium-binding protein	0	1	0	1
CNAG_05657	chr14	894288	895517	1229	null	cytoplasmic protein	0	2	0	2
CNAG_05664	chr14	916619	918258	1639	null	branched-chain-amino-acid transaminase	0	1	0	1
CNAG_07459	chr2	1207	1574	367	null	hypothetical protein CNAG_07459	0	2	0	2
CNAG_06799	chr2	6714	7972	1258	HEM12	uroporphyrinogen decarboxylase	0	1	0	1
CNAG_06798	chr2	8136	10378	2242	null	U3 small nucleolar RNA-associated protein 18	2	0	0	2
CNAG_06797	chr2	10747	17219	6472	null	lysine-specific demethylase 3	0	4	0	4
CNAG_06795	chr2	19646	20697	1051	null	hypothetical protein CNAG_06795	0	0	2	2
CNAG_06794	chr2	21823	22811	988	null	hypothetical protein CNAG_06794	0	2	0	2
CNAG_06793	chr2	23204	27195	3991	null	exonuclease 1	1	3	0	4
CNAG_06792	chr2	27412	28419	1007	null	hypothetical protein CNAG_06792	0	6	1	7
CNAG_06791	chr2	29610	31026	1416	null	hypothetical protein CNAG_06791	1	5	1	7
CNAG_06790	chr2	31607	36801	5194	null	hypothetical protein CNAG_06790	1	0	1	2
CNAG_06787	chr2	40204	42505	2301	null	metacaspase	1	0	1	2
CNAG_06786	chr2	43655	44781	1126	null	hypothetical protein CNAG_06786	0	1	0	1
CNAG_06785	chr2	45235	46548	1313	null	kynurenine 3-monooxygenase	0	1	0	1
CNAG_06782	chr2	51787	53552	1765	MNN2	hypothetical protein CNAG_06782	0	0	1	1
CNAG_06781	chr2	53667	54625	958	null	hypothetical protein CNAG_06781	0	3	0	3
CNAG_06778	chr2	62105	63482	1377	null	serine-threonine kinase receptor-associated protein	0	0	1	1
CNAG_06773	chr2	74190	77097	2907	SEC24	protein transporter SEC24	0	2	0	2
CNAG_06772	chr2	77282	78750	1468	null	nucleoporin SEH1	0	1	0	1
CNAG_06767	chr2	89661	90954	1293	null	F-type H -transporting ATPase subunit delta	0	1	0	1
CNAG_06765	chr2	98930	103013	4083	LMP1	hypothetical protein CNAG_06765	0	0	1	1
CNAG_06763	chr2	106087	107785	1698	null	serine-tRNA ligase	1	1	0	2
CNAG_06752	chr2	140526	142531	2005	null	hypothetical protein CNAG_06752	0	1	0	1
CNAG_06747	chr2	157101	157661	560	null	histone H2A	0	1	0	1
CNAG_06737	chr2	180985	183759	2774	null	vacuolar protein sorting-associated protein	0	1	0	1
CNAG_07463	chr2	185063	192509	7446	null	separase	0	4	1	5

CNAG_06734	chr2	192761	193261	500	null	DNA-directed RNA polymerase I, II, and III subunit rpabc5	0	1	0	1
CNAG_06731	chr2	202071	204779	2708	null	hypothetical protein CNAG_06731	1	0	1	2
CNAG_06730	chr2	206338	207745	1407	GSK3	CMGC/GSK protein kinase	0	1	0	1
CNAG_06728	chr2	209220	212783	3563	null	kinesin	2	0	0	2
CNAG_06727	chr2	213693	216968	3275	null	hypothetical protein CNAG_06727	0	1	0	1
CNAG_07963	chr2	218399	218865	466	null	hypothetical protein CNAG_07963	0	1	0	1
CNAG_06725	chr2	222631	224542	1911	null	DNA polymerase delta subunit 2	0	5	0	5
CNAG_06723	chr2	227368	228386	1018	null	succinate dehydrogenase (ubiquinone) membrane anchor subunit	0	1	0	1
CNAG_06722	chr2	229364	230978	1614	null	hypothetical protein CNAG_06722	1	0	2	3
CNAG_07464	chr2	231155	233770	2615	MBS1	transcription factor	1	4	2	7
CNAG_07465	chr2	234195	235073	878	null	hypothetical protein CNAG_07465	0	1	0	1
CNAG_06719	chr2	235880	239543	3663	null	hypothetical protein CNAG_06719	4	0	0	4
CNAG_06717	chr2	241165	242396	1231	null	acyl-CoA-dependent ceramide synthase	0	1	0	1
CNAG_06716	chr2	242827	243841	1014	null	hypothetical protein CNAG_06716	0	1	0	1
CNAG_06715	chr2	243971	244790	819	null	hypothetical protein CNAG_06715	1	0	0	1
CNAG_06712	chr2	249447	250053	606	null	hypothetical protein CNAG_06712	0	2	0	2
CNAG_06710	chr2	251902	252661	759	null	hypothetical protein CNAG_06710	0	1	0	1
CNAG_06706	chr2	262148	263310	1162	null	proline-, glutamic acid- and leucine-rich protein 1	0	1	0	1
CNAG_03597	chr2	290217	294055	3838	null	hypothetical protein CNAG_03597	0	0	0	0
CNAG_03599	chr2	296389	298911	2522	null	mandelate racemase/muconate lactonizing enzyme	1	0	0	1
CNAG_03600	chr2	299137	304126	4989	STE6	ATP-binding cassette, subfamily B (MDR/TAP), member 1	1	0	2	3
CNAG_07964	chr2	304415	309697	5282	null	hypothetical protein CNAG_07964	1	0	1	2
CNAG_03603	chr2	313707	315542	1835	null	ATP-dependent RNA helicase HAS1	0	1	0	1
CNAG_03606	chr2	319325	323593	4268	null	translation initiation factor aIF-2	1	0	0	1
CNAG_07469	chr2	332247	338382	6135	null	nuclear pore complex protein Nup98-Nup96	0	0	1	1
CNAG_03613	chr2	340710	342148	1438	null	hypothetical protein CNAG_03613	1	1	0	2
CNAG_07470	chr2	344145	348334	4189	PDE2	high-affinity phosphodiesterase	0	3	0	3
CNAG_03617	chr2	351126	352605	1479	CLP1	clampless protein 1	1	0	0	1
CNAG_03619	chr2	354385	356115	1730	null	solute carrier family 29 (equilibrative nucleoside transporter), member 1/2/3	0	0	1	1
CNAG_03620	chr2	356282	360711	4429	null	hypothetical protein CNAG_03620	1	0	0	1
CNAG_03622	chr2	362716	370139	7423	TAO3	cell polarity protein mor2	1	0	2	3
CNAG_03624	chr2	372749	374797	2048	null	pescadillo	0	1	0	1
CNAG_03625	chr2	375002	378300	3298	null	RNA polymerase I-specific transcription initiation factor RRN7	1	1	0	2
CNAG_03626	chr2	378584	383072	4488	null	actin cross-linking protein	0	0	2	2
CNAG_03627	chr2	383378	384100	722	CPA1	peptidyl-prolyl cis-trans isomerase	0	1	0	1
CNAG_03633	chr2	393383	396366	2983	null	hypothetical protein CNAG_03633	0	0	1	1
CNAG_03639	chr2	409491	410510	1019	null	hypothetical protein CNAG_03639	0	1	0	1
CNAG_03647	chr2	431568	432595	1027	null	hypothetical protein CNAG_03647	0	1	0	1
CNAG_03652	chr2	443400	444200	800	null	dynactin 6	0	1	0	1
CNAG_03653	chr2	444276	447039	2763	null	hypothetical protein CNAG_03653	1	0	0	1
CNAG_03657	chr2	459928	464677	4749	null	hypothetical protein CNAG_03657	1	0	0	1
CNAG_03671	chr2	496022	497313	1291	null	hypothetical protein CNAG_03671	1	0	1	2
CNAG_03672	chr2	497504	498464	960	null	BET3 family protein	0	1	0	1
CNAG_03675	chr2	506073	508341	2268	null	pumilio domain-containing protein	1	0	0	1
CNAG_03676	chr2	508503	510312	1809	null	D-lactate dehydrogenase	0	2	0	2
CNAG_03677	chr2	512980	514234	1254	null	hypothetical protein CNAG_03677	0	3	0	3
CNAG_03678	chr2	514537	516287	1750	null	hypothetical protein CNAG_03678	0	2	1	3

CNAG_03680	chr2	518108	522009	3901	ROM21	hypothetical protein CNAG_03680	1	1	0	2
CNAG_03682	chr2	525258	526002	744	FRR1	FK506-binding protein 1	0	3	0	3
CNAG_03686	chr2	532833	533702	869	null	UDP-N-acetylglucosamine transferase subunit ALG14	0	1	0	1
CNAG_03687	chr2	533770	535221	1451	null	hypothetical protein CNAG_03687	1	0	1	2
CNAG_03689	chr2	538113	540467	2354	null	zf-C3HC4 type zinc finger protein	1	2	0	3
CNAG_07473	chr2	541201	545224	4023	null	valine-tRNA ligase	2	0	3	5
CNAG_03694	chr2	550195	551962	1767	CFT3	iron ion transporter	0	0	1	1
CNAG_03695	chr2	552218	554018	1800	CAS41	hypothetical protein CNAG_03695	0	0	1	1
CNAG_03699	chr2	563166	566689	3523	null	pre-mRNA-processing ATP-dependent RNA helicase PRP5	1	0	0	1
CNAG_03700	chr2	567432	568991	1559	null	hypothetical protein CNAG_03700	0	1	0	1
CNAG_03701	chr2	569253	574360	5107	null	3-phosphoshikimate 1-carboxyvinyltransferase	0	1	0	1
CNAG_03705	chr2	577804	578785	981	null	hypothetical protein CNAG_03705	0	1	0	1
CNAG_03707	chr2	582885	585017	2132	null	efflux protein	0	0	1	1
CNAG_07475	chr2	603345	603971	626	null	hypothetical protein CNAG_07475	0	1	0	1
CNAG_03714	chr2	604912	607619	2707	null	microtubule motor protein	0	1	0	1
CNAG_03719	chr2	617366	619662	2296	null	hypothetical protein CNAG_03719	1	0	0	1
CNAG_03722	chr2	622962	624823	1861	null	RAN protein binding protein	0	2	0	2
CNAG_03723	chr2	625352	628025	2673	null	nuclear protein	0	0	1	1
CNAG_03726	chr2	632368	635693	3325	null	hypothetical protein CNAG_03726	1	0	0	1
CNAG_03727	chr2	638234	642299	4065	null	hypothetical protein CNAG_03727	0	1	0	1
CNAG_03733	chr2	653371	656599	3228	null	hypothetical protein CNAG_03733	0	1	0	1
CNAG_03734	chr2	656879	659085	2206	null	hypothetical protein CNAG_03734	2	0	1	3
CNAG_03735	chr2	659468	662324	2856	CAP4	hypothetical protein CNAG_03735	1	3	0	4
CNAG_03740	chr2	670144	671151	1007	null	hypothetical protein CNAG_03740	1	3	0	4
CNAG_03743	chr2	678228	679908	1680	null	glutamine amidotransferase	1	0	0	1
CNAG_03745	chr2	681856	684633	2777	null	hypothetical protein CNAG_03745	0	0	1	1
CNAG_03747	chr2	686626	687348	722	null	large subunit ribosomal protein L27Ae	0	1	0	1
CNAG_03748	chr2	687877	696697	8820	null	large subunit ribosomal protein L19	1	0	0	1
CNAG_03756	chr2	712811	717414	4603	null	hypothetical protein CNAG_03756	0	1	1	2
CNAG_07479	chr2	718243	723403	5160	null	hypothetical protein CNAG_07479	1	0	1	2
CNAG_07480	chr2	723951	724484	533	null	cytochrome b5 reductase	1	0	0	1
CNAG_07203	chr2	724739	725133	394	null	hypothetical protein CNAG_07203	0	1	0	1
CNAG_03761	chr2	730717	734395	3678	null	mRNA 3\'-end-processing protein RNA14	0	1	1	2
CNAG_03763	chr2	738056	745073	7017	null	IQ domain-containing GTPase activating protein	1	0	1	2
CNAG_03764	chr2	745876	746925	1049	null	integral membrane protein	0	1	0	1
CNAG_03766	chr2	751385	752187	802	null	ankyrin repeat-containing protein	1	0	0	1
CNAG_03767	chr2	755293	760187	4894	null	cohesin complex subunit psm1	0	1	0	1
CNAG_03768	chr2	761022	764830	3808	null	hypothetical protein CNAG_03768	1	0	0	1
CNAG_03769	chr2	765734	767835	2101	HXK2	hexokinase	0	2	0	2
CNAG_03771	chr2	769393	771324	1931	null	DNA binding protein Ncp1	0	1	0	1
CNAG_03779	chr2	795887	797310	1423	null	large subunit ribosomal protein L4	0	1	0	1
CNAG_03780	chr2	797795	798508	713	null	small subunit ribosomal protein S16	0	1	0	1
CNAG_03781	chr2	799118	802091	2973	null	hypothetical protein CNAG_03781	1	0	0	1
CNAG_03784	chr2	806773	808974	2201	null	hypothetical protein CNAG_03784	1	0	0	1
CNAG_03785	chr2	809748	813025	3277	NTH1	alpha,alpha-trehalase	0	2	0	2
CNAG_03787	chr2	816598	818387	1789	null	tubulin alpha-1A chain	0	1	0	1
CNAG_03790	chr2	821960	823413	1453	null	hypothetical protein CNAG_03790	0	4	0	4

CNAG_03791	chr2	823857	827411	3554	null	CAMKK/ELM protein kinase	0	2	0	2
CNAG_03794	chr2	830386	832005	1619	null	endoplasmic reticulum protein	1	1	0	2
CNAG_03795	chr2	832152	833484	1332	null	N-glycosylase/DNA lyase	2	2	1	5
CNAG_03796	chr2	833610	835098	1488	null	NAK protein kinase	0	2	1	3
CNAG_03797	chr2	836678	837565	887	null	hypothetical protein CNAG_03797	1	0	0	1
CNAG_03806	chr2	895239	896745	1506	null	acyltransferase	1	0	0	1
CNAG_03809	chr2	902648	904845	2197	null	hypothetical protein CNAG_03809	1	1	1	3
CNAG_03810	chr2	905044	905703	659	null	large subunit ribosomal protein L13	0	1	0	1
CNAG_03811	chr2	906032	908066	2034	null	AGC/YANK protein kinase	0	0	1	1
CNAG_03812	chr2	909282	910649	1367	null	histidinol-phosphate transaminase	0	1	0	1
CNAG_03817	chr2	916894	918012	1118	null	cell growth-regulating nucleolar protein	1	0	1	2
CNAG_03818	chr2	918188	922308	4120	SSK1	osomolarity two-component system, response regulator SSK1	1	0	0	1
CNAG_03821	chr2	927804	931107	3303	null	phosphatidylinositol 3-kinase	1	0	0	1
CNAG_03824	chr2	935139	936568	1429	null	solute carrier family 25 (mitochondrial phosphate transporter), member 3	0	1	0	1
CNAG_03827	chr2	944584	947747	3163	null	hypothetical protein CNAG_03827	0	1	0	1
CNAG_03834	chr2	961619	962767	1148	null	C4-hydroxylase	0	1	0	1
CNAG_03836	chr2	965821	969037	3216	null	hypothetical protein CNAG_03836	1	1	0	2
CNAG_03839	chr2	974291	977393	3102	null	CCR4-NOT transcription complex subunit 4	0	0	1	1
CNAG_03841	chr2	981171	984024	2853	null	hypothetical protein CNAG_03841	1	0	0	1
CNAG_03842	chr2	985088	987205	2117	null	hypothetical protein CNAG_03842	0	0	1	1
CNAG_03845	chr2	995079	997036	1957	FNX1	multidrug resistance protein fnx1	2	0	0	2
CNAG_03848	chr2	1001888	1003267	1379	null	glutathione transferase	0	0	1	1
CNAG_03849	chr2	1004300	1007396	3096	null	nuclear protein	0	1	0	1
CNAG_03851	chr2	1011831	1013622	1791	null	general transcription factor 3C polypeptide 5 (transcription factor C subunit 1)	1	1	0	2
CNAG_03855	chr2	1018971	1020357	1386	null	phosphatidylinositol glycan, class M	1	0	0	1
CNAG_03858	chr2	1025017	1026188	1171	null	hypothetical protein CNAG_03858	0	1	0	1
CNAG_03870	chr2	1050281	1052310	2029	null	delta8-fatty-acid desaturase	1	0	0	1
CNAG_03872	chr2	1053858	1057038	3180	UBP7	hypothetical protein CNAG_03872	0	0	1	1
CNAG_03874	chr2	1060823	1062593	1770	null	oxidoreductase	0	1	0	1
CNAG_03875	chr2	1063020	1064739	1719	null	galactose-1-phosphate uridylyltransferase	0	2	0	2
CNAG_07483	chr2	1075267	1082121	6854	null	DNA polymerase zeta subunit	2	0	0	2
CNAG_03881	chr2	1082420	1085122	2702	null	hypothetical protein CNAG_03881	1	1	0	2
CNAG_03886	chr2	1096775	1099234	2459	null	hypothetical protein CNAG_03886	1	0	0	1
CNAG_03888	chr2	1100510	1104612	4102	null	hypothetical protein CNAG_03888	2	1	0	3
CNAG_03889	chr2	1105521	1107480	1959	null	hypothetical protein CNAG_03889	1	0	0	1
CNAG_03891	chr2	1109513	1111488	1975	null	hsp60-like protein	0	0	1	1
CNAG_03892	chr2	1111703	1112241	538	null	chaperonin GroES	0	1	0	1
CNAG_03893	chr2	1112833	1115678	2845	null	MAP kinase phosphatase	1	0	0	1
CNAG_03894	chr2	1118740	1121260	2520	PDR802	hypothetical protein CNAG_03894	0	0	1	1
CNAG_03896	chr2	1124274	1124771	497	null	hypothetical protein CNAG_03896	0	1	1	2
CNAG_03900	chr2	1133139	1134280	1141	null	capping protein (actin filament) muscle Z-line, beta	0	1	0	1
CNAG_03902	chr2	1136697	1139074	2377	null	transcriptional regulatory protein	1	0	0	1
CNAG_03903	chr2	1139343	1144047	4704	null	hypothetical protein CNAG_03903	1	0	0	1
CNAG_03913	chr2	1170550	1171779	1229	null	hypothetical protein CNAG_03913	0	1	0	1
CNAG_03915	chr2	1176924	1178939	2015	null	hypothetical protein CNAG_03915	1	0	0	1
CNAG_03916	chr2	1179182	1181157	1975	null	glucose-6-phosphate isomerase	0	0	3	3
CNAG_03918	chr2	1184421	1188494	4073	SOG2	hypothetical protein CNAG_03918	1	1	0	2

CNAG_03920	chr2	1189966	1191832	1866	IDP1	isocitrate dehydrogenase, NADP-dependent	0	0	1	1
CNAG_03922	chr2	1195192	1196243	1051	null	hypothetical protein CNAG_03922	1	0	0	1
CNAG_03924	chr2	1200363	1201530	1167	null	nit protein 1	0	1	0	1
CNAG_03925	chr2	1201630	1202446	816	null	mitochondrial inner membrane protease subunit 2	0	2	0	2
CNAG_03927	chr2	1204515	1205024	509	null	hypothetical protein CNAG_03927	1	0	0	1
CNAG_03934	chr2	1216983	1219987	3004	null	hypothetical protein CNAG_03934	0	0	1	1
CNAG_03936	chr2	1224001	1225045	1044	null	NAD(P)H:quinone oxidoreductase, type IV	0	1	0	1
CNAG_03940	chr2	1233055	1235712	2657	null	elongator complex protein 2	1	0	0	1
CNAG_03944	chr2	1247201	1248817	1616	null	chaperone regulator	0	1	0	1
CNAG_03945	chr2	1249162	1250872	1710	null	hypothetical protein CNAG_03945	1	0	1	2
CNAG_03946	chr2	1250994	1252977	1983	null	galactokinase	0	2	0	2
CNAG_07486	chr2	1253370	1254845	1475	null	hypothetical protein CNAG_07486	1	0	0	1
CNAG_03950	chr2	1259254	1264033	4779	null	transcription elongation factor SPT6	1	0	2	3
CNAG_03955	chr2	1270670	1272900	2230	null	L-methionine transporter	0	1	0	1
CNAG_03958	chr2	1276338	1277454	1116	null	hypothetical protein CNAG_03958	0	1	0	1
CNAG_03960	chr2	1279126	1281494	2368	null	Gly-Xaa carboxypeptidase	2	0	0	2
CNAG_03963	chr2	1287314	1288092	778	null	tyrosine phosphatase	0	0	1	1
CNAG_03966	chr2	1291567	1295373	3806	null	hypothetical protein CNAG_03966	1	0	0	1
CNAG_07488	chr2	1300913	1302248	1335	null	hypothetical protein CNAG_07488	0	1	0	1
CNAG_03980	chr2	1324369	1325526	1157	null	hypothetical protein CNAG_03980	0	1	0	1
CNAG_03987	chr2	1336238	1339607	3369	null	elongation factor 1 alpha-like protein	0	1	0	1
CNAG_03991	chr2	1347083	1348997	1914	null	integral membrane protein	0	1	0	1
CNAG_03994	chr2	1353697	1355038	1341	null	leucine carboxyl methyltransferase 1	0	2	1	3
CNAG_03997	chr2	1359050	1359775	725	null	hypothetical protein CNAG_03997	0	0	1	1
CNAG_03998	chr2	1360135	1362029	1894	RLM1	transcriptional activator	1	1	1	3
CNAG_04005	chr2	1376966	1379479	2513	null	cytoplasmic protein	0	0	1	1
CNAG_04011	chr2	1394939	1395680	741	null	large subunit ribosomal protein L37a	0	1	0	1
CNAG_04023	chr2	1423254	1425631	2377	null	hypothetical protein CNAG_04023	0	1	0	1
CNAG_07490	chr2	1426853	1427065	212	null	hypothetical protein CNAG_07490	0	1	0	1
CNAG_04024	chr2	1427982	1429640	1658	ITR5	sugar transporter	0	1	0	1
CNAG_04026	chr2	1433470	1436840	3370	null	chromosome transmission fidelity protein 1	0	1	0	1
CNAG_04028	chr2	1439809	1441317	1508	null	RNA binding protein	1	0	0	1
CNAG_04029	chr2	1441586	1443774	2188	null	cytochrome P450	1	0	0	1
CNAG_04032	chr2	1447920	1450587	2667	null	AFG3 family protein	0	3	0	3
CNAG_04034	chr2	1456128	1457453	1325	null	hypothetical protein CNAG_04034	1	1	0	2
CNAG_04035	chr2	1458188	1459831	1643	null	hypothetical protein CNAG_04035	0	2	0	2
CNAG_04036	chr2	1461932	1464161	2229	null	hypothetical protein CNAG_04036	0	1	0	1
CNAG_04037	chr2	1464768	1465158	390	null	hypothetical protein CNAG_04037	0	1	0	1
CNAG_04038	chr2	1465738	1467836	2098	null	MFS quinate transporter QutD	0	1	0	1
CNAG_04040	chr2	1473636	1477094	3458	null	AGC/RSK protein kinase	0	1	0	1
CNAG_04041	chr2	1477635	1481103	3468	null	hypothetical protein CNAG_04041	0	1	0	1
CNAG_04042	chr2	1481660	1482842	1182	null	hypothetical protein CNAG_04042	0	0	1	1
CNAG_04044	chr2	1485397	1486948	1551	null	U3 small nucleolar RNA-associated protein 6	1	1	0	2
CNAG_04047	chr2	1489438	1492448	3010	null	hypothetical protein CNAG_04047	1	0	0	1
CNAG_04048	chr2	1492664	1494320	1656	null	53 kda brg1-associated factor b	0	1	0	1
CNAG_04051	chr2	1498701	1503979	5278	null	ATP-dependent RNA helicase A	1	0	0	1
CNAG_04052	chr2	1504206	1506898	2692	null	minichromosome maintenance protein 5 (cell division control protein 46)	1	0	0	1

CNAG_04054	chr2	1508529	1509321	792	null	translation initiation factor SUI1	0	0	1	1
CNAG_04055	chr2	1509523	1511096	1573	null	cell cycle checkpoint protein	0	0	1	1
CNAG_04056	chr2	1511765	1513171	1406	null	hypothetical protein CNAG_04056	0	0	2	2
CNAG_07491	chr2	1513644	1515025	1381	null	glutaredoxin	0	6	0	6
CNAG_04059	chr2	1522304	1523732	1428	null	hypothetical protein CNAG_04059	0	1	0	1
CNAG_04060	chr2	1524759	1525830	1071	null	hypothetical protein CNAG_04060	0	1	0	1
CNAG_04065	chr2	1535509	1536230	721	null	UDP-N-acetylglucosamine transferase subunit ALG13	0	1	0	1
CNAG_04068	chr2	1539324	1540537	1213	null	large subunit ribosomal protein L28e	0	2	0	2
CNAG_04070	chr2	1545501	1549164	3663	null	exonuclease	0	1	1	2
CNAG_04072	chr2	1551864	1552785	921	null	60S ribosome subunit biogenesis protein nip7	0	0	1	1
CNAG_04073	chr2	1553822	1557873	4051	null	hypothetical protein CNAG_04073	1	2	0	3
CNAG_07494	chr2	1562709	1565306	2597	null	U3 small nucleolar RNA-associated protein MPP10	0	1	0	1
CNAG_04077	chr2	1566587	1566931	344	null	hypothetical protein CNAG_04077	0	2	0	2
CNAG_04083	chr2	1578334	1581957	3623	null	dolichol kinase	1	0	0	1
CNAG_04084	chr2	1582631	1584222	1591	null	hypothetical protein CNAG_04084	0	1	0	1
CNAG_04085	chr2	1584721	1586410	1689	null	oxidoreductase	1	2	1	4
CNAG_04086	chr2	1587286	1589210	1924	null	hypothetical protein CNAG_04086	1	0	0	1
CNAG_04087	chr2	1589326	1590517	1191	null	hypothetical protein CNAG_04087	1	0	0	1
CNAG_04092	chr2	1597881	1600252	2371	null	sugar transporter	0	1	0	1
CNAG_04097	chr2	1611266	1612295	1029	null	glucose 1-dehydrogenase	0	1	0	1
CNAG_07499	chr3	23655	28012	4357	CHS8	chitin synthase	1	0	0	1
CNAG_06963	chr3	29042	31344	2302	null	sugar transporter	0	0	1	1
CNAG_06962	chr3	33850	35487	1637	null	DNA ligase D, 3\'-phosphoesterase domain-containing protein	1	0	0	1
CNAG_03081	chr3	38076	40211	2135	null	ATP-dependent RNA helicase MRH4, mitochondrial	1	0	1	2
CNAG_03080	chr3	41347	42926	1579	null	fatty acid elongase	0	1	0	1
CNAG_03072	chr3	58337	60254	1917	null	enolase	0	2	0	2
CNAG_03071	chr3	61057	62943	1886	null	hypothetical protein CNAG_03071	0	1	0	1
CNAG_03069	chr3	65593	66479	886	null	hydrolase	0	1	0	1
CNAG_07502	chr3	70244	72682	2438	null	CTP synthase	1	0	0	1
CNAG_03063	chr3	75785	81790	6005	null	hypothetical protein CNAG_03063	1	0	1	2
CNAG_03061	chr3	87231	89316	2085	null	multiple drug resistance protein	0	2	0	2
CNAG_03059	chr3	94461	98231	3770	null	hypothetical protein CNAG_03059	0	1	0	1
CNAG_03056	chr3	103262	103756	494	null	hypothetical protein CNAG_03056	0	1	0	1
CNAG_03052	chr3	110998	113374	2376	null	PP2Cc protein phosphatase	0	0	1	1
CNAG_03051	chr3	114225	116505	2280	null	polyamine transporter	1	2	0	3
CNAG_03040	chr3	136251	138980	2729	null	transketolase	1	1	1	3
CNAG_03039	chr3	139453	141286	1833	null	DNA damage-inducible protein 1	0	0	1	1
CNAG_03038	chr3	141568	142728	1160	null	hypothetical protein CNAG_03038	0	5	0	5
CNAG_03037	chr3	143154	145181	2027	null	hypothetical protein CNAG_03037	1	0	2	3
CNAG_03036	chr3	145411	148931	3520	null	potassium ion transporter	2	4	0	6
CNAG_03033	chr3	154789	156462	1673	null	hypothetical protein CNAG_03033	0	1	0	1
CNAG_07505	chr3	159574	161769	2195	null	hypothetical protein CNAG_07505	2	0	0	2
CNAG_03027	chr3	163293	165866	2573	null	hypothetical protein CNAG_03027	0	1	0	1
CNAG_03024	chr3	169916	179043	9127	null	AGC protein kinase	0	1	1	2
CNAG_03023	chr3	179367	183887	4520	null	hypothetical protein CNAG_03023	1	0	0	1
CNAG_03021	chr3	186079	187177	1098	null	hypothetical protein CNAG_03021	1	0	0	1
CNAG_03019	chr3	190971	194264	3293	null	long-chain acyl-CoA synthetase	0	1	0	1

CNAG_03018	chr3	194871	197973	3102	null	nuclear protein	1	0	1	2
CNAG_03016	chr3	201565	202822	1257	null	chaperone regulator	1	1	0	2
CNAG_03015	chr3	203560	204231	671	null	large subunit ribosomal protein L37-A	0	1	0	1
CNAG_07968	chr3	204592	205299	707	null	hypothetical protein CNAG_07968	1	1	0	2
CNAG_03013	chr3	210067	213646	3579	null	OPT family small oligopeptide transporter	0	2	1	3
CNAG_03012	chr3	214987	215316	329	CQS1	hypothetical protein CNAG_03012	0	3	0	3
CNAG_03010	chr3	220906	221961	1055	null	enoyl-CoA hydratase/isomerase	1	0	0	1
CNAG_03006	chr3	226673	228241	1568	null	hypothetical protein CNAG_03006	1	0	0	1
CNAG_03005	chr3	228528	230400	1872	null	cytoplasmic tRNA 2-thiolation protein 2	1	0	0	1
CNAG_03000	chr3	237769	238595	826	null	small subunit ribosomal protein S19e	0	0	1	1
CNAG_02998	chr3	242767	245370	2603	null	nuclear protein	1	0	0	1
CNAG_02996	chr3	250219	251873	1654	null	flavoprotein oxygenase	1	0	1	2
CNAG_02994	chr3	254194	256955	2761	null	hypothetical protein CNAG_02994	0	1	1	2
CNAG_02992	chr3	259219	262340	3121	null	actin cross-linking protein	1	0	0	1
CNAG_02987	chr3	271663	272151	488	null	hypothetical protein CNAG_02987	0	1	1	2
CNAG_02986	chr3	272800	273842	1042	YSA1	ADP-ribose pyrophosphatase	0	1	0	1
CNAG_02982	chr3	281012	284130	3118	null	U3 small nucleolar RNA-associated protein 13	0	1	0	1
CNAG_02981	chr3	284939	289349	4410	null	paired amphipathic helix protein Sin3a	0	0	2	2
CNAG_02980	chr3	289651	291374	1723	null	membrane dipeptidase	0	4	0	4
CNAG_02979	chr3	291696	292554	858	null	hypothetical protein CNAG_02979	0	2	0	2
CNAG_02977	chr3	293809	299197	5388	null	metal resistance protein ycf1	0	2	1	3
CNAG_02976	chr3	299807	300857	1050	null	riboflavin kinase	0	0	1	1
CNAG_02973	chr3	304991	307000	2009	null	tRNA-splicing endonuclease subunit Sen2	1	0	1	2
CNAG_07506	chr3	308419	312400	3981	null	transcriptional repressor NF-X1	0	0	1	1
CNAG_02966	chr3	321349	323321	1972	null	carboxypeptidase	0	0	1	1
CNAG_02958	chr3	340928	343460	2532	CFO2	ferroxidase	0	0	1	1
CNAG_07508	chr3	343722	346389	2667	null	hypothetical protein CNAG_07508	1	1	0	2
CNAG_02953	chr3	354734	359365	4631	null	tuberin	0	1	0	1
CNAG_02952	chr3	359857	360316	459	null	hypothetical protein CNAG_02952	0	1	0	1
CNAG_02935	chr3	408450	409860	1410	null	malonic semialdehyde reductase	1	0	0	1
CNAG_02930	chr3	418733	424166	5433	LIV10	hypothetical protein CNAG_02930	0	1	0	1
CNAG_02928	chr3	429945	431085	1140	null	large subunit ribosomal protein L5e	0	1	0	1
CNAG_02918	chr3	451551	453152	1601	ERG10	acetyl-CoA C-acetyltransferase	0	3	0	3
CNAG_02914	chr3	461120	461690	570	null	hypothetical protein CNAG_02914	0	1	0	1
CNAG_02912	chr3	464580	470233	5653	null	hypothetical protein CNAG_02912	1	0	1	2
CNAG_07513	chr3	475748	483235	7487	null	hypothetical protein CNAG_07513	2	0	2	4
CNAG_02902	chr3	491291	493345	2054	null	hypothetical protein CNAG_02902	0	1	0	1
CNAG_02900	chr3	496093	497574	1481	null	hypothetical protein CNAG_02900	1	0	0	1
CNAG_02897	chr3	502414	506263	3849	null	inositol hexaphosphate kinase 1	1	0	0	1
CNAG_02895	chr3	509518	512528	3010	null	dynamin GTPase	0	1	0	1
CNAG_07514	chr3	523749	528930	5181	null	Sec7 domain-containing protein	0	0	1	1
CNAG_02886	chr3	529599	530477	878	null	20S proteasome subunit beta 4	0	1	0	1
CNAG_02885	chr3	530663	532716	2053	CAP64	capsular associated protein	0	1	0	1
CNAG_02882	chr3	538335	540967	2632	null	hypothetical protein CNAG_02882	0	0	1	1
CNAG_02878	chr3	546042	547552	1510	null	hypothetical protein CNAG_02878	1	0	0	1
CNAG_02877	chr3	547943	550950	3007	null	hypothetical protein CNAG_02877	0	1	0	1
CNAG_02876	chr3	554303	556055	1752	null	ornithine decarboxylase	1	0	0	1

CNAG_02874	chr3	558511	560817	2306	null	hypothetical protein CNAG_02874	1	0	0	1
CNAG_02871	chr3	563939	565364	1425	null	GPI mannosyltransferase 2	1	1	0	2
CNAG_02870	chr3	565694	567528	1834	MET7	folylpolyglutamate synthase	0	0	2	2
CNAG_02869	chr3	568106	569325	1219	null	hypothetical protein CNAG_02869	1	0	0	1
CNAG_02868	chr3	569838	571647	1809	null	tryptophan 2,3-dioxygenase	1	1	0	2
CNAG_02867	chr3	571915	574220	2305	PLC1	phosphatidylinositol phospholipase C, delta	0	1	1	2
CNAG_02866	chr3	575711	577623	1912	null	pantothenate kinase	0	1	0	1
CNAG_02862	chr3	584703	585766	1063	null	aprataxin	0	0	1	1
CNAG_02861	chr3	585936	587108	1172	null	histidinol-phosphatase (PHP family)	0	1	0	1
CNAG_02860	chr3	587686	589359	1673	EBG1	endo-1,3(4)-beta-glucanase	0	1	0	1
CNAG_02858	chr3	593524	595077	1553	ADE12	adenylosuccinate synthetase	0	1	0	1
CNAG_02857	chr3	595927	598628	2701	null	hypothetical protein CNAG_02857	0	0	1	1
CNAG_02856	chr3	599657	603445	3788	null	potassium ion transporter	0	1	0	1
CNAG_07515	chr3	606771	607159	388	null	hypothetical protein CNAG_07515	0	1	0	1
CNAG_02853	chr3	609351	611437	2086	null	amidophosphoribosyltransferase	0	1	0	1
CNAG_02850	chr3	616119	618961	2842	null	glucan endo-1,3-alpha-glucosidase agn1	0	0	1	1
CNAG_02849	chr3	620274	621973	1699	null	glutathione transferase	0	1	0	1
CNAG_02846	chr3	627835	628825	990	null	endoplasmic reticulum protein	0	1	0	1
CNAG_07518	chr3	648083	648775	692	null	ribonuclease P/MRP protein subunit POP5	1	0	0	1
CNAG_07519	chr3	651365	653839	2474	null	hypothetical protein CNAG_07519	1	0	0	1
CNAG_02834	chr3	654633	660773	6140	null	sterol 3-beta-glucosyltransferase	2	0	0	2
CNAG_02833	chr3	661078	661587	509	null	NADH dehydrogenase (ubiquinone) 1 beta subcomplex 7	0	1	1	2
CNAG_02829	chr3	666913	669761	2848	null	protein arginine N-methyltransferase 5	1	0	1	2
CNAG_02828	chr3	670025	673916	3891	null	hypothetical protein CNAG_02828	0	0	1	1
CNAG_07521	chr3	679391	680522	1131	null	hypothetical protein CNAG_07521	0	1	0	1
CNAG_02824	chr3	680662	682542	1880	null	hypothetical protein CNAG_02824	1	1	0	2
CNAG_02823	chr3	682979	687313	4334	null	bloom syndrome protein	0	0	1	1
CNAG_02822	chr3	690055	692624	2569	null	hypothetical protein CNAG_02822	0	1	0	1
CNAG_02821	chr3	693145	694240	1095	null	hypothetical protein CNAG_02821	1	0	0	1
CNAG_02820	chr3	694530	697611	3081	null	serine/threonine protein kinase	0	0	1	1
CNAG_02816	chr3	703591	706829	3238	null	nuclear pore complex protein Nup93	1	0	0	1
CNAG_02814	chr3	710187	713045	2858	null	glycerol-3-phosphate dehydrogenase	0	0	1	1
CNAG_02813	chr3	713735	714091	356	null	large subunit ribosomal protein L30	0	2	0	2
CNAG_02810	chr3	718237	721401	3164	PUF4	pumilio-family RNA binding protein	0	1	0	1
CNAG_02809	chr3	722162	724328	2166	null	tRNA-specific adenosine deaminase 3	0	2	0	2
CNAG_07522	chr3	724656	727126	2470	null	hypothetical protein CNAG_07522	1	0	0	1
CNAG_02805	chr3	730909	732084	1175	CAN1	carbonic anhydrase	1	0	0	1
CNAG_02801	chr3	737916	738395	479	TRX1	thioredoxin	0	2	0	2
CNAG_02800	chr3	740099	742134	2035	null	hypothetical protein CNAG_02800	1	0	0	1
CNAG_02798	chr3	745033	746303	1270	null	hypothetical protein CNAG_02798	0	1	0	1
CNAG_02796	chr3	748810	751177	2367	null	3-deoxy-7-phosphoheptulonate synthase	0	0	1	1
CNAG_02794	chr3	752230	753995	1765	null	dihydroorotate dehydrogenase (fumarate)	0	1	1	2
CNAG_02790	chr3	760517	761891	1374	null	galactinol synthase	1	0	0	1
CNAG_02789	chr3	762163	764383	2220	null	nitrogen permease regulator 2	0	1	0	1
CNAG_02787	chr3	766380	767417	1037	null	dephospho-CoA kinase	0	0	2	2
CNAG_02784	chr3	772889	774819	1930	null	methyltransferase	1	0	0	1
CNAG_02783	chr3	775278	775961	683	null	hypothetical protein CNAG_02783	0	1	0	1

CNAG_02782	chr3	776653	778280	1627	null	cytoplasmic protein	0	1	0	1
CNAG_02779	chr3	782657	783830	1173	BNA6	nicotinate-nucleotide diphosphorylase (carboxylating)	1	0	0	1
CNAG_02774	chr3	795298	798346	3048	null	hypothetical protein CNAG_02774	0	1	0	1
CNAG_02754	chr3	839792	840350	558	null	small subunit ribosomal protein S12e	0	2	0	2
CNAG_02753	chr3	840610	841657	1047	LIV13	endoplasmic reticulum protein	0	1	0	1
CNAG_02752	chr3	842066	843445	1379	null	short-chain dehydrogenase	0	2	0	2
CNAG_02745	chr3	856228	859574	3346	DCR2	hypothetical protein CNAG_02745	1	0	1	2
CNAG_02742	chr3	863625	866936	3311	DCR1	hypothetical protein CNAG_02742	3	1	0	4
CNAG_02739	chr3	871611	872618	1007	null	hypothetical protein CNAG_02739	0	1	0	1
CNAG_07524	chr3	883001	887280	4279	null	AMP deaminase	0	1	0	1
CNAG_02726	chr3	895869	897240	1371	null	cytoplasmic protein	0	1	0	1
CNAG_02724	chr3	900407	902986	2579	null	hypothetical protein CNAG_02724	0	2	0	2
CNAG_02721	chr3	908292	913363	5071	null	leucine repeat containing protein	1	0	0	1
CNAG_02720	chr3	913720	915992	2272	null	nuclear GTP-binding protein	0	0	1	1
CNAG_02719	chr3	919194	920123	929	null	hypothetical protein CNAG_02719	0	1	0	1
CNAG_02714	chr3	934045	935060	1015	null	elongation factor 1-beta	0	1	0	1
CNAG_02708	chr3	947499	949483	1984	null	prenylcysteine oxidase/farnesylcysteine lyase	0	1	0	1
CNAG_02705	chr3	953836	955693	1857	null	hypothetical protein CNAG_02705	0	1	0	1
CNAG_07525	chr3	958088	958410	322	null	hypothetical protein CNAG_07525	1	0	0	1
CNAG_02702	chr3	964531	968031	3500	CLC1	chloride channel protein	0	0	1	1
CNAG_02701	chr3	968296	970302	2006	null	hypothetical protein CNAG_02701	0	0	1	1
CNAG_02700	chr3	971147	976289	5142	null	hypothetical protein CNAG_02700	0	1	2	3
CNAG_02698	chr3	982804	985910	3106	null	hypothetical protein CNAG_02698	1	0	0	1
CNAG_02696	chr3	989903	994294	4391	null	exocyst protein	0	1	0	1
CNAG_02691	chr3	1006194	1007512	1318	null	hypothetical protein CNAG_02691	0	1	0	1
CNAG_07527	chr3	1011025	1012866	1841	null	alpha-1,6-mannosyltransferase	0	0	1	1
CNAG_02688	chr3	1012919	1013995	1076	null	hypothetical protein CNAG_02688	0	1	0	1
CNAG_02687	chr3	1014238	1015390	1152	null	hypothetical protein CNAG_02687	1	1	0	2
CNAG_02686	chr3	1015753	1018781	3028	null	cystathionine beta-lyase	0	1	0	1
CNAG_02682	chr3	1031882	1035603	3721	null	hypothetical protein CNAG_02682	1	0	0	1
CNAG_02678	chr3	1046719	1048071	1352	null	hypothetical protein CNAG_02678	0	1	0	1
CNAG_02675	chr3	1052864	1056306	3442	null	CAMK/CAMKL/GIN4 protein kinase	0	1	0	1
CNAG_02672	chr3	1061545	1063506	1961	null	nonsense-mediated mRNA decay protein 3	0	1	0	1
CNAG_02671	chr3	1063862	1066707	2845	null	pre-mRNA-splicing factor CEF1	0	1	0	1
CNAG_02670	chr3	1067198	1069493	2295	null	bud emergence protein 1	2	2	1	5
CNAG_07528	chr3	1070630	1072131	1501	null	hypothetical protein CNAG_07528	2	0	0	2
CNAG_02667	chr3	1072493	1074865	2372	null	hypothetical protein CNAG_02667	1	0	0	1
CNAG_02663	chr3	1083642	1085388	1746	null	ribonucleoside-diphosphate reductase subunit M2	0	1	0	1
CNAG_02661	chr3	1091888	1093184	1296	null	hypothetical protein CNAG_02661	2	0	0	2
CNAG_02658	chr3	1098942	1101345	2403	null	hypothetical protein CNAG_02658	1	0	1	2
CNAG_02657	chr3	1106965	1108197	1232	null	translation initiation factor 3 subunit G	1	0	0	1
CNAG_02654	chr3	1111497	1118965	7468	null	DNA polymerase epsilon catalytic subunit A	1	0	1	2
CNAG_07530	chr3	1121512	1125359	3847	null	hypothetical protein CNAG_07530	2	0	2	4
CNAG_07531	chr3	1125506	1126545	1039	null	pyridoxal 5\'-phosphate synthase, glutaminase subunit Pdx2	0	0	1	1
CNAG_07534	chr3	1129762	1134299	4537	null	hypothetical protein CNAG_07534	1	0	1	2
CNAG_07537	chr3	1137033	1138600	1567	null	mitochondrial GTPase 1	1	0	0	1
CNAG_07538	chr3	1138764	1141131	2367	null	calcium/proton exchanger	1	1	1	3

CNAG_07539	chr3	1141959	1145910	3951	null	replication factor C subunit 1	0	1	0	1
CNAG_07541	chr3	1152503	1153579	1076	null	proteasome assembly chaperone 2	1	1	0	2
CNAG_07542	chr3	1154045	1155441	1396	null	hypothetical protein CNAG_07542	0	1	0	1
CNAG_07548	chr3	1167887	1169216	1329	null	cytoplasmic protein	1	0	0	1
CNAG_07553	chr3	1187112	1189928	2816	null	polycomb protein e(z)	0	1	0	1
CNAG_07108	chr3	1192668	1197066	4398	null	tip120-family protein	1	0	0	1
CNAG_07555	chr3	1198130	1200003	1873	null	hypothetical protein CNAG_07555	0	0	1	1
CNAG_07557	chr3	1202311	1203106	795	null	ATP-binding cassette transporter	0	1	0	1
CNAG_07561	chr3	1209055	1211133	2078	null	6-phosphogluconate dehydrogenase, decarboxylating 1	0	1	0	1
CNAG_07564	chr3	1219253	1221622	2369	null	hypothetical protein CNAG_07564	0	0	1	1
CNAG_07566	chr3	1223107	1225525	2418	null	hypothetical protein CNAG_07566	1	1	0	2
CNAG_07567	chr3	1225741	1226968	1227	null	hypothetical protein CNAG_07567	0	1	0	1
CNAG_07970	chr3	1227127	1229427	2300	null	crossover junction endonuclease EME1	1	0	0	1
CNAG_07570	chr3	1229698	1230607	909	null	prenylated SNARE protein Ykt6p	0	1	0	1
CNAG_07572	chr3	1233865	1235975	2110	ELP3	elongator complex protein 3	0	1	0	1
CNAG_02603	chr3	1252670	1254831	2161	null	early growth response protein 1	0	0	1	1
CNAG_02600	chr3	1258840	1260846	2006	null	tartrate transporter	0	2	0	2
CNAG_02598	chr3	1262794	1264647	1853	CHI21	chitinase	0	4	0	4
CNAG_02597	chr3	1265342	1267499	2157	null	hypothetical protein CNAG_02597	0	1	0	1
CNAG_02596	chr3	1268321	1268928	607	null	hypothetical protein CNAG_02596	1	0	0	1
CNAG_02595	chr3	1269576	1271694	2118	null	hypothetical protein CNAG_02595	0	1	0	1
CNAG_02594	chr3	1272594	1274900	2306	null	hypothetical protein CNAG_02594	2	0	0	2
CNAG_02593	chr3	1277117	1279712	2595	null	hypothetical protein CNAG_02593	1	2	0	3
CNAG_02590	chr3	1283994	1286080	2086	null	3-hydroxyisobutyrate dehydrogenase	0	1	0	1
CNAG_02588	chr3	1290204	1293033	2829	null	avenacinase	1	0	0	1
CNAG_02586	chr3	1297973	1300246	2273	null	sugar transporter	1	0	0	1
CNAG_02579	chr3	1313768	1318691	4923	null	hypothetical protein CNAG_02579	0	0	1	1
CNAG_02574	chr3	1329506	1333595	4089	null	cofactor D	4	1	0	5
CNAG_07574	chr3	1337863	1338207	344	null	hypothetical protein CNAG_07574	1	0	0	1
CNAG_07575	chr3	1339252	1340005	753	null	hypothetical protein CNAG_07575	1	0	0	1
CNAG_02568	chr3	1344066	1349237	5171	null	UBA/TS-N domain-containing protein	3	0	1	4
CNAG_02567	chr3	1349411	1350346	935	null	hypothetical protein CNAG_02567	0	1	0	1
CNAG_02566	chr3	1351585	1354444	2859	null	hepatocyte nuclear factor	2	0	0	2
CNAG_02562	chr3	1363696	1365992	2296	null	acyl-CoA dehydrogenase	0	1	0	1
CNAG_07971	chr3	1366526	1366936	410	null	hypothetical protein CNAG_07971	0	2	0	2
CNAG_02561	chr3	1368380	1370391	2011	null	spermine transporter	0	1	0	1
CNAG_07972	chr3	1373905	1374861	956	null	hypothetical protein CNAG_07972	0	3	0	3
CNAG_06881	chr3	1412227	1413220	993	null	SBDS family rRNA metabolism protein	0	1	0	1
CNAG_06882	chr3	1413437	1413904	467	null	hypothetical protein CNAG_06882	0	1	0	1
CNAG_07580	chr3	1422354	1426421	4067	null	CAMK protein kinase	0	0	1	1
CNAG_06889	chr3	1431670	1433335	1665	null	sodium-hydrogen antiporter	0	4	0	4
CNAG_06890	chr3	1434628	1437056	2428	null	membrane transporter	1	4	0	5
CNAG_06891	chr3	1439204	1440114	910	null	hypothetical protein CNAG_06891	0	1	0	1
CNAG_06892	chr3	1440427	1442012	1585	null	preconditioning-inducible protein	0	0	1	1
CNAG_06893	chr3	1442238	1443480	1242	null	hypothetical protein CNAG_06893	0	3	0	3
CNAG_06895	chr3	1444887	1445696	809	null	hypothetical protein CNAG_06895	0	0	2	2
CNAG_06910	chr3	1493211	1494402	1191	null	beta-lactamase	0	1	0	1

CNAG_06925	chr3	1533629	1535516	1887	null	arsenical-resistance protein	1	0	0	1
CNAG_06926	chr3	1535940	1539122	3182	null	hypothetical protein CNAG_06926	0	3	1	4
CNAG_06927	chr3	1540111	1542239	2128	null	peptidyl-prolyl isomerase G (cyclophilin G)	2	0	0	2
CNAG_06928	chr3	1542773	1546689	3916	null	hypothetical protein CNAG_06928	2	3	0	5
CNAG_06929	chr3	1547282	1550105	2823	null	IQ domain-containing GTPase activating protein	0	0	1	1
CNAG_06930	chr3	1551213	1551785	572	null	hypothetical protein CNAG_06930	0	1	0	1
CNAG_06931	chr3	1553308	1556158	2850	null	beta-glucosidase	0	1	0	1
CNAG_07583	chr3	1558846	1561544	2698	null	hypothetical protein CNAG_07583	0	2	0	2
CNAG_06934	chr3	1562223	1564413	2190	null	hexose transporter protein	1	2	3	6
CNAG_06936	chr3	1568423	1571422	2999	null	beta-glucosidase	0	0	1	1
CNAG_04932	chr4	10120	12222	2102	null	hypothetical protein CNAG_04932	0	0	1	1
CNAG_04935	chr4	17510	18922	1412	null	hypothetical protein CNAG_04935	0	1	0	1
CNAG_07795	chr4	36004	36603	599	null	hypothetical protein CNAG_07795	0	2	0	2
CNAG_04943	chr4	40735	43906	3171	null	CBS domain-containing protein	0	2	1	3
CNAG_04944	chr4	46200	47727	1527	null	hypothetical protein CNAG_04944	0	2	1	3
CNAG_04946	chr4	50213	52234	2021	null	hypothetical protein CNAG_04946	0	0	1	1
CNAG_04947	chr4	53252	55084	1832	null	high-affinity nicotinic acid transporter	1	0	0	1
CNAG_04949	chr4	58197	59901	1704	null	hypothetical protein CNAG_04949	0	1	1	2
CNAG_04951	chr4	61878	63276	1398	null	3-deoxy-7-phosphoheptulonate synthase	0	3	1	4
CNAG_04952	chr4	63998	64982	984	null	ADP-ribosylation factor 6	0	1	0	1
CNAG_04953	chr4	67011	68082	1071	null	hypothetical protein CNAG_04953	0	1	0	1
CNAG_04955	chr4	73079	74559	1480	null	oxidoreductase	0	1	1	2
CNAG_04963	chr4	93774	95312	1538	null	hypothetical protein CNAG_04963	0	1	0	1
CNAG_04967	chr4	106657	108392	1735	null	hypothetical protein CNAG_04967	1	0	1	2
CNAG_04968	chr4	109301	110977	1676	null	hypothetical protein CNAG_04968	0	1	0	1
CNAG_04970	chr4	115198	116444	1246	null	hypothetical protein CNAG_04970	1	2	0	3
CNAG_07800	chr4	116759	119764	3005	null	syntaxin-binding protein 1	1	0	0	1
CNAG_04974	chr4	125166	126901	1735	null	hypothetical protein CNAG_04974	1	0	0	1
CNAG_04989	chr4	160924	162111	1187	null	protein FRG1	0	0	1	1
CNAG_04992	chr4	171059	173815	2756	null	hypothetical protein CNAG_04992	0	1	0	1
CNAG_07802	chr4	181736	183468	1732	null	class III aminotransferase	0	0	2	2
CNAG_05000	chr4	190648	193513	2865	null	hypothetical protein CNAG_05000	1	1	0	2
CNAG_05001	chr4	193685	194793	1108	null	hypothetical protein CNAG_05001	1	0	0	1
CNAG_05005	chr4	204147	207494	3347	null	ULK/ULK protein kinase	0	0	1	1
CNAG_05008	chr4	213563	214433	870	null	hypothetical protein CNAG_05008	0	0	1	1
CNAG_05009	chr4	215088	216750	1662	null	integral membrane protein	0	0	1	1
CNAG_05013	chr4	226391	228877	2486	null	RNP domain-containing protein	1	0	0	1
CNAG_05017	chr4	235727	237978	2251	null	GabA permease	0	1	1	2
CNAG_05019	chr4	240125	242641	2516	null	hypothetical protein CNAG_05019	0	1	0	1
CNAG_05026	chr4	253643	254756	1113	null	mango esterase	0	1	0	1
CNAG_05037	chr4	278579	279700	1121	null	hypothetical protein CNAG_05037	1	0	0	1
CNAG_05039	chr4	281409	283556	2147	null	hypothetical protein CNAG_05039	0	1	0	1
CNAG_05041	chr4	285064	285995	931	null	NADH-ubiquinone oxidoreductase subunit 8	0	2	0	2
CNAG_05042	chr4	286205	288871	2666	null	carnitine acetyltransferase	1	0	0	1
CNAG_05043	chr4	290187	293185	2998	null	hypothetical protein CNAG_05043	1	0	0	1
CNAG_05046	chr4	295976	297690	1714	null	hypothetical protein CNAG_05046	1	0	0	1
CNAG_07804	chr4	298523	299112	589	null	protein mgr2	0	1	0	1

CNAG_05049	chr4	303877	306784	2907	null	hypothetical protein CNAG_05049	1	8	1	10
CNAG_07806	chr4	308594	310161	1567	null	hypothetical protein CNAG_07806	3	2	0	5
CNAG_07807	chr4	310772	311242	470	null	histone H4	0	2	0	2
CNAG_05053	chr4	311904	313883	1979	null	hypothetical protein CNAG_05053	4	0	3	7
CNAG_05055	chr4	314520	315733	1213	null	DNA/RNA-binding protein KIN17	0	0	1	1
CNAG_05056	chr4	316251	318711	2460	null	hypothetical protein CNAG_05056	0	0	2	2
CNAG_05057	chr4	318929	319989	1060	null	hypothetical protein CNAG_05057	0	0	1	1
CNAG_05058	chr4	321106	322772	1666	null	GTPase	0	1	0	1
CNAG_05059	chr4	322999	324723	1724	null	pyruvate dehydrogenase E1 component subunit beta	0	1	0	1
CNAG_05062	chr4	330035	332667	2632	PUt2	hypothetical protein CNAG_05062	1	0	0	1
CNAG_05063	chr4	332951	337782	4831	SSK2	STE/STE11/SSK protein kinase	0	1	0	1
CNAG_05064	chr4	338852	340830	1978	null	hypothetical protein CNAG_05064	1	0	1	2
CNAG_05066	chr4	342075	342968	893	null	hypothetical protein CNAG_05066	3	4	1	8
CNAG_05067	chr4	343299	344709	1410	null	hypothetical protein CNAG_05067	1	0	0	1
CNAG_05070	chr4	350591	355120	4529	null	sulfite reductase (NADPH) hemoprotein, beta-component	0	2	1	3
CNAG_05072	chr4	359287	361218	1931	null	endopeptidase	1	0	1	2
CNAG_07808	chr4	363041	364493	1452	null	alanine-glyoxylate transaminase/serine-glyoxylate transaminase/serine-pyruvate transaminase	1	0	0	1
CNAG_05074	chr4	364960	368540	3580	null	hypothetical protein CNAG_05074	2	0	0	2
CNAG_05075	chr4	369285	371679	2394	null	solute carrier family 20 (sodium-dependent phosphate transporter)	1	1	2	4
CNAG_05076	chr4	372638	373673	1035	null	hypothetical protein CNAG_05076	1	1	0	2
CNAG_05078	chr4	379063	380909	1846	null	tRNA \'-O-ribosylphosphate transferase	1	0	0	1
CNAG_05079	chr4	381334	381640	306	null	hypothetical protein CNAG_05079	1	4	0	5
CNAG_05084	chr4	400452	403535	3083	null	phospholipase A-2-activating protein	1	0	1	2
CNAG_05092	chr4	417487	417930	443	null	hypothetical protein CNAG_05092	0	1	0	1
CNAG_05093	chr4	419046	420465	1419	null	hypothetical protein CNAG_05093	0	1	1	2
CNAG_05096	chr4	423624	425305	1681	null	histone deacetylase 1/2	0	0	1	1
CNAG_05111	chr4	455799	456599	800	null	hypothetical protein CNAG_05111	1	0	0	1
CNAG_05116	chr4	470498	472762	2264	null	DNA polymerase mu subunit	1	0	0	1
CNAG_05120	chr4	481882	483663	1781	null	nicotinate phosphoribosyltransferase	1	0	0	1
CNAG_05124	chr4	490981	494050	3069	SEY1	protein SEY1	1	0	1	2
CNAG_05125	chr4	494508	495818	1310	null	diphosphomevalonate decarboxylase	0	0	3	3
CNAG_07812	chr4	496075	496979	904	null	hypothetical protein CNAG_07812	0	3	0	3
CNAG_05129	chr4	500541	502358	1817	null	hypothetical protein CNAG_05129	1	0	0	1
CNAG_05130	chr4	503516	506168	2652	null	hypothetical protein CNAG_05130	0	2	0	2
CNAG_05132	chr4	507849	508616	767	null	cytochrome c oxidase subunit 5b	0	2	0	2
CNAG_07815	chr4	509408	512924	3516	null	hypothetical protein CNAG_07815	0	1	0	1
CNAG_05134	chr4	513249	515061	1812	null	ornithine-oxo-acid transaminase	0	0	1	1
CNAG_05135	chr4	515400	519102	3702	PMR1	Ca2 -transporting ATPase	0	2	0	2
CNAG_05137	chr4	524272	525082	810	null	ER lumen protein retaining receptor	0	1	0	1
CNAG_05142	chr4	541004	542639	1635	null	dolichyl-P-Man:Man(5)GlcNAc(2)-PP-dolichyl mannosyltransferase	0	1	0	1
CNAG_05155	chr4	575331	579175	3844	PTP2	protein-tyrosine-phosphatase	1	0	0	1
CNAG_05157	chr4	584063	585667	1604	null	hypothetical protein CNAG_05157	1	0	0	1
CNAG_05167	chr4	608717	609108	391	null	hypothetical protein CNAG_05167	0	1	0	1
CNAG_05169	chr4	612267	614736	2469	null	L-lactate dehydrogenase (cytochrome)	0	1	0	1
CNAG_05171	chr4	620055	621219	1164	null	CCR4-NOT transcription complex subunit 7/8	0	1	0	1
CNAG_05172	chr4	621447	626221	4774	null	myosin-1	1	0	0	1
CNAG_05176	chr4	637755	639810	2055	null	hypothetical protein CNAG_05176	0	0	1	1

CNAG_05178	chr4	643572	644736	1164	null	hypothetical protein CNAG_05178	0	2	0	2
CNAG_05181	chr4	652296	655892	3596	BWC1	white collar 1 protein	1	0	0	1
CNAG_05185	chr4	666198	667166	968	null	hypothetical protein CNAG_05185	2	0	0	2
CNAG_05187	chr4	669989	672530	2541	null	hypothetical protein CNAG_05187	1	0	0	1
CNAG_05190	chr4	677146	679665	2519	null	hypothetical protein CNAG_05190	1	0	1	2
CNAG_05191	chr4	679785	682090	2305	null	hypothetical protein CNAG_05191	0	0	1	1
CNAG_05192	chr4	683091	683787	696	null	hypothetical protein CNAG_05192	0	1	0	1
CNAG_05193	chr4	684267	686109	1842	null	endoplasmic reticulum-Golgi intermediate compartment protein 2	0	0	1	1
CNAG_05194	chr4	686196	687772	1576	null	lipoyl synthase, mitochondrial	0	0	1	1
CNAG_05195	chr4	688303	692601	4298	null	ubiquitin-conjugation factor E4 B	1	0	0	1
CNAG_05199	chr4	697238	699688	2450	null	chaperone DnaK	0	1	0	1
CNAG_05202	chr4	706757	708652	1895	null	SET domain-containing protein 6	0	2	0	2
CNAG_05212	chr4	748322	749539	1217	null	hypothetical protein CNAG_05212	1	3	1	5
CNAG_05218	chr4	759395	761302	1907	ACA1	adenylyl cyclase-associated protein	1	0	0	1
CNAG_05221	chr4	769364	770024	660	null	histone H2A.Z	0	1	1	2
CNAG_05222	chr4	772148	773296	1148	NRG1	transcriptional regulator Nrg1	0	1	0	1
CNAG_05231	chr4	791421	795114	3693	null	nuclear protein	1	0	0	1
CNAG_05233	chr4	797015	800591	3576	null	hypothetical protein CNAG_05233	1	1	1	3
CNAG_05234	chr4	801161	802491	1330	null	mitochondrial import inner membrane translocase subunit TIM54	0	1	0	1
CNAG_05235	chr4	802785	803830	1045	null	protein BMH2	0	1	0	1
CNAG_05242	chr4	815463	819580	4117	null	hypothetical protein CNAG_05242	1	0	0	1
CNAG_05243	chr4	820542	822630	2088	null	xylulokinase	1	1	0	2
CNAG_05244	chr4	823296	825448	2152	null	hypothetical protein CNAG_05244	0	2	1	3
**CNAG_05245**	**chr4**	**829807**	**830546**	**739**	**null**	**hypothetical protein CNAG_05245**	**0**	**20**	**0**	**20**
**CNAG_05247**	**chr4**	**832949**	**834336**	**1387**	**SLC1**	**lysophosphatidate acyltransferase**	**1**	**6**	**4**	**11**
**CNAG_05248**	**chr4**	**834753**	**837513**	**2760**	**null**	**clathrin binding protein**	**2**	**0**	**10**	**12**
CNAG_07976	chr4	837936	839980	2044	null	hypothetical protein CNAG_07976	1	0	0	1
CNAG_05250	chr4	842196	845206	3010	null	telomere maintenance protein	0	2	0	2
CNAG_05251	chr4	847184	848301	1117	null	hypothetical protein CNAG_05251	0	0	2	2
CNAG_05253	chr4	850754	853253	2499	null	hypothetical protein CNAG_05253	0	0	1	1
CNAG_05265	chr4	883176	883685	509	null	hypothetical protein CNAG_05265	0	1	0	1
CNAG_05268	chr4	889302	890404	1102	null	hypothetical protein CNAG_05268	0	2	0	2
CNAG_05269	chr4	890674	891711	1037	null	20S proteasome subunit alpha 7	1	0	0	1
CNAG_05277	chr4	914359	915384	1025	null	vesicle-associated membrane protein 4	0	4	0	4
CNAG_05279	chr4	919179	920373	1194	null	hypothetical protein CNAG_05279	1	0	0	1
CNAG_05284	chr4	933269	935072	1803	null	hypothetical protein CNAG_05284	1	0	0	1
CNAG_05285	chr4	935348	936641	1293	null	hypothetical protein CNAG_05285	0	1	0	1
CNAG_05286	chr4	936737	937461	724	null	hypothetical protein CNAG_05286	0	1	0	1
CNAG_05294	chr4	963168	966186	3018	null	F-box and WD-40 domain-containing protein CDC4	0	4	0	4
CNAG_05301	chr4	981421	984577	3156	CRN1	deoxyuridine 5\'-triphosphate nucleotidohydrolase	0	1	0	1
CNAG_05306	chr4	999579	1001988	2409	null	hypothetical protein CNAG_05306	0	1	0	1
CNAG_05307	chr4	1002227	1005172	2945	null	beta-catenin-like protein 1	1	0	0	1
CNAG_05308	chr4	1005495	1010119	4624	null	beta-catenin-like protein 1	2	0	0	2
CNAG_05309	chr4	1011242	1013033	1791	null	hypothetical protein CNAG_05309	0	1	0	1
CNAG_05310	chr4	1013505	1014760	1255	null	nipsnap family protein	1	0	1	2
CNAG_05311	chr4	1015086	1018217	3131	null	arsenite-resistant protein ASR2	2	1	0	3
CNAG_05312	chr4	1018841	1020605	1764	null	hypothetical protein CNAG_05312	0	1	1	2

CNAG_05313	chr4	1022725	1027663	4938	null	hypothetical protein CNAG_05313	0	1	1	2
CNAG_05314	chr4	1028055	1029862	1807	GLO3	ADP-ribosylation factor GTPase-activating protein 2/3	0	2	4	6
CNAG_05315	chr4	1030558	1032411	1853	null	taurine catabolism dioxygenase TauD	0	3	0	3
CNAG_05316	chr4	1036081	1037236	1155	null	inositol oxygenase	0	1	0	1
CNAG_05319	chr4	1044598	1045974	1376	null	hypothetical protein CNAG_05319	0	1	0	1
CNAG_07830	chr4	1052247	1055092	2845	null	hypothetical protein CNAG_07830	0	1	0	1
CNAG_05325	chr4	1059075	1060049	974	null	hypothetical protein CNAG_05325	0	2	0	2
CNAG_05326	chr4	1061591	1062658	1067	null	hypothetical protein CNAG_05326	3	0	0	3
CNAG_07395	chr5	2357	3284	927	null	hypothetical protein CNAG_07395	0	1	0	1
CNAG_06869	chr5	28218	29485	1267	null	HPP family protein	1	0	0	1
CNAG_06864	chr5	37955	40559	2604	null	hypothetical protein CNAG_06864	0	0	1	1
CNAG_06863	chr5	40787	41672	885	null	hypothetical protein CNAG_06863	2	1	1	4
CNAG_07978	chr5	55498	61778	6280	null	hypothetical protein CNAG_07978	0	1	0	1
CNAG_07400	chr5	75178	77551	2373	null	aspartate-tRNA(Asn) ligase	0	1	0	1
CNAG_06849	chr5	77880	79456	1576	null	saccharopine dehydrogenase (NAD, L-lysine forming)	0	0	1	1
CNAG_06847	chr5	80480	81642	1162	null	small subunit ribosomal protein S28	0	1	0	1
CNAG_06846	chr5	84959	85723	764	null	hypothetical protein CNAG_06846	1	0	0	1
CNAG_06845	chr5	86262	91908	5646	null	STE/STE11/CDC15 protein kinase	0	0	1	1
CNAG_06844	chr5	92004	92580	576	null	hypothetical protein CNAG_06844	0	0	1	1
**CNAG_07402**	**chr5**	**95502**	**98444**	**2942**	**null**	**pantothenate transporter**	**0**	**0**	**1**	**1**
CNAG_07403	chr5	98745	102743	3998	PEX1	peroxin-1	0	0	1	1
CNAG_06836	chr5	115847	117615	1768	null	oxidoreductase	0	2	1	3
CNAG_06834	chr5	122649	125839	3190	PMT1	dolichyl-phosphate-mannose-protein mannosyltransferase	1	1	1	3
CNAG_06833	chr5	126530	127742	1212	null	hypothetical protein CNAG_06833	1	0	0	1
CNAG_06831	chr5	132300	133921	1621	null	hypothetical protein CNAG_06831	1	0	0	1
CNAG_06830	chr5	134250	136975	2725	null	histidinol dehydrogenase	0	3	0	3
CNAG_06827	chr5	144531	145721	1190	null	hypothetical protein CNAG_06827	0	2	0	2
CNAG_06826	chr5	146447	148300	1853	null	hypothetical protein CNAG_06826	3	0	0	3
CNAG_06825	chr5	148644	150362	1718	null	hypothetical protein CNAG_06825	1	3	1	5
CNAG_06824	chr5	151236	153083	1847	null	hypothetical protein CNAG_06824	0	2	0	2
CNAG_06822	chr5	154814	157479	2665	null	hypothetical protein CNAG_06822	2	0	0	2
CNAG_07405	chr5	172163	174967	2804	null	hypothetical protein CNAG_07405	0	1	0	1
CNAG_06817	chr5	178989	181172	2183	UAP1	NCS2 family nucleobase:cation symporter-2	1	0	0	1
CNAG_06816	chr5	182210	183288	1078	null	hypothetical protein CNAG_06816	0	3	0	3
CNAG_06813	chr5	189844	192096	2252	CAP1alpha	hypothetical protein CNAG_06813	0	1	1	2
CNAG_06812	chr5	194439	200633	6194	SPO14alpha	phospholipase D	1	0	0	1
CNAG_06804	chr5	215900	218759	2859	BSP3	hypothetical protein CNAG_06804	1	1	0	2
CNAG_06980	chr5	233883	237873	3990	STE11alpha	STE/STE11 protein kinase	0	1	1	2
CNAG_07409	chr5	238318	242691	4373	RPO41alpha	DNA-directed RNA polymerase, mitochondrial	0	0	1	1
CNAG_07410	chr5	248166	251116	2950	CID1alpha	hypothetical protein CNAG_07410	0	3	0	3
CNAG_07015	chr5	252101	255269	3168	null	hypothetical protein CNAG_07015	0	1	0	1
CNAG_07411	chr5	256901	262890	5989	RUM1alpha	histone demethylase JARID1	1	0	0	1
CNAG_01455	chr5	270550	271048	498	RPL39alpha	large subunit ribosomal protein L39	0	1	0	1
CNAG_01443	chr5	296646	298152	1506	null	hypothetical protein CNAG_01443	1	0	0	1
CNAG_01441	chr5	300882	303499	2617	null	hypothetical protein CNAG_01441	0	0	1	1
CNAG_01440	chr5	304337	306257	1920	null	hypothetical protein CNAG_01440	0	1	1	2
CNAG_01438	chr5	308617	311733	3116	null	transcription factor	0	1	0	1

CNAG_01437	chr5	312056	313440	1384	null	ribosomal RNA assembly protein	0	1	0	1
CNAG_01436	chr5	313552	314882	1330	null	serine/threonine-protein phosphatase 6 catalytic subunit	0	0	1	1
CNAG_07415	chr5	317189	320945	3756	null	hypothetical protein CNAG_07415	0	1	2	3
CNAG_01432	chr5	323603	325550	1947	null	ribosome assembly protein 4	0	1	0	1
CNAG_01430	chr5	327665	329129	1464	null	hypothetical protein CNAG_01430	1	0	0	1
CNAG_01429	chr5	329941	331320	1379	null	hypothetical protein CNAG_01429	0	1	0	1
CNAG_01428	chr5	331619	332308	689	null	translation initiation factor 5A	0	1	0	1
CNAG_01425	chr5	335648	336605	957	null	hypothetical protein CNAG_01425	1	0	0	1
CNAG_01423	chr5	341846	344488	2642	null	hypothetical protein CNAG_01423	0	1	1	2
CNAG_01418	chr5	361299	363414	2115	null	hypothetical protein CNAG_01418	1	0	0	1
CNAG_07418	chr5	382626	383887	1261	DCN1	defective in Cullin neddylation protein 1	0	0	1	1
CNAG_01408	chr5	383991	389100	5109	null	PH and SEC7 domain-containing protein	1	1	1	3
CNAG_01404	chr5	394173	396133	1960	null	hsp71-like protein	1	0	2	3
CNAG_01403	chr5	396216	397306	1090	null	eclair-PA	0	1	0	1
CNAG_01402	chr5	398223	399984	1761	null	actin binding protein	0	1	1	2
CNAG_01401	chr5	400560	401317	757	null	hypothetical protein CNAG_01401	1	1	0	2
CNAG_01400	chr5	402242	403675	1433	null	3-deoxy-7-phosphoheptulonate synthase	0	1	0	1
CNAG_01398	chr5	405853	409462	3609	null	ATP-dependent RNA helicase DOB1	0	1	0	1
CNAG_07419	chr5	414828	416247	1419	null	hypothetical protein CNAG_07419	1	0	0	1
CNAG_07420	chr5	418428	419470	1042	null	hypothetical protein CNAG_07420	0	10	1	11
CNAG_01392	chr5	419671	421105	1434	null	hypothetical protein CNAG_01392	1	0	0	1
CNAG_01391	chr5	421166	422868	1702	null	cell division cycle protein 37	1	1	2	4
CNAG_01390	chr5	423131	423827	696	null	adenine phosphoribosyltransferase	0	0	4	4
CNAG_01389	chr5	424539	426692	2153	null	hypothetical protein CNAG_01389	0	8	0	8
CNAG_01388	chr5	426854	429303	2449	null	hypothetical protein CNAG_01388	3	0	0	3
CNAG_01387	chr5	429734	431165	1431	null	hypothetical protein CNAG_01387	0	0	1	1
CNAG_01385	chr5	432334	435188	2854	null	hypothetical protein CNAG_01385	1	0	0	1
CNAG_01376	chr5	455582	459840	4258	null	stress response protein NST1	0	1	1	2
CNAG_01375	chr5	460132	462846	2714	ABP1	hypothetical protein CNAG_01375	1	0	0	1
CNAG_01373	chr5	468400	469873	1473	CDC10	septin ring protein	0	1	0	1
CNAG_01371	chr5	473665	475148	1483	CRG2	hypothetical protein CNAG_01371	0	2	0	2
CNAG_01365	chr5	488612	491066	2454	null	hypothetical protein CNAG_01365	1	1	0	2
CNAG_01363	chr5	492372	493795	1423	null	XPA-binding protein 1	0	0	1	1
CNAG_01362	chr5	494228	497195	2967	null	cell cycle control protein cwf19	0	1	0	1
CNAG_01361	chr5	497478	499748	2270	null	importin alpha subunit	0	1	1	2
CNAG_01358	chr5	507376	508453	1077	null	hypothetical protein CNAG_01358	0	2	0	2
CNAG_01357	chr5	509051	510169	1118	null	hypothetical protein CNAG_01357	1	0	0	1
CNAG_01356	chr5	510657	515079	4422	null	hypothetical protein CNAG_01356	6	0	4	10
CNAG_01353	chr5	520201	522175	1974	null	hypothetical protein CNAG_01353	1	0	0	1
CNAG_07423	chr5	522243	522980	737	null	hypothetical protein CNAG_07423	0	2	0	2
CNAG_01352	chr5	523663	528046	4383	null	hypothetical protein CNAG_01352	0	0	1	1
CNAG_01350	chr5	529929	531337	1408	RMD5	hypothetical protein CNAG_01350	0	2	0	2
CNAG_01349	chr5	533989	535024	1035	null	hypothetical protein CNAG_01349	0	1	0	1
CNAG_01348	chr5	535256	536040	784	null	cyanate hydratase	0	1	0	1
CNAG_01344	chr5	543469	545266	1797	null	hypothetical protein CNAG_01344	0	0	1	1
CNAG_01343	chr5	545431	547793	2362	null	ATP-dependent Clp protease ATP-binding subunit ClpX	0	0	2	2
CNAG_01337	chr5	565291	567702	2411	null	WD-repeat protein	1	0	1	2

CNAG_01336	chr5	568067	569683	1616	null	hypothetical protein CNAG_01336	0	1	0	1
CNAG_01335	chr5	570276	572200	1924	null	hypothetical protein CNAG_01335	0	1	0	1
CNAG_01334	chr5	573160	577414	4254	null	hypothetical protein CNAG_01334	6	1	1	8
CNAG_01333	chr5	578252	579985	1733	null	Haspin protein kinase	1	0	0	1
CNAG_01332	chr5	580238	581006	768	null	small subunit ribosomal protein S24e	0	1	0	1
CNAG_01330	chr5	583261	586125	2864	null	hypothetical protein CNAG_01330	1	0	0	1
CNAG_07425	chr5	591908	592967	1059	null	hypothetical protein CNAG_07425	0	9	1	10
CNAG_07980	chr5	593519	593892	373	null	hypothetical protein CNAG_07980	0	2	0	2
CNAG_01323	chr5	596965	597857	892	null	ubiquinol-cytochrome c reductase subunit 7	0	3	0	3
CNAG_01322	chr5	598090	600034	1944	null	hypothetical protein CNAG_01322	1	0	0	1
CNAG_01321	chr5	600451	601242	791	null	acyl carrier protein	0	2	0	2
CNAG_01315	chr5	610846	613655	2809	null	protein transporter	1	0	0	1
CNAG_01314	chr5	613753	616586	2833	null	vacuolar protein	0	0	1	1
CNAG_07426	chr5	617681	618356	675	null	proteasome maturation protein	0	1	0	1
CNAG_01308	chr5	624613	627833	3220	null	protein NRD1	2	0	0	2
CNAG_01306	chr5	631028	634989	3961	null	chromodomain-helicase-DNA-binding protein 3	1	0	0	1
CNAG_01304	chr5	638540	641028	2488	null	polynucleotide 5\'-hydroxyl-kinase GRC3	1	0	0	1
CNAG_01300	chr5	647105	647673	568	null	small subunit ribosomal protein S21e	0	1	0	1
CNAG_01299	chr5	648147	650243	2096	null	hypothetical protein CNAG_01299	1	0	0	1
CNAG_01297	chr5	657032	658675	1643	null	hypothetical protein CNAG_01297	1	0	0	1
CNAG_01292	chr5	674899	678659	3760	null	pol II transcription elongation factor	0	1	0	1
CNAG_01288	chr5	681113	682711	1598	null	solute carrier family 25, member 33/36	0	1	0	1
CNAG_01278	chr5	709097	715033	5936	APT2	phospholipid-translocating ATPase	0	2	0	2
CNAG_01277	chr5	716067	717677	1610	null	hypothetical protein CNAG_01277	1	1	0	2
CNAG_01273	chr5	733079	734006	927	null	cellular nucleic acid-binding protein	0	1	0	1
CNAG_07429	chr5	740815	742564	1749	null	asparagine-tRNA ligase	0	1	0	1
CNAG_01263	chr5	754551	757599	3048	null	vacuolar transporter chaperone 4	1	0	1	2
CNAG_01261	chr5	759327	764045	4718	null	myosin I binding protein	1	0	0	1
CNAG_01258	chr5	779926	784257	4331	null	hypothetical protein CNAG_01258	2	0	3	5
CNAG_01256	chr5	786968	790460	3492	null	hypothetical protein CNAG_01256	1	0	1	2
CNAG_01255	chr5	791280	792869	1589	null	hypothetical protein CNAG_01255	0	0	2	2
CNAG_01254	chr5	793192	794050	858	null	protein-tyrosine-phosphatase	0	1	0	1
CNAG_01252	chr5	795508	796818	1310	null	thiosulfate/3-mercaptopyruvate sulfurtransferase	0	2	0	2
CNAG_01251	chr5	797289	803731	6442	UFD4	E3 ubiquitin-protein ligase TRIP12	1	0	1	2
CNAG_01250	chr5	804163	807327	3164	null	tRNA ligase	1	0	0	1
CNAG_01249	chr5	807765	810530	2765	null	hypothetical protein CNAG_01249	1	0	0	1
CNAG_01248	chr5	810891	814966	4075	null	vacuole morphology and inheritance protein 14	0	0	1	1
CNAG_01247	chr5	815203	816215	1012	null	hypothetical protein CNAG_01247	1	0	0	1
CNAG_01243	chr5	823454	828261	4807	SET101	histone-lysine N-methyltransferase, H3 lysine-4 specific	0	1	0	1
CNAG_01241	chr5	831923	833387	1464	null	enzyme regulator	0	2	0	2
CNAG_01240	chr5	834320	836419	2099	null	hypothetical protein CNAG_01240	0	0	1	1
CNAG_01239	chr5	839089	840616	1527	CDA3	chitin deacetylase	1	1	0	2
CNAG_01238	chr5	844297	846002	1705	null	arginine biosynthesis ArgJ, mitochondrial	1	0	0	1
CNAG_01233	chr5	859047	863718	4671	null	hypothetical protein CNAG_01233	1	0	0	1
CNAG_07432	chr5	879700	882293	2593	null	hypothetical protein CNAG_07432	2	0	0	2
CNAG_01223	chr5	886059	887234	1175	null	hypothetical protein CNAG_01223	0	1	0	1
CNAG_07433	chr5	891391	896593	5202	null	hypothetical protein CNAG_07433	2	0	0	2

CNAG_01217	chr5	896777	898875	2098	null	protein MPE1	0	0	1	1
CNAG_01208	chr5	920020	927591	7571	CCH1	high-affinity cell membrane calcium channel protein	0	2	0	2
CNAG_01207	chr5	927963	930606	2643	null	hypothetical protein CNAG_01207	0	1	0	1
CNAG_01201	chr5	940369	941039	670	null	hypothetical protein CNAG_01201	0	1	0	1
CNAG_01200	chr5	941267	942415	1148	null	pre-mRNA-splicing factor 38A	1	0	0	1
CNAG_01183	chr5	982919	985239	2320	LSB1	hypothetical protein CNAG_01183	1	0	0	1
CNAG_01173	chr5	1009270	1012596	3326	PAN1	hypothetical protein CNAG_01173	0	0	1	1
CNAG_01172	chr5	1013000	1015304	2304	PBX1	hypothetical protein CNAG_01172	0	1	0	1
CNAG_01171	chr5	1015576	1015988	412	null	hypothetical protein CNAG_01171	1	3	0	4
CNAG_01167	chr5	1021546	1026054	4508	URE2	chromosome associated protein	0	0	1	1
CNAG_01166	chr5	1026273	1027256	983	URE4	urease accessory protein	0	2	0	2
CNAG_01164	chr5	1030035	1032571	2536	null	glutamine-fructose-6-phosphate transaminase (isomerizing)	1	1	0	2
CNAG_01162	chr5	1040137	1041507	1370	null	hypothetical protein CNAG_01162	1	0	0	1
CNAG_01161	chr5	1041906	1042837	931	null	hypothetical protein CNAG_01161	1	0	0	1
CNAG_07439	chr5	1043611	1047025	3414	null	U3 small nucleolar RNA-associated protein 21	1	0	2	3
CNAG_01159	chr5	1047117	1049085	1968	null	pre-mRNA-splicing factor SLU7	1	1	0	2
CNAG_01157	chr5	1052515	1054242	1727	null	hypothetical protein CNAG_01157	0	1	0	1
CNAG_01156	chr5	1054904	1057196	2292	CAP2	hypothetical protein CNAG_01156	0	2	1	3
CNAG_01155	chr5	1057786	1060367	2581	null	glycerol kinase	0	0	1	1
CNAG_01154	chr5	1061029	1061759	730	null	hypothetical protein CNAG_01154	0	8	0	8
CNAG_01153	chr5	1065111	1065763	652	null	small subunit ribosomal protein S13e	0	5	0	5
CNAG_01150	chr5	1069146	1070825	1679	null	omega-6 fatty acid desaturase (delta-12 desaturase)	0	1	0	1
CNAG_01149	chr5	1071881	1077008	5127	null	hypothetical protein CNAG_01149	3	1	0	4
CNAG_01147	chr5	1079533	1080202	669	null	ARP2/3 complex 20 kDa subunit	0	1	0	1
CNAG_01146	chr5	1080317	1084402	4085	null	U3 small nucleolar RNA-associated protein 22	2	1	1	4
CNAG_01145	chr5	1084604	1085963	1359	null	hypothetical protein CNAG_01145	1	0	1	2
CNAG_01143	chr5	1088741	1090031	1290	null	hypothetical protein CNAG_01143	0	1	0	1
CNAG_01142	chr5	1090182	1094237	4055	null	endoplasmic reticulum protein	0	1	0	1
CNAG_01140	chr5	1095868	1098467	2599	null	ATP-dependent RNA helicase DRS1	0	1	0	1
CNAG_01136	chr5	1108324	1109491	1167	null	hypothetical protein CNAG_01136	1	0	1	2
CNAG_01134	chr5	1112268	1116037	3769	null	hypothetical protein CNAG_01134	2	1	0	3
CNAG_01127	chr5	1129874	1132018	2144	null	hypothetical protein CNAG_01127	1	0	0	1
CNAG_01126	chr5	1132708	1139266	6558	null	guanine nucleotide exchange protein for ADP-robosylation factor	1	0	1	2
CNAG_01121	chr5	1150000	1150562	562	null	hypothetical protein CNAG_01121	0	1	0	1
CNAG_01120	chr5	1151774	1153458	1684	null	pyruvate dehydrogenase complex dihydrolipoamide acetyltransferase	0	1	0	1
CNAG_01119	chr5	1154216	1156436	2220	null	POT family proton-dependent oligopeptide transporter	1	0	0	1
CNAG_01118	chr5	1158958	1160875	1917	null	AAT family amino acid transporter	1	0	0	1
CNAG_01117	chr5	1163379	1166733	3354	EF3	elongation factor 3	0	2	0	2
CNAG_01115	chr5	1169400	1179250	9850	null	hypothetical protein CNAG_01115	1	0	0	1
CNAG_01114	chr5	1179708	1180342	634	null	hypothetical protein CNAG_01114	0	0	1	1
CNAG_01111	chr5	1183338	1184415	1077	null	translation initiation factor 3 subunit K	0	1	0	1
CNAG_01108	chr5	1187761	1190690	2929	DAL2,3,3	allantoicase	0	0	1	1
CNAG_01106	chr5	1193990	1197049	3059	VPH1	V-type H -transporting ATPase subunit I	1	0	0	1
CNAG_01098	chr5	1218866	1221238	2372	null	pre-rRNA-processing protein IPI1	0	2	0	2
CNAG_01090	chr5	1234829	1235977	1148	null	alpha/beta hydrolase fold protein	1	0	0	1
CNAG_01087	chr5	1239999	1242199	2200	null	hypothetical protein CNAG_01087	0	0	1	1
CNAG_01084	chr5	1246223	1247208	985	UBC4	ubiquitin-conjugating enzyme 4	0	1	0	1

CNAG_01081	chr5	1255581	1256850	1269	null	hypothetical protein CNAG_01081	0	1	0	1
CNAG_01072	chr5	1277358	1278890	1532	null	amidase	0	1	0	1
CNAG_01070	chr5	1281913	1283146	1233	null	class II aldolase/adducin family protein	0	2	0	2
CNAG_01069	chr5	1284269	1287339	3070	null	hypothetical protein CNAG_01069	0	1	4	5
CNAG_07441	chr5	1287550	1288271	721	null	hypothetical protein CNAG_07441	2	2	1	5
CNAG_07443	chr5	1290948	1292419	1471	null	hypothetical protein CNAG_07443	0	2	1	3
CNAG_01062	chr5	1304684	1308392	3708	null	CAMK/CAMKL/PASK protein kinase	1	1	0	2
CNAG_01061	chr5	1309270	1312427	3157	null	serine/threonine protein kinase	1	1	1	3
CNAG_01060	chr5	1312651	1314259	1608	null	hypothetical protein CNAG_01060	0	1	0	1
CNAG_01059	chr5	1314602	1316331	1729	null	vacuolar membrane protein	0	2	1	3
CNAG_01058	chr5	1316412	1317526	1114	null	nitric oxide synthase-interacting protein	0	1	0	1
CNAG_07444	chr5	1321787	1323925	2138	null	hypothetical protein CNAG_07444	1	1	0	2
CNAG_01051	chr5	1338299	1342848	4549	null	dynactin 1	2	0	0	2
CNAG_01041	chr5	1364777	1365593	816	null	hypothetical protein CNAG_01041	0	1	0	1
CNAG_01040	chr5	1366829	1368770	1941	null	carboxypeptidase D	1	0	1	2
CNAG_01039	chr5	1370513	1375666	5153	null	superkiller protein 3	2	0	0	2
CNAG_01035	chr5	1382896	1384129	1233	null	20S proteasome subunit alpha 4	0	0	1	1
CNAG_01032	chr5	1387662	1392052	4390	null	hypothetical protein CNAG_01032	1	1	0	2
CNAG_01031	chr5	1393095	1394418	1323	null	hypothetical protein CNAG_01031	1	0	1	2
CNAG_01029	chr5	1396496	1398111	1615	null	impact family protein	0	1	0	1
CNAG_01026	chr5	1401928	1404670	2742	null	gamma-glutamyltransferase	0	0	1	1
CNAG_01025	chr5	1405036	1406480	1444	null	hypothetical protein CNAG_01025	0	1	0	1
CNAG_01023	chr5	1407975	1410303	2328	null	cohesin complex subunit SCC1	0	0	1	1
CNAG_01018	chr5	1418354	1420208	1854	null	histone-lysine N-methyltransferase Su(var)3-9	0	0	1	1
CNAG_01016	chr5	1427076	1431641	4565	null	vacuolar membrane protein	1	0	1	2
CNAG_01014	chr5	1434211	1435638	1427	null	hypothetical protein CNAG_01014	0	1	0	1
CNAG_01008	chr5	1445912	1447555	1643	null	hypothetical protein CNAG_01008	0	0	1	1
CNAG_01004	chr5	1454546	1457659	3113	null	hypothetical protein CNAG_01004	1	0	0	1
CNAG_01003	chr5	1458060	1460510	2450	NCP1	NADPH-ferrihemoprotein reductase	0	0	1	1
CNAG_01001	chr5	1464356	1466496	2140	null	hypothetical protein CNAG_01001	0	1	0	1
CNAG_00998	chr5	1473831	1478759	4928	null	hypothetical protein CNAG_00998	1	4	0	5
CNAG_00992	chr5	1488718	1490613	1895	null	homocitrate synthase, mitochondrial	0	0	1	1
CNAG_00989	chr5	1493795	1495134	1339	UCH2	ubiquitin carboxyl-terminal hydrolase L5	0	1	0	1
CNAG_00988	chr5	1495524	1498482	2958	null	importin subunit beta-1	0	1	0	1
CNAG_00987	chr5	1501726	1502669	943	null	prefoldin subunit	0	1	0	1
CNAG_00986	chr5	1502917	1504479	1562	UBA4	adenylyltransferase and sulfurtransferase	0	1	0	1
CNAG_00982	chr5	1511379	1512466	1087	null	hypothetical protein CNAG_00982	1	0	0	1
CNAG_00979	chr5	1517917	1518715	798	CTR4	solute carrier family 31 (copper transporter), member 1	0	2	0	2
CNAG_00978	chr5	1519054	1520492	1438	null	NADH dehydrogenase (ubiquinone) 1 alpha subcomplex 9	0	2	0	2
CNAG_00977	chr5	1520873	1522784	1911	null	VHS domain-containing protein	0	0	1	1
CNAG_00974	chr5	1528319	1529289	970	null	beta-lactamase	1	0	0	1
CNAG_00971	chr5	1532590	1535021	2431	null	vacuolar fusion protein MON1	0	1	0	1
CNAG_00968	chr5	1540456	1541665	1209	null	hypothetical protein CNAG_00968	0	0	1	1
CNAG_07445	chr5	1547048	1549801	2753	null	transketolase	0	1	0	1
CNAG_07446	chr5	1552259	1552624	365	null	mitochondrial protein	1	0	0	1
CNAG_00961	chr5	1553461	1554897	1436	null	hypothetical protein CNAG_00961	0	1	0	1
CNAG_07447	chr5	1561568	1562663	1095	null	hypothetical protein CNAG_07447	0	1	0	1

CNAG_00941	chr5	1580221	1580739	518	null	hypothetical protein CNAG_00941	0	1	0	1
CNAG_00940	chr5	1581510	1585029	3519	RRP44	exosome complex exonuclease DIS3/RRP44	0	2	0	2
CNAG_00938	chr5	1587848	1588748	900	null	hypothetical protein CNAG_00938	0	1	0	1
CNAG_00936	chr5	1591428	1593816	2388	null	lipid particle protein	1	2	1	4
CNAG_00927	chr5	1610777	1612244	1467	null	guanyl nucleotide binding protein	0	1	0	1
CNAG_00917	chr5	1634277	1636601	2324	null	alkylbase DNA N-glycosylase	1	0	0	1
CNAG_00915	chr5	1639675	1640862	1187	null	hypothetical protein CNAG_00915	1	0	0	1
CNAG_00914	chr5	1642229	1644317	2088	KRE6	glucosidase	0	1	0	1
CNAG_00908	chr5	1660563	1661540	977	null	hypothetical protein CNAG_00908	0	0	2	2
CNAG_00903	chr5	1676877	1677636	759	null	hypothetical protein CNAG_00903	0	1	0	1
CNAG_00895	chr5	1701697	1703029	1332	ZIP1	solute carrier family 39 (zinc transporter), member 1/2/3	0	1	0	1
CNAG_00894	chr5	1705377	1705847	470	null	hypothetical protein CNAG_00894	0	1	0	1
CNAG_00890	chr5	1714010	1716778	2768	null	hypothetical protein CNAG_00890	0	1	0	1
CNAG_07453	chr5	1736636	1738641	2005	null	hypothetical protein CNAG_07453	0	1	0	1
CNAG_00872	chr5	1761351	1765145	3794	null	ATP-dependent DNA helicase	2	1	0	3
CNAG_00871	chr5	1767017	1768854	1837	null	hypothetical protein CNAG_00871	0	1	0	1
CNAG_00870	chr5	1771379	1772152	773	null	hypothetical protein CNAG_00870	0	1	0	1
CNAG_00869	chr5	1775551	1780923	5372	PDR5	ATP-binding cassette transporter	0	1	1	2
CNAG_00865	chr5	1790357	1791427	1070	CAS92	maltose O-acetyltransferase	0	1	0	1
CNAG_00864	chr5	1793803	1795826	2023	ITR2	MFS transporter, SP family, solute carrier family 2 (myo-inositol transporter), member 13	0	1	0	1
CNAG_02557	chr6	3409	5731	2322	null	fungal specific transcription factor	0	2	0	2
CNAG_02554	chr6	11769	13915	2146	null	sugar transporter	1	0	1	2
CNAG_02549	chr6	22142	24277	2135	null	hypothetical protein CNAG_02549	0	1	0	1
CNAG_02544	chr6	39014	39503	489	null	DNA repair protein Swi5/Sae3	0	1	0	1
CNAG_02542	chr6	41711	42957	1246	null	fructosamine kinase	1	0	0	1
CNAG_02540	chr6	48961	50984	2023	null	hypothetical protein CNAG_02540	0	2	0	2
CNAG_02538	chr6	55387	56566	1179	null	hypothetical protein CNAG_02538	0	1	0	1
CNAG_02536	chr6	58257	60018	1761	TAF6	transcription initiation factor TFIID subunit 6	2	0	1	3
CNAG_02535	chr6	60162	61167	1005	null	hypothetical protein CNAG_02535	0	3	1	4
CNAG_02534	chr6	61282	62497	1215	null	tRNA 2\'-phosphotransferase	0	0	1	1
CNAG_02533	chr6	62964	64578	1614	null	hypothetical protein CNAG_02533	1	2	1	4
CNAG_02531	chr6	68671	70815	2144	CPK1	CMGC/MAPK protein kinase	0	1	0	1
CNAG_02530	chr6	71519	74200	2681	null	hypothetical protein CNAG_02530	1	0	0	1
CNAG_02528	chr6	75281	78208	2927	null	hypothetical protein CNAG_02528	0	0	2	2
CNAG_02527	chr6	78964	81426	2462	null	multidrug transporter	0	1	0	1
CNAG_02526	chr6	85040	85943	903	null	hypothetical protein CNAG_02526	0	4	1	5
CNAG_02525	chr6	87188	90889	3701	null	hypothetical protein CNAG_02525	1	2	1	4
CNAG_02523	chr6	95780	100320	4540	null	hypothetical protein CNAG_02523	1	1	1	3
CNAG_02522	chr6	101444	102809	1365	MRS3/4	solute carrier family 25 (mitochondrial iron transporter), member 28/37	0	0	1	1
CNAG_02520	chr6	104604	106188	1584	null	DNA-directed RNA polymerase III subunit RPC4	0	1	0	1
CNAG_02516	chr6	113130	115353	2223	null	hypothetical protein CNAG_02516	1	0	0	1
CNAG_02512	chr6	121394	125423	4029	null	DNA repair protein RAD16	0	0	1	1
CNAG_02510	chr6	131056	132984	1928	null	hypothetical protein CNAG_02510	1	1	0	2
CNAG_02509	chr6	133790	135331	1541	null	hypothetical protein CNAG_02509	2	0	1	3
CNAG_02508	chr6	135751	136694	943	null	hypothetical protein CNAG_02508	0	2	0	2
CNAG_02507	chr6	136977	138342	1365	null	translation initiation factor 3 subunit H	0	3	1	4
CNAG_02506	chr6	138743	139701	958	null	3,4-dihydroxy-2-butanone-4-phosphate synthase	1	0	0	1

CNAG_02505	chr6	140236	142235	1999	null	cardiolipin-specific phospholipase	1	1	0	2
CNAG_02504	chr6	142631	143872	1241	null	hypothetical protein CNAG_02504	1	0	1	2
CNAG_02498	chr6	153058	157357	4299	null	hypothetical protein CNAG_02498	1	1	0	2
CNAG_02497	chr6	157989	159923	1934	null	thaumatin family protein	1	1	0	2
CNAG_02489	chr6	178139	179391	1252	null	alcohol dehydrogenase, propanol-preferring	0	1	0	1
CNAG_02488	chr6	183631	186787	3156	null	methionyl-tRNA formyltransferase	1	0	5	6
CNAG_02487	chr6	187201	188372	1171	null	3-hydroxy acyl-CoA dehydratase	0	2	0	2
CNAG_02486	chr6	188521	189770	1249	null	translation initiation factor 2 subunit 2	0	2	0	2
CNAG_02485	chr6	190100	191393	1293	null	ATP synthase F1, gamma subunit	0	1	0	1
CNAG_07625	chr6	193040	196216	3176	null	plasma membrane protein	0	1	1	2
CNAG_02483	chr6	196925	198402	1477	null	hypothetical protein CNAG_02483	3	0	1	4
CNAG_02480	chr6	203319	205522	2203	null	cell cycle checkpoint control protein RAD9A	0	0	1	1
CNAG_02479	chr6	206610	209018	2408	null	monosaccharide transporter	0	1	0	1
CNAG_02476	chr6	213646	217692	4046	YRM101	hypothetical protein CNAG_02476	1	0	1	2
CNAG_02475	chr6	218037	220112	2075	null	flavin-containing monooxygenase	0	1	0	1
CNAG_02472	chr6	226624	228567	1943	null	endosomal P24A protein	0	0	1	1
CNAG_02470	chr6	230999	232844	1845	null	phosphoric monoester hydrolase	0	1	0	1
CNAG_02468	chr6	237920	241147	3227	null	AP-3 complex subunit delta-1	0	0	1	1
CNAG_02467	chr6	241356	245284	3928	null	DNA repair/transcription protein MET18/MMS19	1	1	0	2
CNAG_02466	chr6	245462	248249	2787	null	hypothetical protein CNAG_02466	0	1	0	1
CNAG_02465	chr6	249286	251415	2129	null	allantoate permease	1	0	0	1
CNAG_02464	chr6	252409	257109	4700	null	5-oxoprolinase (ATP-hydrolysing)	0	0	2	2
CNAG_02463	chr6	257762	259283	1521	null	hypothetical protein CNAG_02463	0	2	0	2
CNAG_07627	chr6	260608	266063	5455	null	hypothetical protein CNAG_07627	1	0	1	2
CNAG_02455	chr6	284161	286304	2143	null	choline transporter	0	2	0	2
CNAG_02454	chr6	287045	289269	2224	null	H/ACA ribonucleoprotein complex non-core subunit NAF1	0	0	1	1
CNAG_02452	chr6	296080	297253	1173	null	hypothetical protein CNAG_02452	1	0	0	1
CNAG_02449	chr6	302003	304647	2644	null	long-chain fatty acid CoA ligase	2	0	0	2
CNAG_02446	chr6	307664	308554	890	null	response regulator receiver protein	1	0	0	1
CNAG_02443	chr6	312979	313476	497	null	NADH dehydrogenase	0	1	0	1
CNAG_02439	chr6	320827	321804	977	null	hypothetical protein CNAG_02439	0	1	0	1
CNAG_02437	chr6	326214	331017	4803	null	rRNA biogenesis protein RRP5	2	0	0	2
CNAG_02433	chr6	336764	337917	1153	null	thiol oxidase	1	0	0	1
CNAG_02432	chr6	338758	341884	3126	null	hypothetical protein CNAG_02432	1	0	0	1
CNAG_02430	chr6	344225	349995	5770	null	ABC transporter ABCC.6	0	2	0	2
CNAG_02428	chr6	351815	354599	2784	null	GC-rich sequence DNA-binding factor	1	0	0	1
CNAG_02424	chr6	361099	363921	2822	null	hypothetical protein CNAG_02424	0	0	1	1
CNAG_02423	chr6	364246	365785	1539	null	hypothetical protein CNAG_02423	1	0	0	1
CNAG_02419	chr6	371109	373752	2643	null	hypothetical protein CNAG_02419	1	0	1	2
CNAG_02418	chr6	373890	375993	2103	null	asparagine-tRNA ligase	1	1	1	3
CNAG_02415	chr6	380495	382236	1741	null	annexin XIV	0	1	0	1
CNAG_02409	chr6	396438	397913	1475	LIV5	hypothetical protein CNAG_02409	1	2	0	3
CNAG_02407	chr6	400029	404250	4221	null	phospholipase D	0	0	1	1
CNAG_02406	chr6	404527	406934	2407	null	cell division control protein 45	1	0	1	2
CNAG_02401	chr6	418974	421895	2921	null	G patch domain-containing protein 1	1	1	0	2
CNAG_02396	chr6	435003	436506	1503	null	hypothetical protein CNAG_02396	1	0	0	1
CNAG_02395	chr6	437194	439715	2521	null	ubiquitin carboxyl-terminal hydrolase 9/13	0	1	0	1

CNAG_02392	chr6	449250	450070	820	null	hypothetical protein CNAG_02392	0	1	0	1
CNAG_02388	chr6	459756	460896	1140	null	hypothetical protein CNAG_02388	0	1	0	1
CNAG_02383	chr6	468156	469590	1434	null	trimethylguanosine synthase	0	1	1	2
CNAG_02381	chr6	470982	472579	1597	null	hypothetical protein CNAG_02381	1	0	0	1
CNAG_02378	chr6	476459	477292	833	null	H/ACA ribonucleoprotein complex subunit 2	0	1	0	1
CNAG_02376	chr6	479717	480995	1278	null	hypothetical protein CNAG_02376	0	1	0	1
CNAG_02375	chr6	481191	484674	3483	null	polyphosphoinositide phosphatase	0	2	2	4
CNAG_02374	chr6	485289	488190	2901	null	hypothetical protein CNAG_02374	0	0	2	2
CNAG_02373	chr6	488230	490771	2541	null	ATP-binding protein	2	3	4	9
CNAG_02371	chr6	492915	494179	1264	null	coiled-coil domain-containing protein 130	1	1	0	2
CNAG_02368	chr6	501233	503505	2272	null	hypothetical protein CNAG_02368	0	3	0	3
CNAG_02366	chr6	505479	507426	1947	null	4-aminobutyrate aminotransferase	1	0	0	1
CNAG_02364	chr6	510958	512315	1357	null	hypothetical protein CNAG_02364	1	0	0	1
CNAG_02363	chr6	512630	514606	1976	null	SCR1 protein	0	1	0	1
CNAG_02360	chr6	521383	522304	921	null	hypothetical protein CNAG_02360	0	1	0	1
CNAG_02359	chr6	522865	523486	621	null	small subunit ribosomal protein S25e	0	3	0	3
CNAG_02358	chr6	523602	525037	1435	null	MRP family ATP-binding protein	0	0	1	1
CNAG_02356	chr6	527902	528877	975	null	phosphatidylinositol glycan, class F	0	0	1	1
CNAG_02355	chr6	529165	531035	1870	null	solute carrier family 35 (UDP-xylose/UDP-N-acetylglucosamine transporter), member B4	1	0	1	2
CNAG_07634	chr6	537032	541797	4765	null	hypothetical protein CNAG_07634	1	2	1	4
CNAG_02350	chr6	546702	547637	935	null	hypothetical protein CNAG_02350	1	0	0	1
CNAG_02347	chr6	553316	553930	614	null	hypothetical protein CNAG_02347	0	1	0	1
CNAG_02343	chr6	559422	561981	2559	null	hypothetical protein CNAG_02343	0	1	0	1
CNAG_02342	chr6	562464	563854	1390	null	hypothetical protein CNAG_02342	0	1	0	1
CNAG_02340	chr6	569067	569772	705	null	hypothetical protein CNAG_02340	1	1	0	2
CNAG_02339	chr6	570155	571309	1154	null	hypothetical protein CNAG_02339	0	0	1	1
CNAG_02335	chr6	579635	581798	2163	null	hypothetical protein CNAG_02335	0	1	0	1
CNAG_02332	chr6	588707	590100	1393	null	hypothetical protein CNAG_02332	1	0	0	1
CNAG_02328	chr6	597696	600656	2960	null	pre-rRNA-processing protein TSR1	1	0	0	1
CNAG_02311	chr6	640609	642749	2140	null	hypothetical protein CNAG_02311	1	0	0	1
CNAG_02290	chr6	687540	689245	1705	null	parafibromin	0	1	0	1
CNAG_02285	chr6	700547	701486	939	null	nucleoside diphosphate kinase	0	1	0	1
CNAG_07641	chr6	724990	727183	2193	null	monosaccharide transporter	1	0	1	2
CNAG_07642	chr6	730825	732501	1676	null	hypothetical protein CNAG_07642	0	2	0	2
CNAG_02269	chr6	735423	737087	1664	null	autophagy-like protein 18 Atg18	0	2	0	2
CNAG_02268	chr6	737252	738786	1534	null	nuclear membrane protein	0	1	1	2
CNAG_02267	chr6	738936	740522	1586	null	cell cycle arrest protein BUB3	1	0	0	1
CNAG_07163	chr6	742938	744113	1175	null	preprotein translocase subunit YidC	0	2	0	2
CNAG_02260	chr6	763961	765531	1570	CRG1	protein FAM50	0	0	1	1
CNAG_02255	chr6	775676	776970	1294	null	BNR/Asp-box repeat family protein	1	0	0	1
CNAG_02240	chr6	821972	825033	3061	null	cytoplasmic protein	0	2	0	2
CNAG_02237	chr6	828703	832439	3736	SLA2	cytoskeleton assembly control protein	0	2	0	2
CNAG_02233	chr6	836740	844476	7736	null	serine/threonine-protein kinase ATR	0	0	1	1
CNAG_02231	chr6	846395	847564	1169	null	cytosolic Fe-S cluster assembly factor NBP35	1	0	0	1
CNAG_02228	chr6	857468	858291	823	null	hypothetical protein CNAG_02228	0	0	1	1
CNAG_02220	chr6	876475	879689	3214	null	hypothetical protein CNAG_02220	0	1	0	1
CNAG_02216	chr6	893411	899411	6000	null	hypothetical protein CNAG_02216	1	0	0	1

CNAG_02213	chr6	902038	903949	1911	null	epsin	1	0	0	1
CNAG_02210	chr6	909059	911304	2245	null	ribonuclease Z	0	1	0	1
CNAG_02206	chr6	921006	921355	349	null	hypothetical protein CNAG_02206	0	1	0	1
CNAG_02204	chr6	923322	924739	1417	null	endonuclease G, mitochondrial	1	1	0	2
CNAG_02202	chr6	928799	929659	860	null	adenylylsulfate kinase	0	2	0	2
CNAG_02196	chr6	937189	938733	1544	CDC11	septin	1	0	0	1
CNAG_02194	chr6	942579	945166	2587	null	AGC/NDR protein kinase	0	0	1	1
CNAG_02188	chr6	955708	956577	869	null	hypothetical protein CNAG_02188	0	1	0	1
CNAG_02175	chr6	991751	997012	5261	null	5\'-3\' exoribonuclease 1	0	1	1	2
CNAG_02174	chr6	997358	999617	2259	null	signal recognition particle protein SRP54	1	0	0	1
CNAG_02172	chr6	1001107	1007049	5942	null	ubiquitin-protein ligase	0	1	0	1
CNAG_02169	chr6	1013219	1014715	1496	null	hypothetical protein CNAG_02169	0	0	1	1
CNAG_02167	chr6	1021153	1023643	2490	null	vacuolar protein sorting-associated protein 27	0	1	0	1
CNAG_02165	chr6	1031181	1034260	3079	null	cytoplasmic protein	2	0	0	2
CNAG_02164	chr6	1034846	1038644	3798	null	hypothetical protein CNAG_02164	2	0	1	3
CNAG_02161	chr6	1041966	1046131	4165	null	cortical actin cytoskeleton protein asp1	0	1	0	1
CNAG_02159	chr6	1049803	1050555	752	null	hypothetical protein CNAG_02159	1	0	0	1
CNAG_02157	chr6	1056521	1058534	2013	null	hypothetical protein CNAG_02157	0	1	0	1
CNAG_02152	chr6	1070021	1071730	1709	null	hypothetical protein CNAG_02152	1	0	0	1
CNAG_02151	chr6	1071894	1075109	3215	null	amidohydrolase	1	0	0	1
CNAG_02150	chr6	1075494	1077201	1707	null	mRNA methyltransferase	0	3	0	3
CNAG_02148	chr6	1078083	1078972	889	null	ubiquitin-conjugating enzyme E2 35	0	1	0	1
CNAG_02144	chr6	1084033	1084871	838	null	large subunit ribosomal protein L1-A	0	1	0	1
CNAG_02139	chr6	1093211	1095369	2158	null	oxysterol-binding protein	0	1	0	1
CNAG_02138	chr6	1096639	1101563	4924	null	DNA replication ATP-dependent helicase Dna2	0	1	2	3
CNAG_02130	chr6	1117336	1119601	2265	NSR1	RNA-binding protein 39	0	1	0	1
CNAG_02123	chr6	1134460	1135883	1423	null	methionyl-tRNA formyltransferase	1	0	0	1
CNAG_02122	chr6	1136239	1137759	1520	null	cytoplasmic tRNA 2-thiolation protein 1	1	0	0	1
CNAG_02121	chr6	1138097	1140589	2492	null	hypothetical protein CNAG_02121	0	0	1	1
CNAG_02119	chr6	1142653	1144170	1517	null	hypothetical protein CNAG_02119	0	0	1	1
CNAG_02115	chr6	1150136	1151082	946	NHP6B02	nonhistone protein 6	0	0	1	1
CNAG_02114	chr6	1152058	1154094	2036	null	hypothetical protein CNAG_02114	0	0	1	1
CNAG_02111	chr6	1157050	1159241	2191	null	protein Clp1	0	1	0	1
CNAG_02108	chr6	1167621	1170002	2381	null	GTPase activating protein	0	1	0	1
CNAG_02106	chr6	1173017	1173905	888	null	mitochondrial intermembrane space import and assembly protein 40	1	0	0	1
CNAG_02103	chr6	1177308	1183318	6010	null	hypothetical protein CNAG_02103	1	1	0	2
CNAG_02102	chr6	1184299	1186432	2133	null	hypothetical protein CNAG_02102	1	0	0	1
CNAG_02101	chr6	1186689	1187357	668	null	hypothetical protein CNAG_02101	0	1	0	1
CNAG_02097	chr6	1205333	1206538	1205	null	hypothetical protein CNAG_02097	0	2	0	2
CNAG_02096	chr6	1206727	1208093	1366	null	peroxin-16	0	3	0	3
CNAG_02095	chr6	1210820	1212566	1746	null	cyclin	0	3	0	3
CNAG_02094	chr6	1213609	1214850	1241	null	arp2/3 complex 34 kda subunit	0	1	0	1
CNAG_02091	chr6	1221770	1223473	1703	null	nucleosome assembly protein 1-like 1	0	1	0	1
CNAG_02090	chr6	1223904	1225574	1670	GPA3	guanine nucleotide-binding protein G(o) subunit alpha	0	1	0	1
**CNAG_02089**	**chr6**	**1227443**	**1229691**	**2248**	**null**	**hypothetical protein CNAG_02089**	**0**	**27**	**0**	**27**
CNAG_02088	chr6	1230428	1231913	1485	null	hypothetical protein CNAG_02088	0	4	1	5
**CNAG_02087**	**chr6**	**1232584**	**1234447**	**1863**	**null**	**acyl-CoA-dependent ceramide synthase**	**2**	**12**	**0**	**14**

CNAG_02086	chr6	1235336	1237294	1958	null	acyl-CoA-dependent ceramide synthase	0	3	1	4
CNAG_02085	chr6	1237703	1239261	1558	HST302	hypothetical protein CNAG_02085	1	1	1	3
CNAG_02084	chr6	1240228	1241779	1551	ERG20	farnesyl diphosphate synthase	0	2	0	2
CNAG_02083	chr6	1242140	1244614	2474	SIT2	siderochrome-iron transporter	2	1	1	4
CNAG_02079	chr6	1254558	1258929	4371	null	cytoplasmic protein	0	1	0	1
CNAG_02078	chr6	1259240	1260650	1410	null	hydrolase	0	2	0	2
CNAG_02071	chr6	1273623	1276344	2721	BNI4	hypothetical protein CNAG_02071	1	0	0	1
CNAG_02069	chr6	1281767	1282557	790	null	hypothetical protein CNAG_02069	0	1	0	1
CNAG_02068	chr6	1282955	1284487	1532	null	stromal membrane-associated protein	0	1	0	1
CNAG_02064	chr6	1290929	1297060	6131	null	TATA-box-binding protein-associated factor	1	0	1	2
CNAG_02062	chr6	1302181	1305630	3449	null	glycoside hydrolase family 2	0	1	1	2
CNAG_02059	chr6	1310589	1311749	1160	null	CCR4-NOT complex subunit CAF16	0	1	0	1
CNAG_02056	chr6	1316930	1318339	1409	null	hypothetical protein CNAG_02056	1	0	1	2
CNAG_02053	chr6	1322542	1324133	1591	null	hypothetical protein CNAG_02053	1	0	0	1
CNAG_02052	chr6	1324193	1325679	1486	null	dimeric dihydrodiol dehydrogenase	1	1	0	2
CNAG_02051	chr6	1326116	1329840	3724	null	kinesin family member 20/23	0	1	0	1
CNAG_02050	chr6	1330219	1331731	1512	QDR1	hypothetical protein CNAG_02050	0	1	0	1
CNAG_02049	chr6	1332040	1334052	2012	PUT1	proline dehydrogenase	0	3	0	3
CNAG_02046	chr6	1340537	1343214	2677	null	poly(A)-binding protein binding protein	1	0	0	1
CNAG_02045	chr6	1343679	1346270	2591	null	acetoacetate-CoA ligase	1	0	0	1
CNAG_02043	chr6	1348248	1349048	800	null	hypothetical protein CNAG_02043	0	2	0	2
CNAG_02041	chr6	1355915	1357097	1182	null	hypothetical protein CNAG_02041	0	1	0	1
CNAG_02040	chr6	1357885	1359066	1181	null	hypothetical protein CNAG_02040	0	1	0	1
CNAG_02038	chr6	1361741	1364045	2304	null	hypothetical protein CNAG_02038	1	0	1	2
CNAG_02036	chr6	1365774	1367915	2141	CAS4	hypothetical protein CNAG_02036	0	0	2	2
CNAG_02035	chr6	1368392	1369347	955	null	triose-phosphate isomerase	0	2	0	2
CNAG_07650	chr6	1371941	1372556	615	null	hypothetical protein CNAG_07650	2	0	0	2
CNAG_02032	chr6	1372865	1378196	5331	null	nuclear export factor	2	0	0	2
CNAG_02031	chr6	1379008	1380210	1202	null	hypothetical protein CNAG_02031	1	0	2	3
CNAG_02030	chr6	1381644	1383944	2300	null	glyoxal oxidase	0	2	1	3
CNAG_02029	chr6	1386476	1388205	1729	WSP1	wiskott-Aldrich syndrome protein	0	1	0	1
CNAG_02028	chr6	1388990	1391323	2333	null	CMGC/SRPK protein kinase	0	1	0	1
CNAG_02027	chr6	1393560	1397159	3599	null	hypothetical protein CNAG_02027	0	5	0	5
CNAG_02026	chr6	1398846	1401885	3039	null	hypothetical protein CNAG_02026	1	2	0	3
CNAG_02025	chr6	1402084	1404075	1991	null	DNA-directed RNA polymerase III subunit RPC3	0	5	2	7
CNAG_02024	chr6	1404293	1408098	3805	null	hypothetical protein CNAG_02024	4	0	1	5
CNAG_02023	chr6	1408263	1409712	1449	null	hypothetical protein CNAG_02023	2	2	0	4
CNAG_02022	chr6	1410446	1411879	1433	null	DNA-directed RNA polymerase II subunit RPB3	0	3	0	3
CNAG_02021	chr6	1412176	1413126	950	null	N-terminal acetyltransferase A complex catalytic subunit Ard1	0	2	1	3
CNAG_02020	chr6	1413549	1415166	1617	null	SWR1-complex protein 4	0	0	3	3
**CNAG_07651**	**chr6**	**1416143**	**1418201**	**2058**	**null**	**DEAD-box ATP-dependent RNA helicase 26**	**3**	**5**	**5**	**13**
CNAG_06524	chr7	7512	10013	2501	FRE3	hypothetical protein CNAG_06524	1	0	0	1
CNAG_06525	chr7	10451	11473	1022	null	nuclear protein	0	3	0	3
CNAG_06526	chr7	12826	13962	1136	null	hypothetical protein CNAG_06526	1	0	0	1
CNAG_06530	chr7	22749	24234	1485	null	hypothetical protein CNAG_06530	0	1	0	1
CNAG_06532	chr7	29820	33104	3284	null	hypothetical protein CNAG_06532	2	2	0	4
CNAG_06533	chr7	33308	37622	4314	null	ATP-dependent permease	0	1	0	1

CNAG_06534	chr7	38815	42994	4179	HMG1	hydroxymethylglutaryl-CoA reductase (NADPH)	0	0	1	1
CNAG_06535	chr7	43601	44850	1249	null	ribosome biogenesis protein UTP30	0	1	0	1
CNAG_06537	chr7	47693	49459	1766	null	monocarboxylic acid transporter	1	0	0	1
CNAG_06543	chr7	66917	68608	1691	null	ethanolaminephosphotransferase	1	0	0	1
CNAG_06544	chr7	69231	69773	542	null	non-histone chromosomal protein 6	0	1	0	1
CNAG_06546	chr7	73794	75011	1217	null	hypothetical protein CNAG_06546	1	0	0	1
CNAG_06550	chr7	84042	90807	6765	null	nucleoporin family protein	1	0	1	2
CNAG_06551	chr7	91772	94746	2974	null	carnitine O-acetyltransferase	0	1	0	1
CNAG_06564	chr7	125971	132518	6547	null	clathrin-coated vesicle protein	1	0	0	1
CNAG_07659	chr7	151685	153133	1448	null	hypothetical protein CNAG_07659	1	0	0	1
CNAG_06576	chr7	170370	171300	930	CAR1	allergen	0	2	0	2
CNAG_07661	chr7	184497	187767	3270	null	hypothetical protein CNAG_07661	1	0	0	1
CNAG_06583	chr7	188287	189633	1346	null	hypothetical protein CNAG_06583	0	0	1	1
CNAG_06584	chr7	190339	192644	2305	null	hypothetical protein CNAG_06584	2	0	0	2
CNAG_06585	chr7	193893	200713	6820	null	pre-mRNA-splicing helicase BRR2	0	0	1	1
CNAG_06589	chr7	206413	206947	534	null	endoribonuclease L-PSP	0	1	0	1
CNAG_06591	chr7	211114	216010	4896	SET302	hypothetical protein CNAG_06591	1	1	0	2
CNAG_06592	chr7	217139	218312	1173	UBC7	ubiquitin-conjugating enzyme E2 G1	0	0	1	1
CNAG_06593	chr7	219909	223098	3189	null	rhamnogalacturonan lyase	0	2	0	2
CNAG_06598	chr7	232088	232418	330	null	hypothetical protein CNAG_06598	0	0	1	1
CNAG_06601	chr7	238389	241026	2637	null	amidohydrolase	1	0	0	1
CNAG_06602	chr7	242630	243388	758	null	cysteine-type peptidase	1	0	0	1
CNAG_06603	chr7	244426	248394	3968	null	di- and tripeptidase	0	2	0	2
CNAG_06604	chr7	248628	250652	2024	null	hypothetical protein CNAG_06604	1	0	1	2
CNAG_06611	chr7	273169	277266	4097	null	DNA-directed RNA polymerase I subunit RPA2	0	0	1	1
CNAG_06612	chr7	278844	279733	889	null	hypothetical protein CNAG_06612	0	1	0	1
CNAG_06616	chr7	287155	289139	1984	null	hypothetical protein CNAG_06616	0	0	1	1
CNAG_06625	chr7	310622	312218	1596	null	UBX domain-containing protein 1	0	2	0	2
CNAG_06632	chr7	329292	331664	2372	null	Atypical/ABC1 protein kinase	0	0	1	1
CNAG_06634	chr7	335697	337784	2087	null	DNA polymerase epsilon subunit B	1	0	0	1
CNAG_06635	chr7	338106	340607	2501	null	CCR4-NOT transcription complex subunit 3	0	1	0	1
CNAG_06637	chr7	344742	346981	2239	null	ubiquitin carboxyl-terminal hydrolase 22/27/51	0	2	0	2
CNAG_06639	chr7	351428	356649	5221	null	E3 ubiquitin-protein ligase MARCH6	1	0	1	2
CNAG_06642	chr7	361513	369953	8440	TOR1	Atypical/PIKK/FRAP protein kinase	1	0	0	1
CNAG_06644	chr7	372447	374319	1872	ERG5	C-22 sterol desaturase	0	1	0	1
CNAG_06648	chr7	382103	384126	2023	RTF1	RNA polymerase-associated protein RTF1	0	1	0	1
CNAG_06651	chr7	388229	389873	1644	null	amidohydrolase	0	1	0	1
CNAG_06652	chr7	390974	392935	1961	null	allantoate permease	0	1	0	1
CNAG_06655	chr7	395779	397255	1476	GPI8	phosphatidylinositol glycan, class K	0	1	0	1
CNAG_06662	chr7	413455	414792	1337	null	hypothetical protein CNAG_06662	0	1	0	1
CNAG_06664	chr7	416148	418388	2240	null	sorting nexin MVP1	1	1	0	2
CNAG_06665	chr7	418607	420533	1926	null	hypothetical protein CNAG_06665	1	0	0	1
CNAG_06667	chr7	425706	427378	1672	null	hypothetical protein CNAG_06667	0	2	0	2
CNAG_06671	chr7	432454	435814	3360	null	TKL protein kinase	1	0	0	1
CNAG_06678	chr7	456484	458965	2481	CSR1	enzyme activator	0	0	1	1
CNAG_07176	chr7	473916	474788	872	null	hypothetical protein CNAG_07176	0	0	1	1
CNAG_06683	chr7	475833	480998	5165	null	hypothetical protein CNAG_06683	2	0	0	2

CNAG_06684	chr7	481151	482722	1571	null	pre-rRNA-processing protein IPI3	1	0	0	1
CNAG_06686	chr7	486000	486904	904	null	hypothetical protein CNAG_06686	0	1	1	2
CNAG_06687	chr7	488136	493510	5374	null	hypothetical protein CNAG_06687	1	1	0	2
CNAG_06691	chr7	501719	505146	3427	null	tuftelin-interacting protein 11	0	0	1	1
CNAG_06692	chr7	505434	506231	797	null	glucosamine-phosphate N-acetyltransferase	0	1	0	1
CNAG_06695	chr7	509046	510157	1111	null	hypothetical protein CNAG_06695	0	1	0	1
CNAG_07990	chr7	517424	518220	796	null	hypothetical protein CNAG_07990	1	1	0	2
CNAG_06698	chr7	519709	520860	1151	null	phosphatase	0	2	1	3
CNAG_06699	chr7	523887	525564	1677	GPD	glyceraldehyde-3-phosphate dehydrogenase	0	3	0	3
CNAG_05670	chr7	568362	568806	444	null	hypothetical protein CNAG_05670	0	2	0	2
CNAG_05672	chr7	573257	574244	987	null	nucleolar protein	0	4	0	4
CNAG_05673	chr7	574855	577881	3026	null	GPI inositol-deacylase	1	0	0	1
CNAG_05674	chr7	579182	580982	1800	null	solute carrier family 35, member E1	0	1	0	1
CNAG_05675	chr7	581177	582945	1768	null	trimethyllysine dioxygenase	0	1	0	1
CNAG_07666	chr7	584587	586098	1511	null	hypothetical protein CNAG_07666	1	0	0	1
CNAG_05678	chr7	593667	594882	1215	null	membrane protein	0	0	1	1
CNAG_07667	chr7	597354	599921	2567	null	HAL protein kinase	1	0	0	1
CNAG_05682	chr7	603193	603860	667	null	hypothetical protein CNAG_05682	0	2	0	2
CNAG_05686	chr7	615474	615934	460	null	hypothetical protein CNAG_05686	0	1	0	1
CNAG_05687	chr7	616260	619670	3410	null	hypothetical protein CNAG_05687	2	0	0	2
CNAG_05691	chr7	624573	624951	378	null	hypothetical protein CNAG_05691	0	1	0	1
CNAG_05692	chr7	625711	627817	2106	null	sphingosine-1-phosphate phosphatase	0	0	1	1
CNAG_05695	chr7	633846	638113	4267	null	glucosamine 6-phosphate N-acetyltransferase	0	0	1	1
CNAG_05696	chr7	639246	640119	873	null	ubiquitin-conjugating enzyme E2-16 kDa	0	3	0	3
CNAG_05700	chr7	650268	651613	1345	null	endoplasmic reticulum protein	1	0	0	1
CNAG_05703	chr7	655253	660108	4855	LRG1	rho GTPase activator	0	0	1	1
CNAG_05704	chr7	661042	661869	827	null	ESCRT-II complex subunit VPS22	0	0	1	1
CNAG_05708	chr7	667396	670285	2889	UBP14	ubiquitin carboxyl-terminal hydrolase 5/13	0	1	1	2
CNAG_05712	chr7	672933	673705	772	RIB4	6,7-dimethyl-8-ribityllumazine synthase	0	2	1	3
CNAG_05713	chr7	674276	678720	4444	null	elongator complex protein 1	3	0	2	5
CNAG_05714	chr7	679034	680689	1655	null	hypothetical protein CNAG_05714	1	0	0	1
CNAG_05716	chr7	683711	684485	774	null	hypothetical protein CNAG_05716	0	1	0	1
CNAG_05717	chr7	685388	686566	1178	null	hypothetical protein CNAG_05717	0	2	0	2
CNAG_05720	chr7	691705	692726	1021	null	GTPase inhibitor	0	0	1	1
CNAG_05724	chr7	704603	706771	2168	null	hypothetical protein CNAG_05724	0	1	0	1
CNAG_05725	chr7	707080	708651	1571	null	ketol-acid reductoisomerase, mitochondrial	0	1	0	1
CNAG_05729	chr7	715960	716644	684	null	hypothetical protein CNAG_05729	1	0	0	1
CNAG_05731	chr7	718898	721243	2345	null	glyoxal oxidase	0	2	0	2
CNAG_05733	chr7	722655	724807	2152	null	cytoplasmic protein	0	1	0	1
CNAG_05735	chr7	729495	730022	527	null	hypothetical protein CNAG_05735	0	0	1	1
CNAG_05736	chr7	731399	732374	975	null	hypothetical protein CNAG_05736	0	1	0	1
CNAG_05737	chr7	733248	736105	2857	null	oligopeptide transporter 8	0	0	1	1
CNAG_05738	chr7	736757	738024	1267	null	hypothetical protein CNAG_05738	0	1	0	1
CNAG_05742	chr7	743828	745870	2042	STP1	hypothetical protein CNAG_05742	0	0	1	1
CNAG_05745	chr7	749042	752653	3611	null	cytoplasmic protein	1	0	0	1
CNAG_05746	chr7	752895	755614	2719	null	Swi5-dependent recombination DNA repair protein 1	0	1	0	1
CNAG_05755	chr7	781857	783274	1417	null	protein MAK16	0	1	0	1

CNAG_05756	chr7	783523	784442	919	null	mitochondrial protein	0	1	0	1
CNAG_05761	chr7	802205	802836	631	null	cyclin-dependent kinase regulatory subunit CKS1	0	1	0	1
CNAG_05762	chr7	803485	804044	559	null	large subunit acidic ribosomal protein P2	0	1	0	1
CNAG_05766	chr7	809669	812043	2374	SPR1	endoglucanase C	0	1	0	1
CNAG_05767	chr7	812478	815007	2529	null	hypothetical protein CNAG_05767	1	0	0	1
CNAG_05770	chr7	821806	823134	1328	null	proteasome component PRE2	1	0	0	1
CNAG_05771	chr7	823466	833154	9688	null	serine/threonine-protein kinase TEL1	1	0	0	1
CNAG_05772	chr7	833604	836038	2434	null	hypothetical protein CNAG_05772	1	1	1	3
CNAG_05775	chr7	841832	844293	2461	null	phosphatidylinositol glycan, class S	1	0	0	1
CNAG_05778	chr7	847554	849551	1997	null	WSC domain-containing protein	0	0	1	1
CNAG_07674	chr7	850946	852264	1318	null	hypothetical protein CNAG_07674	1	0	0	1
CNAG_05785	chr7	859860	862848	2988	STB4	hypothetical protein CNAG_05785	1	1	0	2
CNAG_05787	chr7	871732	875308	3576	null	symplekin	0	0	1	1
CNAG_05789	chr7	876449	877729	1280	null	hypothetical protein CNAG_05789	0	0	1	1
CNAG_05791	chr7	882360	885656	3296	ZDS3	hypothetical protein CNAG_05791	1	0	0	1
CNAG_05793	chr7	888123	889470	1347	null	hypothetical protein CNAG_05793	0	1	0	1
CNAG_05797	chr7	903154	906902	3748	XRN2	5\'-3\' exoribonuclease 2	0	1	0	1
CNAG_05799	chr7	908401	910178	1777	CDA1	chitin deacetylase	0	1	0	1
CNAG_05800	chr7	911724	912471	747	null	large subunit ribosomal protein L33-b	0	1	0	1
CNAG_05803	chr7	916011	919286	3275	null	exo-beta-1,3-glucanase	2	0	0	2
CNAG_05804	chr7	919627	925414	5787	null	nuclear pore complex protein Nup188	2	2	2	6
CNAG_05805	chr7	925703	926863	1160	null	hypothetical protein CNAG_05805	1	0	0	1
CNAG_05807	chr7	928057	929347	1290	null	hypothetical protein CNAG_05807	0	0	1	1
CNAG_05808	chr7	929388	930666	1278	null	syntaxin 18	0	1	0	1
CNAG_05811	chr7	934412	935515	1103	null	hypothetical protein CNAG_05811	0	0	1	1
CNAG_05814	chr7	942355	942904	549	null	small subunit ribosomal protein S10e	0	1	0	1
CNAG_05825	chr7	969721	972416	2695	null	minichromosome maintenance protein 7 (cell division control protein 47)	0	0	1	1
CNAG_05827	chr7	975550	977102	1552	null	methionine-tRNA ligase, beta subunit	0	0	2	2
CNAG_05828	chr7	977276	978941	1665	null	UDP-N-acetylglucosamine pyrophosphorylase	0	2	0	2
CNAG_05829	chr7	979312	983768	4456	null	hypothetical protein CNAG_05829	0	0	1	1
CNAG_05830	chr7	984455	988114	3659	null	cytoplasmic protein	2	0	0	2
CNAG_05833	chr7	991130	993265	2135	null	allantoate transporter	0	1	0	1
CNAG_05838	chr7	1008850	1011921	3071	RGD1	GTPase activating protein	2	0	0	2
CNAG_05839	chr7	1012211	1013052	841	null	cytochrome c oxidase subunit 6b	0	3	0	3
CNAG_05840	chr7	1013327	1014055	728	null	hypothetical protein CNAG_05840	0	4	0	4
CNAG_05841	chr7	1014259	1016232	1973	null	hypothetical protein CNAG_05841	3	0	2	5
CNAG_05843	chr7	1020297	1022266	1969	null	translation initiation factor 4E	1	0	0	1
CNAG_05844	chr7	1022656	1023225	569	null	mitochondrial import inner membrane translocase subunit TIM8	0	1	1	2
CNAG_05846	chr7	1024832	1028508	3676	null	ubiquitin-conjugating enzyme E2 O	0	1	2	3
CNAG_05847	chr7	1029581	1031043	1462	TRR1	thioredoxin reductase	0	6	0	6
CNAG_07677	chr7	1036998	1038200	1202	null	hypothetical protein CNAG_07677	4	1	1	6
CNAG_07679	chr7	1052726	1054356	1630	null	transcription elongation factor S-II	0	1	0	1
CNAG_05861	chr7	1059361	1061425	2064	null	hypothetical protein CNAG_05861	1	1	0	2
CNAG_05865	chr7	1073902	1074938	1036	null	20S proteasome subunit beta 6	0	3	0	3
CNAG_05866	chr7	1075539	1078958	3419	PRM1	plasma membrane fusion protein PRM1	2	1	2	5
CNAG_05867	chr7	1080074	1081875	1801	null	L-fucose transporter	1	0	6	7
CNAG_05868	chr7	1082041	1082606	565	null	glutamate-1-semialdehyde 2,1-aminomutase	0	10	0	10

CNAG_05869	chr7	1083606	1085485	1879	null	endopeptidase	0	0	3	3
CNAG_05871	chr7	1087104	1087827	723	null	hypothetical protein CNAG_05871	0	3	1	4
CNAG_05872	chr7	1088487	1090524	2037	null	endopeptidase	1	0	1	2
CNAG_05873	chr7	1091016	1092272	1256	null	hypothetical protein CNAG_05873	0	1	0	1
CNAG_05874	chr7	1093169	1095374	2205	null	hypothetical protein CNAG_05874	1	0	1	2
CNAG_05875	chr7	1095759	1096668	909	null	cytochrome c heme-lyase	1	1	0	2
CNAG_05877	chr7	1097734	1099068	1334	null	CDK-activating kinase assembly factor MAT1	1	0	2	3
CNAG_05879	chr7	1101513	1101836	323	null	hypothetical protein CNAG_05879	0	0	1	1
CNAG_07681	chr7	1109684	1111203	1519	null	hypothetical protein CNAG_07681	0	0	1	1
CNAG_05885	chr7	1116116	1118204	2088	null	hypothetical protein CNAG_05885	0	1	0	1
CNAG_05886	chr7	1118971	1119814	843	MMS2	ubiquitin-conjugating enzyme E2	0	2	0	2
CNAG_05887	chr7	1120067	1121372	1305	null	pre-mRNA-splicing factor 18	0	3	0	3
CNAG_05888	chr7	1121481	1121995	514	null	hypothetical protein CNAG_05888	2	2	0	4
CNAG_05894	chr7	1132927	1148016	15089	null	dynein heavy chain 1, cytosolic	0	1	3	4
**CNAG_05903**	**chr7**	**1170289**	**1171602**	**1313**	**null**	**hypothetical protein CNAG_05903**	**2**	**15**	**2**	**19**
CNAG_05905	chr7	1172992	1175444	2452	null	transcription factor IIIB 90 kDa subunit	0	0	1	1
CNAG_05906	chr7	1175883	1177139	1256	null	hypothetical protein CNAG_05906	1	0	0	1
CNAG_05908	chr7	1182581	1185172	2591	null	hypothetical protein CNAG_05908	1	1	0	2
CNAG_05909	chr7	1187122	1188390	1268	null	cytochrome c1, heme protein, mitochondrial	0	1	0	1
**CNAG_05910**	**chr7**	**1189418**	**1193699**	**4281**	**null**	**hypothetical protein CNAG_05910**	**3**	**4**	**5**	**12**
CNAG_05911	chr7	1194262	1195759	1497	null	hypothetical protein CNAG_05911	6	0	1	7
CNAG_05912	chr7	1196368	1199805	3437	null	hypothetical protein CNAG_05912	0	1	0	1
CNAG_05913	chr7	1200590	1202647	2057	null	alpha-glucosidase	0	1	0	1
CNAG_05917	chr7	1210607	1211256	649	null	hypothetical protein CNAG_05917	1	0	0	1
CNAG_07682	chr7	1222798	1227884	5086	null	hypothetical protein CNAG_07682	0	1	0	1
CNAG_05933	chr7	1250203	1252717	2514	null	hypothetical protein CNAG_05933	0	4	1	5
CNAG_05934	chr7	1253654	1254935	1281	LIV15	hypothetical protein CNAG_05934	0	2	0	2
CNAG_05936	chr7	1256635	1261872	5237	null	DNA-directed RNA polymerase III subunit RPC1	1	0	0	1
CNAG_05939	chr7	1266928	1267252	324	null	hypothetical protein CNAG_05939	0	1	0	1
CNAG_07683	chr7	1280016	1281080	1064	null	hypothetical protein CNAG_07683	0	3	0	3
CNAG_07685	chr7	1284516	1286566	2050	null	UMF1 family MFS transporter	0	2	0	2
CNAG_07686	chr7	1287338	1291248	3910	null	topoisomerase 1-associated factor 1	0	0	1	1
CNAG_07689	chr7	1298002	1300752	2750	null	pre-mRNA-splicing factor CWC22	0	1	0	1
CNAG_07690	chr7	1302289	1303065	776	null	hypothetical protein CNAG_07690	0	1	0	1
CNAG_07692	chr7	1306836	1309960	3124	null	nucleoside phosphatase	0	0	1	1
CNAG_07188	chr7	1310169	1315979	5810	null	hypothetical protein CNAG_07188	4	1	1	6
CNAG_07693	chr7	1317832	1320188	2356	null	high-affinity methionine permease	3	1	0	4
CNAG_07695	chr7	1323585	1325409	1824	null	gamma-aminobutyric acid transporter	0	0	1	1
CNAG_07699	chr7	1329889	1335387	5498	null	zinc finger family protein	1	1	2	4
CNAG_05962	chr7	1343055	1344680	1625	null	hypothetical protein CNAG_05962	0	1	0	1
CNAG_05963	chr7	1345536	1347838	2302	null	hypothetical protein CNAG_05963	0	1	1	2
CNAG_05964	chr7	1347971	1349200	1229	null	poly(3-hydroxybutyrate) depolymerase	0	2	0	2
CNAG_05967	chr7	1353539	1354646	1107	null	hypothetical protein CNAG_05967	1	2	0	3
CNAG_05968	chr7	1355129	1355895	766	CDC420	cell division control protein 42	0	1	0	1
CNAG_05978	chr7	1385416	1388045	2629	null	glutamate-tRNA ligase	0	0	1	1
CNAG_05980	chr7	1391518	1392789	1271	null	large subunit ribosomal protein L7/L12	0	1	0	1
CNAG_06944	chr8	3855	6312	2457	null	hypothetical protein CNAG_06944	2	0	0	2

CNAG_07706	chr8	10046	12151	2105	null	hypothetical protein CNAG_07706	0	0	1	1
CNAG_07707	chr8	21436	23958	2522	null	glycoside hydrolase family 3 domain-containing protein	1	0	0	1
CNAG_03090	chr8	24519	26505	1986	null	hypothetical protein CNAG_03090	0	1	0	1
CNAG_03091	chr8	28052	29426	1374	null	hypothetical protein CNAG_03091	0	1	0	1
CNAG_03101	chr8	54135	56107	1972	null	efflux protein EncT	0	1	0	1
CNAG_03102	chr8	57102	58433	1331	null	hypothetical protein CNAG_03102	0	1	1	2
CNAG_03103	chr8	58978	61734	2756	null	Cullin 3	1	0	0	1
CNAG_03108	chr8	68241	70175	1934	null	hypothetical protein CNAG_03108	1	0	0	1
CNAG_03109	chr8	70428	72440	2012	null	ubiquitin carboxyl-terminal hydrolase 14	0	0	1	1
CNAG_03110	chr8	73240	76533	3293	null	hypothetical protein CNAG_03110	2	0	1	3
CNAG_07994	chr8	77398	80100	2702	null	hypothetical protein CNAG_07994	1	0	0	1
CNAG_03115	chr8	85097	87498	2401	null	hypothetical protein CNAG_03115	0	0	2	2
CNAG_07711	chr8	87540	88112	572	null	hypothetical protein CNAG_07711	0	1	0	1
CNAG_03117	chr8	97494	102289	4795	null	hypothetical protein CNAG_03117	2	1	0	3
CNAG_03120	chr8	106153	114132	7979	AGS1	alpha-1,3-glucan synthase	1	0	0	1
CNAG_03121	chr8	115374	120079	4705	null	hypothetical protein CNAG_03121	0	0	1	1
CNAG_03123	chr8	123256	123862	606	null	hypothetical protein CNAG_03123	1	1	0	2
CNAG_03125	chr8	127699	129946	2247	null	hypothetical protein CNAG_03125	0	0	1	1
CNAG_03135	chr8	151598	154074	2476	null	hypothetical protein CNAG_03135	0	0	1	1
CNAG_03140	chr8	168779	170954	2175	null	sugar transporter	0	0	1	1
CNAG_03141	chr8	171633	172358	725	null	hypothetical protein CNAG_03141	1	0	0	1
CNAG_03150	chr8	192767	194028	1261	null	solute carrier family 25 (mitochondrial folate transporter), member 32	0	0	1	1
CNAG_03151	chr8	194368	195863	1495	CSN4	COP9 signalosome complex subunit 4	1	1	0	2
CNAG_03153	chr8	198229	199434	1205	SEC14-1	sec14 cytosolic factor	0	2	0	2
CNAG_03154	chr8	200430	201202	772	null	hypothetical protein CNAG_03154	1	0	0	1
CNAG_03160	chr8	214743	217680	2937	null	DNA cross-link repair 1A protein	0	0	1	1
CNAG_03168	chr8	233647	237026	3379	null	sulfite reductase (NADPH) flavoprotein alpha-component	0	1	0	1
CNAG_03171	chr8	241906	245469	3563	SWE1	Wee protein kinase	0	1	0	1
CNAG_03172	chr8	246420	247400	980	null	hypothetical protein CNAG_03172	1	0	0	1
CNAG_03188	chr8	290291	293439	3148	SET202	histone-lysine N-methyltransferase, H3 lysine-36 specific	0	0	2	2
CNAG_03189	chr8	293836	297624	3788	null	DIL and ankyrin domain-containing protein	1	1	0	2
CNAG_03190	chr8	299574	301917	2343	CCR4	glucose-repressible alcohol dehydrogenase transcriptional effector	0	0	1	1
CNAG_03193	chr8	308381	310241	1860	null	hypothetical protein CNAG_03193	2	1	0	3
CNAG_03194	chr8	311449	313101	1652	null	saccharopine dehydrogenase	0	0	1	1
CNAG_07717	chr8	325159	329777	4618	null	ubiquitin carboxyl-terminal hydrolase 4/11/15	1	0	0	1
CNAG_03202	chr8	330152	337452	7300	CAC1	adenylate cyclase	2	0	1	3
CNAG_03205	chr8	341766	343799	2033	null	ATP-dependent RNA helicase ROK1	0	1	0	1
CNAG_03206	chr8	343992	345512	1520	null	endonuclease III	0	0	1	1
CNAG_03211	chr8	354567	356070	1503	null	hypothetical protein CNAG_03211	0	1	0	1
CNAG_03216	chr8	367676	370405	2729	null	CAMK protein kinase	0	1	0	1
CNAG_03217	chr8	370734	373800	3066	null	hypothetical protein CNAG_03217	0	1	0	1
CNAG_03221	chr8	380123	380677	554	null	large subunit ribosomal protein L29	0	3	0	3
CNAG_03222	chr8	381032	383181	2149	null	RNA lariat debranching enzyme	0	1	0	1
CNAG_03225	chr8	387416	388886	1470	null	malate dehydrogenase, NAD-dependent	0	1	0	1
CNAG_03227	chr8	392333	393371	1038	null	hypothetical protein CNAG_03227	0	3	1	4
**CNAG_03229**	**chr8**	**395211**	**396697**	**1486**	**YOX101**	**specific transcriptional repressor**	**4**	**7**	**3**	**14**
CNAG_03230	chr8	398101	398745	644	null	hypothetical protein CNAG_03230	2	8	0	10

CNAG_03231	chr8	400885	401562	677	null	hypothetical protein CNAG_03231	2	7	0	9
CNAG_03232	chr8	402125	403325	1200	null	lactamase	0	1	0	1
CNAG_03233	chr8	404581	405238	657	null	hypothetical protein CNAG_03233	0	2	0	2
CNAG_03234	chr8	406178	409470	3292	null	cell wall surface anchor protein	4	1	2	7
CNAG_03235	chr8	409652	412413	2761	null	THO complex subunit 1	1	3	1	5
CNAG_03238	chr8	415069	416267	1198	null	dioxygenase subfamily protein	0	1	1	2
CNAG_03239	chr8	416572	417849	1277	null	hypothetical protein CNAG_03239	1	0	0	1
CNAG_03240	chr8	419691	422563	2872	null	mannosyl-oligosaccharide 1,2-alpha-mannosidase	2	1	0	3
CNAG_03247	chr8	439081	443496	4415	null	sterol 3beta-glucosyltransferase	0	1	0	1
CNAG_03258	chr8	505898	508168	2270	null	AGC protein kinase	0	2	1	3
CNAG_03259	chr8	509641	510069	428	null	hypothetical protein CNAG_03259	0	2	0	2
CNAG_03263	chr8	514889	516569	1680	TUF1	translation elongation factor Tu	0	0	1	1
CNAG_03264	chr8	516608	517725	1117	null	hypothetical protein CNAG_03264	0	1	0	1
CNAG_03265	chr8	517990	520956	2966	null	hypothetical protein CNAG_03265	2	0	0	2
CNAG_03266	chr8	522651	524285	1634	null	malate dehydrogenase, NAD-dependent	0	2	0	2
CNAG_03268	chr8	527992	528454	462	null	hypothetical protein CNAG_03268	0	1	0	1
CNAG_03271	chr8	534448	536353	1905	null	pre-mRNA-splicing factor PRP46	2	0	0	2
CNAG_03272	chr8	537344	538843	1499	null	hypothetical protein CNAG_03272	2	2	0	4
CNAG_03275	chr8	546580	548524	1944	null	glutaryl-CoA dehydrogenase	0	1	1	2
CNAG_03276	chr8	548842	550326	1484	null	hypothetical protein CNAG_03276	1	0	2	3
CNAG_03279	chr8	554569	557313	2744	null	hypothetical protein CNAG_03279	0	0	1	1
CNAG_03282	chr8	561452	562836	1384	null	hypothetical protein CNAG_03282	0	0	1	1
CNAG_03284	chr8	564540	565967	1427	null	rRNA biogenesis protein RRP36	1	1	0	2
CNAG_03285	chr8	566262	568067	1805	null	hypothetical protein CNAG_03285	1	0	0	1
CNAG_03289	chr8	573672	575516	1844	null	methionine aminopeptidase, type II	1	0	0	1
CNAG_03290	chr8	575740	577890	2150	null	STE/STE20/YSK protein kinase	0	1	0	1
CNAG_03292	chr8	582931	583510	579	null	hypothetical protein CNAG_03292	0	5	0	5
CNAG_03295	chr8	589904	592678	2774	null	hypothetical protein CNAG_03295	0	0	1	1
CNAG_03299	chr8	597594	601029	3435	null	coatomer beta subunit	0	1	3	4
CNAG_03304	chr8	607814	609410	1596	null	hypothetical protein CNAG_03304	1	0	0	1
CNAG_03308	chr8	615164	618483	3319	null	hypothetical protein CNAG_03308	0	2	1	3
CNAG_03311	chr8	622927	624758	1831	ERG13	hydroxymethylglutaryl-CoA synthase	1	1	0	2
CNAG_03313	chr8	626487	628335	1848	null	hypothetical protein CNAG_03313	0	0	2	2
CNAG_03314	chr8	628784	631700	2916	null	YTH domain family 2	0	0	1	1
CNAG_03319	chr8	638163	641582	3419	null	phospholipid binding protein	1	0	0	1
CNAG_03327	chr8	659488	660291	803	null	hypothetical protein CNAG_03327	1	0	0	1
CNAG_03329	chr8	662943	664763	1820	null	PHD-finger protein	1	0	0	1
CNAG_03330	chr8	664959	666636	1677	null	RecQ-mediated genome instability protein 1	1	0	0	1
CNAG_03332	chr8	668775	670081	1306	null	replication factor C subunit 2/4	0	2	0	2
CNAG_03333	chr8	670648	673560	2912	null	cytoplasmic protein	0	1	0	1
CNAG_03337	chr8	682367	682978	611	null	hypothetical protein CNAG_03337	0	2	0	2
CNAG_03339	chr8	684431	686559	2128	null	biotin transporter	2	0	0	2
CNAG_03341	chr8	689458	692510	3052	null	minichromosome maintenance protein 2	1	1	0	2
CNAG_03343	chr8	694814	695857	1043	null	hypothetical protein CNAG_03343	0	1	0	1
CNAG_03347	chr8	711146	713803	2657	null	ATP-dependent Clp protease ATP-binding subunit ClpB	1	2	0	3
CNAG_03348	chr8	714434	717017	2583	null	hypothetical protein CNAG_03348	1	1	0	2
CNAG_03349	chr8	719057	721284	2227	null	pre-mRNA-processing factor 17	1	0	0	1

CNAG_07724	chr8	721729	725020	3291	CUF1	ligand-regulated transcription factor	0	1	0	1
CNAG_03354	chr8	728169	729027	858	null	hypothetical protein CNAG_03354	0	1	0	1
CNAG_03355	chr8	730393	738152	7759	TCO4	hypothetical protein CNAG_03355	2	0	1	3
CNAG_03356	chr8	738298	739608	1310	null	hypothetical protein CNAG_03356	1	0	0	1
CNAG_03358	chr8	742230	743796	1566	null	phosphoglycerate kinase	0	0	1	1
CNAG_03360	chr8	745090	746790	1700	null	hypothetical protein CNAG_03360	0	0	1	1
CNAG_03361	chr8	747084	748851	1767	null	hypothetical protein CNAG_03361	0	1	0	1
CNAG_03362	chr8	748963	750774	1811	null	hypothetical protein CNAG_03362	0	1	0	1
CNAG_03376	chr8	791569	794219	2650	null	cytoplasmic protein	0	1	0	1
CNAG_03392	chr8	832600	836769	4169	null	DNA supercoiling protein	0	1	0	1
CNAG_07728	chr8	847574	847915	341	null	solute carrier family 39 (zinc transporter), member 1/2/3	0	1	0	1
CNAG_03405	chr8	868668	870041	1373	null	hypothetical protein CNAG_03405	0	1	0	1
CNAG_03418	chr8	898941	903062	4121	null	exportin-1	1	1	0	2
CNAG_03425	chr8	919285	922373	3088	null	hypothetical protein CNAG_03425	0	0	2	2
CNAG_03427	chr8	925140	928307	3167	ACO1	aconitate hydratase, mitochondrial	2	0	0	2
CNAG_07730	chr8	928815	930002	1187	null	tricarboxylate transporter	0	0	1	1
CNAG_07732	chr8	931383	932392	1009	null	amidohydrolase 2	1	0	0	1
CNAG_03432	chr8	936093	937966	1873	null	solute carrier family 2	0	0	1	1
CNAG_03433	chr8	938300	939781	1481	null	endo alpha-1,4 polygalactosaminidase precursor	1	0	0	1
CNAG_03434	chr8	940970	943865	2895	null	solute carrier family 45, member 1/2/4	1	2	0	3
CNAG_03436	chr8	945397	947174	1777	null	alanine transaminase	0	2	0	2
CNAG_03439	chr8	955545	956395	850	null	hypothetical protein CNAG_03439	1	0	1	2
CNAG_03441	chr8	958811	959462	651	null	small subunit ribosomal protein S21	0	1	0	1
CNAG_03443	chr8	962315	962966	651	null	hypothetical protein CNAG_03443	0	1	1	2
CNAG_03444	chr8	963952	964607	655	null	hypothetical protein CNAG_03444	0	2	0	2
CNAG_03450	chr8	975965	981943	5978	null	ATP-binding cassette transporter	1	0	1	2
CNAG_03451	chr8	982238	985215	2977	null	hypothetical protein CNAG_03451	1	1	1	3
CNAG_03453	chr8	987848	991728	3880	KIP1	kinesin family member 11	2	0	0	2
CNAG_03454	chr8	992122	993410	1288	null	hypothetical protein CNAG_03454	0	2	0	2
CNAG_03455	chr8	996349	997313	964	null	hypothetical protein CNAG_03455	1	1	0	2
CNAG_03456	chr8	997366	998460	1094	null	hypothetical protein CNAG_03456	0	1	0	1
CNAG_03458	chr8	1001656	1002612	956	null	hypothetical protein CNAG_03458	0	1	1	2
CNAG_03460	chr8	1004986	1006248	1262	null	phosphoglycerate dehydrogenase	0	1	0	1
CNAG_03461	chr8	1007047	1008383	1336	null	hypothetical protein CNAG_03461	0	4	0	4
CNAG_03464	chr8	1013755	1016444	2689	LAC2	Cu-oxidase	2	0	0	2
CNAG_03465	chr8	1021759	1024340	2581	LAC1	laccase	1	1	0	2
CNAG_03466	chr8	1025186	1029851	4665	RDP1	RNA-dependent RNA polymerase 1	1	0	0	1
CNAG_03467	chr8	1030527	1031561	1034	null	hypothetical protein CNAG_03467	1	2	0	3
CNAG_03468	chr8	1032357	1035151	2794	null	hypothetical protein CNAG_03468	0	1	0	1
CNAG_07736	chr8	1038430	1041090	2660	null	glucan endo-1,3-alpha-glucosidase agn1	0	1	0	1
CNAG_07738	chr8	1047236	1047663	427	null	hypothetical protein CNAG_07738	1	0	0	1
CNAG_03473	chr8	1048240	1049365	1125	null	lipoyl(octanoyl) transferase	0	2	0	2
CNAG_03474	chr8	1049445	1051172	1727	null	efflux protein EncT	0	0	1	1
CNAG_03475	chr8	1052386	1055172	2786	null	hypothetical protein CNAG_03475	0	1	1	2
CNAG_03480	chr8	1071785	1075494	3709	null	hypothetical protein CNAG_03480	1	1	0	2
CNAG_03490	chr8	1095028	1096579	1551	null	hypothetical protein CNAG_03490	1	0	0	1
CNAG_03494	chr8	1102723	1104435	1712	null	hypothetical protein CNAG_03494	1	0	0	1

CNAG_03498	chr8	1111329	1113683	2354	FRE201	metalloreductase	0	1	0	1
CNAG_03500	chr8	1117626	1123693	6067	null	osmosensor	0	1	0	1
CNAG_03502	chr8	1125071	1127981	2910	ALR2	magnesium transporter	0	1	0	1
CNAG_03503	chr8	1128340	1133301	4961	YOR1	ATP-binding cassette transporter protein YOR1	0	2	0	2
CNAG_03504	chr8	1136047	1141720	5673	null	hypothetical protein CNAG_03504	0	2	0	2
CNAG_07739	chr8	1142109	1145250	3141	null	hypothetical protein CNAG_07739	0	1	1	2
CNAG_03509	chr8	1148243	1149598	1355	null	pyruvate dehydrogenase X component	1	0	0	1
CNAG_03510	chr8	1150713	1151316	603	null	large subunit ribosomal protein L36e	0	1	0	1
CNAG_03511	chr8	1151532	1152112	580	null	hypothetical protein CNAG_03511	0	2	0	2
CNAG_03512	chr8	1152471	1153714	1243	null	hypothetical protein CNAG_03512	0	1	1	2
CNAG_03513	chr8	1153833	1155091	1258	null	ribosomal RNA-processing protein 7	1	0	0	1
CNAG_07740	chr8	1155341	1156547	1206	null	hypothetical protein CNAG_07740	0	0	1	1
CNAG_03524	chr8	1180709	1186594	5885	null	transmembrane receptor	0	3	0	3
CNAG_03528	chr8	1198427	1202221	3794	null	AP-2 complex subunit alpha	0	1	0	1
CNAG_07995	chr8	1202853	1205833	2980	null	hypothetical protein CNAG_07995	0	5	1	6
CNAG_03531	chr8	1206371	1207397	1026	null	3-beta hydroxysteroid dehydrogenase/isomerase	2	0	1	3
CNAG_03532	chr8	1207965	1209003	1038	null	3-beta hydroxysteroid dehydrogenase/isomerase	1	0	0	1
CNAG_03533	chr8	1209375	1212622	3247	null	JmjC domain-containing histone demethylation protein 1	1	0	1	2
CNAG_03539	chr8	1226784	1229272	2488	null	hypothetical protein CNAG_03539	1	0	0	1
CNAG_03543	chr8	1238425	1241661	3236	null	hypothetical protein CNAG_03543	1	0	1	2
CNAG_03544	chr8	1242305	1243254	949	null	hypothetical protein CNAG_03544	0	1	0	1
CNAG_03547	chr8	1250273	1251079	806	null	hypothetical protein CNAG_03547	1	1	1	3
CNAG_03551	chr8	1255993	1256390	397	null	hypothetical protein CNAG_03551	0	1	0	1
CNAG_03553	chr8	1259499	1263316	3817	null	hypothetical protein CNAG_03553	1	0	0	1
CNAG_03556	chr8	1269763	1271921	2158	null	dimethylaniline monooxygenase	0	0	1	1
CNAG_03562	chr8	1282101	1283435	1334	null	D-amino-acid oxidase	0	1	0	1
CNAG_03565	chr8	1289492	1293714	4222	null	plasma-membrane proton-efflux P-type ATPase	0	0	1	1
CNAG_03572	chr8	1304898	1306114	1216	OPS1	opsin 1	1	0	0	1
CNAG_07748	chr8	1321708	1323092	1384	null	tRNA (uracil-5-)-methyltransferase TRM9	0	0	1	1
CNAG_07749	chr8	1323275	1324445	1170	null	3-ketodihydrosphingosine reductase TSC10	0	0	1	1
CNAG_03582	chr8	1326882	1330110	3228	RIM20	pH-response regulator protein palA/RIM20	0	0	1	1
CNAG_03586	chr8	1339453	1341097	1644	null	hypothetical protein CNAG_03586	1	1	0	2
CNAG_03592	chr8	1357570	1359560	1990	null	phosphomethylpyrimidine kinase	0	1	0	1
CNAG_07751	chr8	1359932	1361927	1995	null	siderophore iron transporter MirB	0	1	0	1
CNAG_06976	chr8	1364624	1366333	1709	FRE6	ferric-chelate reductase	1	0	0	1
CNAG_06977	chr8	1366722	1368262	1540	null	L-iditol 2-dehydrogenase	0	0	1	1
CNAG_06993	chr8	1370189	1371594	1405	null	hypothetical protein CNAG_06993	0	1	0	1
CNAG_04103	chr9	11549	13742	2193	null	hypothetical protein CNAG_04103	0	2	0	2
CNAG_04108	chr9	24023	26178	2155	null	pyruvate dehydrogenase kinase	0	1	0	1
CNAG_04109	chr9	26984	28599	1615	null	hypothetical protein CNAG_04109	0	2	0	2
CNAG_07756	chr9	42531	44264	1733	null	cell division control protein	0	2	0	2
CNAG_04117	chr9	49919	52595	2676	null	hypothetical protein CNAG_04117	1	0	0	1
CNAG_04118	chr9	53393	57012	3619	null	CMGC/CDK/CRK7 protein kinase	1	0	0	1
CNAG_04121	chr9	68507	68902	395	null	hypothetical protein CNAG_04121	0	1	0	1
CNAG_04130	chr9	83556	85406	1850	null	hypothetical protein CNAG_04130	0	1	0	1
CNAG_07757	chr9	96422	100522	4100	null	hypothetical protein CNAG_07757	2	0	0	2
CNAG_04136	chr9	101022	102468	1446	null	methylthioribose-1-phosphate isomerase	1	1	0	2

CNAG_04137	chr9	102604	106242	3638	null	hypothetical protein CNAG_04137	0	1	0	1
CNAG_04140	chr9	111607	112000	393	null	hypothetical protein CNAG_04140	1	0	0	1
CNAG_04142	chr9	114921	116957	2036	null	tartrate transporter	0	2	0	2
CNAG_04148	chr9	131882	134549	2667	null	NEK protein kinase	1	0	0	1
CNAG_04155	chr9	151480	152016	536	null	small nuclear ribonucleoprotein E	0	1	0	1
CNAG_04161	chr9	164272	166628	2356	null	hypothetical protein CNAG_04161	0	2	0	2
CNAG_04165	chr9	177374	178387	1013	null	hypothetical protein CNAG_04165	0	1	0	1
CNAG_04167	chr9	179873	181591	1718	null	hypothetical protein CNAG_04167	0	1	0	1
CNAG_04169	chr9	184842	185422	580	null	holo	0	1	1	2
CNAG_04172	chr9	190879	194016	3137	null	transcription factor C subunit 6	0	1	0	1
CNAG_04175	chr9	200310	201775	1465	null	hypothetical protein CNAG_04175	1	0	1	2
CNAG_04179	chr9	215113	217253	2140	null	lysine-tRNA ligase	1	1	0	2
CNAG_04180	chr9	217459	218392	933	null	large subunit ribosomal protein L15	1	0	0	1
CNAG_04183	chr9	223117	226359	3242	null	hypothetical protein CNAG_04183	1	0	0	1
CNAG_04186	chr9	234218	235035	817	null	hypothetical protein CNAG_04186	0	0	1	1
CNAG_04187	chr9	235655	237615	1960	null	GPI-anchored wall transfer protein 1	2	0	0	2
CNAG_04189	chr9	240206	242875	2669	null	succinate dehydrogenase	0	1	2	3
CNAG_04192	chr9	251743	255810	4067	null	phosphoribosylformylglycinamidine synthase	0	1	1	2
CNAG_04193	chr9	256388	257013	625	null	hypothetical protein CNAG_04193	0	1	0	1
CNAG_04197	chr9	264161	268128	3967	null	CMGC/DYRK/YAK protein kinase	0	0	1	1
CNAG_04204	chr9	285709	286208	499	null	transcription elongation factor SPT4	0	1	0	1
CNAG_04206	chr9	289082	290029	947	null	hypothetical protein CNAG_04206	0	1	0	1
CNAG_07762	chr9	291486	292074	588	null	hypothetical protein CNAG_07762	0	1	0	1
CNAG_04207	chr9	292512	294915	2403	null	homeobox protein cut-like	1	0	0	1
CNAG_04210	chr9	300460	302602	2142	null	MFS transporter, SP family, general alpha glucoside:H symporter	0	1	0	1
CNAG_04211	chr9	303764	306281	2517	null	nuclear RNA export factor 1/2	0	0	1	1
CNAG_04212	chr9	306503	307176	673	null	hypothetical protein CNAG_04212	0	2	0	2
CNAG_04213	chr9	307694	311942	4248	null	signal transducer	0	1	0	1
CNAG_04216	chr9	317698	318435	737	null	hypothetical protein CNAG_04216	1	0	0	1
CNAG_04219	chr9	325054	325921	867	null	lactoylglutathione lyase	0	1	0	1
CNAG_04221	chr9	329117	331282	2165	null	6-phosphofructo-2-kinase/fructose-2,6-bisphosphatase	0	0	1	1
CNAG_04225	chr9	337912	339915	2003	null	hypothetical protein CNAG_04225	2	0	1	3
CNAG_04226	chr9	340643	342933	2290	null	U4/U6.U5 tri-snRNP-associated protein 1	1	0	0	1
CNAG_04227	chr9	343072	349137	6065	null	hypothetical protein CNAG_04227	1	3	1	5
CNAG_04229	chr9	352155	353322	1167	null	phytanoyl-CoA dioxygenase	1	0	2	3
CNAG_04232	chr9	359257	359852	595	null	hypothetical protein CNAG_04232	0	1	0	1
CNAG_04234	chr9	362164	363148	984	null	hypothetical protein CNAG_04234	0	2	0	2
CNAG_04235	chr9	363351	364042	691	null	hypothetical protein CNAG_04235	1	0	0	1
CNAG_04238	chr9	366455	367659	1204	null	peroxisomal 2,4-dienoyl-CoA reductase	0	0	1	1
CNAG_04241	chr9	371053	378492	7439	null	cytoplasmic protein	1	2	2	5
CNAG_04242	chr9	378885	381129	2244	null	hypothetical protein CNAG_04242	0	1	0	1
CNAG_04243	chr9	384843	388564	3721	CDC24	cell division control protein 24	0	2	0	2
CNAG_04244	chr9	389287	392475	3188	null	hypothetical protein CNAG_04244	1	1	1	3
CNAG_04245	chr9	394168	396817	2649	CHI22	chitinase	2	1	0	3
CNAG_04246	chr9	397311	398390	1079	null	hypothetical protein CNAG_04246	2	0	1	3
CNAG_04247	chr9	398485	402375	3890	null	hypothetical protein CNAG_04247	1	0	0	1
CNAG_04252	chr9	405726	408471	2745	null	hypothetical protein CNAG_04252	1	0	0	1

CNAG_04254	chr9	411118	413164	2046	null	hypothetical protein CNAG_04254	1	0	0	1
CNAG_04258	chr9	418358	419010	652	ARF1	arf/Sar family protein	0	0	1	1
CNAG_04262	chr9	424198	426565	2367	null	E3 ubiquitin-protein ligase NRDP1	0	0	2	2
CNAG_04263	chr9	427114	429540	2426	null	hypothetical protein CNAG_04263	1	0	0	1
CNAG_04264	chr9	429820	431793	1973	null	ubiquinone biosynthesis monooxygenase COQ6	0	1	0	1
CNAG_04267	chr9	435845	437022	1177	null	mitochondrial genome maintenance protein	1	0	0	1
CNAG_04268	chr9	437346	439920	2574	null	exodeoxyribonuclease III	1	1	0	2
CNAG_04275	chr9	453709	455604	1895	null	metalloendopeptidase	1	0	0	1
CNAG_04283	chr9	478289	480700	2411	null	integral membrane protein	0	4	0	4
CNAG_04285	chr9	484823	485770	947	null	hypothetical protein CNAG_04285	0	1	0	1
CNAG_04289	chr9	489662	492489	2827	null	hypothetical protein CNAG_04289	0	3	0	3
**CNAG_04292**	**chr9**	**495878**	**496990**	**1112**	**null**	**hypothetical protein CNAG_04292**	**0**	**8**	**3**	**11**
CNAG_04293	chr9	497681	501496	3815	VPS41	vacuolar protein sorting 41	0	0	1	1
CNAG_04294	chr9	501650	502198	548	null	hypothetical protein CNAG_04294	0	1	0	1
CNAG_04296	chr9	504818	505762	944	null	hypothetical protein CNAG_04296	0	1	0	1
CNAG_04298	chr9	507561	510395	2834	null	nucleolar complex protein 3	0	1	0	1
CNAG_04299	chr9	510690	511010	320	null	hypothetical protein CNAG_04299	1	0	0	1
CNAG_04301	chr9	512298	514583	2285	null	hypothetical protein CNAG_04301	0	0	1	1
CNAG_04302	chr9	514766	517103	2337	null	hypothetical protein CNAG_04302	0	0	1	1
CNAG_04303	chr9	517443	520398	2955	null	E3 ubiquitin-protein ligase BRE1	1	0	0	1
CNAG_04304	chr9	520833	522925	2092	null	T-complex protein 1 subunit zeta	0	1	0	1
CNAG_04307	chr9	526410	527798	1388	URO1	urate oxidase	0	2	0	2
CNAG_04308	chr9	528315	529896	1581	HAD1	3-hydroxyacyl-CoA dehydrogenase	0	0	1	1
CNAG_04310	chr9	531720	533544	1824	null	hypothetical protein CNAG_04310	0	1	0	1
CNAG_04311	chr9	533816	535626	1810	null	charged multivesicular body protein 7	0	0	1	1
CNAG_04315	chr9	542846	545524	2678	null	hypothetical protein CNAG_04315	1	1	0	2
CNAG_04316	chr9	546426	549571	3145	null	NAD kinase	1	1	1	3
CNAG_04317	chr9	549916	551427	1511	null	hypothetical protein CNAG_04317	3	0	0	3
CNAG_04318	chr9	551542	555124	3582	null	telomere length regulation protein	4	0	1	5
CNAG_04319	chr9	555415	556035	620	null	hypothetical protein CNAG_04319	0	1	0	1
CNAG_04320	chr9	556375	558054	1679	CPS1	polysaccharide synthase Cps1p	0	1	1	2
CNAG_04324	chr9	567440	570158	2718	null	hypothetical protein CNAG_04324	0	1	1	2
CNAG_04325	chr9	571124	572330	1206	null	extradiol ring-cleavage dioxygenase	0	0	1	1
CNAG_04326	chr9	572417	574857	2440	null	malate dehydrogenase (oxaloacetate-decarboxylating)	1	0	1	2
CNAG_04330	chr9	583135	584779	1644	null	hypothetical protein CNAG_04330	0	1	0	1
CNAG_04333	chr9	591905	594210	2305	null	hypothetical protein CNAG_04333	0	1	0	1
CNAG_04335	chr9	596225	603495	7270	null	phosphatidylinositol 4-kinase	1	0	0	1
CNAG_04339	chr9	609400	612004	2604	null	exocyst complex component EXO84	0	1	0	1
CNAG_04341	chr9	615438	619038	3600	null	recyclin-1	2	0	0	2
CNAG_04345	chr9	628080	631545	3465	null	specific RNA polymerase II transcription factor	1	1	0	2
CNAG_04348	chr9	636854	638194	1340	null	chaperone	0	1	0	1
CNAG_04353	chr9	648889	651230	2341	null	hypothetical protein CNAG_04353	0	0	2	2
CNAG_04357	chr9	658614	659422	808	null	hypothetical protein CNAG_04357	0	1	0	1
CNAG_04358	chr9	659598	662062	2464	ATM1	iron-sulfur clusters transporter ATM1, mitochondrial	0	1	0	1
CNAG_04359	chr9	662580	666258	3678	null	hypothetical protein CNAG_04359	0	1	0	1
CNAG_04362	chr9	672766	674969	2203	null	ATP-dependent RNA helicase DBP5	1	0	0	1
CNAG_04377	chr9	713030	717298	4268	null	vacuolar protein	1	3	0	4

CNAG_04380	chr9	719689	722166	2477	null	peptidase	0	1	3	4
CNAG_04383	chr9	726739	727704	965	null	acetyltransferase	1	0	0	1
CNAG_04384	chr9	727941	728807	866	null	peptidyl-tRNA hydrolase	0	1	0	1
CNAG_04395	chr9	752754	754334	1580	null	protein BFR2	1	0	0	1
CNAG_04401	chr9	762970	764125	1155	null	alpha-soluble NSF attachment protein	0	0	1	1
CNAG_04402	chr9	764356	765308	952	null	splicing factor U2AF 35 kDa subunit	0	1	0	1
CNAG_04403	chr9	765460	767204	1744	null	CCCH zinc finger protein	0	1	0	1
CNAG_04410	chr9	780379	782401	2022	null	endoplasmic reticulum protein	0	0	1	1
CNAG_04411	chr9	782550	784208	1658	null	hypothetical protein CNAG_04411	1	1	1	3
CNAG_04413	chr9	787063	788542	1479	null	hypothetical protein CNAG_04413	1	0	0	1
CNAG_04416	chr9	795368	797389	2021	null	major facilitator superfamily transporter	0	0	1	1
CNAG_07768	chr9	799086	799478	392	null	hypothetical protein CNAG_07768	0	3	1	4
CNAG_07769	chr9	840577	852305	11728	TOM1	E3 ubiquitin-protein ligase HUWE1	1	0	0	1
CNAG_04433	chr9	854944	860523	5579	null	CMGC/DYRK/DYRK2 protein kinase	1	0	1	2
CNAG_04434	chr9	860926	864228	3302	null	hypothetical protein CNAG_04434	0	0	1	1
CNAG_04439	chr9	873060	875153	2093	null	V-type proton ATPase subunit B	0	1	0	1
CNAG_04441	chr9	876626	878793	2167	null	polyadenylate-binding protein, cytoplasmic and nuclear	0	1	0	1
CNAG_04443	chr9	880908	882153	1245	null	hypothetical protein CNAG_04443	1	2	0	3
CNAG_04446	chr9	887452	888610	1158	null	hypothetical protein CNAG_04446	0	0	2	2
CNAG_04451	chr9	901435	904412	2977	null	ribonuclease P/MRP protein subunit POP1	0	0	2	2
CNAG_04453	chr9	905446	906895	1449	IPC1	inositolphosphorylceramide synthase	1	0	0	1
CNAG_04454	chr9	907132	908194	1062	null	hypothetical protein CNAG_04454	1	1	0	2
CNAG_04457	chr9	914368	915684	1316	null	hypothetical protein CNAG_04457	0	1	0	1
CNAG_04460	chr9	921083	922873	1790	null	RNA polymerase II transcription factor	0	1	0	1
CNAG_04461	chr9	923265	929403	6138	HFM1	ATP-dependent DNA helicase HFM1/MER3	1	0	0	1
CNAG_04462	chr9	929511	931529	2018	null	F-box protein 9	0	1	1	2
CNAG_04463	chr9	931725	932521	796	null	hypothetical protein CNAG_04463	1	0	0	1
CNAG_04466	chr9	936397	938746	2349	null	hypothetical protein CNAG_04466	0	1	0	1
CNAG_04467	chr9	939356	941311	1955	null	succinate-semialdehyde dehydrogenase (NADP)	0	2	0	2
CNAG_04468	chr9	941767	943259	1492	null	tartrate dehydrogenase	0	0	1	1
CNAG_04469	chr9	943620	945383	1763	null	4-aminobutyrate transaminase	0	2	1	3
CNAG_04470	chr9	946041	946952	911	null	haloacid dehalogenase, type II	0	1	0	1
CNAG_04471	chr9	947441	949222	1781	null	FAD dependent oxidoreductase	0	1	0	1
CNAG_04472	chr9	949899	951689	1790	null	membrane protein	1	0	0	1
CNAG_04475	chr9	959137	960190	1053	null	hypothetical protein CNAG_04475	1	1	0	2
CNAG_04476	chr9	960381	963440	3059	null	hypothetical protein CNAG_04476	2	0	2	4
CNAG_07771	chr9	964714	967625	2911	null	peptidase	0	1	0	1
CNAG_04478	chr9	968037	971128	3091	null	hypothetical protein CNAG_04478	2	1	2	5
CNAG_04479	chr9	971585	973214	1629	null	hypothetical protein CNAG_04479	0	0	1	1
CNAG_07772	chr9	973346	976575	3229	null	hypothetical protein CNAG_07772	2	2	2	6
CNAG_07773	chr9	977371	979477	2106	null	hypothetical protein CNAG_07773	0	1	0	1
CNAG_07774	chr9	979819	982146	2327	null	hypothetical protein CNAG_07774	2	0	2	4
CNAG_04484	chr9	983144	986823	3679	null	hypothetical protein CNAG_04484	1	0	0	1
CNAG_04485	chr9	987131	989549	2418	null	long-chain acyl-CoA synthetase	0	0	1	1
CNAG_04486	chr9	990661	991544	883	null	hypothetical protein CNAG_04486	0	1	0	1
CNAG_07777	chr9	999087	999797	710	null	hypothetical protein CNAG_07777	0	1	0	1
CNAG_04492	chr9	1000069	1002725	2656	null	hypothetical protein CNAG_04492	1	0	1	2

CNAG_04493	chr9	1003331	1006922	3591	null	ubiquitin carboxyl-terminal hydrolase 48	2	5	0	7
CNAG_04495	chr9	1009095	1009868	773	null	hypothetical protein CNAG_04495	1	3	0	4
CNAG_04496	chr9	1010368	1014044	3676	null	rab GTPase activator	1	1	3	5
CNAG_04497	chr9	1014541	1015683	1142	null	hypothetical protein CNAG_04497	0	1	0	1
CNAG_04498	chr9	1015954	1019570	3616	null	alpha-1,2-mannosidase	0	0	1	1
CNAG_04499	chr9	1020456	1021550	1094	null	clathrin light chain	1	0	0	1
CNAG_04500	chr9	1021773	1024046	2273	null	hypothetical protein CNAG_04500	1	0	0	1
CNAG_04501	chr9	1024146	1026500	2354	TRP1	anthranilate synthase/indole-3-glycerol phosphate synthase/phosphoribosylanthranilate isomerase	1	0	0	1
CNAG_04502	chr9	1027279	1030718	3439	null	hypothetical protein CNAG_04502	0	2	0	2
CNAG_04504	chr9	1032945	1034521	1576	null	hypothetical protein CNAG_04504	0	0	1	1
CNAG_04505	chr9	1036332	1037938	1606	GPA1	guanine nucleotide-binding protein subunit alpha	0	3	0	3
CNAG_04506	chr9	1041920	1043142	1222	null	hypothetical protein CNAG_04506	1	0	0	1
CNAG_04507	chr9	1043724	1044981	1257	null	hypothetical protein CNAG_04507	0	1	0	1
CNAG_04508	chr9	1046488	1047414	926	null	hypothetical protein CNAG_04508	0	1	0	1
CNAG_04509	chr9	1048030	1049278	1248	null	hypothetical protein CNAG_04509	1	0	1	2
CNAG_04513	chr9	1054403	1058448	4045	null	DNA-directed RNA polymerase III subunit RPC2	0	1	0	1
CNAG_04514	chr9	1058771	1060580	1809	MPK1	CMGC/MAPK protein kinase	2	0	0	2
CNAG_04518	chr9	1067183	1069760	2577	null	hypothetical protein CNAG_04518	0	1	0	1
CNAG_04520	chr9	1072301	1074293	1992	null	D-aminoacylase	0	1	0	1
CNAG_04521	chr9	1076601	1078434	1833	null	oxidoreductase	0	1	1	2
CNAG_04522	chr9	1079853	1081435	1582	null	hypothetical protein CNAG_04522	0	1	1	2
CNAG_04524	chr9	1084902	1088197	3295	null	zinc metalloprotease	1	0	0	1
CNAG_04526	chr9	1091869	1093518	1649	null	hypothetical protein CNAG_04526	3	0	0	3
CNAG_04527	chr9	1093802	1095055	1253	null	hypothetical protein CNAG_04527	0	2	0	2
**CNAG_04528**	**chr9**	**1095325**	**1098629**	**3304**	**null**	**hypothetical protein CNAG_04528**	**8**	**0**	**4**	**12**
CNAG_04529	chr9	1099033	1099619	586	null	cytochrome c oxidase-assembly factor COX16	0	1	0	1
CNAG_04530	chr9	1099830	1101257	1427	null	DNA-binding protein	0	1	0	1
CNAG_04531	chr9	1101680	1102697	1017	null	enoyl-CoA hydratase	0	1	0	1
CNAG_04534	chr9	1106819	1110016	3197	null	hypothetical protein CNAG_04534	1	0	0	1
CNAG_04535	chr9	1110498	1111916	1418	null	tartrate dehydrogenase	0	1	0	1
CNAG_04536	chr9	1112968	1115229	2261	null	nicotinamide mononucleotide permease	0	2	0	2
CNAG_04537	chr9	1115470	1117090	1620	null	hypothetical protein CNAG_04537	1	1	1	3
CNAG_04538	chr9	1117620	1120096	2476	null	ubiquitin-like modifier-activating enzyme ATG7	0	0	1	1
CNAG_04541	chr9	1123822	1125901	2079	null	cleavage stimulation factor subunit 2	0	1	1	2
CNAG_07780	chr9	1133103	1134516	1413	null	geranylgeranyl diphosphate synthase, type III	0	1	0	1
CNAG_07781	chr9	1134904	1141509	6605	null	ATP-dependent bile acid transporter	1	0	0	1
CNAG_07020	chr9	1142403	1144112	1709	null	hypothetical protein CNAG_07020	1	0	0	1

Genes with more than 20 variants are indicated in bold
