# Supplementary material for: Identification of Pathogen Genomic Differences That Impact Human Immune Response and Disease during Cryptococcus neoformans Infection
Source: mBio. 2019 Jul 16;10(4):e01440-19. doi: 10.1128/mBio.01440-19 (PMC6635531; doi:10.1128/mBio.01440-19)
Supplement: TABLE S2 [file mBio.01440-19-st002.pdf]

TableS2. ST93A and ST93B clade-specific variants

CHROM	POS	REF	ALT	gene	effect	impact	class	aaChange	clade	alias	description
1	944973	G	C	CNAG_00372	nonsynonymous	MODERATE	MISSENSE	A672G	A	null	chromatin structure-remodeling complex protein RSC7
1	1583615	C	T	CNAG_00619	nonsynonymous	MODERATE	MISSENSE	A223T	A	null	tubulin folding cofactor C
2	150696	T	C	CNAG_06749	nonsynonymous	MODERATE	MISSENSE	I14T	A	null	hypothetical protein
2	774357	A	C	CNAG_03772	nonsynonymous	MODERATE	MISSENSE	C126G	A	HXS1	high-affinity glucose transporter (HXS1)
3	1180025	C	T	CNAG_07552	nonsynonymous	MODERATE	MISSENSE	A256V	A	null	DNA repair protein Rad8
4	577501	G	A	CNAG_05155	nonsynonymous	MODERATE	MISSENSE	A539V	A	PTP2	protein tyrosine phosphatase (PTP2)
6	239210	G	A	CNAG_02468	nonsynonymous	MODERATE	MISSENSE	S545F	A	null	AP-3 complex subunit delta-1
6	602467	C	G	CNAG_02327	nonsynonymous	MODERATE	MISSENSE	A113P	A	null	xaa-Pro dipeptidase
6	1167676	C	G	CNAG_02108	nonsynonymous	MODERATE	MISSENSE	A19G	A	null	GTPase activating protein
7	230511	A	G	CNAG_06597	nonsynonymous	MODERATE	MISSENSE	Q479R	A	SPT8	predicted saga histone acetyltransferase complex component (SPT8)
7	1191543	C	T	CNAG_05910	nonsynonymous	MODERATE	MISSENSE	G515S	A	null	hypothetical protein
8	294928	T	A	CNAG_03189	nonsynonymous	MODERATE	MISSENSE	R793S	A	null	DIL and ankyrin domain-containing protein
8	1083181	T	C	CNAG_03484	nonsynonymous	MODERATE	MISSENSE	S555P	A	null	kinetochore protein Mis13/DSN1
8	1256218	AC	A	CNAG_03551	indel	HIGH	NONE	V58	A	null	hypothetical protein
9	534468	C	T	CNAG_04311	nonsynonymous	MODERATE	MISSENSE	A184V	A	null	charged multivesicular body protein 7
11	153063	A	G	CNAG_01512	nonsynonymous	LOW	NONE	NA	A	null	hypothetical protein
11	433361	G	A	CNAG_01616	nonsynonymous	MODERATE	MISSENSE	T835I	A	null	hypothetical protein
11	621705	A	C	CNAG_01690	nonsynonymous	MODERATE	MISSENSE	N518T	A	null	MFS transporter
11	674162	C	A	CNAG_01708	nonsynonymous	MODERATE	MISSENSE	L636I	A	null	hypothetical protein
13	111766	G	A	CNAG_06292	nonsynonymous	MODERATE	MISSENSE	L476F	A	null	sugar transporter
13	748576	C	G	CNAG_07041	nonsynonymous	MODERATE	MISSENSE	P115A	A	null	hypothetical protein
14	23327	C	T	CNAG_05341	nonsynonymous	LOW	NONE	NA	A	null	hypothetical protein
3	401715	C	T	CNAG_02936	nonsynonymous	MODERATE	MISSENSE	S437L	B	null	CCR4-NOT transcription complex subunit 1
9	272607	G	A	CNAG_04199	nonsynonymous	MODERATE	MISSENSE	V83M	B	null	hypothetical protein
9	491535	G	A	CNAG_04289	nonsynonymous	MODERATE	MISSENSE	P285L	B	null	hypothetical protein
11	236388	G	A	CNAG_01539	nonsynonymous	MODERATE	MISSENSE	R141C	B	null	myo-inositol-1-phosphate synthase
14	68046	G	A	CNAG_05352	nonsynonymous	MODERATE	MISSENSE	A376T	B	null	hypothetical protein
14	271762	C	T	CNAG_05422	nonsynonymous	MODERATE	MISSENSE	S737N	B	LIV11	virulence related protein of unknown function (LIV11)
