# Supplementary figures and images for: Identification of Pathogen Genomic Differences That Impact Human Immune Response and Disease during Cryptococcus neoformans Infection
Source: mBio. 2019 Jul 16;10(4):e01440-19. doi: 10.1128/mBio.01440-19 (PMC6635531; doi:10.1128/mBio.01440-19)

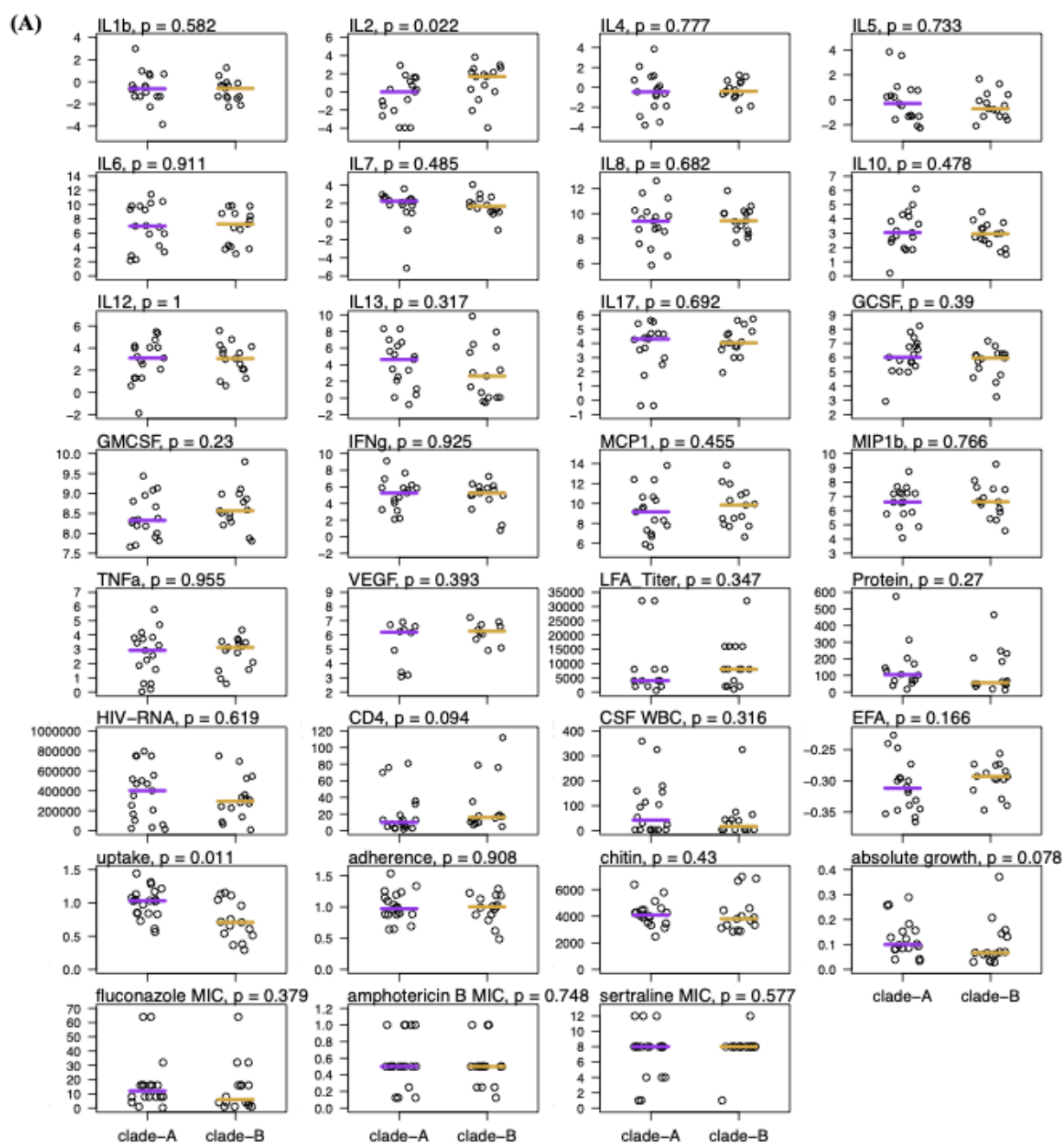

Figure S1. Clade-specific differences in phenotype. Bar indicates median value.

Supplement: FIG S1 [file mBio.01440-19-sf001.pdf]
