# Supplementary material for: Identification of Pathogen Genomic Differences That Impact Human Immune Response and Disease during Cryptococcus neoformans Infection
Source: mBio. 2019 Jul 16;10(4):e01440-19. doi: 10.1128/mBio.01440-19 (PMC6635531; doi:10.1128/mBio.01440-19)
Supplement: TABLE S3 [file mBio.01440-19-st003.pdf]

Table S3. Statistical analysis of ST93 Clade-specific associations with quantitative infection phenotypes

phenotype	W	A strains mean - B strains mean	pvalue	significant
IL1b	142.5	0.23	0.582	
IL2	66.5	-1.62	0.022	*
IL4	119.5	-0.17	0.777	
IL5	137	0.4	0.733	
IL6	131	0.09	0.911	
IL7	146.5	-0.14	0.485	
IL8	116	-0.27	0.682	
IL10	147	0.29	0.478	
IL12	127	-0.18	1	
IL13	154.5	1	0.317	
IL17	116.5	-0.51	0.692	
GCSF	151	0.43	0.39	
GMCSF	95	-0.21	0.23	
IFNg	124.5	-0.18	0.925	
MCP1	107	-0.57	0.455	
MIP1b	119	-0.17	0.766	
TNFa	129.5	-0.06	0.955	
VEGF	38	-0.82	0.393	
LFA_Titer	77	-1300	0.347	
Protein	131	29.17	0.27	
Survival_from_CM	197	92.09	0.14	
hivrna_002	167.5	54728.58	0.619	
cd4_002	101	-7.48	0.094	
csf_wbc_a00	152	43.95	0.316	
csf_wbc_000	152	43.95	0.316	
uptake	226	0.27	0.011	*
adherence	154	0.03	0.908	
chitin	166	-37.04	0.43	
Absolute.Growth	195.5	0.03	0.078	
Fluconazole.MIC	169.5	3.37	0.379	
Amphotericin.B.MIC	153	0.04	0.748	
Sertraline.MIC	130.5	-0.59	0.577	
