# Supplementary material for: Identification of Pathogen Genomic Differences That Impact Human Immune Response and Disease during Cryptococcus neoformans Infection
Source: mBio. 2019 Jul 16;10(4):e01440-19. doi: 10.1128/mBio.01440-19 (PMC6635531; doi:10.1128/mBio.01440-19)
Supplement: TABLE S4 [file mBio.01440-19-st004.pdf]

Table S4: Phenotypes measured from patients enrolled in the COAT trial (clinical and cytokines) and *in vitro*.

Class	Phenotype	Lines with data missing
Clinical	LFA titer	UgCl390, UgCl447, UgCl450, UgCl461, UgCl462, UgCl466, UgCl468, UgCl495 UgCl541
Clinical	protein	UgCl447, UgCl450, UgCl461, UgCl462, UgCl466, UgCl468, UgCl495 UgCl541
Clinical	HIV-viral	UgCl360, UgCl395, UgCl447, UgCl547
Clinical	EFA	UgCl332, UgCl447
Clinical	CD4	UgCl447
Clinical	CSF WBC	UgCl360, UgCl395, UgCl447, UgCl547
Clinical	Survival	UgCl447
Cytokines	IL1-b	UgCl447, UgCl461, UgCl541
Cytokines	IL2	UgCl447, UgCl461, UgCl541
Cytokines	IL4	UgCl447, UgCl461, UgCl541
Cytokines	IL-5	UgCl447, UgCl461, UgCl541
Cytokines	IL-6	UgCl447, UgCl461, UgCl541
Cytokines	IL-7	UgCl447, UgCl461, UgCl541
Cytokines	IL-8	UgCl447, UgCl461, UgCl541
Cytokines	IL-10	UgCl447, UgCl461, UgCl541
Cytokines	IL-12	UgCl447, UgCl461, UgCl541
Cytokines	IL-13	UgCl447, UgCl461, UgCl541
Cytokines	IL-17	UgCl447, UgCl461, UgCl541
Cytokines	GCSF	UgCl447, UgCl461, UgCl541
Cytokines	GMCSF	UgCl447, UgCl461, UgCl541
Cytokines	IFNg	UgCl447, UgCl461, UgCl541
Cytokines	**MCP1**	UgCl447, UgCl461, UgCl541
Cytokines	**TNFα**	UgCl447, UgCl461, UgCl541
Cytokines	MIP1b	UgCl447, UgCl461, UgCl541
in vitro	uptake	UgCl212
in vitro	adherence	UgCl212
in vitro	chitin	UgCl212, UgCl549
in vitro	absolute growth	UgCl357, UgCl422
in vitro	fluconaozle MIC	UgCl357, UgCl422
in vitro	amphoterecin B MIC	UgCl357, UgCl422
in vitro	sertraline MIC	UgCl357, UgCl422
