# Supplementary material for: Identification of Pathogen Genomic Differences That Impact Human Immune Response and Disease during Cryptococcus neoformans Infection
Source: mBio. 2019 Jul 16;10(4):e01440-19. doi: 10.1128/mBio.01440-19 (PMC6635531; doi:10.1128/mBio.01440-19)
Supplement: TABLE S5 [file mBio.01440-19-st005.pdf]

Table S5. Significant variants in genes and hypothetical RNAs associated with quantitative infection phenotypes based on class designation

gene	chr	pos	class	effect	pheno	numPhen	loc
CNAG_00014	1	47564	b	ns	GCSF	1	740
CNAG_00014	1	47575	b	ns	GCSF	1	751
CNAG_00014	1	47671	b	ns	GMCSF	1	847
CNAG_00363	1	927896	b	ns	IL2	1	4528
CNAG_00363	1	927901	b	ns	IL2	1	4533
CNAG_01241	5	836479	b	upstream	IL2	1	3092
CNAG_01241	5	836697	ab	upstream	IL4;IL5;IL7;IL17;GMCSF;TNFa;chitin	7	3310
CNAG_01241	5	836899	ab	upstream	IL5;IL12;IL13;IL17;GCSF;TNFa	6	3512
CNAG_01371	5	475470	a	UTR-5	MCP1;hivrna	2	322
CNAG_01802	11	966644	b	upstream	csf_wbc	1	2357
CNAG_01802	11	966669	b	upstream	IL2	1	2382
CNAG_01802	11	966700	b	upstream	IL7	1	2413
CNAG_02112	6	1160524	b	upstream	AMP	1	3788
CNAG_02112	6	1160528	b	upstream	AMP	1	3792
CNAG_02112	6	1160532	b	upstream	AMP	1	3796
CNAG_02176	6	988843	b	ns	MIP1b	1	2018
CNAG_02176	6	988922	b	ns	LFA_Titer	1	1939
CNAG_02176	6	989188	b	ns	IL12	1	1673
CNAG_02176	6	989334	b	ns	AMP	1	1527
CNAG_02176	6	989732	b	ns	IL2	1	1129
CNAG_02176	6	990777	b	STOP_GAINED	MIP1b	1	84
CNAG_02176	6	990885	b	start+	SERT	1	-24
CNAG_02176	6	988405	ab	UTR-3	chitin;SERT	2	-92
CNAG_02176	6	988733	ab	STOP_GAINED	IL1b;IL13;MCP1;MIP1b	4	2128
CNAG_02176	6	989490	ab	ns	hivrna;SERT	2	1371
CNAG_02176	6	990771	ab	ns	IL10;MIP1b	2	90
CNAG_02176	6	990851	ab	ns	IL10;MIP1b	2	10
CNAG_02176	6	991027	ab	UTR-5	IL13;TNFa;Survival	3	166
CNAG_02177	6	990701	a	upstream	IL1b;IL6;IL10	3	3827
CNAG_02475	6	221275	b	upstream	growth	1	1163
CNAG_02475	6	221282	b	upstream	growth	1	1170
CNAG_02475	6	221273	ab	upstream	IL7;growth	2	1161

CNAG_02798	3	750294	a	upstream	cd4;AMP	2	3991
CNAG_04100	9	7729	b	upstream	growth	1	3092
CNAG_04100	9	5213	ab	upstream	adherence;FLC;SERT	3	576
CNAG_04100	9	8171	ab	upstream	efa;SERT	2	3534
CNAG_04102	9	10033	a	UTR-3	GMCSF;efa	2	-705
CNAG_04179	9	220963	a	upstream	efa;SERT	2	3710
CNAG_04373	9	706175	b	upstream	Survival	1	2598
CNAG_04373	9	705343	ab	upstream	IL8;efa	2	1766
CNAG_04535	9	1115286	a	upstream	IL17;GCSF;LFA_Titer;protein	4	3370
CNAG_04922	10	18908	b	upstream	IL2	1	762
CNAG_04922	10	18915	b	upstream	IL2	1	769
CNAG_04922	10	18933	b	upstream	IL2	1	787
CNAG_04922	10	18941	b	upstream	IL2	1	795
CNAG_04922	10	18988	b	upstream	adherence	1	842
CNAG_04922	10	18992	b	upstream	adherence	1	846
CNAG_04922	10	18997	b	upstream	adherence	1	851
CNAG_05185	4	667446	b	UTR-5	uptake	1	280
CNAG_05185	4	667433	ab	UTR-5	Survival;uptake	2	267
CNAG_05450	14	342562	a	ns	IL6;IL7;IL12;IL13;GCSF;MIP1b	6	1998
CNAG_05661	14	909638	b	upstream	adherence	1	4406
CNAG_05661	14	910152	b	upstream	uptake	1	4920
CNAG_05661	14	908850	ab	upstream	IL8;GMCSF;IFNg;MCP1	4	3618
CNAG_05661	14	909011	ab	upstream	IL1b;IL8;MIP1b;uptake;FLC	5	3779
CNAG_05661	14	910181	ab	upstream	IL1b;IL6;IFNg;hivrna	4	4949
CNAG_05661	14	908994	ab	upstream	uptake;FLC	2	3762
CNAG_05662	14	910742	b	UTR-3	AMP	1	-141
CNAG_05662	14	910834	b	downstream	Survival	1	-233
CNAG_05662	14	910926	b	downstream	SERT	1	-325
CNAG_05662	14	910966	b	downstream	Survival	1	-365
CNAG_05662	14	911262	b	downstream	MCP1	1	-661
CNAG_05662	14	911292	b	downstream	IL2	1	-691
CNAG_05662	14	911308	b	downstream	adherence	1	-707
CNAG_05662	14	911321	b	downstream	IL5	1	-720
CNAG_05662	14	911352	b	downstream	MCP1	1	-751

CNAG_05662	14	910822	ab	downstream	Survival;FLC	2	-221
CNAG_05662	14	910939	ab	downstream	growth;SERT	2	-338
CNAG_05662	14	910964	ab	downstream	Survival;AMP	2	-363
CNAG_05662	14	910979	ab	downstream	Survival;uptake	2	-378
CNAG_05662	14	911099	ab	downstream	IL12;GMCSF;MCP1;TNFa;growth	5	-498
CNAG_05662	14	911129	ab	downstream	IL12;IL13;IL17;MIP1b;TNFa;growth;FLC;AMP;SERT	9	-528
CNAG_05662	14	911206	ab	downstream	IL8;MCP1;MIP1b	3	-605
CNAG_05663	14	910323	b	downstream	TNFa	1	-1905
CNAG_05663	14	910555	b	downstream	Survival	1	-1673
CNAG_05663	14	910328	ab	downstream	IL1b;IL13;TNFa	3	-1900
CNAG_05913	7	1205599	ab	upstream	MIP1b;adherence	2	2952
CNAG_05913	7	1205600	ab	upstream	IL13;IL17;MIP1b;adherence	4	2953
CNAG_05937	7	1263610	a	UTR-5	uptake;SERT	2	10
CNAG_05937	7	1263646	b	UTR-5	SERT	1	46
CNAG_05937	7	1263647	b	UTR-5	SERT	1	47
CNAG_05987	12	14009	b	ns	IL2	1	990
CNAG_05987	12	14035	b	ns	IL2	1	964
CNAG_05987	12	15014	b	UTR-5	adherence	1	15
CNAG_05987	12	14197	ab	ns	efa;adherence	2	802
CNAG_05987	12	14202	ab	frameshift	efa;adherence	2	797
CNAG_05987	12	14125	b	CODON_CHANGE_PLUS_CODON_DELETION	chitin	1	874
CNAG_06169	12	502808	b	UTR-3	IL8	1	-1164
CNAG_06169	12	503311	b	UTR-3	GCSF	1	-661
CNAG_06169	12	502888	ab	UTR-3	GMCSF;growth	2	-1084
CNAG_06169	12	502890	ab	UTR-3	IL6;IL8;GMCSF	3	-1082
CNAG_06169	12	503049	ab	UTR-3	GMCSF;hivrna	2	-923
CNAG_06169	12	503112	ab	UTR-3	hivrna;csf_wbc	2	-860
CNAG_06169	12	503313	ab	UTR-3	IL12;IL13;GCSF	3	-659
CNAG_06169	12	503321	ab	UTR-3	IL12;IL13;GCSF;MIP1b	4	-651
CNAG_06169	12	503327	ab	UTR-3	IL12;IL13;MIP1b	3	-645
CNAG_06169	12	503401	ab	UTR-3	IL10;chitin	2	-571
CNAG_06256	13	11130	b	upstream	TNFa	1	4551
CNAG_06256	13	11118	ab	upstream	IFNg;TNFa	2	4539
CNAG_06332	13	219311	b	upstream	efa	1	2073

CNAG_06332	13	219312	b	upstream	efa	1	2074
CNAG_06332	13	219021	b	upstream	adherence	1	1783
CNAG_06422	13	436551	b	UTR-5	IL2	1	58
CNAG_06422	13	436554	b	UTR-5	IL2	1	55
CNAG_06490	13	655915	a	frameshift	hivrna;cd4	2	2826
CNAG_06525	7	11056	ab	ns	IL5;IL10	2	417
CNAG_06525	7	14006	ab	upstream	IL6;IL8	2	2533
CNAG_06574	7	164473	b	upstream	hivrna	1	615
CNAG_06574	7	165027	b	upstream	MIP1b	1	1169
CNAG_06574	7	165873	b	upstream	IL13	1	2015
CNAG_06574	7	166309	b	upstream	growth	1	2451
CNAG_06574	7	167135	b	upstream	IL13	1	3277
CNAG_06574	7	167224	b	upstream	GMCSF	1	3366
CNAG_06574	7	164887	ab	upstream	IL2;TNFa	2	1029
CNAG_06574	7	164926	ab	upstream	IL2;MIP1b	2	1068
CNAG_06574	7	165704	ab	upstream	Survival;efa	2	1846
CNAG_06574	7	167292	ab	upstream	IL1b;GCSF;MIP1b;uptake	4	3434
CNAG_06574	7	167370	ab	upstream	cd4;uptake	2	3512
CNAG_06704	2	270700	a	upstream	IL2;protein	2	3904
CNAG_06876	5	7093	a	UTR-3	IFNg;MIP1b;TNFa	3	-192
CNAG_06968	8	1383765	a	CODON_INSERTION	IL12;IL17	2	236
CNAG_07026	12	11092	ab	upstream	IL1b;IL13;Survival;efa	4	2904
CNAG_07026	12	11094	ab	upstream	IL13;LFA_Titer;Survival	3	2906
CNAG_07026	12	11400	ab	upstream	IL1b;IL7;IL13;LFA_Titer	4	3212
CNAG_07026	12	11406	ab	upstream	IL1b;IL7;IL13;LFA_Titer	4	3218
CNAG_07026	12	11407	ab	upstream	IL1b;IL7;IL13;LFA_Titer	4	3219
CNAG_07026	12	11410	ab	upstream	IL1b;IL7;IL13;LFA_Titer	4	3222
CNAG_07026	12	11413	ab	upstream	IL1b;LFA_Titer	2	3225
CNAG_07703	7	1341024	a	ns	IL6;IL8	2	521
CNAG_07837	10	13558	b	UTR-5	IL2	1	129
CNAG_07837	10	15302	b	UTR-3	cd4	1	-526
CNAG_07837	10	15288	b	UTR-3	csf_wbc	1	-512
CNAG_07950	1	975397	b	upstream	efa	1	1550
CNAG_07950	1	975152	ab	upstream	IL8;hivrna	2	1795

CNAG_07950	1	975212	ab	upstream	IL4;IL6;IL8;GMCSF;IFNg;FLC	6	1735
CNAG_08006	11	804710	ab	UTR-5	IL4;IL5;IL6;MIP1b;TNFa;adherence;chitin	7	164
CNAG_08006	11	804742	ab	UTR-5	IL4;IFNg;MCP1;adherence	4	132
CNAG_12610	7	49744	a	upstream	MCP1;uptake	2	4416
CNAG_13108	13	128625	ab	upstream	IL13;GCSF	2	339
CNAG_13108	13	128715	ab	upstream	IL13;GCSF	2	249
CNAG_13108	13	128729	ab	upstream	IL13;GCSF	2	235
CNAG_13204	14	924025	b	upstream	GMCSF	1	4660
CNAG_13204	14	924047	b	upstream	IL13	1	4682
CNAG_13204	14	924049	b	upstream	IL13	1	4684
CNAG_13204	14	924050	b	upstream	IL13	1	4685
