# Supplementary material for: Identification of Pathogen Genomic Differences That Impact Human Immune Response and Disease during Cryptococcus neoformans Infection
Source: mBio. 2019 Jul 16;10(4):e01440-19. doi: 10.1128/mBio.01440-19 (PMC6635531; doi:10.1128/mBio.01440-19)
Supplement: TABLE S6 [file mBio.01440-19-st006.pdf]

Table S6. Logistic regression analysis of all significant variants in genes and hypothetical RNAs associated with quantitative infection phenotypes

gene	chr	pos	class	effect	pheno	odds ratio (CI)	logistic regression
CNAG_00014	1	47564	b	ns	GCSF	0.22 (0.05,0.97)	est = -1.53, error = 0.76, z = -2, p = 0.045
CNAG_00014	1	47575	b	ns	GCSF	0.2 (0.04,0.91)	est = -1.62, error = 0.78, z = -2.08, p = 0.038
CNAG_00014	1	47671	b	ns	GMCSF	3.17 (1.21,8.32)	est = 1.15, error = 0.49, z = 2.35, p = 0.019
CNAG_00363	1	927896	b	ns	IL2	1.78 (1.04,3.04)	est = 0.57, error = 0.27, z = 2.09, p = 0.036
CNAG_00363	1	927901	b	ns	IL2	1.78 (1.04,3.04)	est = 0.57, error = 0.27, z = 2.09, p = 0.036
CNAG_01241	5	836479	b	upstream	IL2	1.97 (1.15,3.37)	est = 0.68, error = 0.28, z = 2.46, p = 0.014
CNAG_01241	5	836697	ab	upstream	IL4	1.93 (1.03,3.62)	est = 0.66, error = 0.32, z = 2.06, p = 0.039
CNAG_01241	5	836697	ab	upstream	IL5	2.81 (1.27,6.2)	est = 1.03, error = 0.4, z = 2.55, p = 0.011
CNAG_01241	5	836697	ab	upstream	IL7	3.05 (1.08,8.64)	est = 1.11, error = 0.53, z = 2.1, p = 0.036
CNAG_01241	5	836697	ab	upstream	IL17	2.11 (1.01,4.41)	est = 0.75, error = 0.38, z = 1.99, p = 0.047
CNAG_01241	5	836697	ab	upstream	GMCSF	3.18 (1.26,8.02)	est = 1.16, error = 0.47, z = 2.45, p = 0.014
CNAG_01241	5	836697	ab	upstream	TNFa	2.3 (1.16,4.56)	est = 0.83, error = 0.35, z = 2.4, p = 0.017
CNAG_01241	5	836697	ab	upstream	chitin	1.0009 (1.0001,1.0017)	est = 0, error = 0, z = 2.14, p = 0.032
CNAG_01241	5	836899	ab	upstream	IL5	0.17 (0.03,0.88)	est = -1.74, error = 0.82, z = -2.11, p = 0.035
CNAG_01241	5	836899	ab	upstream	IL12	0.3 (0.11,0.81)	est = -1.21, error = 0.51, z = -2.38, p = 0.017
CNAG_01241	5	836899	ab	upstream	IL13	0.38 (0.16,0.9)	est = -0.96, error = 0.44, z = -2.19, p = 0.028
CNAG_01241	5	836899	ab	upstream	IL17	0.45 (0.23,0.9)	est = -0.79, error = 0.35, z = -2.25, p = 0.025
CNAG_01241	5	836899	ab	upstream	GCSF	12.58 (1.38,114.77)	est = 2.53, error = 1.13, z = 2.24, p = 0.025
CNAG_01241	5	836899	ab	upstream	TNFa	0.34 (0.14,0.81)	est = -1.08, error = 0.44, z = -2.43, p = 0.015
CNAG_01371	5	475470	a	UTR-5	MCP1	0.56 (0.34,0.91)	est = -0.59, error = 0.25, z = -2.32, p = 0.02
CNAG_01371	5	475470	a	UTR-5	hivrna	1 (1,1)	est = 0, error = 0, z = 2.25, p = 0.025
CNAG_01802	11	966644	b	upstream	csf_wbc	1.01 (1,1.03)	est = 0.01, error = 0.01, z = 2.37, p = 0.018
CNAG_01802	11	966669	b	upstream	IL2	1.54 (1,2.36)	est = 0.43, error = 0.22, z = 1.96, p = 0.05
CNAG_01802	11	966700	b	upstream	IL7	0.35 (0.13,0.98)	est = -1.04, error = 0.52, z = -2, p = 0.045
CNAG_02112	6	1160524	b	upstream	AMP	12.99 (1.05,161.24)	est = 2.56, error = 1.29, z = 1.99, p = 0.046
CNAG_02112	6	1160528	b	upstream	AMP	12.99 (1.05,161.24)	est = 2.56, error = 1.29, z = 1.99, p = 0.046
CNAG_02112	6	1160532	b	upstream	AMP	12.99 (1.05,161.24)	est = 2.56, error = 1.29, z = 1.99, p = 0.046
CNAG_02176	6	988843	b	ns	MIP1b	0.45 (0.22,0.92)	est = -0.79, error = 0.36, z = -2.18, p = 0.029
CNAG_02176	6	988922	b	ns	LFA_Titer	0.4 (0.16,0.97)	est = -0.92, error = 0.45, z = -2.02, p = 0.043
CNAG_02176	6	989188	b	ns	IL12	1.75 (1,3.06)	est = 0.56, error = 0.29, z = 1.97, p = 0.049
CNAG_02176	6	989334	b	ns	AMP	NA	est = -3.16, error = 1.58, z = -2, p = 0.045
CNAG_02176	6	989732	b	ns	IL2	1.53 (1.01,2.34)	est = 0.43, error = 0.21, z = 2, p = 0.046
CNAG_02176	6	990777	b	STOP_GAINED	MIP1b	0.48 (0.24,0.97)	est = -0.73, error = 0.36, z = -2.04, p = 0.041
CNAG_02176	6	990885	b	start+	SERT	NA	est = -0.31, error = 0.15, z = -2.04, p = 0.042
CNAG_02176	6	988405	ab	UTR-3	chitin	NA	est = 0, error = 0, z = -1.99, p = 0.047
CNAG_02176	6	988405	ab	UTR-3	SERT	NA	est = -0.29, error = 0.15, z = -2, p = 0.046
CNAG_02176	6	988733	ab	STOP_GAINED	IL1b	0.41 (0.18,0.93)	est = -0.88, error = 0.41, z = -2.13, p = 0.033
CNAG_02176	6	988733	ab	STOP_GAINED	IL13	0.76 (0.59,0.98)	est = -0.27, error = 0.13, z = -2.08, p = 0.038
CNAG_02176	6	988733	ab	STOP_GAINED	MCP1	0.6 (0.39,0.92)	est = -0.51, error = 0.22, z = -2.34, p = 0.019
CNAG_02176	6	988733	ab	STOP_GAINED	MIP1b	0.27 (0.11,0.7)	est = -1.3, error = 0.48, z = -2.69, p = 0.007
CNAG_02176	6	989490	ab	ns	hivrna	1 (1,1)	est = 0, error = 0, z = -2.15, p = 0.032
CNAG_02176	6	989490	ab	ns	SERT	NA	est = -0.41, error = 0.19, z = -2.21, p = 0.027
CNAG_02176	6	990771	ab	ns	IL10	0.39 (0.16,0.97)	est = -0.95, error = 0.47, z = -2.03, p = 0.042

CNAG_02176	6	990771	ab	ns	MIP1b	0.49 (0.24,0.98)	est = -0.72, error = 0.36, z = -2, p = 0.045
CNAG_02176	6	990851	ab	ns	IL10	0.39 (0.15,1)	est = -0.95, error = 0.48, z = -1.97, p = 0.049
CNAG_02176	6	990851	ab	ns	MIP1b	0.45 (0.22,0.92)	est = -0.81, error = 0.37, z = -2.18, p = 0.029
CNAG_02176	6	991027	ab	UTR-5	IL13	0.69 (0.48,0.97)	est = -0.38, error = 0.18, z = -2.11, p = 0.034
CNAG_02176	6	991027	ab	UTR-5	TNFa	0.5 (0.26,0.98)	est = -0.68, error = 0.34, z = -2.03, p = 0.042
CNAG_02176	6	991027	ab	UTR-5	Survival	0.991 (0.9841,0.9978)	est = -0.01, error = 0, z = -2.58, p = 0.01
CNAG_02177	6	990701	a	upstream	IL1b	0.36 (0.15,0.87)	est = -1.03, error = 0.46, z = -2.26, p = 0.024
CNAG_02177	6	990701	a	upstream	IL6	0.69 (0.5,0.95)	est = -0.37, error = 0.16, z = -2.3, p = 0.021
CNAG_02177	6	990701	a	upstream	IL10	0.28 (0.11,0.76)	est = -1.26, error = 0.5, z = -2.49, p = 0.013
CNAG_02475	6	221275	b	upstream	growth	9270592642782.33 (874.43,98286070453695393300480)	est = 29.86, error = 11.78, z = 2.54, p = 0.011
CNAG_02475	6	221282	b	upstream	growth	695429.76 (1.9,255059140770.7)	est = 13.45, error = 6.54, z = 2.06, p = 0.04
CNAG_02475	6	221273	ab	upstream	IL7	2.93 (1.03,8.3)	est = 1.07, error = 0.53, z = 2.02, p = 0.043
CNAG_02475	6	221273	ab	upstream	growth	2488091875835.39 (290.99,21274349201601838710784)	est = 28.54, error = 11.67, z = 2.45, p = 0.014
CNAG_02798	3	750294	a	upstream	cd4	1.03 (1,1.06)	est = 0.03, error = 0.01, z = 2.03, p = 0.042
CNAG_02798	3	750294	a	upstream	AMP	NA	est = -3.44, error = 1.74, z = -1.98, p = 0.048
CNAG_04100	9	7729	b	upstream	growth	62151.83 (1.37,2829792631.21)	est = 11.04, error = 5.47, z = 2.02, p = 0.044
CNAG_04100	9	5213	ab	upstream	adherence	90.72 (1.29,6383.5)	est = 4.51, error = 2.17, z = 2.08, p = 0.038
CNAG_04100	9	5213	ab	upstream	FLC	1.05 (1,1.1)	est = 0.05, error = 0.02, z = 2.05, p = 0.041
CNAG_04100	9	5213	ab	upstream	SERT	NA	est = -0.41, error = 0.17, z = -2.39, p = 0.017
CNAG_04100	9	8171	ab	upstream	efa	0 (0,0.28)	est = -26.89, error = 13.08, z = -2.06, p = 0.04
CNAG_04100	9	8171	ab	upstream	SERT	NA	est = -0.32, error = 0.16, z = -2.06, p = 0.04
CNAG_04102	9	10033	a	UTR-3	GMCSF	0.28 (0.09,0.87)	est = -1.26, error = 0.57, z = -2.2, p = 0.028
CNAG_04102	9	10033	a	UTR-3	efa	0 (0,0.03)	est = -35.91, error = 16.55, z = -2.17, p = 0.03
CNAG_04373	9	706175	b	upstream	Survival	1.0064 (1.0015,1.0113)	est = 0.01, error = 0, z = 2.55, p = 0.011
CNAG_04373	9	705343	ab	upstream	IL8	1.97 (1.05,3.69)	est = 0.68, error = 0.32, z = 2.11, p = 0.035
CNAG_04373	9	705343	ab	upstream	efa	614781858952922 (682.99,553385113861605342717149184)	est = 34.05, error = 14.04, z = 2.42, p = 0.015
CNAG_04535	9	1115286	a	upstream	IL17	0.35 (0.15,0.85)	est = -1.05, error = 0.45, z = -2.33, p = 0.02
CNAG_04535	9	1115286	a	upstream	GCSF	5.34 (1.13,25.21)	est = 1.67, error = 0.79, z = 2.11, p = 0.035
CNAG_04535	9	1115286	a	upstream	LFA_Titer	7.78 (1.01,60.03)	est = 2.05, error = 1.04, z = 1.97, p = 0.049
CNAG_04535	9	1115286	a	upstream	protein	0.9998 (0.9996,1)	est = 0, error = 0, z = -2.01, p = 0.044
CNAG_04922	10	18908	b	upstream	IL2	0.61 (0.4,0.95)	est = -0.49, error = 0.22, z = -2.2, p = 0.028
CNAG_04922	10	18915	b	upstream	IL2	0.61 (0.4,0.95)	est = -0.49, error = 0.22, z = -2.2, p = 0.028
CNAG_04922	10	18933	b	upstream	IL2	0.63 (0.41,0.97)	est = -0.46, error = 0.22, z = -2.11, p = 0.035
CNAG_04922	10	18941	b	upstream	IL2	0.63 (0.41,0.97)	est = -0.46, error = 0.22, z = -2.09, p = 0.036
CNAG_05185	4	667446	b	UTR-5	uptake	NA	est = -3.43, error = 1.49, z = -2.3, p = 0.021
CNAG_05185	4	667433	ab	UTR-5	Survival	0.9946 (0.9899,0.9993)	est = -0.01, error = 0, z = -2.25, p = 0.024
CNAG_05185	4	667433	ab	UTR-5	uptake	NA	est = -3.76, error = 1.55, z = -2.42, p = 0.016
CNAG_05450	14	342562	a	ns	IL6	0.75 (0.56,0.99)	est = -0.29, error = 0.15, z = -2, p = 0.046
CNAG_05450	14	342562	a	ns	IL7	0.32 (0.11,0.91)	est = -1.15, error = 0.54, z = -2.14, p = 0.032
CNAG_05450	14	342562	a	ns	IL12	0.53 (0.3,0.94)	est = -0.63, error = 0.29, z = -2.19, p = 0.028
CNAG_05450	14	342562	a	ns	IL13	0.74 (0.57,0.96)	est = -0.3, error = 0.13, z = -2.28, p = 0.023
CNAG_05450	14	342562	a	ns	GCSF	12.55 (1.94,81.35)	est = 2.53, error = 0.95, z = 2.65, p = 0.008
CNAG_05450	14	342562	a	ns	MIP1b	0.31 (0.13,0.75)	est = -1.17, error = 0.45, z = -2.58, p = 0.01
CNAG_05661	14	909638	b	upstream	adherence	87.56 (1.76,4353.6)	est = 4.47, error = 1.99, z = 2.24, p = 0.025
CNAG_05661	14	910152	b	upstream	uptake	NA	est = -3.29, error = 1.51, z = -2.17, p = 0.03

CNAG_05661	14	908850	ab	upstream	IL8	0.54 (0.3,0.97)	est = -0.62, error = 0.3, z = -2.06, p = 0.039
CNAG_05661	14	908850	ab	upstream	GMCSF	0.39 (0.17,0.89)	est = -0.95, error = 0.42, z = -2.25, p = 0.024
CNAG_05661	14	908850	ab	upstream	IFNg	0.61 (0.37,0.98)	est = -0.5, error = 0.24, z = -2.05, p = 0.041
CNAG_05661	14	908850	ab	upstream	MCP1	0.64 (0.43,0.95)	est = -0.44, error = 0.2, z = -2.2, p = 0.028
CNAG_05661	14	909011	ab	upstream	IL1b	2.76 (1.06,7.22)	est = 1.02, error = 0.49, z = 2.08, p = 0.038
CNAG_05661	14	909011	ab	upstream	IL8	2.43 (1.05,5.63)	est = 0.89, error = 0.43, z = 2.07, p = 0.038
CNAG_05661	14	909011	ab	upstream	MIP1b	5.04 (1.16,21.9)	est = 1.62, error = 0.75, z = 2.16, p = 0.031
CNAG_05661	14	909011	ab	upstream	uptake	156.75 (1.71,14336.35)	est = 5.05, error = 2.3, z = 2.19, p = 0.028
CNAG_05661	14	909011	ab	upstream	FLC	1.05 (1,1.1)	est = 0.05, error = 0.02, z = 2.01, p = 0.045
CNAG_05661	14	910181	ab	upstream	IL1b	0.42 (0.19,0.96)	est = -0.86, error = 0.42, z = -2.05, p = 0.04
CNAG_05661	14	910181	ab	upstream	IL6	0.73 (0.54,0.99)	est = -0.32, error = 0.15, z = -2.05, p = 0.04
CNAG_05661	14	910181	ab	upstream	IFNg	0.49 (0.26,0.93)	est = -0.72, error = 0.33, z = -2.19, p = 0.028
CNAG_05661	14	910181	ab	upstream	hivrna	1 (1,1)	est = 0, error = 0, z = 2.16, p = 0.031
CNAG_05662	14	910742	b	UTR-3	AMP	12.92 (1,166.43)	est = 2.56, error = 1.3, z = 1.96, p = 0.05
CNAG_05662	14	910834	b	downstream	Survival	0.994 (0.9885,0.9996)	est = -0.01, error = 0, z = -2.12, p = 0.034
CNAG_05662	14	910926	b	downstream	SERT	NA	est = -0.36, error = 0.18, z = -1.99, p = 0.046
CNAG_05662	14	910966	b	downstream	Survival	0.9933 (0.9885,0.998)	est = -0.01, error = 0, z = -2.77, p = 0.006
CNAG_05662	14	911262	b	downstream	MCP1	1.63 (1.02,2.59)	est = 0.49, error = 0.24, z = 2.06, p = 0.039
CNAG_05662	14	911292	b	downstream	IL2	1.66 (1.01,2.74)	est = 0.51, error = 0.25, z = 2, p = 0.045
CNAG_05662	14	911308	b	downstream	adherence	227.24 (1.95,26486)	est = 5.43, error = 2.43, z = 2.23, p = 0.025
CNAG_05662	14	911321	b	downstream	IL5	1.95 (1.02,3.74)	est = 0.67, error = 0.33, z = 2.02, p = 0.043
CNAG_05662	14	911352	b	downstream	MCP1	0.67 (0.45,1)	est = -0.4, error = 0.2, z = -1.98, p = 0.048
CNAG_05662	14	910822	ab	downstream	Survival	0.994 (0.989,0.9991)	est = -0.01, error = 0, z = -2.32, p = 0.02
CNAG_05662	14	910822	ab	downstream	FLC	1.05 (1,1.1)	est = 0.05, error = 0.02, z = 1.98, p = 0.047
CNAG_05662	14	910939	ab	downstream	growth	6808134.83 (3.38,13731197477679.4)	est = 15.73, error = 7.41, z = 2.12, p = 0.034
CNAG_05662	14	910939	ab	downstream	SERT	NA	est = -0.5, error = 0.25, z = -2.01, p = 0.044
CNAG_05662	14	910964	ab	downstream	Survival	0.9935 (0.9887,0.9982)	est = -0.01, error = 0, z = -2.68, p = 0.007
CNAG_05662	14	910964	ab	downstream	AMP	NA	est = -2.75, error = 1.39, z = -1.97, p = 0.048
CNAG_05662	14	910979	ab	downstream	Survival	0.9951 (0.9903,0.9999)	est = 0, error = 0, z = -2.01, p = 0.045
CNAG_05662	14	910979	ab	downstream	uptake	NA	est = -3.3, error = 1.6, z = -2.06, p = 0.04
CNAG_05662	14	911099	ab	downstream	IL12	1.97 (1.03,3.78)	est = 0.68, error = 0.33, z = 2.04, p = 0.041
CNAG_05662	14	911099	ab	downstream	GMCSF	2.36 (1.01,5.54)	est = 0.86, error = 0.43, z = 1.98, p = 0.048
CNAG_05662	14	911099	ab	downstream	MCP1	1.66 (1.08,2.55)	est = 0.51, error = 0.22, z = 2.32, p = 0.021
CNAG_05662	14	911099	ab	downstream	TNFa	2.15 (1.03,4.47)	est = 0.76, error = 0.37, z = 2.04, p = 0.042
CNAG_05662	14	911099	ab	downstream	growth	334412.19 (1.67,67113909729.92)	est = 12.72, error = 6.23, z = 2.04, p = 0.041
CNAG_05662	14	911129	ab	downstream	IL12	2.32 (1.18,4.55)	est = 0.84, error = 0.34, z = 2.44, p = 0.015
CNAG_05662	14	911129	ab	downstream	IL13	1.36 (1.04,1.78)	est = 0.31, error = 0.14, z = 2.26, p = 0.024
CNAG_05662	14	911129	ab	downstream	IL17	2.67 (1.13,6.33)	est = 0.98, error = 0.44, z = 2.23, p = 0.026
CNAG_05662	14	911129	ab	downstream	MIP1b	2.1 (1.04,4.26)	est = 0.74, error = 0.36, z = 2.06, p = 0.039
CNAG_05662	14	911129	ab	downstream	TNFa	2.26 (1.14,4.47)	est = 0.82, error = 0.35, z = 2.35, p = 0.019
CNAG_05662	14	911129	ab	downstream	growth	168095118.56 (19.27,1466671649037040)	est = 18.94, error = 8.15, z = 2.32, p = 0.02
CNAG_05662	14	911129	ab	downstream	FLC	1.08 (1.01,1.15)	est = 0.07, error = 0.03, z = 2.11, p = 0.035
CNAG_05662	14	911129	ab	downstream	AMP	NA	est = -2.7, error = 1.36, z = -1.98, p = 0.048
CNAG_05662	14	911129	ab	downstream	SERT	NA	est = -0.55, error = 0.25, z = -2.25, p = 0.025
CNAG_05662	14	911206	ab	downstream	IL8	1.9 (1.01,3.57)	est = 0.64, error = 0.32, z = 1.98, p = 0.048

CNAG_05662	14	911206	ab	downstream	MCP1	1.87 (1.17,3)	est = 0.63, error = 0.24, z = 2.6, p = 0.009
CNAG_05662	14	911206	ab	downstream	MIP1b	2.15 (1.01,4.58)	est = 0.77, error = 0.38, z = 1.99, p = 0.046
CNAG_05663	14	910323	b	downstream	TNFa	0.5 (0.27,0.92)	est = -0.69, error = 0.31, z = -2.22, p = 0.026
CNAG_05663	14	910555	b	downstream	Survival	0.9951 (0.9905,0.9998)	est = 0, error = 0, z = -2.06, p = 0.039
CNAG_05663	14	910328	ab	downstream	IL1b	0.43 (0.19,0.97)	est = -0.84, error = 0.41, z = -2.04, p = 0.041
CNAG_05663	14	910328	ab	downstream	IL13	0.75 (0.57,0.99)	est = -0.28, error = 0.14, z = -2, p = 0.045
CNAG_05663	14	910328	ab	downstream	TNFa	0.45 (0.23,0.86)	est = -0.8, error = 0.33, z = -2.42, p = 0.015
CNAG_05913	7	1205599	ab	upstream	MIP1b	2.26 (1.04,4.92)	est = 0.82, error = 0.4, z = 2.07, p = 0.039
CNAG_05913	7	1205599	ab	upstream	adherence	89.41 (1.77,4515.37)	est = 4.49, error = 2, z = 2.25, p = 0.025
CNAG_05913	7	1205600	ab	upstream	IL13	1.3 (1,1.69)	est = 0.26, error = 0.13, z = 1.98, p = 0.048
CNAG_05913	7	1205600	ab	upstream	IL17	2.36 (1.02,5.46)	est = 0.86, error = 0.43, z = 2.01, p = 0.044
CNAG_05913	7	1205600	ab	upstream	MIP1b	2.3 (1.05,5.05)	est = 0.83, error = 0.4, z = 2.08, p = 0.038
CNAG_05913	7	1205600	ab	upstream	adherence	86.82 (1.72,4382.67)	est = 4.46, error = 2, z = 2.23, p = 0.026
CNAG_05937	7	1263610	a	UTR-5	uptake	NA	est = -3.16, error = 1.52, z = -2.08, p = 0.038
CNAG_05937	7	1263610	a	UTR-5	SERT	1.38 (1.02,1.86)	est = 0.32, error = 0.15, z = 2.1, p = 0.036
CNAG_05987	12	14009	b	ns	IL2	0.58 (0.37,0.92)	est = -0.55, error = 0.23, z = -2.34, p = 0.019
CNAG_05987	12	14035	b	ns	IL2	1.54 (1.03,2.28)	est = 0.43, error = 0.2, z = 2.13, p = 0.034
CNAG_05987	12	15014	b	UTR-5	adherence	152.66 (1.58,14704.12)	est = 5.03, error = 2.33, z = 2.16, p = 0.031
CNAG_06169	12	502808	b	UTR-3	IL8	0.5 (0.26,0.96)	est = -0.7, error = 0.34, z = -2.09, p = 0.037
CNAG_06169	12	503311	b	UTR-3	GCSF	0.15 (0.03,0.79)	est = -1.92, error = 0.86, z = -2.23, p = 0.025
CNAG_06169	12	502888	ab	UTR-3	GMCSF	0.43 (0.19,0.98)	est = -0.84, error = 0.42, z = -2, p = 0.046
CNAG_06169	12	502888	ab	UTR-3	growth	637044.06 (1.59,254738795304.8)	est = 13.36, error = 6.58, z = 2.03, p = 0.042
CNAG_06169	12	502890	ab	UTR-3	IL6	0.69 (0.49,0.98)	est = -0.37, error = 0.18, z = -2.09, p = 0.037
CNAG_06169	12	502890	ab	UTR-3	IL8	0.52 (0.27,1)	est = -0.65, error = 0.33, z = -1.97, p = 0.049
CNAG_06169	12	502890	ab	UTR-3	GMCSF	0.36 (0.14,0.91)	est = -1.03, error = 0.47, z = -2.17, p = 0.03
CNAG_06169	12	503049	ab	UTR-3	GMCSF	2.7 (1.06,6.89)	est = 0.99, error = 0.48, z = 2.08, p = 0.037
CNAG_06169	12	503049	ab	UTR-3	hivrna	1 (1,1)	est = 0, error = 0, z = 2.19, p = 0.028
CNAG_06169	12	503112	ab	UTR-3	hivrna	1 (1,1)	est = 0, error = 0, z = -2.16, p = 0.031
CNAG_06169	12	503112	ab	UTR-3	csf_wbc	1.02 (1,1.04)	est = 0.02, error = 0.01, z = 2.2, p = 0.028
CNAG_06169	12	503313	ab	UTR-3	IL12	1.78 (1.02,3.1)	est = 0.58, error = 0.28, z = 2.03, p = 0.042
CNAG_06169	12	503313	ab	UTR-3	IL13	1.32 (1.01,1.72)	est = 0.28, error = 0.14, z = 2.04, p = 0.041
CNAG_06169	12	503313	ab	UTR-3	GCSF	0.14 (0.03,0.77)	est = -1.97, error = 0.87, z = -2.26, p = 0.024
CNAG_06169	12	503321	ab	UTR-3	IL12	2.44 (1.22,4.87)	est = 0.89, error = 0.35, z = 2.52, p = 0.012
CNAG_06169	12	503321	ab	UTR-3	IL13	1.37 (1.04,1.8)	est = 0.31, error = 0.14, z = 2.23, p = 0.026
CNAG_06169	12	503321	ab	UTR-3	GCSF	0.15 (0.03,0.84)	est = -1.92, error = 0.89, z = -2.15, p = 0.031
CNAG_06169	12	503321	ab	UTR-3	MIP1b	2.27 (1.03,4.97)	est = 0.82, error = 0.4, z = 2.04, p = 0.041
CNAG_06169	12	503327	ab	UTR-3	IL12	2.25 (1.15,4.41)	est = 0.81, error = 0.34, z = 2.38, p = 0.017
CNAG_06169	12	503327	ab	UTR-3	IL13	1.31 (1.01,1.7)	est = 0.27, error = 0.13, z = 2.01, p = 0.044
CNAG_06169	12	503327	ab	UTR-3	MIP1b	2.17 (1,4.68)	est = 0.77, error = 0.39, z = 1.97, p = 0.049
CNAG_06169	12	503401	ab	UTR-3	IL10	2.85 (1.15,7.08)	est = 1.05, error = 0.46, z = 2.26, p = 0.024
CNAG_06169	12	503401	ab	UTR-3	chitin	1.0007 (1,1.0014)	est = 0, error = 0, z = 2.02, p = 0.044
CNAG_06256	13	11130	b	upstream	TNFa	0.42 (0.21,0.84)	est = -0.87, error = 0.35, z = -2.45, p = 0.014
CNAG_06256	13	11118	ab	upstream	IFNg	0.6 (0.38,0.97)	est = -0.51, error = 0.24, z = -2.1, p = 0.036
CNAG_06256	13	11118	ab	upstream	TNFa	0.45 (0.22,0.91)	est = -0.8, error = 0.36, z = -2.21, p = 0.027
CNAG_06332	13	219311	b	upstream	efa	119853562388238492018898108416 (6907942.89,2.07947237642355e+51)	est = 66.96, error = 26.13, z = 2.56, p = 0.01

CNAG_06332	13	219312	b	upstream	efa	1.60006999119291e+35 (29822873.86,8.58476613812001e+62)	est = 81.06, error = 32.58, z = 2.49, p = 0.013
CNAG_06422	13	436551	b	UTR-5	IL2	1.5 (1.02,2.19)	est = 0.4, error = 0.19, z = 2.06, p = 0.039
CNAG_06422	13	436554	b	UTR-5	IL2	1.5 (1.02,2.19)	est = 0.4, error = 0.19, z = 2.06, p = 0.039
CNAG_06490	13	655915	a	frameshift	hivrna	1 (1,1)	est = 0, error = 0, z = 2.5, p = 0.012
CNAG_06490	13	655915	a	frameshift	cd4	1.06 (1,1.12)	est = 0.06, error = 0.03, z = 2.12, p = 0.034
CNAG_06525	7	11056	ab	ns	IL5	2.2 (1.09,4.46)	est = 0.79, error = 0.36, z = 2.19, p = 0.028
CNAG_06525	7	11056	ab	ns	IL10	2.7 (1.1,6.67)	est = 0.99, error = 0.46, z = 2.16, p = 0.031
CNAG_06525	7	14006	ab	upstream	IL6	0.69 (0.49,0.99)	est = -0.36, error = 0.18, z = -2.03, p = 0.043
CNAG_06525	7	14006	ab	upstream	IL8	0.49 (0.25,0.97)	est = -0.71, error = 0.35, z = -2.04, p = 0.041
CNAG_06574	7	164473	b	upstream	hivrna	1 (1,1)	est = 0, error = 0, z = -1.98, p = 0.047
CNAG_06574	7	165027	b	upstream	MIP1b	0.4 (0.18,0.89)	est = -0.92, error = 0.41, z = -2.24, p = 0.025
CNAG_06574	7	165873	b	upstream	IL13	1.34 (1.02,1.77)	est = 0.29, error = 0.14, z = 2.07, p = 0.039
CNAG_06574	7	166309	b	upstream	growth	6311961.93 (2.64,15118988811853.8)	est = 15.66, error = 7.49, z = 2.09, p = 0.037
CNAG_06574	7	167135	b	upstream	IL13	0.69 (0.51,0.92)	est = -0.38, error = 0.15, z = -2.54, p = 0.011
CNAG_06574	7	167224	b	upstream	GMCSF	0.35 (0.13,0.9)	est = -1.06, error = 0.49, z = -2.17, p = 0.03
CNAG_06574	7	164887	ab	upstream	IL2	1.64 (1.03,2.61)	est = 0.49, error = 0.24, z = 2.08, p = 0.038
CNAG_06574	7	164887	ab	upstream	TNFa	0.53 (0.29,0.97)	est = -0.63, error = 0.31, z = -2.05, p = 0.04
CNAG_06574	7	164926	ab	upstream	IL2	0.56 (0.33,0.95)	est = -0.58, error = 0.27, z = -2.16, p = 0.031
CNAG_06574	7	164926	ab	upstream	MIP1b	2.18 (1.07,4.43)	est = 0.78, error = 0.36, z = 2.15, p = 0.031
CNAG_06574	7	165704	ab	upstream	Survival	1.0048 (1.0001,1.0096)	est = 0, error = 0, z = 2, p = 0.045
CNAG_06574	7	165704	ab	upstream	efa	0 (0,0.4)	est = -25.89, error = 12.75, z = -2.03, p = 0.042
CNAG_06574	7	167292	ab	upstream	IL1b	0.43 (0.19,0.98)	est = -0.84, error = 0.42, z = -2.01, p = 0.045
CNAG_06574	7	167292	ab	upstream	GCSF	11.82 (1.55,89.87)	est = 2.47, error = 1.04, z = 2.39, p = 0.017
CNAG_06574	7	167292	ab	upstream	MIP1b	0.3 (0.11,0.82)	est = -1.19, error = 0.51, z = -2.35, p = 0.019
CNAG_06574	7	167292	ab	upstream	uptake	NA	est = -3.5, error = 1.58, z = -2.22, p = 0.027
CNAG_06574	7	167370	ab	upstream	cd4	1.09 (1.01,1.18)	est = 0.09, error = 0.04, z = 2.23, p = 0.026
CNAG_06574	7	167370	ab	upstream	uptake	NA	est = -4.03, error = 1.67, z = -2.42, p = 0.016
CNAG_06704	2	270700	a	upstream	IL2	1.45 (1.01,2.09)	est = 0.37, error = 0.18, z = 2.03, p = 0.042
CNAG_06704	2	270700	a	upstream	protein	0.9998 (0.9996,1)	est = 0, error = 0, z = -2.2, p = 0.028
CNAG_06876	5	7093	a	UTR-3	IFNg	0.53 (0.29,0.96)	est = -0.64, error = 0.3, z = -2.11, p = 0.035
CNAG_06876	5	7093	a	UTR-3	MIP1b	0.44 (0.21,0.95)	est = -0.81, error = 0.39, z = -2.08, p = 0.037
CNAG_06876	5	7093	a	UTR-3	TNFa	0.47 (0.24,0.93)	est = -0.75, error = 0.35, z = -2.16, p = 0.031
CNAG_06968	8	1383765	a	CODON_INSERTION	IL12	0.56 (0.32,0.97)	est = -0.58, error = 0.28, z = -2.07, p = 0.038
CNAG_06968	8	1383765	a	CODON_INSERTION	IL17	0.45 (0.21,0.97)	est = -0.8, error = 0.39, z = -2.05, p = 0.041
CNAG_07026	12	11092	ab	upstream	IL1b	2.34 (1.04,5.26)	est = 0.85, error = 0.41, z = 2.06, p = 0.039
CNAG_07026	12	11092	ab	upstream	IL13	1.34 (1.01,1.77)	est = 0.29, error = 0.14, z = 2.01, p = 0.045
CNAG_07026	12	11092	ab	upstream	Survival	1.0083 (1.0013,1.0153)	est = 0.01, error = 0, z = 2.32, p = 0.02
CNAG_07026	12	11092	ab	upstream	efa	0 (0,0.2)	est = -27.82, error = 13.37, z = -2.08, p = 0.037
CNAG_07026	12	11094	ab	upstream	IL13	1.4 (1.02,1.93)	est = 0.34, error = 0.16, z = 2.06, p = 0.04
CNAG_07026	12	11094	ab	upstream	LFA_Titer	0.43 (0.19,0.99)	est = -0.84, error = 0.42, z = -1.99, p = 0.047
CNAG_07026	12	11094	ab	upstream	Survival	1.0075 (1.0001,1.0149)	est = 0.01, error = 0, z = 2, p = 0.046
CNAG_07026	12	11400	ab	upstream	IL1b	0.37 (0.15,0.93)	est = -1, error = 0.47, z = -2.12, p = 0.034
CNAG_07026	12	11400	ab	upstream	IL7	0.27 (0.07,0.98)	est = -1.32, error = 0.67, z = -1.99, p = 0.047
CNAG_07026	12	11400	ab	upstream	IL13	0.71 (0.51,1)	est = -0.34, error = 0.17, z = -1.96, p = 0.05
CNAG_07026	12	11400	ab	upstream	LFA_Titer	3.91 (1.23,12.37)	est = 1.36, error = 0.59, z = 2.32, p = 0.021

CNAG_07026	12	11406	ab	upstream	IL1b	0.37 (0.15,0.93)	est = -1, error = 0.47, z = -2.12, p = 0.034
CNAG_07026	12	11406	ab	upstream	IL7	0.27 (0.07,0.98)	est = -1.32, error = 0.67, z = -1.99, p = 0.047
CNAG_07026	12	11406	ab	upstream	IL13	0.71 (0.51,1)	est = -0.34, error = 0.17, z = -1.96, p = 0.05
CNAG_07026	12	11406	ab	upstream	LFA_Titer	3.91 (1.23,12.37)	est = 1.36, error = 0.59, z = 2.32, p = 0.021
CNAG_07026	12	11407	ab	upstream	IL1b	0.37 (0.15,0.93)	est = -1, error = 0.47, z = -2.12, p = 0.034
CNAG_07026	12	11407	ab	upstream	IL7	0.27 (0.07,0.98)	est = -1.32, error = 0.67, z = -1.99, p = 0.047
CNAG_07026	12	11407	ab	upstream	IL13	0.71 (0.51,1)	est = -0.34, error = 0.17, z = -1.96, p = 0.05
CNAG_07026	12	11407	ab	upstream	LFA_Titer	3.91 (1.23,12.37)	est = 1.36, error = 0.59, z = 2.32, p = 0.021
CNAG_07026	12	11410	ab	upstream	IL1b	0.37 (0.15,0.93)	est = -1, error = 0.47, z = -2.12, p = 0.034
CNAG_07026	12	11410	ab	upstream	IL7	0.27 (0.07,0.98)	est = -1.32, error = 0.67, z = -1.99, p = 0.047
CNAG_07026	12	11410	ab	upstream	IL13	0.71 (0.51,1)	est = -0.34, error = 0.17, z = -1.96, p = 0.05
CNAG_07026	12	11410	ab	upstream	LFA_Titer	3.91 (1.23,12.37)	est = 1.36, error = 0.59, z = 2.32, p = 0.021
CNAG_07026	12	11413	ab	upstream	IL1b	0.38 (0.15,0.95)	est = -0.98, error = 0.47, z = -2.08, p = 0.038
CNAG_07026	12	11413	ab	upstream	LFA_Titer	3.91 (1.23,12.37)	est = 1.36, error = 0.59, z = 2.32, p = 0.021
CNAG_07703	7	1341024	a	ns	IL6	0.63 (0.43,0.91)	est = -0.47, error = 0.19, z = -2.42, p = 0.015
CNAG_07703	7	1341024	a	ns	IL8	0.48 (0.24,0.94)	est = -0.74, error = 0.34, z = -2.15, p = 0.032
CNAG_07837	10	13558	b	UTR-5	IL2	1.5 (1.02,2.19)	est = 0.4, error = 0.19, z = 2.06, p = 0.039
CNAG_07837	10	15302	b	UTR-3	cd4	0.83 (0.68,1)	est = -0.19, error = 0.1, z = -1.96, p = 0.049
CNAG_07950	1	975397	b	upstream	efa	0 (0,0.01)	est = -31.27, error = 13.51, z = -2.32, p = 0.021
CNAG_07950	1	975152	ab	upstream	IL8	2.67 (1.12,6.34)	est = 0.98, error = 0.44, z = 2.23, p = 0.026
CNAG_07950	1	975152	ab	upstream	hivrna	1 (1,1)	est = 0, error = 0, z = 1.98, p = 0.048
CNAG_07950	1	975212	ab	upstream	IL4	0.45 (0.23,0.92)	est = -0.79, error = 0.36, z = -2.21, p = 0.027
CNAG_07950	1	975212	ab	upstream	IL6	0.65 (0.47,0.91)	est = -0.43, error = 0.17, z = -2.48, p = 0.013
CNAG_07950	1	975212	ab	upstream	IL8	0.32 (0.14,0.75)	est = -1.13, error = 0.43, z = -2.62, p = 0.009
CNAG_07950	1	975212	ab	upstream	GMCSF	0.4 (0.17,0.92)	est = -0.92, error = 0.42, z = -2.17, p = 0.03
CNAG_07950	1	975212	ab	upstream	IFNg	0.51 (0.28,0.93)	est = -0.67, error = 0.31, z = -2.18, p = 0.029
CNAG_07950	1	975212	ab	upstream	FLC	NA	est = -0.06, error = 0.03, z = -2.11, p = 0.034
CNAG_08006	11	804710	ab	UTR-5	IL4	0.41 (0.18,0.94)	est = -0.89, error = 0.42, z = -2.11, p = 0.035
CNAG_08006	11	804710	ab	UTR-5	IL5	0.32 (0.13,0.84)	est = -1.13, error = 0.48, z = -2.33, p = 0.02
CNAG_08006	11	804710	ab	UTR-5	IL6	0.71 (0.51,0.99)	est = -0.34, error = 0.17, z = -2.02, p = 0.043
CNAG_08006	11	804710	ab	UTR-5	MIP1b	0.39 (0.16,0.97)	est = -0.93, error = 0.46, z = -2.04, p = 0.042
CNAG_08006	11	804710	ab	UTR-5	TNFa	0.34 (0.14,0.84)	est = -1.07, error = 0.46, z = -2.35, p = 0.019
CNAG_08006	11	804710	ab	UTR-5	adherence	NA	est = -4.63, error = 2.32, z = -1.99, p = 0.046
CNAG_08006	11	804710	ab	UTR-5	chitin	NA	est = 0, error = 0, z = -1.99, p = 0.047
CNAG_08006	11	804742	ab	UTR-5	IL4	0.49 (0.25,0.96)	est = -0.72, error = 0.34, z = -2.08, p = 0.038
CNAG_08006	11	804742	ab	UTR-5	IFNg	0.6 (0.37,0.97)	est = -0.51, error = 0.25, z = -2.09, p = 0.036
CNAG_08006	11	804742	ab	UTR-5	MCP1	0.57 (0.34,0.95)	est = -0.56, error = 0.26, z = -2.16, p = 0.031
CNAG_08006	11	804742	ab	UTR-5	adherence	NA	est = -6.75, error = 2.65, z = -2.54, p = 0.011
CNAG_12610	7	49744	a	upstream	MCP1	0.65 (0.43,0.98)	est = -0.43, error = 0.21, z = -2.06, p = 0.039
CNAG_12610	7	49744	a	upstream	uptake	NA	est = -3.86, error = 1.77, z = -2.19, p = 0.029
CNAG_13108	13	128625	ab	upstream	IL13	0.74 (0.55,1)	est = -0.3, error = 0.15, z = -1.97, p = 0.049
CNAG_13108	13	128625	ab	upstream	GCSF	8.41 (1.28,55.01)	est = 2.13, error = 0.96, z = 2.22, p = 0.026
CNAG_13108	13	128715	ab	upstream	IL13	0.59 (0.39,0.89)	est = -0.53, error = 0.21, z = -2.55, p = 0.011
CNAG_13108	13	128715	ab	upstream	GCSF	8.04 (1.29,50.02)	est = 2.08, error = 0.93, z = 2.23, p = 0.025
CNAG_13108	13	128729	ab	upstream	IL13	0.65 (0.43,0.96)	est = -0.44, error = 0.2, z = -2.16, p = 0.031

CNAG_13108	13	128729	ab	upstream	GCSF	6.7 (1.06,42.24)	est = 1.9, error = 0.94, z = 2.02, p = 0.043
CNAG_13204	14	924025	b	upstream	GMCSF	3.18 (1.22,8.29)	est = 1.16, error = 0.49, z = 2.37, p = 0.018
CNAG_13204	14	924047	b	upstream	IL13	1.41 (1.06,1.86)	est = 0.34, error = 0.14, z = 2.4, p = 0.017
CNAG_13204	14	924049	b	upstream	IL13	1.41 (1.06,1.86)	est = 0.34, error = 0.14, z = 2.4, p = 0.017
CNAG_13204	14	924050	b	upstream	IL13	1.41 (1.06,1.86)	est = 0.34, error = 0.14, z = 2.4, p = 0.017
