# Supplementary material for: Identification of Pathogen Genomic Differences That Impact Human Immune Response and Disease during Cryptococcus neoformans Infection
Source: mBio. 2019 Jul 16;10(4):e01440-19. doi: 10.1128/mBio.01440-19 (PMC6635531; doi:10.1128/mBio.01440-19)
Supplement: TABLE S7 [file mBio.01440-19-st007.pdf]

Table S7. The majority of genes associated with quantitative infection phenotypes are uncharacterized

Gene	Class	Orthology	Description	Significant PCA	KO survival
CNAG_00014	b	basidio	hypothetical protein		
CNAG_00363	b	basidio	TCO6		no effect
CNAG_01241	ab	conserved	enzyme regulator	yes	
CNAG_01371	a	conserved	CRG2, regulator of G protein signaling		
CNAG_01802	b	conserved	DRE2		
CNAG_02112	b	basidio	ubiquitin-conjugating enzyme E2 W		
CNAG_02176	ab	C. gattii	hypothetical protein	yes	decreased
CNAG_02177	a	conserved	PP2A catalytic subunit		
CNAG_02475	ab	basidio	flavin-containing monooxygenase		
CNAG_02798	a	conserved	hypothetical protein		
CNAG_04100	ab	basidio	hypothetical		
CNAG_04102	a	C. gattii, Puccinia graminis	hypothetical protein		
CNAG_04179	a	conserved	lysine-tRNA ligase		
CNAG_04373	ab	basidio	alginate lyase		no effect
CNAG_04535	a	conserved	dehydrogenase		no effect
CNAG_04922	b	C.gattii	hypothetical protein		increased
CNAG_05185	ab	C.gattii	hypothetical protein		
CNAG_05450	a	Tremella, Melampsora larici-populina	hypothetical protein	yes	
CNAG_05661	ab	conserved	FACT complex subunit POB3	yes	
CNAG_05662	ab	conserved	ITR4	yes	increased
CNAG_05663	ab	conserved	WHI3/SCW1	yes	no effect
CNAG_05913	ab	conserved	alpha gluconsidase		no effect
CNAG_05937	ab	C.gattii	hypothetical protein		no effect
CNAG_05987	b	C.gattii	hypothetical protein	yes	
CNAG_06169	ab	conserved	hypothetical protein	yes	no effect
CNAG_06256	ab	basidio	hypothetical protein		
CNAG_06332	b	C. gattii, Tremella mesenterica	hypothetical protein		decreased
CNAG_06422	b	basidio	hypothetical protein		
CNAG_06490	a	C.gattii	CAMK/CAMKL protein kinase		no effect
CNAG_06525	ab	none	nuclear protein		
CNAG_06574	ab	C. gattii	APP1		decreased
CNAG_06704	a	basidio	hypothetical protein		

CNAG_06876	a	conserved	dioxygenase		no effect
CNAG_06968	a	conserved	co-chaperone GrpE		increased
CNAG_07026	ab	C.gattii	hypothetical protein		
CNAG_07727		none	hypothetical protein	yes	
CNAG_07703	a	C.gattii	hypothetical protein	yes	no effect
CNAG_07837	b	none	hypothetical protein		no effect
CNAG_07950	ab	C. gattii	hypothetical protein	yes	
CNAG_08006	ab	none	hypothetical protein	yes	
CNAG_12610	a	NA	hypothetical RNA		
CNAG_13108	ab	NA	hypothetical RNA		
CNAG_13204	b	NA	hypothetical RNA		
