# Supplementary material for: Identification of Pathogen Genomic Differences That Impact Human Immune Response and Disease during Cryptococcus neoformans Infection
Source: mBio. 2019 Jul 16;10(4):e01440-19. doi: 10.1128/mBio.01440-19 (PMC6635531; doi:10.1128/mBio.01440-19)
Supplement: FIG S2 [file mBio.01440-19-sf002.pdf]

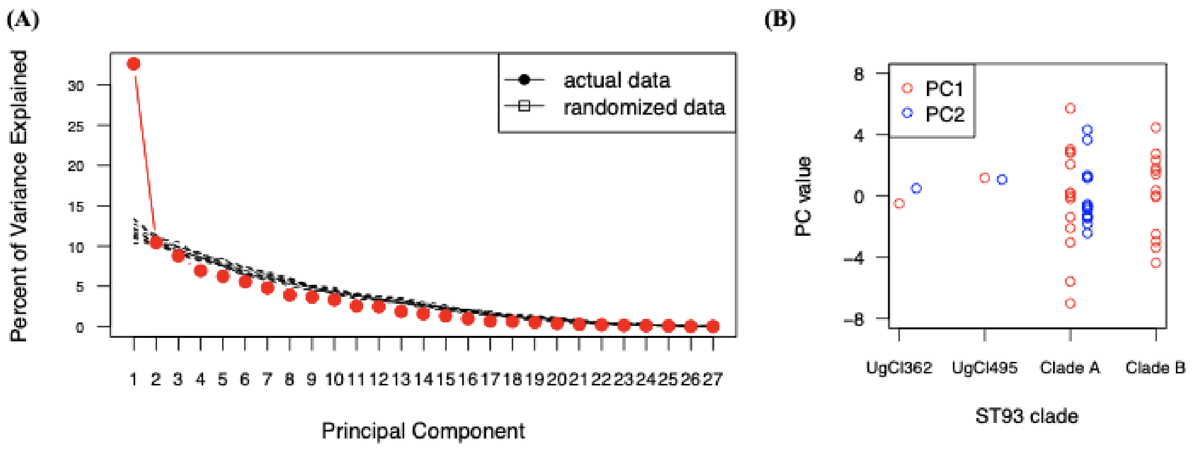

Figure S2. PCA analysis. A) Each dashed line represents one of 20 randomized trials. B) There was no association between PC1 or PC2 and clade.
