# Supplementary material for: Identification of Pathogen Genomic Differences That Impact Human Immune Response and Disease during Cryptococcus neoformans Infection
Source: mBio. 2019 Jul 16;10(4):e01440-19. doi: 10.1128/mBio.01440-19 (PMC6635531; doi:10.1128/mBio.01440-19)
Supplement: FIG S3 [file mBio.01440-19-sf003.pdf]

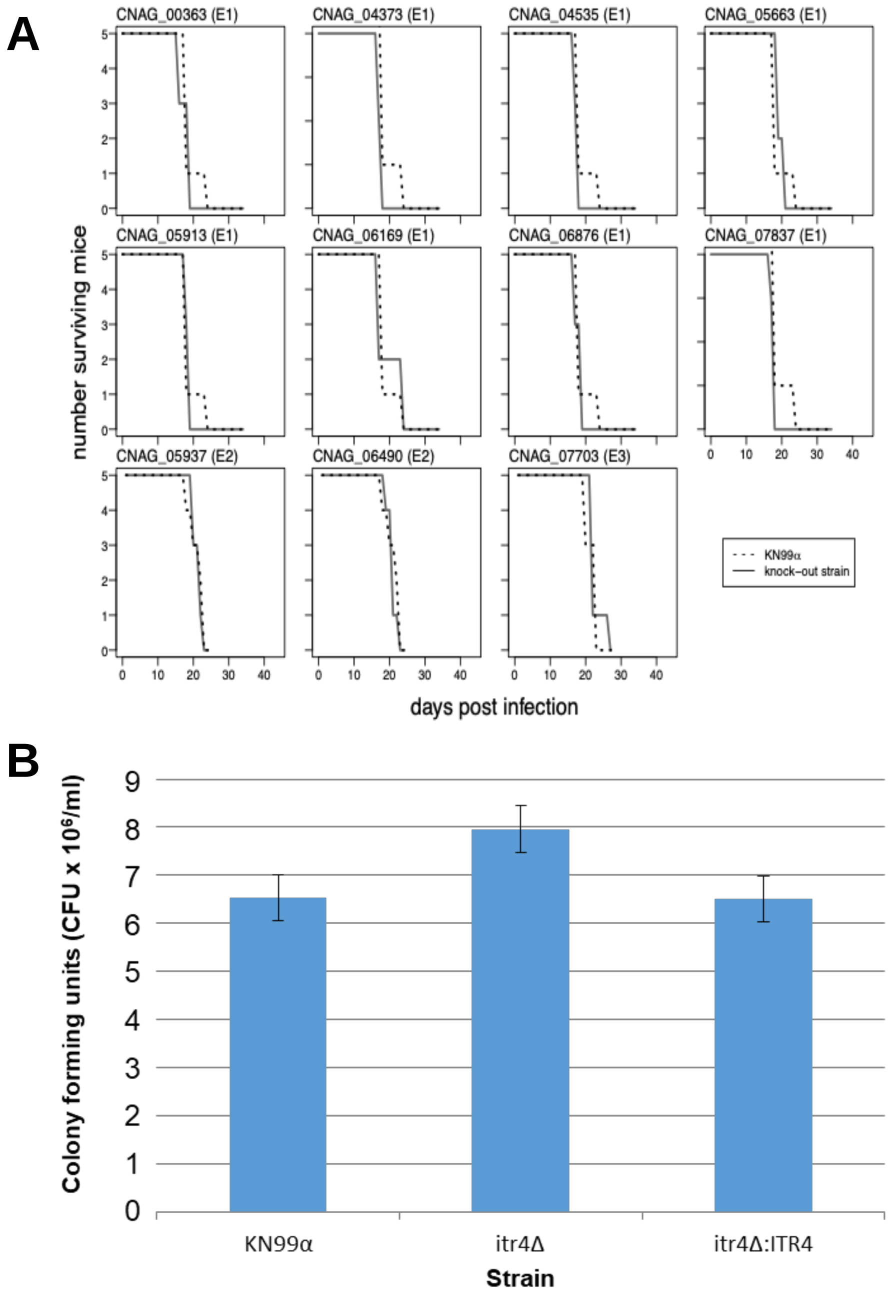

Figure S3. Deletion strain virulence in mice. A) Groups of five 6-8 week old C57Bl/6 mice were infected intranasally with 5 X 10^4 cells. Progression to severe morbidity was monitored for 35 days and mice were sacrificed when endpoint criteria were reached. Strains were tested in two separate experiments, E1 or E2, respectively. The deletions strains were compared against the KN99α strain in the same experiment. **B)** Groups of four 6-8 week old C57Bl/6 mice were infected intranasally with 1 X 10^3 cells. Mice were sacrificed at 7 days post infection, lungs homogenized in 4 ml of PBS, and serial dilutions plated on YPD with cholamphenicol medium. Colony forming units were enumerated at 48 hours.
